# NET-Associated Transcriptional Signatures and T-Cell Functional States During Allogeneic CAR-γδT-Cell Therapy: A Longitudinal Single-Cell Analysis

**DOI:** 10.3390/genes17101272

**Published:** 2026-10-13

**Authors:** Lingsong Yao, Zhiyu Liu, Ying Wang, Wanjun Gu, Yun-Fei Bai

**Affiliations:** 1 State Key Laboratory of Bioelectronics, School of Biological Science and Medical Engineering, Southeast University, Nanjing 210096, China; yaolsyy@foxmail.com (L.Y.);; 2 College of Rehabilitation and Physical Education, Nanjing Normal University of Special Education, Nanjing 210038, China; 15195986641@163.com; 3 Collaborative Innovation Center of Jiangsu Province of Cancer Prevention and Treatment of Chinese Medicine, Nanjing University of Chinese Medicine, Nanjing 210023, China; 4 Jiangsu Province Engineering Research Center of TCM Intelligence Health Service, Nanjing University of Chinese Medicine, Nanjing 210023, China; 5 School of Artificial Intelligence and Information Technology, Nanjing University of Chinese Medicine, Nanjing 210023, China

**Keywords:** CAR–γδT, NETs, lymphoma, neutrophils, immunotherapy

## Abstract

Background: CAR T-cell therapy has transformed the treatment of hematologic malignancies, although conventional autologous αβ CAR-T products remain limited by complex manufacturing, variable persistence, and relapse risk. γδT cells provide a promising donor source for next-generation off-the-shelf CAR-T therapies due to their MHC-independent tumor recognition, potent cytotoxicity, and minimal risk of graft-versus-host disease; however, the determinants of treatment durability following allogeneic CAR–γδT-cell therapy remain poorly defined. NETs have recently emerged as key immunoregulatory components of the TME, where excessive NET formation sustains inflammation, reshapes immune–stromal interactions, and impairs lymphocyte function, yet their impact on CAR-T-cell fate is largely unknown. Methods: In this study, we performed longitudinal single-cell transcriptomic profiling of patients treated with allogeneic CAR–γδT cells and integrated ligand–receptor inference, pathway enrichment, and transcriptional regulatory analyses. Results: We identified a 69-gene NET-associated transcriptional signature in the neutrophil population captured in PBMC samples. The NET-associated transcriptional score increased longitudinally in the single patient who subsequently relapsed, whereas longitudinal patterns varied across individual patients. Exploratory analyses identified associations between NET-associated transcriptional activity and T-cell functional programs, including an inverse association with proliferation. NicheNet, pathway enrichment, and SCENIC analyses highlighted candidate inflammatory and regulatory programs involving IL-1-related signaling, adhesion pathways, SMAD3 regulon activity, and E2F-related proliferative programs. Analysis of an independent cohort provided supportive evidence for related inflammatory transcriptional patterns, although differences in therapeutic platform and cellular composition limited direct validation. Discussion: Together, these findings suggest that NET-associated inflammatory states may be linked to peripheral immune remodeling and altered circulating T-cell functional states during CAR–γδT-cell therapy. Larger longitudinal cohorts, more comprehensive clinical annotation, and orthogonal measurements of NET formation are needed to establish the clinical and mechanistic significance of these associations.

## 1. Introduction

Cancer remains one of the leading causes of mortality worldwide. Therefore, new antitumor strategies need to be developed to reduce mortality and improve patients’ quality of life [1]. Compared with conventional radiotherapy and chemotherapy, which often produce substantial systemic toxicity, immunotherapy offers a more selective strategy by harnessing the immune system to recognize and eradicate malignant cells [2,3]. Among various immunotherapeutic approaches, CAR T-cell therapy has demonstrated remarkable clinical efficacy in hematologic malignancies. However, traditional autologous CAR-T manufacturing involves isolating patient-derived αβ T cells, followed by ex vivo genetic modification and expansion, resulting in high cost, long production cycles, and limited scalability [4,5]. Compared with αβT cells, γδT cells recognize tumor antigens independently of major histocompatibility complex (MHC)-mediated antigen presentation and exhibit an exceptionally low risk of graft-versus-host disease (GvHD) [6]. This feature enables the safe utilization of γδT cells in allogeneic therapeutic applications. Thus, γδT cells constitute a highly suitable donor population for the development of off-the-shelf CAR-T-cell therapies [7]. However, despite these advantages, CAR-γδT-cell therapy still faces several challenges. Limitations include suboptimal in vivo persistence, inconsistent expansion efficiency, and the risk of tumor relapse due to antigen loss or insufficient clearance [8,9]. Addressing these issues is critical for improving the therapeutic efficacy of CAR-γδT cells.

The TME is a major determinant of CAR-T-cell persistence and therapeutic durability [10,11]. Over the past decade, the role of neutrophils in cancer biology has gained increasing attention. They are now recognized as critical components of the TME and exert functional influences across multiple stages of cancer progression [12,13,14]. Neutrophils are abundant innate immune cells that exert context-dependent, bidirectional influences on tumor biology [15]. Although neutrophils can participate in antitumor immunity by enhancing antigen presentation and by recruiting dendritic cells, natural killer cells, and T cells, TANs can be co-opted by the TME to promote malignant progression [16,17,18]. TANs facilitate angiogenesis, remodel the extracellular matrix, suppress the activity of cytotoxic lymphocytes, drive epithelial–mesenchymal transition, and establish pre-metastatic niches [19,20]. These effects are mediated by NETs, web-like DNA–protein structures that can capture circulating tumor cells, proteolytically remodel the extracellular matrix, shield tumor cells from immune surveillance, reactivate dormant cancer cells, and promote therapeutic resistance. Emerging evidence indicates that NETs are associated with clinical prognosis in diffuse large B-cell lymphoma [21]. Nevertheless, their functional contribution to the TME in the context of CAR-γδT-cell therapy remains poorly defined. Elucidating how NETs contribute to the remodeling of the tumor microenvironment during CAR-γδT-cell therapy, and defining the roles they play in this process, is critical for understanding mechanisms of therapeutic response and resistance.

Single-cell RNA sequencing (scRNA-seq) has been increasingly applied in recent years to investigate the mechanisms underlying CAR-T-cell therapies [22]. In this study, we employed scRNA-seq to generate a longitudinal atlas of the peripheral immune landscape in peripheral blood mononuclear cells (PBMCs) collected at four post-infusion time points from five patients treated with allogeneic CAR-γδT cells. Our analysis identified an association between NET-associated inflammatory programs and disease relapse following CAR-γδT-cell therapy, and we further assessed the reproducibility of these transcriptional patterns in an independent publicly available CAR-T-cell dataset.

## 2. Materials and Methods

### 2.1. Study Population

This study enrolled five patients with CD19-positive acute B lymphoblastic leukemia and diffuse large B-cell lymphoma who received CAR-γδT-cell therapy at the First Affiliated Hospital of the University of Science and Technology of China between January 2024 and July 2024. Exclusion criteria included a history of autoimmune diseases or other malignancies; severe cardiac disease; hepatitis B, hepatitis C, or syphilis; active or uncontrolled infection within seven days prior to screening; pregnancy; and treatment with systemic corticosteroids. The study protocol adhered to the principles of the Declaration of Helsinki and was approved by the Ethics Committee of the First Affiliated Hospital of the University of Science and Technology of China (Approval No. 2023KY303). Written informed consent was obtained from all participants prior to their inclusion in this study.

### 2.2. Generation and Expansion of CAR-γδT

CAR-γδT cells were generated from peripheral blood obtained from healthy donors. After isolation of PBMCs (Ficoll-Paque PLUS) by density-gradient centrifugation, Vδ1 T cells were enriched using anti-TCR Vδ1 magnetic bead selection. Purified Vδ1 T cells were activated with anti-CD3/CD28 magnetic beads for 48 h, followed by lentiviral transduction with a second-generation CD19 CAR at an MOI of 10. The CAR construct comprised a GM-CSF receptor-derived signal peptide, a humanized FMC63 anti-CD19 scFv, an IgG4 hinge region, a CD28 transmembrane domain, and intracellular CD28 and CD3ζ signaling domains. Following transduction, cells were expanded for 19 to 21 days in X-VIVO medium supplemented with IL-2, IL-15, and IL-21. To enhance proliferation, irradiated artificial antigen-presenting cells derived from K562 cells and engineered to co-express CD64, CD86, and CD137L were added on days 7 and 14. Large-scale expansion was subsequently performed in a WAVE bioreactor. The final CAR-γδT product was characterized by flow cytometry to confirm CAR expression, Vδ1 T-cell purity, memory phenotype, and minimal residual αβ T-cell contamination. Unless otherwise specified, the reagents and materials used for CAR-γδT cell generation and expansion were provided by PersonGen.Anke Cellular Therapeutics Co., Ltd. (Hefei, Anhui, China).

### 2.3. Single-Cell Suspension Preparation

Peripheral blood samples were collected longitudinally after CAR-γδT cell infusion. Sampling schedules were not identical across patients, and the available collection time points included days 7, 14, 15, 22, and 28, as applicable to individual patients. A detailed patient-by-time-point sampling matrix is provided in Supplementary Figure S1. PBMCs were isolated by density-gradient centrifugation using Ficoll-Paque Plus (GE Healthcare, Cytiva, Uppsala, Swenden), followed by washing with Ca^2+^/Mg^2+^-free PBS (Yuanpei, Shanghai, China). To lyse residual erythrocytes, 2 mL of GEXSCOPE^®^ red blood cell lysis buffer (RCLB, Singleron, Nanjing, China) was added and incubated for 10 min at 25 °C. The suspension was subsequently centrifuged at 500× *g* for 5 min, and the pellet was resuspended in PBS. Samples were then centrifuged at 400× *g* for 5 min at 4 °C, after which the supernatant was discarded. PBMCs were further purified by centrifugation at 400× *g* for 10 min at 4 °C, and the resulting pellet was resuspended in phosphate-buffered saline to obtain a single-cell suspension. Finally, cell viability was assessed microscopically following Trypan Blue staining.

### 2.4. Sequencing Library Preparation and Single-Cell RNA Sequencing

Single-cell suspensions (2 × 105 cells/mL) with PBS (HyClone, Logan, UT, USA) were loaded onto microwell chip using the Singleron Matrix^®^ Single Cell Processing System. Barcoding Beads were subsequently collected from the microwell chip, followed by reverse transcription of the mRNA captured by the Barcoding Beads to obtain cDNA, and PCR amplification. The amplified cDNA was then fragmented and ligated with sequencing adapters. The scRNA-seq libraries were constructed according to the protocol of the GEXSCOPE^®^ Single Cell RNA Library Kits (Singleron, Nanjing, China) [23]. Individual libraries were diluted to 4 nM, pooled, and sequenced on Illumina novaseq 6000 (Illumina, San Diego, CA, USA) with 150 bp paired-end reads.

### 2.5. Primary Analysis of scRNA-seq Raw Read Data

Raw reads from scRNA-seq were processed to generate gene expression matrixes using the CeleScope (https://github.com/singleron-RD/CeleScope, accessed on 1 April 2022) v1.9.0 pipeline. Briefly, raw reads were first processed with CeleScope to remove low-quality reads with Cutadapt v1.17 [24] to trim poly-A tail and adapter sequences. Cell barcode and UMI were extracted. After that, we used STAR v2.6.1a [25] to map reads to the reference genome GRCh38 (ensembl version 92 annotation). UMI counts and gene counts of each cell were acquired with featureCounts v2.0.1 [26] software, and used to generate expression matrix files for subsequent analysis.

### 2.6. Quality Control, Dimensionality Reduction, Clustering, and Cell Type Annotation

The Seurat R package (v5.2.1) was used for quality control, dimensionality reduction, and clustering analysis. For each dataset, the gene expression matrix was filtered according to the following criteria: (1) cells with fewer than 200 detected genes; (2) cells with mitochondrial gene content greater than 30%; and (3) genes expressed in fewer than five cells. Detailed data quality metrics are provided in Supplementary Table S1.

**Figure 1:**
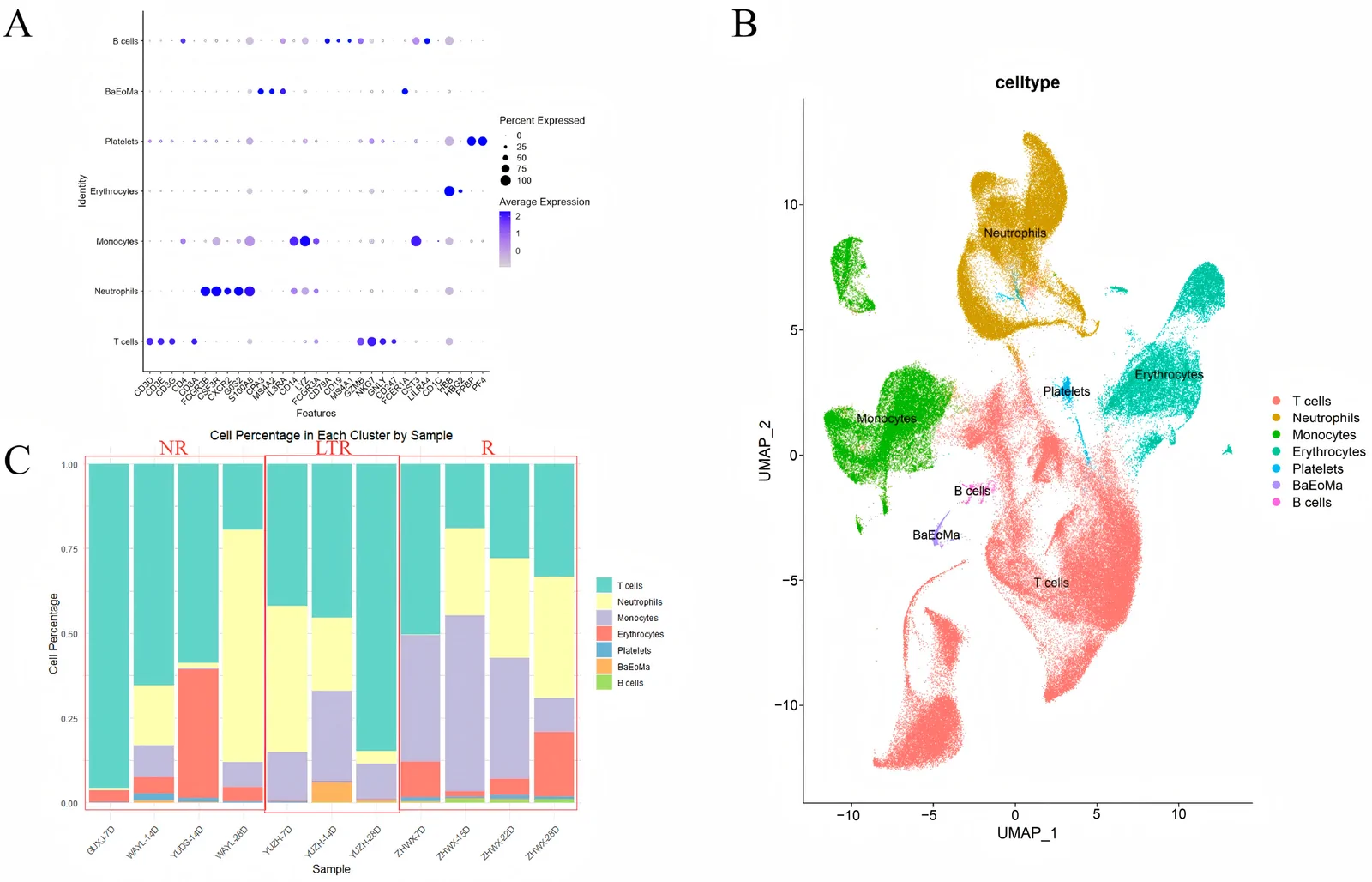
Study overview and scRNA-seq profiling. (**A**) Cell subpopulations were annotated based on canonical cell type marker genes. (**B**) UMAP visualization of 132,810 high-quality PBMCs, identifying seven major immune cell subsets. (**C**) Proportions of each cell subset across individual samples. The red boxes indicate the three clinical groups, from left to right: NR, LTR, and R.

After filtering, a total of 132,810 PBMCs were retained for downstream analyses. Batch effects across samples were corrected using the Harmony algorithm. Gene expression matrices were normalized and scaled using the “NormalizeData” and “ScaleData” functions. The top 3000 highly variable genes were identified using the “FindVariableFeatures” function and subsequently subjected to principal component analysis (PCA). Using the top 20 principal components and a clustering resolution parameter of 0.1, cells were clustered into 15 populations with the “FindClusters” function. Uniform Manifold Approximation and Projection (UMAP) was applied via the “RunUMAP” function for visualization. Cell type identities of each cluster were determined based on canonical marker genes curated from CellMarkerDB, PanglaoDB, and the recently published literature on single-cell transcriptomic profiling. The granulocyte population was identified based on the expression of canonical neutrophil/granulocyte markers, including FCGR3B, CSF3R, CXCR2, G0S2, and S100A8, which showed characteristic enrichment in the annotated neutrophil population (Figure 1).

### 2.7. Single-Cell Enrichment Analysis

GSEA was performed to assess pathway-level transcriptional alterations across conditions. For pseudobulk profiles, gene module enrichment was quantified using Seurat’s “AddModuleScore” function, which computes the averaged expression of predefined gene sets after controlling for background expression. Enrichment scores were then compared across groups to evaluate differential pathway activities at the sample and cell type levels.

### 2.8. Differentially Expressed Gene (DEG) Analysis

To identify DEGs, we applied the Seurat “FindMarkers” function using the Wilcoxon rank-sum test. Differential expression analysis was performed between high-NET-signature transcriptional and low-NET-signature transcriptional neutrophils, restricting the comparison within the neutrophil population. Only genes expressed in at least 5% of cells in either group (min.pct = 0.05) and with a log fold change greater than 0.1 (logfc.threshold = 0.1) were retained. To focus on upregulated genes associated with heightened NET-associated signature score, only positively enriched genes were reported (only.pos = TRUE). *p* values were adjusted using Bonferroni correction, and genes with adjusted *p* < 0.05 were considered statistically significant.

### 2.9. Ligand–Receptor-Based Signaling Inference

To examine whether circulating T-cell transcriptional states were associated with variation in neutrophil-associated NET-related programs, we constructed a transcriptional neighborhood score based on low-dimensional gene-expression similarity. Following dimensionality reduction, Euclidean distances between T cells and neutrophil cells were calculated in the first 30 principal components. For each T cell, the five most transcriptionally similar neutrophil-like cells (K = 5) were identified, and the mean NET-associated transcriptional signature score of these cells was calculated as the NET-associated transcriptional neighborhood score. Here, the term “neighborhood” refers exclusively to transcriptional similarity in the reduced-dimensionality expression space and does not imply anatomical proximity, physical cell–cell contact, or in vivo exposure. The resulting score was used as an exploratory measure of the transcriptional context surrounding individual T cells. To assess the robustness of this approach, sensitivity analyses were performed using alternative neighborhood sizes where applicable.

NicheNet was subsequently used to identify putative ligand–target relationships associated with T-cell transcriptional programs. The neutrophil population was considered a candidate sender population, and T cells were considered receiver cells. These analyses were interpreted as computationally inferred associations and were not considered evidence of direct ligand-mediated signaling or physical interaction in vivo.

### 2.10. Gene Regulatory Network Inference

Gene regulatory network inference and transcription factor activity analysis were performed using the SCENIC workflow. First, co-expression modules between TFs and candidate target genes were inferred using GRNBoost2. Next, motif enrichment analysis was conducted with cisTarget to identify direct TF–target interactions and define regulons. Regulon activity was then quantified at the single-cell level using AUCell, generating an AUC matrix representing regulon activation scores across cells. Finally, regulon specificity scores were calculated to evaluate cell type-specific regulatory programs, and selected regulons were visualized by ranked specificity plots and heatmaps for downstream interpretation.

### 2.11. Statistical Analysis

All statistical analyses were performed using R (https://www.r-project.org/, accessed on 1 April 2022). Continuous variables are summarized as the mean ± standard deviation or median with interquartile range, and categorical variables are presented as counts and percentages. Comparisons between two groups were conducted using the Wilcoxon rank-sum test (for non-normally distributed data) or Student’s *t*-test (for normally distributed data). For multiple group comparisons, Kruskal–Wallis test or one-way ANOVA with appropriate post hoc correction was applied. Correlation analyses were conducted using Spearman’s rank correlation coefficient. For single-cell analyses, pseudobulk aggregation was applied when required, and module/activity scores were compared using non-parametric tests. Multiple hypothesis testing was adjusted using the Benjamini–Hochberg FDR or Bonferroni correction where applicable. A two-sided *p* value < 0.05 was considered statistically significant.

## 3. Results

### 3.1. Single-Cell Atlas of Peripheral Blood from Patients Receiving CAR-γδT-Cell Therapy

Five patients with CD19-positive B-cell malignancies underwent lymphodepleting chemotherapy followed by infusion of allogeneic CAR-γδT cells. To construct a single-cell transcriptional atlas of the peripheral immune landscape during CAR-γδT-cell therapy, a total of 11 peripheral blood samples were collected across multiple time points. After scRNA-seq processing and stringent quality control, 132,810 high-quality PBMCs were retained for downstream analyses. Among the enrolled patients, YUZH achieved a partial response (PR) that was sustained throughout follow-up, whereas ZHWX achieved a complete response (CR) at day 21 but subsequently experienced relapse two months post infusion. Detailed patient characteristics are summarized in Table 1.

We analyzed 11 longitudinal PBMC samples collected from five independent patients receiving allogeneic CAR-γδT-cell therapy. The patients comprised three non-responders (NR; four samples), one long-term responder (LTR; three samples), and one patient who achieved an initial response followed by relapse (R; four samples). Thus, the LTR and R categories each represent a single independent patient, and longitudinal observations within a patient should not be considered independent biological replicates. Accordingly, comparisons across clinical categories were interpreted descriptively and as hypothesis-generating. After quality control, single-cell transcriptomic profiles were integrated using Harmony to remove batch effects, followed by UMAP dimensionality reduction and clustering. Based on canonical marker genes, we annotated seven major immune and hematopoietic cell lineages (Figure 1A,B), namely, B cells, T cells, monocytes, neutrophils, erythrocytes, platelets, and basophil/eosinophil/mast cell populations (BaEoMa). The overall clustering patterns were highly consistent across samples from different patient cohorts, confirming successful integration.

Descriptive examination of cell type composition across the longitudinal samples revealed heterogeneous patterns among individual patients and clinical outcome categories (Figure 1C). Given the limited number of independent patients and unequal longitudinal sampling, these patterns were interpreted primarily at the individual-patient level rather than as definitive differences between clinical groups. In the NR group, T cells constituted a disproportionately high fraction at early time points but progressively declined over time, whereas the LTR group exhibited an opposite trend, with T-cell proportions gradually increasing. Monocytes were more abundant in the R group than in both the LTR and NR groups, suggesting a potential association between monocyte enrichment and disease relapse.

### 3.2. T-Cell Exhaustion Distinguishes Longitudinal Clinical Response Patterns

**Figure 2:**
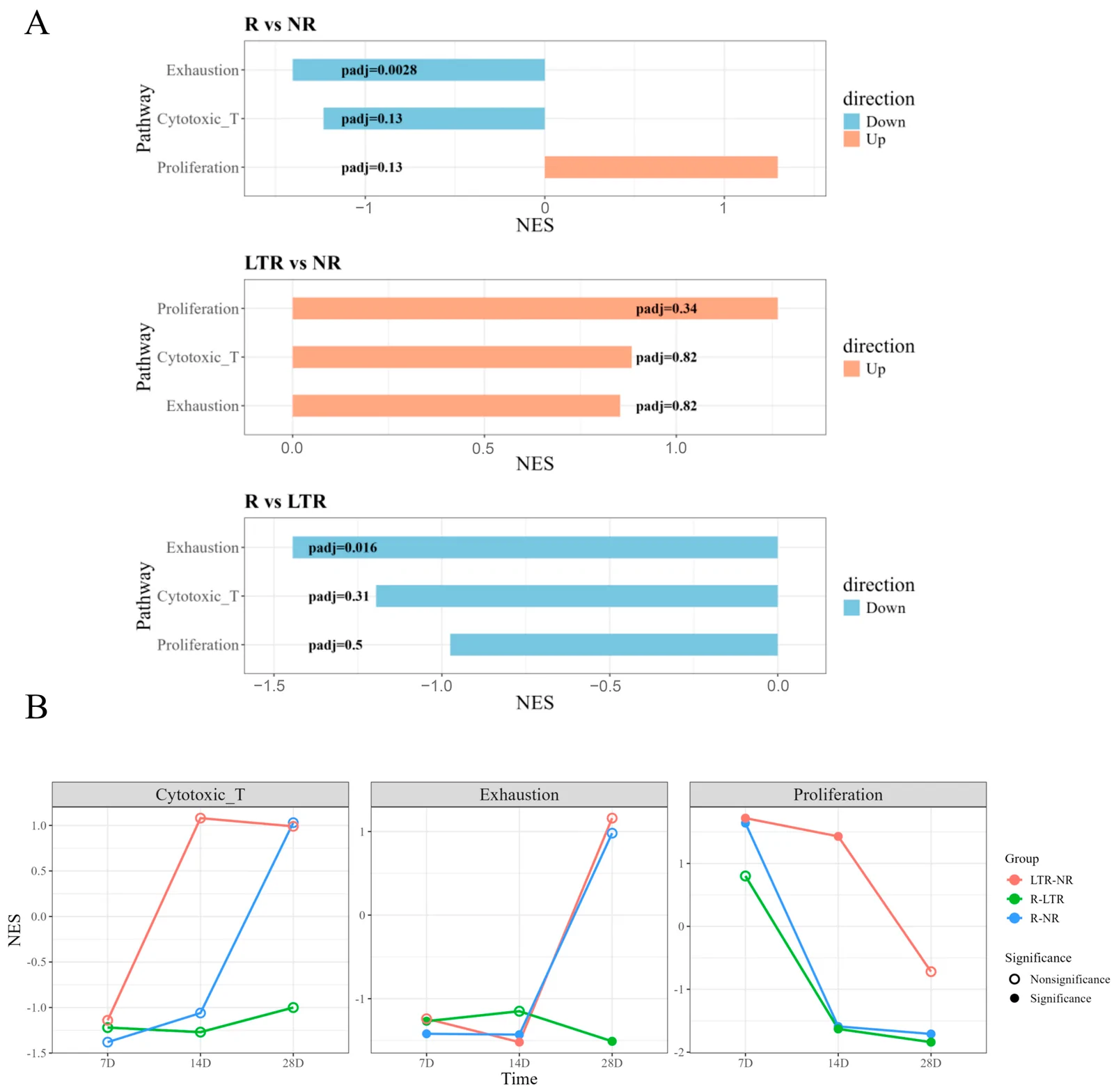
Functional state enrichment of T cells during CAR-γδT-cell therapy. (**A**) Ranked GSEA was performed to compare T-cell transcriptional programs among the three clinical outcome groups (R, NR, and LTR). Pairwise comparisons (R vs. NR, LTR vs. NR, and R vs. LTR) revealed differences in the enrichment of proliferation-, cytotoxicity-, and exhaustion-related signatures. (**B**) Functional scores of proliferation, cytotoxicity, and exhaustion programs were further evaluated in the R, NR, and LTR groups at approximately day 7, day 14, and day 28, respectively, to characterize their longitudinal dynamics. The labels day 7, day 14, and day 28 indicate approximate post-treatment time windows, as actual sample collection days varied across patients according to clinical sampling schedules. The actual collection days are shown in Supplementary Figure S1.

To characterize functional differences in T cells across clinical outcomes, we quantified enrichment scores for three curated gene modules—cytotoxicity, exhaustion, and proliferation—using gene sets derived from previously published studies [27,28]. For statistical comparisons, T-cell transcriptomes were aggregated at the individual-sample level to generate sample-specific pseudobulk profiles, with each of the 11 longitudinal samples treated as one observational unit. Ranked gene set enrichment analysis (GSEA) was then performed using sample-level differential-expression statistics for the comparisons R versus NR, LTR versus NR, and R versus LTR (Figure 2A). Across all contrasts, neither the cytotoxicity nor the proliferation gene sets exhibited significant enrichment (non-significant *p* values and false discovery rate (FDR)), indicating that the overall cytolytic potential and recent proliferative activity of T cells were largely comparable between groups. In contrast, the exhaustion-associated transcriptional program showed lower enrichment in the R patient than in the LTR and NR samples (NES < 0; FDR < 0.1), revealing a distinctly lower exhaustion state in the R patient. Given that the R patient initially achieved CR, whereas the LTR patient maintained only PR, this lower exhaustion-associated transcriptional activity coincided with the initial achievement of CR in the R patient, but its functional significance remains uncertain.

To examine longitudinal changes in circulating T-cell functional states, we evaluated the available serial samples from each patient. Because sampling schedules differed between patients, longitudinal patterns were interpreted primarily within individuals rather than as balanced group trajectories. Cytotoxicity scores showed no significant differences at any time point (Figure 2B). In contrast, exhaustion scores showed early divergence: at days 7 and 14, the NR samples showed higher exhaustion-associated scores than the R and LTR samples at the corresponding sampling windows, with the R cohort maintaining persistently low exhaustion during this early phase. By day 28, the differences among groups became less pronounced overall, although a clear separation between the R and LTR cohorts remained. These findings suggest that the NR cohort enters an early state of high exhaustion and low proliferation, potentially due to chronic stimulation or an inhibitory microenvironment driving T cells toward exhaustion at the onset of therapy, thereby preventing effective and sustained expansion or cytotoxic activity, and this may be consistent with an early dysfunctional or highly stimulated T-cell state. In the R cohort, the decline in proliferation at day 14 may reflect the achievement of complete remission and the accompanying reduction in tumor burden; however, the continued decrease in proliferative activity thereafter may contribute to subsequent relapse. In contrast, the LTR patient showed relatively high exhaustion-associated transcriptional activity together with sustained proliferation. This pattern may reflect heterogeneous T-cell states rather than terminal dysfunction. Although some exhaustion-associated cells may retain progenitor- or memory-associated features, our subclustering analysis did not identify a sufficiently discrete population to confidently define progenitor-exhausted T cells [29,30]. Therefore, this interpretation remains hypothetical and requires further phenotypic, functional, and multimodal validation. In addition, host- or microenvironment-derived factors may help maintain long-term disease control, which could explain why these patients did not relapse despite elevated exhaustion scores.

### 3.3. NET-Associated Transcriptional Signatures Are Associated with T-Cell Functional States and Clinical Relapse

#### 3.3.1. Longitudinal NET Dynamics Differ Across Clinical Outcome Groups

Neutrophils play pivotal and context-dependent roles in tumor development, exerting both antitumor and pro-tumor functions [31]. In the innate immune response, neutrophils contribute critically to nonspecific immunity by promoting the recruitment, activation, and maturation of monocytes, macrophages, DCs, and NK cells. In adaptive immunity, they facilitate antigen-specific responses by supporting the differentiation of monocytes and DCs into professional antigen-presenting cells (APCs), thereby enhancing T- and B-cell activation [32]. When considering the role of neutrophils in cancer progression or therapeutic response, the formation of NETs has emerged as a key biological process [33].

**Figure 3:**
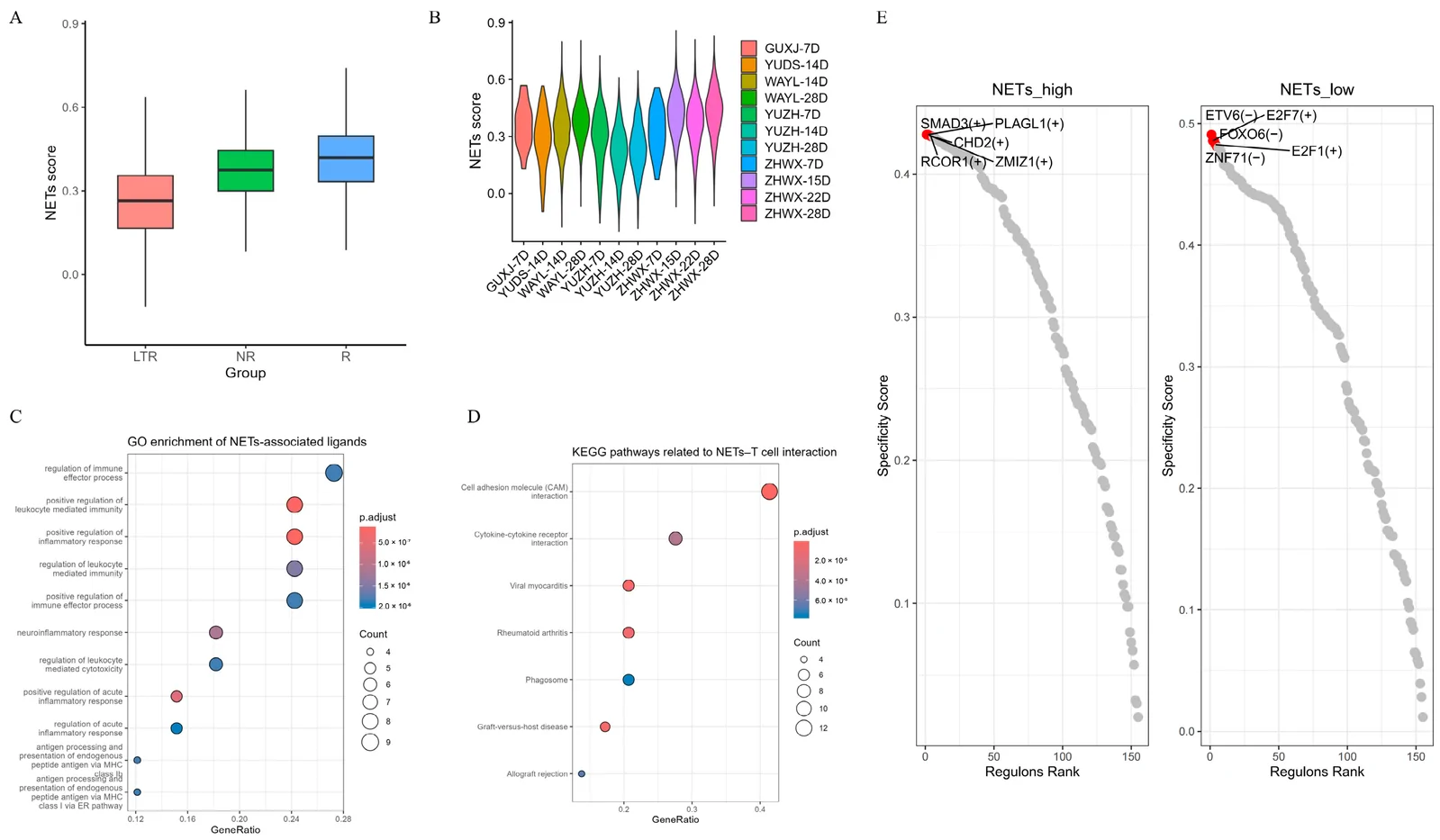
NET-associated transcriptional signatures and circulating T-cell functional programs during CAR-γδT-cell therapy. (**A**) NET module score across the three clinical groups (R, NR, and LTR). (**B**) NET module score at the individual sample level. (**C**) GO enrichment analysis of differentially expressed ligand–receptor pair-associated genes between high-NET-signature transcriptional and low-NET-signature transcriptional T-cell groups. (**D**) KEGG pathway enrichment analysis of differentially expressed ligand–receptor pair-associated genes between high-NET-signature transcriptional and low-NET-signature transcriptional T-cell groups. (**E**) Top five transcription factors identified by SCENIC analysis in the high-NET-signature transcriptional or low-NET-signature transcriptional T-cell group.

In our single-cell sequencing workflow, we applied a microwell-based chip platform in which cells naturally settle into microwells without mechanical stress, allowing for efficient capture of neutrophils. We curated a set of 69 NET-associated genes based on the published literature and computed NET signature scores to quantify NET-associated signatures across samples and patient cohorts [34]. Because this score is derived from transcriptomic data, it should be interpreted as a transcriptional surrogate associated with NET-related biology rather than as a direct measurement of extracellular NET formation. Notably, although patients in the R group initially demonstrated favorable therapeutic responses at the time of sampling, their NET scores continued to rise longitudinally, suggesting that increasing NET-associated transcriptional signature was temporally associated with subsequent relapse in this patient and raising the hypothesis that NET-associated inflammatory states may be relevant to disease control (Figure 3A). In contrast, LTR maintained consistently low NET scores which further declined over time (Figure 3B).

#### 3.3.2. Exploratory Associations Between NET-Associated Transcriptional Signatures and T-Cell Functional Programs

To further investigate the relationship between NET-associated transcriptional signatures and T-cell functional states, including exhaustion, proliferation, activation, and cytotoxicity, we calculated the corresponding gene set scores for each cell and then summarized the scores at the sample level. For each of the 11 PBMC samples, the NET-associated score was summarized across neutrophils, while T-cell functional scores were summarized across T cells, yielding one paired observation per sample. Associations between NET-associated scores and T-cell functional states were assessed using two-sided Spearman rank correlation across the 11 samples. Because multiple longitudinal samples were obtained from the same five patients, these sample-level correlations were considered exploratory and do not represent five independent patient-level observations. We observed a significant negative correlation between NET scores and T-cell proliferation, whereas a positive correlation was detected between NET scores and cytotoxicity. No significant associations were observed for the remaining states.

To further assess the robustness of these findings, multiple complementary statistical approaches were employed (Table 2). The negative association between NET scores and T-cell proliferation was supported by permutation testing (*p*_emp_ = 0.019), bootstrap confidence intervals (CI = −0.935 to −0.146), and leave-one-out (LOO) analysis (−0.762 to −0.464), while the mixed-effects model showed a borderline association (β = −0.698; *p* = 0.055). These findings suggest an inverse association between NET-associated transcriptional activity and the T-cell proliferation-related transcriptional program, although the association was not uniformly significant across all statistical approaches. For cytotoxicity, permutation testing (*p*_emp_ = 0.039), LOO analysis (0.214–0.786), and mixed-effects modeling (β = 1.28; *p* = 0.049) yielded evidence of a positive association with NET levels; however, the bootstrap confidence interval (CI = −0.042 to 0.858) crossed zero, indicating uncertainty regarding the robustness of this association. T-cell activation showed a significant positive association only in the mixed-effects model (β = 0.769; *p* = 0.015), whereas other metrics indicated unstable or non-significant correlations.

Overall, these results suggest that elevated NET-associated transcriptional activity is associated with reduced proliferative transcriptional activity in circulating T cells, while remaining positively associated with cytotoxic and activation-related states. This pattern raises the possibility that NET-associated inflammatory states may coexist with effector-associated T-cell programs while being accompanied by reduced proliferative activity; however, this interpretation remains hypothesis-generating and requires further validation.

#### 3.3.3. NET-Associated Ligand–Receptor Interactions Suggest Chronic Inflammatory Remodeling of T-Cell States

To investigate the transcriptional association between neutrophil NET-associated transcriptional signatures and T-cell functional states, we first computed a NET-associated gene set score (NET score) at the single-cell level and classified neutrophils into high-NET-signature transcriptional and low-NET-signature transcriptional populations. To further characterize the transcriptional association between T cells and high-NET-signature transcriptional neutrophil states, we applied a transcriptional neighborhood-based scoring approach derived from PCA embeddings. Specifically, for each T cell, its five nearest neighboring neutrophils (K = 5) were identified using the Euclidean distance calculated across the first 30 principal components. The mean NET-associated signature score of these neighboring neutrophils was then computed and defined as the “neighboring NET score.”

Based on the median value of this neighboring NET score, T cells were stratified into high-NET-signature transcriptional and low-NET-signature transcriptional groups, reflecting their relative transcriptional association with high- or low-NET-signature neutrophil states Differential expression analysis was subsequently performed between the two T-cell groups, and the results were integrated with NicheNet analysis to infer potential ligand-associated genes. This approach identified a set of putative candidate ligands, including PLEKHO2, COL18A1, SERPINA1, HLA-C, ALOX5AP, HLA-F, CMTM2, LRRC4, IL16, OSM, GRN, FBRSL1, PTGS2, HLA-B, ICAM3, ADM, HLA-E, NAMPT, IL1RN, MMP9, ITGB2, TNFSF13B, IL1B, CD58, TGFB1, TNFSF8, SELL, SELPLG, PROK2, CXCL1, ITGAM, ITGAL, UTS2, and HMGB2.

Among the identified ligands, HMGB1 and HMGB2 can be released by neutrophils during NETosis and incorporated into NETs, thereby amplifying inflammatory responses [35]. Within NETs, HMGB1 cooperates with DNA, histones, and other components to form a web-like scaffold and modulates immune cell function through interactions with pattern-recognition receptors such as RAGE and TLR4. Extracellular HMGB1 activates downstream NF-κB and MAPK signaling pathways, promoting pro-inflammatory cytokine production and enhancing TCR-mediated T-cell responses [36]. However, in the context of chronic inflammation or the tumor microenvironment, sustained HMGB1 signaling and persistent immune stimulation are associated with the recruitment and differentiation of immunosuppressive cell populations, including myeloid-derived suppressor cells and regulatory T cells, as well as with the upregulation of immune checkpoint pathways such as PD-1 and TIM-3. Together, these mechanisms may be consistent with effector T-cell exhaustion and functional impairment, ultimately limiting antitumor immunity.

Emerging evidence indicates that NETs can induce the production of IL-1β within the peripheral immune microenvironment. In turn, IL-1β activates inflammatory cascades through NF-κB and MAPK signaling pathways, leading to the induction of downstream pro-inflammatory mediators such as IL-6, TNF-α, and CXCL8. Sustained activation of these pathways promotes a chronic inflammatory microenvironment that supports tumor cell proliferation and survival, while profoundly reshaping the peripheral immune microenvironment [37].

Neutrophil-associated factors, including MMP9 and PTGS2/COX-2, act as critical mediators linking inflammation to tumor progression. Neutrophil-derived MMP9 facilitates extracellular matrix degradation and angiogenesis, thereby enhancing tumor invasion and growth [38]. In parallel, COX-2-derived prostaglandin E2 suppresses effector T-cell function and promotes the expansion of regulatory T cells and myeloid-derived suppressor cells, contributing to an immunosuppressive immune microenvironment [39]. In addition, inflammatory mediators such as NAMPT, TGFB1 and CXCL1 further participate in immune suppression and tumor progression. NAMPT functions as a pro-inflammatory cytokine that promotes immune evasion by regulating suppressive myeloid cell populations and impairing T-cell-mediated antitumor immunity. TGFB1 is a potent immunosuppressive factor that directly inhibits T-cell proliferation and effector function while promoting regulatory T-cell differentiation. Meanwhile, CXCL1 recruits neutrophils and other myeloid cells into the peripheral immune microenvironment, reinforcing inflammatory and immunosuppressive circuits.

Cell adhesion and immune synapse-related molecules, including SELPLG, SELL, ITGAM, ITGAL, ICAM3 and CD58, play essential roles in regulating immune cell trafficking, cell–cell interactions, and T-cell function within the tumor microenvironment. SELPLG acts as an immune checkpoint molecule that promotes T-cell exhaustion, whereas ITGAM expressed on myeloid cells mediates suppression of T-cell activation and proliferation [40]. Altered ITGAL–ICAM interactions, together with reduced CD58-mediated costimulatory signaling, further impair immune synapse formation and effective antitumor T-cell responses, ultimately facilitating immune evasion in cancer [41].

Next, functional enrichment analysis was performed on these candidate ligands. Gene Ontology (GO) analysis revealed significant enrichment of multiple inflammation-related regulatory pathways, suggesting the presence of a sustained amplification of innate immune inflammatory signaling within high-NET-signature transcriptional neighborhoods (Figure 3C). Previous studies have shown that NETs are frequently associated with chronic inflammation, tissue damage, and the accumulation of inflammatory mediators, and can act as amplifiers of inflammatory responses, thereby shaping a nonspecific immune activation background [42,43]. In such environments, persistent inflammatory stimulation is thought to interfere with the finely tuned, TCR-dependent activation process, rendering T cells more prone to impaired proliferative capacity and functional dysfunction [44]. Concurrently, the enrichment of antigen processing and presentation pathways indicates a remodeling of antigen presentation programs, involving not only classical MHC class I molecules but also non-classical MHC class Ib molecules, such as HLA-E and HLA-F. The expression of non-classical MHC molecules has been reported to be associated with chronic inflammatory states and tumor immune evasion, suggesting that NET-associated inflammatory microenvironments may alter the antigen presentation landscape and thereby affect the quality, rather than merely the magnitude, of T-cell activation [45]. In addition, the enrichment of leukocyte-mediated immune effector and regulatory pathways, together with the significant enrichment of the cell adhesion molecule (CAM) pathway in Kyoto Encyclopedia of Genes and Genomes (KEGG) analysis, suggests that T-cell migration, cell–cell interactions, and immune synapse formation may be modulated in a high-NET-signature transcriptional context (Figure 3D). Previous studies have demonstrated that NETs can reshape and restrict effective immune cell trafficking and intercellular interactions through their physical barrier properties and adhesive characteristics [44]. Notably, enrichment of the cytokine–cytokine receptor interaction pathway highlights a cytokine network characterized by the coexistence of inflammatory activating factors and immunosuppressive mediators. This immune state of “persistent stimulation but inefficient effector responses” has been widely recognized as a critical background for T-cell dysfunction and exhaustion under chronic inflammatory conditions [44].

Taken together, the GO and KEGG enrichment analyses provide convergent evidence for a coherent mechanistic framework in which, within high-NET-signature transcriptional contexts, amplified innate inflammation, remodeling of antigen presentation modalities, and alterations in adhesion and immune synapse-related processes act in concert to place T cells in a state of continuous stimulation but impaired capacity to generate high-quality effector responses, provides a possible explanation reduced T-cell proliferation and functional abnormalities.

In this study, SCENIC analysis revealed alterations in transcription factor regulatory networks within T cells in high-NET-signature transcriptional versus low-NET-signature transcriptional microenvironments (Figure 3E). Based on the aforementioned NET-associated ligand–receptor interactions and pathway enrichment results, we further investigated whether these NET-associated extrinsic signals are transduced into specific transcriptional programs within T cells. To this end, T cells were stratified into high-NET-signature transcriptional and low-NET-signature transcriptional states, and regulon activity was calculated for each cell using single-cell transcriptome data. In the high-NET-signature transcriptional group, higher regulon activity such as SMAD3, PLAGL1, CHD2, RCOR1, and ZMIZ1 indicated that these T cells exhibited higher activity of transcriptional regulatory programs associated with immunoregulatory states. SMAD3, a key transcriptional mediator of TGF-β signaling, is known to be involved in TGF-β-mediated regulation of T-cell differentiation and supports activation of TGF-β pathways, which is consistent with the enrichment of TGFB1 and inflammation-related ligands in high-NET-signature transcriptional microenvironments [46,47]. Upregulation of SMAD3 has been associated with suppressed T-cell proliferation and enhanced regulatory T-cell differentiation [48]. PLAGL1, CHD2, RCOR1, and ZMIZ1 upregulation reflects transcriptional programs associated with cell cycle arrest, chromatin repression, and functional impairment, suggesting that these transcriptional differences may be associated with functional limitations or exhaustion-like states in T cells under NET-associated inflammatory conditions. However, this interpretation remains hypothesis-generating and was not directly functionally validated in the present study. This transcriptional signature is consistent with GO and KEGG enrichment results showing immune effector regulation, chronic inflammatory responses, and CAM pathways. In contrast, the transcription factor expression pattern in the NET-low group showed features of higher proliferative and effector function. Specifically, upregulation of E2F1 and E2F7 is known to promote cell cycle progression and drive T-cell proliferation and effector differentiation [49,50]. Meanwhile, lower regulon activity of FOXO6 and ETV6 may relieve transcriptional repression and potentially promote T-cell activation and metabolic activity; FOXO family transcription factors are established regulators of T-cell differentiation and function [51,52]. Taken together, SCENIC analysis suggests that differences in NET-associated transcriptional contexts are associated with distinct intracellular transcriptional regulatory programs in T cells, highlighting candidate regulatory mechanisms for future investigation.

### 3.4. External Supportive Analysis in an Independent CAR-T Cohort

**Figure 4:**
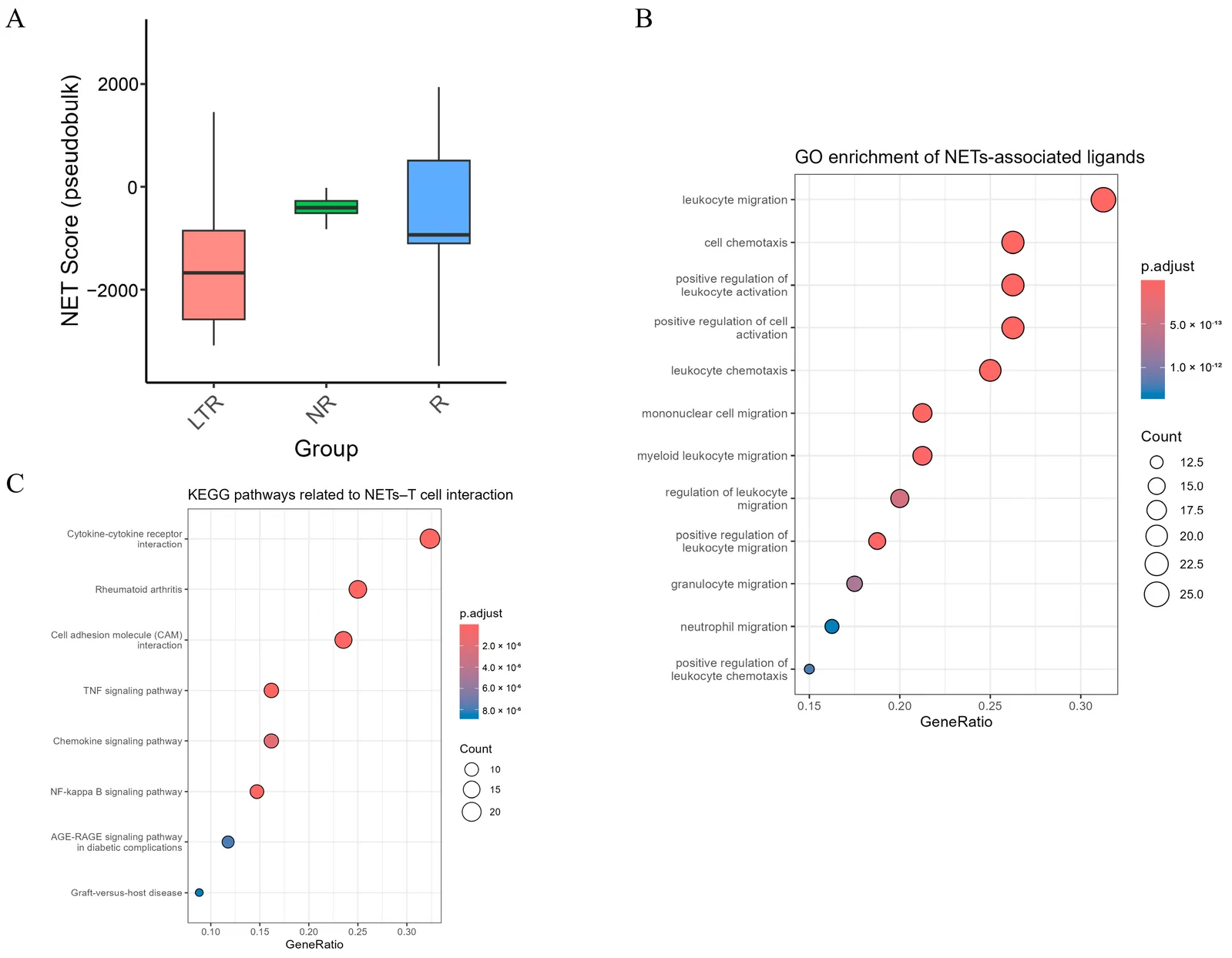
NET-associated transcriptional programs and their impact on T-cell states and regulatory programs in the external cohort. (**A**) NET module score across the three clinical groups (R, NR, and LTR) in the external cohort. (**B**) GO enrichment analysis of differentially expressed ligand–receptor pair-associated genes between high-NET-signature transcriptional and low-NET-signature transcriptional T-cell groups in the external cohort. (**C**) KEGG pathway enrichment analysis of differentially expressed ligand–receptor pair-associated genes between high-NET-signature transcriptional and low-NET-signature transcriptional T-cell groups in the external cohort.

Given the limited number of clinical samples in our study, we performed an external supportive analysis using the publicly available dataset GSE290722, which contains longitudinal PBMC single-cell transcriptomic data from patients with relapsed/refractory large B-cell lymphoma treated with axicabtagene ciloleucel (axi-cel), an autologous CD19-directed CAR-T cell product. In this cohort, 13 patients achieved LTR, 8 were NR, and 7 experienced R. To enable patient-level comparison, we performed pseudobulk analysis and calculated NET-associated scores using the same NET gene signature applied in our primary dataset. Consistently, NET scores were markedly lower in the LTR group compared with the R group, indicating a reduced NET-associated inflammatory burden in patients with durable clinical benefit (Figure 4A). These observations provide supportive evidence that NET-associated inflammatory transcriptional programs are associated with clinical response across related CAR-T treatment settings.

We next analyzed T cells to examine whether NET-associated transcriptional patterns were linked to T-cell functional states in the external cohort. Because neutrophils were not captured in GSE290722, we did not interpret the NET score as a direct measure of neutrophil-derived NETs. Instead, we used a K-nearest-neighbor approach to infer a NET-associated transcriptional neighborhood for T cells based on the NET scores of surrounding non-neutrophil cells. T cells were subsequently stratified according to the inferred NET-associated neighborhood score. Within this external cohort, a total of 81 candidate ligands were identified, among which 15 overlapped with our primary cohort, namely, SERPINA1, IL1B, GRN, NAMPT, TNFSF13B, ADM, IL1RN, ITGAM, PLEKHO2, SELL, PTGS2, OSM, ITGB2, CD58, and ALOX5AP. Notably, several key inflammatory mediators consistently observed across both datasets—such as IL1B, PTGS2/COX-2, and NAMPT, which were discussed previously. These overlapping signals are compatible with a broader inflammatory and immune-regulatory transcriptional state associated with the NET gene signature. The recurrence of adhesion- and immune-interaction-related ligands, including ITGAM, ITGB2, SELL, and CD58, is consistent with alterations in cell–cell communication associated with the inferred transcriptional neighborhood. In addition, regulatory mediators such as TNFSF13B, GRN, SERPINA1, and ADM further support the concept of immune microenvironment remodeling rather than merely acute immune activation. Taken together, the overlap in ligand-associated signals provides supportive cross-cohort evidence for shared inflammatory, adhesion-related, and immunoregulatory transcriptional features associated with the NET gene signature.

Notably, GO and KEGG enrichment analyses based on the external cohort revealed a highly coherent pattern of immune and inflammatory alterations (Figure 4B,C). At the GO level, the identified ligands were enriched in leukocyte migration, leukocyte chemotaxis, and myeloid- and neutrophil-related trafficking terms, suggesting that the transcriptional program captured by these ligands is associated with immune cell recruitment and inflammatory communication. Consistently, KEGG analysis further highlighted chemokine and cytokine signaling pathways, suggesting the presence of a complex cytokine network that continuously sustains inflammatory activation. Meanwhile, the significant enrichment of the CAM pathway implies that intercellular interaction, immune synapse stability, and immune cell trafficking may be structurally remodeled in NET-associated transcriptional contexts. In addition, classical inflammatory cascades, including the TNF signaling pathway, NF-κB signaling pathway, and pathways related to immune-mediated tissue injury, were significantly enriched, collectively indicating a chronic inflammatory and immunopathological state. These enrichment patterns are compatible with an immune and inflammatory transcriptional state involving chemokine signaling, cytokine regulation, adhesion, and immune cell communication. Together with the primary-cohort findings, they provide supportive evidence that the transcriptional program captured by the NET signature is associated with broader immune-state remodeling.

We next examined the relationship between the inferred NET-associated transcriptional neighborhood score and T-cell functional states, including proliferation, activation, cytotoxicity, and exhaustion. We observed that, at the pseudobulk (sample) level, NET transcriptional neighborhood was strongly positively associated with T-cell activation and cytotoxicity scores, and showed a moderate but highly significant association with T-cell exhaustion (Table 3). For proliferation, the sample-level Spearman analysis showed a modest positive association, whereas the mixed-effects model showed a negative coefficient (β = −0.033; *p* < 0.001). Thus, the two statistical approaches did not yield concordant estimates for proliferation, and this association should not be considered replicated or robust. The discrepancy likely reflects, at least in part, the different units and assumptions of the two analyses: the Spearman analysis evaluates the marginal association between sample-level summaries, whereas the mixed-effects model evaluates the conditional association using individual T-cell observations while accounting for clustering within samples through a sample-level random intercept. Given the limited number of biological samples and the different CAR-T treatment setting, these results are best interpreted as exploratory and hypothesis-generating rather than as evidence of a reproducible NET–T-cell proliferation relationship.

In the primary cohort, the T-cell-associated NET transcriptional neighborhood was derived from the transcriptional features of neighboring neutrophil populations and therefore more directly reflected the relationship between neutrophil-associated NET signatures and T-cell states. In contrast, because neutrophils were absent from the external cohort, the corresponding T-cell score was inferred from NET signature scores of surrounding non-neutrophil cells using a transcriptional neighborhood approximation based on K-nearest neighbors. Thus, the external-cohort metric is better interpreted as a NET-associated inflammatory neighborhood rather than as evidence of direct physical proximity to or interaction with neutrophils.

Accordingly, the external cohort does not provide direct confirmation of the NET–T-cell functional associations identified in the primary cohort. Rather, it provides supportive evidence that the NET-associated transcriptional program is linked to broader inflammatory and T-cell functional states in a related but distinct CAR-T treatment setting. These observations should therefore be interpreted as supportive associations rather than as reproducible or mechanistically validated NET-specific effects.

## 4. Discussion

In this study, we performed a longitudinal single-cell transcriptomic analysis of the peripheral immune landscape in patients receiving allogeneic CAR–γδT-cell therapy and identified NET-associated transcriptional activity as a feature associated with longitudinal clinical outcome and circulating T-cell states. Our data demonstrate that, although some patients initially achieved clinical responses, NET-associated transcriptional signatures increased upon relapse, suggesting that NET-associated inflammatory remodeling of the peripheral immune microenvironment may contribute to treatment failure.

By integrating ligand–receptor inference, pathway enrichment, and transcriptional regulatory network analyses, we further delineated a coherent mechanistic framework. A high-NET-signature transcriptional peripheral immune microenvironment establishes a chronic inflammatory milieu characterized by extensive release of pro-inflammatory and immunoregulatory mediators: IL1B/IL1RN sustaining persistent inflammatory signaling and promoting leukocyte activation and adhesion; OSM, GRN, ADM, SERPINA1, and TNFSF13B contributing to tissue remodeling, immune regulation, and maintenance of chronic inflammation; and adhesion molecules and CAM pathways (ITGAM, ITGB2, CD58, SELL, and PECAM1) reinforcing prolonged immune–immune interactions. Concurrently, chemokine–cytokine cascades together with NF-κB/TNF axis signaling lock the immune environment into an “inflammation-permissive state.” These observations suggest a candidate transcriptional framework involving SMAD3-associated regulatory activity and E2F-related proliferative programs.

This study has limitations, including the relatively limited clinical sample size and reliance on transcriptional inference rather than direct functional perturbation. To provide external supportive evidence, we analyzed a publicly available CAR-T-cell dataset with an independent cohort and treatment setting. Because neutrophils were not captured in the external dataset, we inferred a NET-associated transcriptional neighborhood for T cells based on the NET signature scores of neighboring non-neutrophil cells using a K-nearest-neighbor approach. Similarly, T cells in high-NET-signature transcriptional exposure conditions exhibited enhanced activation and cytotoxic features but simultaneously displayed elevated exhaustion signatures; a similar pattern of concurrent activation- and exhaustion-associated transcriptional features was observed across the two cohorts. Moreover, sample-level associations analyses demonstrated a similar association between NET-related inflammatory signaling and T-cell functional decline, supporting an association between NET-associated inflammatory programs, circulating immune states, and clinical outcomes during CAR-T-cell therapy. Although the therapeutic product was generated from donor-derived Vδ1-enriched γδT cells, the post-infusion PBMC scRNA-seq data did not allow for unambiguous assignment of individual sequenced T cells to the infused CAR-γδT cell product. In the absence of definitive CAR transgene, donor-specific, or lineage-tracing information at the single-cell level, the T-cell populations characterized in this study should therefore be interpreted as circulating T-cell states observed during CAR-γδT-cell therapy and may include endogenous recipient T cells. Accordingly, the observed transcriptional states cannot be attributed exclusively to the persistence or functional fate of the infused CAR-γδT-cell product.

In addition, detailed longitudinal clinical and treatment-related variables that could influence inflammatory and neutrophil-associated transcriptional states, including infection, corticosteroid exposure, lymphodepletion, cytokine-release syndrome, tumor burden, disease progression, and concomitant medications, were not available in a sufficiently standardized form for systematic adjustment because of data sharing and institutional restrictions within the collaborative clinical project. Therefore, the longitudinal increase in NET-associated transcriptional activity, particularly in the single patient who subsequently relapsed, cannot be attributed specifically to impending relapse.

Future work in larger, multicenter cohorts is required to establish the causal relationship between NETs and therapeutic outcomes, as well as to experimentally validate the intervenable potential of key nodes, such as the IL-1 axis, adhesion molecule pathways, or SMAD3-mediated regulation. Notably, the increase in the NET-associated transcriptional signature observed before relapse in the single relapsing patient warrants further investigation as a potential longitudinal biomarker. Future studies combining NET-targeting approaches with CAR-T therapy will be needed to determine whether modulation of NET-associated inflammatory pathways can improve the durability and efficacy of CAR-T-cell treatment.

## Figures and Tables

**Table 1:** Baseline demographics, disease characteristics, and clinical outcomes of enrolled patients.

Patient ID	Gender	Disease	Response
GUXJ	F	DLBCL	NR
YUDS	M	B-ALL	NR
WAYL	F	DLBCL	NR
YUZH	M	DLBCL	LTR
ZHWX	F	B-ALL	R

**Table 2:** Correlation of NET signature scores with T-cell exhaustion, activation, proliferation, and cytotoxicity scores.

Gene Set	rho	*p*	Perm *p*	Bootstrap 95% CI	LOO ρ	Mixed Model β	Mixed Model *p*
Exhaustion	−0.160	0.63	0.637	[−0.654, 0.570]	[−0.500, 0.310]	−0.001	0.995
Proliferation	−0.700	0.016	0.019	[−0.935, −0.146]	[−0.464, −0.762]	−0.698	0.055
Activation	0.400	0.22	0.22	[−0.296, 0.908]	[0.214, 0.833]	0.769	0.015
Cytotoxicity	0.636	0.035	0.039	[−0.042, 0.858]	[0.214, 0.786]	1.28	0.049

Note: Correlation analyses were performed across n = 11 PBMC samples, with one observation per sample. Two-sided Spearman rank correlations were used for the primary association analysis. Permutation *p* values were obtained by sample-level label permutation, bootstrap confidence intervals by sample-level resampling, and LOO estimates by sequential exclusion of individual samples. Mixed-effects models included patient identity as a random intercept to account for repeated longitudinal samples. All analyses were exploratory given the five-patient cohort.

**Table 3:** Correlation between NET signature scores and T-cell exhaustion, activation, proliferation, and cytotoxicity scores in external cohort.

Gene Set	rho	*p*	Perm *p*	Bootstrap 95% CI	LOO ρ	Mixed Model β	Mixed Model *p*
Exhaustion	0.472	<0.001	<0.001	[0.227, 0.634]	[0.441, 0.503]	0.113	<0.001
Proliferation	0.275	0.046	0.043	[0.007, 0.508]	[0.233, 0.313]	−0.033	<0.001
Activation	0.795	<0.001	<0.001	[0.687, 0.884]	[0.783, 0.810]	0.225	<0.001
Cytotoxicity	0.742	<0.001	<0.001	[0.556, 0.841]	[0.730, 0.788]	0.276	<0.001

## Data Availability

The data presented in this study are available from the corresponding author upon reasonable request. The data are not publicly available due to ongoing analyses and planned future publications.

## Supplementary Materials

The following supporting information can be downloaded at: https://www.mdpi.com/article/10.3390/genes17101272/s1, Figure S1: Sampling schedule for different patients. Blue indicates the sampling time; Table S1: Data Quality Metrics, Table S2: GSEA gene set, Table S3: NicheNet-based ligand prioritization and predictive, Table S4: Differentially expressed genes in neutrophils between NET-high and NET-low groups, Table S5: Differentially expressed genes in T cells between NET-high and NET-low groups.

## Abbreviations

The following abbreviations are used in this manuscript:

APCs	Antigen-presenting cells
axi-cel	Axicabtagene ciloleucel
BaEoMa	Basophil/eosinophil/mast cell populations
CAM	Cell adhesion molecule
CAR	Chimeric antigen receptor
CR	Complete response
DEGs	Differentially expressed genes
GO	Gene Ontology
GSEA	Gene set enrichment analysis
GvHD	Graft-versus-host disease
KEGG	Kyoto Encyclopedia of Genes and Genomes
LTR	Long-term responders
NETs	Neutrophil extracellular traps
NR	Non-responders
R	Relapsed
scRNA-seq	Single-cell RNA sequencing
PBMCs	Peripheral blood mononuclear cells
PCA	Principal component analysis
PR	Partial response
TANs	Tumor-associated neutrophils
TME	Tumor microenvironment
