# Supplementary material for: NET-Associated Transcriptional Signatures and T-Cell Functional States During Allogeneic CAR-γδT-Cell Therapy: A Longitudinal Single-Cell Analysis
Source: Genes (Basel). 2026 Oct 13;17(10):1272. doi: 10.3390/genes17101272 (PMC13704038; doi:10.3390/genes17101272)

Supplementary Table S1：Data Quality Metrics

| orig.ident | cells_qc | median_genes | mean_genes | median_UMIs | mean_UMIs | median_pct_mt | mean_pct_mt | IQR_genes | IQR_UMIs | Doublet rate |
|---|---|---|---|---|---|---|---|---|---|---|
| GUXJ-7D | 5,902 | 1,222 | 1,234.08 | 2,798.5 | 3,309.70 | 5.44 | 5.53 | 529.75 | 1,619.75 | 5.29% |
| WAYL-14D | 14,438 | 1,128 | 1,233.75 | 2,431 | 3,203.65 | 4.39 | 4.12 | 570.75 | 1,640.5 | 6.14% |
| WAYL-28D | 12,342 | 853 | 950.86 | 2,121.5 | 2,682.08 | 1.11 | 1.82 | 406 | 1,467.75 | 6.12% |
| YUDS-14D | 12,642 | 632 | 677.82 | 2,247 | 4,283.40 | 3.83 | 3.58 | 527 | 5,469.75 | 6.38% |
| YUZH-14D | 9,997 | 1,331 | 1,412.38 | 2,810 | 3,664.64 | 3.99 | 3.95 | 1,117 | 3,485 | 6.66% |
| YUZH-28D | 16,004 | 1,590 | 1,692.87 | 3,421.5 | 4,348.91 | 3.14 | 3.32 | 907.25 | 2,868.25 | 6.30% |
| YUZH-7D | 6,518 | 1,224 | 1,504.63 | 2,973 | 4,293.96 | 3.15 | 3.89 | 891 | 2,795 | 6.29% |
| ZHWX-15D | 9,424 | 1,240 | 1,417.11 | 2,970.5 | 3,907.13 | 3.37 | 3.33 | 1,247.25 | 3,999.25 | 6.27% |
| ZHWX-22D | 8,541 | 992 | 1,198.46 | 2,277 | 3,216.79 | 2.15 | 2.63 | 881 | 2,928 | 6.66% |
| ZHWX-28D | 20,877 | 1,137 | 1,254.78 | 2,977 | 3,755.17 | 0.49 | 1.58 | 1,021 | 1,912 | 6.65% |
| ZHWX-7D | 14,995 | 1,359 | 1,508.65 | 3,611 | 4,730.80 | 5.74 | 5.84 | 1,569 | 5,143.5 | 6.82% |

Supplementary Table S2：GSEA gene set

| Gene set | Gene ID |
|---|---|
| NETS | "ALPL", "BST1", "CD93", "CEACAM3", "CREB5", "CRISPLD2", "CSF3R", "CYP4F3", "DYSF", "FCAR", "FCGR3B", "CPPED1", "FPR1", "FPR2", "G0S2", "HIST1H2BC", "HPSE", "CXCR1", "CXCR2", "KCNJ15", "LILRB2", "MGAM", "MME", "PDE4B", "S100A12", "SIGLEC5", "SLC22A4", "SLC25A37", "TECPR2", "TNFRSF10C", "VNN3", "AKT1", "AKT2", "ATG7", "CLEC6A", "CSF3", "CTSG", "CYBB", "DNASE1", "ELANE", "ENTPD4", "F3", "HMGB1", "IL17A", "IL1B", "IL6", "IL8", "IRAK4", "ITGAM", "ITGB2", "KCNN3", "MAPK1", "MAPK3", "MMP9", "MPO", "MTOR", "PADI4", "PTAFR", "PIK3CA", "RIPK1", "RIPK3", "SELP", "SELPLG", "SIGLEC14", "TLR2", "TLR4", "TLR7", "TLR8", "TNF" |
| Cytotoxic | "PRF1", "IFNG", "GNLY", "NKG7", "GZMB", "GZMA", "HZMH", "KLRK1", "KLRKB1", "KLRD1", "CTSW", "CST7", "CX3CR1", "FGFBP2", "S1PR5", "FCGR3A", "PLAC8", "C1orf21", "TGFBR3", "PLEK", "FGR", "KLRF1", "SPON2", "CD300A", "S1PR1", "FAM65B", "EFHD2", "STK38", "C1orf162", "SORL1", "EMP3", "ARL4C", "BIN2", "CCND3", "FCRL6", "SAMD3", "TRDC", "TYROBP", "KLRG1" |
| Exhaustion | "FCRL3", "CD27", "PRKCH", "B2M", "ITM2A", "TIGIT", "ID3", "GBP2", "PDCD1", "KLRK1", "HSPA1A", "SRGN", "TNFRSF9", "TMBIM6", "TNFRSF1B", "CADM1", "ACTB", "CD8A", "RGS2", "FAIM3", "EID1", "HSPB1", "RNF19A", "IFI16", "LYST", "PRF1", "STAT1", "UBC", "CD74", "IL2RG", "FYN", "PTPN6", "HLA-DRB1", "HNRNPC", "UBB", "CD8B", "HAVCR2", "IRF8", "LAG3", "ATP5B", "STAT3", "IGFLR1", "MGEA5", "HSPA1B", "COTL1", "VCAM1", "HLA-DMA", "PDE7B", "TBC1D4", "SNAP47", "RGS4", "CBLB", "TOX", "CALM2", "ATHL1", "SPDYE5", "DDX5", "SLA", "PTPRCAP", "IRF9", "MATR3", "LITAF", "TPI1", "ETV1", "PAM", "ARID4B", "NAB1", "RAPGEF6", "LDHA", "WARS", "RASSF5", "OSBPL3", "FAM3C", "TAP1", "HLA-DRB6", "FABP5", "CD200", "CTLA4", "SNX9", "ETNK1", "MALAT1", "ZDHHC6", "ARL6IP5", "DUSP2", "HLA-DQB1", "HNRNPK", "DGKH", "LRMP", "H3F3B", "IDH2", "TRAF5", "TBL1XR1", "ANKRD10", "ALDOA", "LSP1", "PTPN7", "NSUN2", "RNF149", "CD2", "SRSF1", "GOLPH3", "HLA-A", "LIMS1", "SDF4", "ROCK1", "EDEM1", "APLP2", "ITK", "TRIM22", "SPRY2", "ACTG1", "HLA-DPA1", "EWSR1", "SRSF4", "ESYT1", "LUC7L3", "ARNT", "GNAS", "ARF6", "ARPC5L", "NCOA3", "PAPOLA", "GFOD1", "GPR174", "DDX3X", "CAPRIN1", "ARPC2", "PDIA6", "SEMA4A", "CSDE1", "PSMB9", "NFATC1", "PTPN11", "AGFG1", "PCED1B", "CCL4L1", "CCND2", "CCL4L2", "CXCR6", "AKAP5", "IFNG", "MIR155HG", "ENTPD1", "TOX2", "CD7", "RAB27A", "ITGAE", "PHLDA1", "PAG1", "CSF1", "NBL1", "CCL3", "ILRB", "FASLG", "ZC3H12C", "MYO7A", "SIRPG", "GALNT1", "UBE2F", "DUSP4", "SYT11", "TRAC", "TNS3", "RDH10", "PTMS", "CXCL13", "KIR2DL4" |
| Proliferation | "AURKA", "BUB1", "CCNB1", "CCND1", "CCNE1", "DEK", "E2F1", "FEN1", "FOXM1", "H2AFZ", "HMGB2", "MCM2", "MCM3", "MCM4", "MCM5", "MCM6", "MKI67", "MYBL2", "PCNA", "PLK1", "TOP2A", "TYMS", "ZWINT" |
| activation | "CD69","IL2RA","TNFRSF9","ICOS","PDCD1","CTLA4","HAVCR2","LAG3","TIGIT", |

Supplementary Table S3：NicheNet-based ligand prioritization and predictive

performance

| Test_ligand | Auroc | Aupr | Aupr_corrected | Pearson |
|---|---|---|---|---|
| MMP9 | 0.5821087 | 0.10364913 | 0.020648768 | 0.036677126 |
| PROK2 | 0.5911343 | 0.10311184 | 0.020111476 | 0.025825455 |
| ITGB2 | 0.5559265 | 0.09082362 | 0.007823257 | 0.030491616 |
| ITGAM | 0.5299898 | 0.08603022 | 0.003029858 | 0.007441784 |
| ALOX5AP | 0.5803206 | 0.09886149 | 0.015861131 | 0.06326991 |
| SELL | 0.5880055 | 0.10119719 | 0.018196827 | 0.031598504 |
| SELPLG | 0.5858071 | 0.09752244 | 0.014522076 | 0.034413411 |
| GRN | 0.5656529 | 0.09702299 | 0.014022626 | 0.051832976 |
| HLA-E | 0.597099 | 0.10862905 | 0.025628688 | 0.036625615 |
| PTGS2 | 0.5962752 | 0.1067738 | 0.023773439 | 0.043960214 |
| ICAM3 | 0.5969173 | 0.10119538 | 0.01819502 | 0.038101264 |
| HLA-C | 0.5869529 | 0.1013739 | 0.018373541 | 0.074403773 |
| OSM | 0.6014191 | 0.10645045 | 0.023450082 | 0.063503411 |
| CMTM2 | 0.5814561 | 0.09708315 | 0.014082791 | 0.053998011 |
| CXCL1 | 0.5681753 | 0.09513069 | 0.012130326 | 0.022314805 |
| NAMPT | 0.6170814 | 0.11387216 | 0.030871801 | 0.040015845 |
| SERPINA1 | 0.6008418 | 0.1062305 | 0.023230138 | 0.07616235 |
| TGFB1 | 0.5974197 | 0.10628357 | 0.023283208 | 0.024336036 |
| HMGB2 | 0.5756239 | 0.09558447 | 0.012584108 | -0.011961377 |
| FBRSL1 | 0.5658947 | 0.09127951 | 0.008279144 | 0.049103166 |
| IL1B | 0.6379934 | 0.11976614 | 0.036765774 | 0.034532237 |
| TNFSF8 | 0.5836165 | 0.09817949 | 0.015179128 | 0.033281151 |
| IL1RN | 0.588701 | 0.10248344 | 0.019483077 | 0.036885751 |
| COL18A1 | 0.6025417 | 0.10824393 | 0.025243565 | 0.092531053 |
| HLA-B | 0.5685429 | 0.09571154 | 0.012711175 | 0.049202438 |
| HLA-F | 0.5865761 | 0.09801257 | 0.015012202 | 0.073339772 |
| LRRC4 | 0.5684292 | 0.09565185 | 0.01265149 | 0.049395718 |
| ITGAL | 0.5883884 | 0.09886717 | 0.015866803 | 0.00250982 |
| TNFSF13B | 0.5782686 | 0.09976114 | 0.016760773 | 0.033418643 |
| CD58 | 0.5769669 | 0.09584448 | 0.01284412 | 0.026477962 |
| ADM | 0.5973654 | 0.10633454 | 0.023334179 | 0.038455502 |
| PLEKHO2 | 0.5941852 | 0.10771034 | 0.024709976 | 0.090344943 |
| IL16 | 0.5903209 | 0.10054201 | 0.017541644 | 0.064461364 |
| UTS2 | 0.5487775 | 0.08897415 | 0.005973784 | -0.005098265 |

Supplementary Table S4：Differentially expressed genes in neutrophils between NET-high and NET-low groups

| Gene | P_val | Avg_log2FC | Pct.1 | Pct.2 | P_val_adj |
|---|---|---|---|---|---|
| ALPL | 0 | 0.771667418840313 | 0.801 | 0.517 | 0 |
| MMP9 | 0 | 1.08090925221737 | 0.659 | 0.381 | 0 |
| MGAM | 0 | 0.788648550056898 | 0.583 | 0.343 | 0 |
| DYSF | 0 | 0.614884147241434 | 0.684 | 0.45 | 0 |
| S100A12 | 0 | 0.450876776327146 | 0.941 | 0.732 | 0 |
| FPR1 | 0 | 0.392883554289312 | 0.982 | 0.872 | 0 |
| PROK2 | 1.27859562422134e-302 | 0.700302221796049 | 0.638 | 0.408 | 3.15327252845468e-298 |
| MME | 2.60304684677237e-302 | 0.821590399576585 | 0.477 | 0.256 | 6.41963413351001e-298 |
| TLR4 | 6.92681126819798e-286 | 0.860965488147926 | 0.454 | 0.244 | 1.70829019496298e-281 |
| PADI4 | 7.7110048520358e-285 | 0.7731723990237 | 0.545 | 0.332 | 1.90168801660907e-280 |

| Gene | P_val | Avg_log2FC | Pct.1 | Pct.2 | P_val_adj |
|---|---|---|---|---|---|
| BASP1 | 4.8364055754086e-282 | 0.447528549401501 | 0.909 | 0.754 | 1.19275434300727e-277 |
| SLC11A1 | 1.13831889836019e-277 | 0.590853187225145 | 0.787 | 0.592 | 2.80732206713589e-273 |
| CXCR1 | 4.18737967739448e-274 | 0.683588069034033 | 0.535 | 0.311 | 1.03269157603903e-269 |
| ITGB2 | 7.36408250210985e-257 | 0.527201510515209 | 0.798 | 0.602 | 1.81613002667033e-252 |
| CD177 | 1.28896115206424e-255 | 0.627365788118512 | 0.683 | 0.48 | 3.17883599322082e-251 |
| CYP4F3 | 5.24475168067596e-255 | 0.829064203251202 | 0.45 | 0.257 | 1.29346065948831e-250 |
| SPI1 | 5.35934809145742e-234 | 0.431443579229487 | 0.921 | 0.81 | 1.32172242631523e-229 |
| KCNJ15 | 6.51986819953538e-233 | 0.540568637246259 | 0.597 | 0.383 | 1.60792989536941e-228 |
| TNFRSF10C | 1.10457720124829e-216 | 0.71872197492924 | 0.431 | 0.246 | 2.72410829371852e-212 |
| CST7 | 1.04201177400564e-210 | 0.494203390027433 | 0.698 | 0.5 | 2.56980943705271e-206 |
| FCAR | 2.78117689922052e-207 | 0.816869034722825 | 0.349 | 0.188 | 6.85893846885765e-203 |
| RGS2 | 4.36173315070083e-207 | 0.530051513489031 | 0.775 | 0.618 | 1.07569062962584e-202 |
| PYGL | 9.06715024918356e-207 | 0.531309646934905 | 0.696 | 0.516 | 2.23614059445365e-202 |
| ITGAM | 8.33233759220112e-200 | 0.618837395880659 | 0.52 | 0.335 | 2.05492109698864e-195 |
| VNN3 | 2.1721197517767e-199 | 0.865253399383306 | 0.292 | 0.142 | 5.3568817318317e-195 |
| CXCR2 | 1.93858727666971e-196 | 0.37536015036894 | 0.736 | 0.517 | 4.78094394172285e-192 |
| CDA | 2.3516758798711e-196 | 0.594956053504519 | 0.618 | 0.443 | 5.7997030549381e-192 |
| BST1 | 6.06837170156988e-195 | 0.690516402827132 | 0.463 | 0.288 | 1.49658182904116e-190 |
| LILRB2 | 7.52683057128158e-194 | 0.569711861762899 | 0.563 | 0.374 | 1.85626695548946e-189 |
| S100A9 | 1.3939312267387e-190 | 0.239989726179422 | 1 | 0.994 | 3.43771319138297e-186 |
| NCF4 | 1.62904858528681e-190 | 0.509019176869232 | 0.716 | 0.548 | 4.01755962103434e-186 |
| QPCT | 5.38857931886273e-185 | 0.628051683276025 | 0.473 | 0.299 | 1.32893143161793e-180 |
| G0S2 | 1.37826559850067e-182 | 0.344498980005986 | 0.934 | 0.789 | 3.39907861902236e-178 |
| LRP10 | 4.11240773216797e-182 | 0.597373784534276 | 0.571 | 0.399 | 1.01420199490726e-177 |
| ANPEP | 4.8986097863697e-182 | 0.586200886676025 | 0.564 | 0.391 | 1.2080951455145e-177 |
| LSP1 | 1.42501064548018e-175 | 0.444907430834981 | 0.833 | 0.708 | 3.51436125388322e-171 |
| CREB5 | 1.9080769183889e-173 | 0.428503864748929 | 0.603 | 0.409 | 4.70569929613069e-169 |
| CEACAM3 | 4.17037528466652e-173 | 0.761094939029319 | 0.349 | 0.2 | 1.02849795270446e-168 |
| MMP25 | 4.83818458553272e-172 | 0.494786111131357 | 0.616 | 0.439 | 1.19319308248408e-167 |
| GAPDH | 1.84924089783869e-171 | 0.362512318195792 | 0.858 | 0.708 | 4.56059790224977e-167 |
| NCF1 | 2.93312154099258e-166 | 0.440161679424754 | 0.902 | 0.821 | 7.2336643443959e-162 |
| FPR2 | 3.82824789817953e-163 | 0.592258808714142 | 0.418 | 0.256 | 9.44122496649036e-159 |

| Gene | P_val | Avg_log2FC | Pct.1 | Pct.2 | P_val_adj |
|---|---|---|---|---|---|
| LILRB3 | 6.09507059623351e-163 | 0.474271785011503 | 0.694 | 0.534 | 1.50316631044311e-158 |
| SLC2A3 | 1.30631494849691e-161 | 0.403704664680121 | 0.738 | 0.579 | 3.22163392598307e-157 |
| C5AR1 | 1.66518719786718e-158 | 0.40344726206951 | 0.779 | 0.625 | 4.10668466738003e-154 |
| S100A11 | 3.01440805319806e-155 | 0.254485472534369 | 0.988 | 0.93 | 7.43413314079705e-151 |
| ALOX5AP | 4.35878901184647e-155 | 0.361619459965981 | 0.829 | 0.681 | 1.07496454610158e-150 |
| VIM | 9.25436134724417e-155 | 0.42758420725115 | 0.829 | 0.686 | 2.28231059545736e-150 |
| FCGR3B | 2.80624277357722e-151 | 0.258772911088296 | 0.99 | 0.886 | 6.92075592819614e-147 |
| SELL | 2.96309731807535e-151 | 0.298216195294531 | 0.929 | 0.801 | 7.30759060583743e-147 |
| SELPLG | 9.75735001198565e-151 | 0.529800225570535 | 0.475 | 0.312 | 2.4063576599559e-146 |
| GRN | 4.54494087133847e-148 | 0.548101935426199 | 0.688 | 0.548 | 1.12087331768949e-143 |
| ZYX | 7.4194241785591e-147 | 0.388844412010842 | 0.778 | 0.628 | 1.82977839091624e-142 |
| MAPK3 | 8.18667331911361e-147 | 0.82200976434539 | 0.243 | 0.124 | 2.0189973739598e-142 |
| VNN2 | 4.12378147969026e-146 | 0.331249326583939 | 0.78 | 0.596 | 1.01700698852121e-141 |
| ADGRG3 | 7.45825013961508e-146 | 0.446381944263523 | 0.626 | 0.471 | 1.83935364943187e-141 |
| PPP1R15A | 5.66184393747225e-140 | 0.448343317973128 | 0.68 | 0.533 | 1.39632395185941e-135 |
| SULT1A1 | 4.71523491979836e-139 | 0.788711866124072 | 0.319 | 0.19 | 1.16287123592067e-134 |
| ACSL1 | 1.12531995392832e-135 | 0.275706382424192 | 0.863 | 0.714 | 2.77526407037803e-131 |
| HSPA1A | 1.88374269809401e-135 | 0.54910397666599 | 0.467 | 0.317 | 4.64568624203945e-131 |
| IL4R | 4.47814254296551e-135 | 0.591119491324378 | 0.437 | 0.295 | 1.10439951394615e-130 |
| FLOT2 | 4.48079010733852e-129 | 0.507778812845657 | 0.483 | 0.339 | 1.10505245627182e-124 |
| TKT | 1.61412715242115e-126 | 0.39562859399641 | 0.74 | 0.599 | 3.98076038330104e-122 |
| ARHGAP9 | 2.45118155335341e-126 | 0.476471724738061 | 0.481 | 0.334 | 6.04510394688017e-122 |
| CKAP4 | 3.20144298397529e-125 | 0.555854912393309 | 0.456 | 0.316 | 7.89539868707986e-121 |
| SLC22A4 | 4.02143244834113e-125 | 0.815778648059857 | 0.202 | 0.1 | 9.91765670409889e-121 |
| HLA-E | 1.73718908164417e-123 | 0.288443464526928 | 0.978 | 0.938 | 4.28425571315085e-119 |
| ABTB1 | 6.38022556182972e-123 | 0.46636109566103 | 0.592 | 0.452 | 1.57349122805845e-118 |
| MCEMP1 | 2.48315044152228e-120 | 0.602008155016002 | 0.408 | 0.277 | 6.12394561888225e-116 |
| PTGS2 | 3.93085539842266e-120 | 0.545878629462901 | 0.346 | 0.215 | 9.69427558358996e-116 |
| RNF10 | 1.10654493906557e-118 | 0.383499432844727 | 0.569 | 0.414 | 2.7289611287235e-114 |
| VSIR | 1.85861939350796e-118 | 0.292594048207111 | 0.847 | 0.724 | 4.58372714826934e-114 |
| LIMD2 | 1.1901427598343e-117 | 0.426232750141766 | 0.578 | 0.427 | 2.93513007430336e-113 |
| TUBA1A | 1.52385570228505e-117 | 0.358167999966986 | 0.693 | 0.539 | 3.7581329329754e-113 |

| Gene | P_val | Avg_log2FC | Pct.1 | Pct.2 | P_val_adj |
|---|---|---|---|---|---|
| CLEC4E | 2.55153840411904e-116 | 0.331968197304861 | 0.641 | 0.479 | 6.29260401223837e-112 |
| STXBP2 | 5.99404425621575e-115 | 0.4295793258088 | 0.539 | 0.397 | 1.47825119446793e-110 |
| TLR8 | 1.66914138756241e-114 | 0.635893532497247 | 0.266 | 0.153 | 4.11643649000642e-110 |
| JAML | 1.74979345251886e-114 | 0.31342716633185 | 0.783 | 0.633 | 4.31534061260202e-110 |
| MYL6 | 1.82036025481247e-114 | 0.324119212402483 | 0.896 | 0.798 | 4.4893724604185e-110 |
| TP53I11 | 4.32213264363656e-114 | 0.86679370102453 | 0.222 | 0.122 | 1.06592435257365e-109 |
| MAP4K4 | 4.9629362232938e-114 | 0.396587107193348 | 0.493 | 0.348 | 1.22395933138872e-109 |
| FRAT2 | 7.76667395775549e-114 | 0.467870121950068 | 0.456 | 0.314 | 1.91541713146166e-109 |
| ICAM3 | 9.39216429806078e-113 | 0.454315173931892 | 0.605 | 0.475 | 2.31629555918775e-108 |
| CD93 | 2.15943624549874e-111 | 0.5974281294278 | 0.269 | 0.156 | 5.32560166864898e-107 |
| CORO1A | 5.35042315053065e-111 | 0.377507408246268 | 0.81 | 0.702 | 1.31952135738387e-106 |
| HLA-C | 1.35632053507078e-110 | 0.24869549051045 | 0.995 | 0.978 | 3.34495770359157e-106 |
| JUNB | 3.04953843247438e-110 | 0.26948253830279 | 0.944 | 0.859 | 7.52077168216831e-106 |
| TLR2 | 1.39169729403985e-109 | 0.398615394453872 | 0.488 | 0.343 | 3.43220386656108e-105 |
| HCLS1 | 1.7825467685397e-109 | 0.379636478964868 | 0.722 | 0.602 | 4.39611684057262e-105 |
| ARHGAP4 | 1.38480720945969e-108 | 0.511559546679048 | 0.403 | 0.279 | 3.41521153996948e-104 |
| AL139042.1 | 1.57408740080412e-107 | 0.594205793823129 | 0.241 | 0.137 | 3.88201434786313e-103 |
| APMAP | 3.54291185329023e-107 | 0.611000858642438 | 0.342 | 0.224 | 8.73752921258435e-103 |
| PLP2 | 8.75943120703533e-106 | 0.385539853093871 | 0.565 | 0.419 | 2.16025092427905e-101 |
| ALOX5 | 1.44154920642083e-105 | 0.302267939648351 | 0.732 | 0.59 | 3.55514865287504e-101 |
| METTL9 | 5.84192296465975e-105 | 0.408565773657678 | 0.521 | 0.378 | 1.44073504154439e-100 |
| CRISPLD2 | 5.88936228949329e-105 | 0.709793915023567 | 0.202 | 0.107 | 1.45243452783484e-100 |
| MCL1 | 1.96434249445329e-104 | 0.277826120271646 | 0.855 | 0.741 | 4.8444614598207e-100 |
| RUBCNL | 3.47463565147376e-103 | 0.4247910973942 | 0.367 | 0.236 | 8.56914644366458e-99 |
| KLF6 | 1.13172774822836e-102 | 0.354041400866651 | 0.795 | 0.686 | 2.79106697268078e-98 |
| CD14 | 2.73261546705205e-102 | 0.266250187886439 | 0.672 | 0.506 | 6.73917626484376e-98 |
| FGD3 | 5.16502242702604e-101 | 0.436911906384861 | 0.402 | 0.275 | 1.27379783095316e-96 |
| LINC00937 | 5.1644251484573e-100 | 0.525175668234118 | 0.328 | 0.212 | 1.27365053011254e-95 |
| UBC | 2.82752546692598e-99 | 0.334544196880404 | 0.93 | 0.869 | 6.97324330653284e-95 |
| S1PR4 | 1.23408323330314e-98 | 0.564101030850026 | 0.324 | 0.21 | 3.0434960699722e-94 |
| TREM1 | 1.75718362879665e-98 | 0.3193764710745 | 0.617 | 0.465 | 4.3335662653383e-94 |
| MYADM | 1.45379846597252e-97 | 0.475280091241812 | 0.476 | 0.348 | 3.58535777678142e-93 |

| Gene | P_val | Avg_log2FC | Pct.1 | Pct.2 | P_val_adj |
|---|---|---|---|---|---|
| CD44 | 1.64440571774292e-97 | 0.334373409382364 | 0.574 | 0.431 | 4.05543338109759e-93 |
| CCDC88B | 1.89502914304121e-97 | 0.526411630090543 | 0.358 | 0.243 | 4.67352087256824e-93 |
| ZNF467 | 4.41309111256349e-96 | 0.571001432698124 | 0.267 | 0.163 | 1.08835653018041e-91 |
| CLEC4D | 5.07257981282444e-96 | 0.441066483422482 | 0.396 | 0.271 | 1.25099963343876e-91 |
| FKBP8 | 8.38344841180808e-96 | 0.45952631768544 | 0.436 | 0.311 | 2.06752604732011e-91 |
| LILRA6 | 3.88056371490109e-94 | 0.495459954952615 | 0.368 | 0.248 | 9.57024623368908e-90 |
| GNB2 | 1.48745881876268e-93 | 0.304216910590464 | 0.728 | 0.606 | 3.66837093883252e-89 |
| CEBPD | 1.31585361735298e-92 | 0.375418381762793 | 0.566 | 0.434 | 3.24515819111592e-88 |
| ADAM8 | 4.49693360658564e-92 | 0.353889013701901 | 0.548 | 0.417 | 1.10903376605615e-87 |
| FTH1 | 5.54563940253911e-92 | 0.198166532616844 | 0.998 | 0.963 | 1.3676655894542e-87 |
| CPPED1 | 5.63141557024369e-92 | 0.197290013747048 | 0.55 | 0.394 | 1.3888197079335e-87 |
| CSRNP1 | 2.75522817243525e-91 | 0.440604304812318 | 0.378 | 0.256 | 6.79494371885981e-87 |
| PNPLA2 | 7.20603651102e-91 | 0.597406189479145 | 0.304 | 0.201 | 1.77715272434775e-86 |
| PDLIM7 | 1.65953156266476e-90 | 0.436585791076629 | 0.361 | 0.244 | 4.09273673984384e-86 |
| FMNL1 | 6.89429799874624e-90 | 0.335644378404553 | 0.655 | 0.531 | 1.7002717724508e-85 |
| SIGLEC5 | 7.1813603666035e-90 | 0.985592774268033 | 0.119 | 0.053 | 1.77106709361176e-85 |
| RBP7 | 1.26132662668639e-89 | 0.676173645301315 | 0.203 | 0.115 | 3.11068372673398e-85 |
| CTSD | 1.91070081169671e-89 | 0.464195374135715 | 0.55 | 0.436 | 4.71217034180642e-85 |
| SLC37A3 | 4.20305885795378e-89 | 0.747830806447426 | 0.18 | 0.098 | 1.03655837554856e-84 |
| TUBA4A | 7.6445282521498e-89 | 0.470558064894522 | 0.444 | 0.326 | 1.88529355754518e-84 |
| TRIB1 | 1.19961416387061e-87 | 0.368820539787078 | 0.513 | 0.38 | 2.95848845093769e-83 |
| IL1R2 | 2.46800434050603e-87 | 0.274708945674274 | 0.581 | 0.436 | 6.08659230455598e-83 |
| WSB1 | 3.90051237916801e-87 | 0.348250157476288 | 0.586 | 0.459 | 9.61944362950415e-83 |
| FCGRT | 3.91366693260274e-87 | 0.438917401676031 | 0.424 | 0.305 | 9.65188538918487e-83 |
| BCL2A1 | 8.5826180002865e-87 | 0.269540529185642 | 0.861 | 0.726 | 2.11664525123066e-82 |
| NBEAL2 | 1.73171040558252e-86 | 0.399677769199713 | 0.447 | 0.331 | 4.2707442022476e-82 |
| ZNF394 | 2.09775898613022e-86 | 0.467594865048568 | 0.401 | 0.288 | 5.17349321159436e-82 |
| HPSE | 5.13809155402415e-86 | 0.807913906236274 | 0.152 | 0.078 | 1.26715613905344e-81 |
| NLRP12 | 9.45373064046461e-86 | 0.72502172076548 | 0.187 | 0.105 | 2.33147905055138e-81 |
| THEMIS2 | 1.07115711375421e-85 | 0.322431771189863 | 0.579 | 0.443 | 2.64168767394064e-81 |
| CR1 | 1.55387921107137e-85 | 0.241272240337127 | 0.679 | 0.527 | 3.83217691034422e-81 |
| USP10 | 2.19783539456612e-85 | 0.451147330428131 | 0.33 | 0.22 | 5.42030165007896e-81 |

| Gene | P_val | Avg_log2FC | Pct.1 | Pct.2 | P_val_adj |
|---|---|---|---|---|---|
| GRAMD1A | 3.50753910332274e-85 | 0.510146017588822 | 0.276 | 0.176 | 8.65029293661455e-81 |
| SLC25A37 | 3.77078865182985e-85 | 0.186544619424246 | 0.905 | 0.764 | 9.29951897314277e-81 |
| SHKBP1 | 6.70829527558072e-85 | 0.350085960872269 | 0.561 | 0.434 | 1.65439978086372e-80 |
| DAPK2 | 2.56942954234895e-84 | 0.525930446801303 | 0.246 | 0.152 | 6.33672713734098e-80 |
| MBOAT7 | 3.12167895490742e-84 | 0.309168265418549 | 0.633 | 0.501 | 7.69868463859269e-80 |
| GRK6 | 7.66969587448944e-84 | 0.442762844956284 | 0.376 | 0.265 | 1.89150039656659e-79 |
| AQP9 | 1.98091895076758e-83 | 0.233653878594151 | 0.763 | 0.609 | 4.88534231638301e-79 |
| OSM | 4.00234122808297e-83 | 0.569001958567816 | 0.225 | 0.135 | 9.87057393669823e-79 |
| ACAP1 | 1.321062249925e-81 | 0.473796163738165 | 0.364 | 0.259 | 3.25800372076504e-77 |
| PTAFR | 1.77545534493143e-81 | 0.336621060673614 | 0.47 | 0.345 | 4.37862797166988e-77 |
| IFRD1 | 2.12021759517529e-81 | 0.305368544099755 | 0.449 | 0.325 | 5.2288806332213e-77 |
| IL17RA | 3.49892301563809e-81 | 0.344333511160992 | 0.588 | 0.466 | 8.62904394116665e-77 |
| JMJD6 | 7.0245353770636e-81 | 0.417551234578314 | 0.366 | 0.254 | 1.73239091469142e-76 |
| SIPA1 | 5.04632947906522e-80 | 0.459498712077541 | 0.329 | 0.224 | 1.24452577612706e-75 |
| CTSH | 7.3883264189999e-80 | 0.750866243178874 | 0.186 | 0.107 | 1.82210906145376e-75 |
| MEGF9 | 9.23872125511947e-80 | 0.330973151938765 | 0.556 | 0.435 | 2.27845343593756e-75 |
| CD55 | 1.14531417957603e-79 | 0.27137661579055 | 0.669 | 0.536 | 2.8245738296704e-75 |
| ORAI2 | 2.26478507356462e-79 | 0.446515378542466 | 0.391 | 0.282 | 5.58541294842507e-75 |
| CSF3R | 7.53132134426716e-79 | 0.149347340825256 | 0.996 | 0.961 | 1.85737446992317e-74 |
| TUBB4B | 7.69705705923826e-79 | 0.528538896577376 | 0.291 | 0.194 | 1.89824821194934e-74 |
| F5 | 8.01775488102761e-79 | 0.432008853737297 | 0.306 | 0.203 | 1.97733870875903e-74 |
| LRG1 | 1.75896316456625e-78 | 0.323870090467452 | 0.503 | 0.377 | 4.33795495645328e-74 |
| DOK3 | 1.81685942524401e-78 | 0.328362183201115 | 0.478 | 0.356 | 4.48073871453677e-74 |
| GYG1 | 2.87119588324587e-78 | 0.360264968401971 | 0.48 | 0.361 | 7.08094328726096e-74 |
| RGS14 | 2.96245772540485e-78 | 0.68599459480008 | 0.2 | 0.119 | 7.30601324239344e-74 |
| NCF2 | 5.36487881230265e-78 | 0.250018902049646 | 0.788 | 0.669 | 1.32308641269008e-73 |
| FUT7 | 8.58093455400063e-77 | 0.717590709159343 | 0.154 | 0.083 | 2.11623007970763e-72 |
| FCER1G | 1.23805793570759e-76 | 0.249184133426019 | 0.87 | 0.758 | 3.05329848104207e-72 |
| DHRS7 | 3.73484501815399e-76 | 0.370817394817098 | 0.392 | 0.28 | 9.21087478377137e-72 |
| BTG1 | 3.29537505331059e-75 | 0.272066953493786 | 0.742 | 0.625 | 8.12705395647457e-71 |
| FRMD4B | 6.58602554849685e-75 | 0.6999736809523 | 0.187 | 0.11 | 1.62424562077029e-70 |
| FCN1 | 1.96569932630049e-74 | 0.265194037158198 | 0.625 | 0.495 | 4.84780767852226e-70 |

| Gene | P_val | Avg_log2FC | Pct.1 | Pct.2 | P_val_adj |
|---|---|---|---|---|---|
| TCIRG1 | 2.65102071213802e-74 | 0.341040404252077 | 0.454 | 0.343 | 6.53794728027477e-70 |
| RTN3 | 3.21310000283932e-74 | 0.261894564075794 | 0.692 | 0.571 | 7.92414722700234e-70 |
| NINJ1 | 8.85328233522929e-74 | 0.267336580447884 | 0.582 | 0.454 | 2.18339648951425e-69 |
| SAMSN1 | 1.56561441902502e-73 | 0.244654812072306 | 0.694 | 0.567 | 3.8611182801995e-69 |
| FOS | 3.56298464846682e-73 | 0.233996731346259 | 0.889 | 0.791 | 8.78703274004888e-69 |
| TSC22D4 | 5.15838139878833e-73 | 0.378307441391107 | 0.401 | 0.29 | 1.27216002056918e-68 |
| HK3 | 7.71881280558289e-73 | 0.410534865410197 | 0.407 | 0.299 | 1.90361361411285e-68 |
| UNC13D | 4.38589814490396e-72 | 0.401442753659563 | 0.343 | 0.242 | 1.08165020049621e-67 |
| UBALD2 | 1.09026096918932e-71 | 0.257752527055612 | 0.653 | 0.526 | 2.6888016022147e-67 |
| GNAS | 1.16961807192434e-71 | 0.354217494982304 | 0.498 | 0.384 | 2.8845120889798e-67 |
| ECE1 | 3.7197943675696e-71 | 0.289759675841128 | 0.463 | 0.346 | 9.17375686930015e-67 |
| AC005392.2 | 4.08901541675545e-71 | 0.822345680198214 | 0.137 | 0.072 | 1.00843298208023e-66 |
| BTNL3 | 4.49095380579231e-71 | 1.30421148451294 | 0.074 | 0.028 | 1.1075590275845e-66 |
| TMEM91 | 4.61741336764041e-71 | 0.552582483391926 | 0.225 | 0.142 | 1.13874648472748e-66 |
| GPSM3 | 5.25434334234203e-71 | 0.232579935457597 | 0.794 | 0.676 | 1.29582615508839e-66 |
| EHD1 | 5.68734810874373e-71 | 0.365307850646797 | 0.351 | 0.249 | 1.40261379057838e-66 |
| PILRA | 6.79891949768785e-71 | 0.28029742448957 | 0.525 | 0.399 | 1.67674952651978e-66 |
| IVNS1ABP | 1.20859592491656e-70 | 0.31904927921075 | 0.574 | 0.459 | 2.98063927002923e-66 |
| PNRC1 | 2.62940906801032e-70 | 0.194267710641386 | 0.873 | 0.76 | 6.48464864352705e-66 |
| PFKFB3 | 2.90653250691775e-70 | 0.304213154039265 | 0.369 | 0.262 | 7.16809046856055e-66 |
| MAPK1 | 5.70178604065632e-70 | 0.368659508890583 | 0.299 | 0.203 | 1.40617447334666e-65 |
| MYH9 | 9.19533628911731e-70 | 0.340035125782967 | 0.605 | 0.498 | 2.26775383562211e-65 |
| TNIP1 | 1.16463688130577e-69 | 0.354801937678664 | 0.398 | 0.29 | 2.8722274766763e-65 |
| CMTM2 | 1.23092295221214e-69 | 0.195922392044923 | 0.677 | 0.535 | 3.03570218474558e-65 |
| H2BC21 | 1.2653584004265e-69 | 0.625778002390349 | 0.185 | 0.11 | 3.12062688713182e-65 |
| KIAA0319L | 2.73462289375191e-69 | 0.528201264599207 | 0.223 | 0.141 | 6.74412698057097e-65 |
| PLCB2 | 4.85369323680077e-69 | 0.429513379299349 | 0.358 | 0.259 | 1.19701782605981e-64 |
| FBXL5 | 2.2338742407569e-68 | 0.231971320882229 | 0.675 | 0.553 | 5.50918065255466e-64 |
| AP5B1 | 3.31332821466456e-68 | 0.373656658026293 | 0.402 | 0.296 | 8.17133004300575e-64 |
| MX1 | 3.88248220885472e-68 | 0.250817448056792 | 0.572 | 0.452 | 9.57497762347751e-64 |
| LY6E | 8.56738025970496e-68 | 0.18966462247508 | 0.84 | 0.726 | 2.11288731964844e-63 |
| CLIC1 | 1.45459894289622e-67 | 0.199696475254572 | 0.82 | 0.7 | 3.58733191297066e-63 |

| Gene | P_val | Avg_log2FC | Pct. 1 | Pct. 2 | P_val_adj |
|---|---|---|---|---|---|
| JUN | 1.6161959303683e-67 | 0.43707893264208 | 0.427 | 0.328 | 3.98586240347431e-63 |
| TECPR2 | 2.02712420867377e-67 | 0.385748619582882 | 0.239 | 0.152 | 4.99929372343126e-63 |
| ANXA11 | 2.91745710908405e-67 | 0.335120313041608 | 0.489 | 0.38 | 7.19503272242308e-63 |
| CAP1 | 6.45492894830037e-67 | 0.221103343347622 | 0.82 | 0.708 | 1.59191457722984e-62 |
| ARHGEF1 | 6.49747780376161e-67 | 0.333414155452997 | 0.408 | 0.302 | 1.60240797596369e-62 |
| CNN2 | 6.02024607682801e-66 | 0.253902931036799 | 0.653 | 0.533 | 1.48471308746732e-61 |
| ADAR | 9.19932854201154e-66 | 0.279388487149672 | 0.573 | 0.46 | 2.26873840503089e-61 |
| CHST2 | 1.26292124609149e-65 | 0.768507380869129 | 0.122 | 0.063 | 3.11461637711082e-61 |
| CXCL1 | 1.28267537417185e-65 | 0.715526972156766 | 0.152 | 0.086 | 3.16333400778261e-61 |
| DBNL | 1.33473021760857e-65 | 0.400394683161626 | 0.366 | 0.266 | 3.29171166266625e-61 |
| SLA | 1.4480377239815e-65 | 0.281881175262605 | 0.575 | 0.456 | 3.57115063488318e-61 |
| UBN1 | 3.86586651768454e-65 | 0.290988140020995 | 0.407 | 0.301 | 9.5340000059136e-61 |
| LILRA5 | 1.09643599565048e-64 | 0.3434051389946 | 0.43 | 0.321 | 2.70403045247321e-60 |
| C19orf38 | 1.62027253132329e-64 | 0.381060734559878 | 0.362 | 0.263 | 3.99591611674949e-60 |
| CDKN2D | 1.86555933792645e-64 | 0.237106766160161 | 0.682 | 0.564 | 4.60084243919421e-60 |
| CA4 | 1.95973578655363e-64 | 0.40240858840083 | 0.296 | 0.203 | 4.83310039679856e-60 |
| SLC12A9 | 2.67306607625273e-64 | 0.428059769985716 | 0.263 | 0.176 | 6.59231555725449e-60 |
| RGS19 | 3.117395657453e-64 | 0.323846536646179 | 0.481 | 0.37 | 7.6881211704106e-60 |
| AC004151.1 | 5.51717573257446e-64 | 0.334030865571274 | 0.401 | 0.3 | 1.36064587916751e-59 |
| DGAT2 | 5.93132614444201e-64 | 0.279938509678717 | 0.436 | 0.319 | 1.46278365374229e-59 |
| OASL | 6.91183039794715e-64 | 0.302916770723205 | 0.375 | 0.272 | 1.70459561274173e-59 |
| MAPK13 | 9.53795902057869e-64 | 0.624439568102098 | 0.142 | 0.078 | 2.35225145365512e-59 |
| MID1IP1 | 1.2572475112178e-63 | 0.572833180030472 | 0.228 | 0.15 | 3.10062381216534e-59 |
| PHC2 | 3.40647587156816e-63 | 0.239951242136528 | 0.506 | 0.387 | 8.40105079446139e-59 |
| ANKRD13D | 6.19834138777399e-63 | 0.381530342284121 | 0.286 | 0.197 | 1.52863495305282e-58 |
| RHOG | 7.81838091898839e-63 | 0.231834618193735 | 0.688 | 0.56 | 1.92816910224092e-58 |
| CHST15 | 1.18774205417619e-62 | 0.258942063846132 | 0.463 | 0.345 | 2.92920945400932e-58 |
| ARPC1B | 2.41773725384618e-62 | 0.253140080721532 | 0.615 | 0.498 | 5.96262361543544e-58 |
| ARHGAP30 | 6.52815495581028e-62 | 0.257591244230284 | 0.588 | 0.475 | 1.60997357520193e-57 |
| P2RX1 | 1.51367088363028e-61 | 0.376032095022468 | 0.459 | 0.359 | 3.73301513320899e-57 |
| RGL4 | 4.07804704129752e-60 | 0.368732238779093 | 0.414 | 0.314 | 1.0057279613248e-55 |
| LINC00671 | 4.23808447901574e-60 | 0.948678729936042 | 0.097 | 0.048 | 1.04519639421486e-55 |

| Gene | P_val | Avg_log2FC | Pct.1 | Pct.2 | P_val_adj |
|---|---|---|---|---|---|
| DDIT3 | 6.14571261046439e-60 | 0.330502244888738 | 0.277 | 0.188 | 1.51565564399273e-55 |
| TLE3 | 8.29319035051477e-60 | 0.327520319840236 | 0.323 | 0.228 | 2.04526660424395e-55 |
| EVI2B | 9.4914197139704e-60 | 0.189094858087313 | 0.845 | 0.728 | 2.34077392985938e-55 |
| NAMPT | 1.70234505458285e-59 | 0.138675308254741 | 0.998 | 0.948 | 4.19832337361224e-55 |
| TBC1D10C | 1.87310325558641e-59 | 0.406092937488681 | 0.26 | 0.177 | 4.6194472489272e-55 |
| XPO6 | 2.54883659787972e-59 | 0.158906559132875 | 0.689 | 0.554 | 6.28594081769096e-55 |
| CNTNAP3 | 8.65540508860686e-59 | 0.872564176830202 | 0.088 | 0.041 | 2.13459600295222e-54 |
| OAZ1 | 9.78457583396041e-59 | 0.207032143395226 | 0.862 | 0.772 | 2.41307209217132e-54 |
| RNPEPL1 | 1.13425118970856e-58 | 0.608289777850826 | 0.167 | 0.102 | 2.79729028405925e-54 |
| FAM160A2 | 3.09279394798779e-58 | 0.611910075337739 | 0.176 | 0.109 | 7.62744843452748e-54 |
| APOBR | 4.64753347869376e-58 | 0.296062434147055 | 0.476 | 0.373 | 1.14617470651546e-53 |
| IMPDH1 | 5.14984949825992e-58 | 0.426378143566034 | 0.245 | 0.165 | 1.27005588326086e-53 |
| TNFRSF1B | 6.80188798950561e-58 | 0.23227848360318 | 0.588 | 0.472 | 1.67748161597187e-53 |
| MYO1F | 7.1615873056147e-58 | 0.195330616733774 | 0.797 | 0.72 | 1.7661906613107e-53 |
| IFITM2 | 7.63311927126677e-58 | 0.188088515724777 | 0.997 | 0.968 | 1.88247987467981e-53 |
| IRF7 | 9.31796030093239e-58 | 0.280188431769184 | 0.395 | 0.295 | 2.29799536941595e-53 |
| MSL1 | 1.10230391227081e-56 | 0.294938419951865 | 0.395 | 0.295 | 2.71850190844227e-52 |
| NRBF2 | 1.15513032830235e-56 | 0.2966307782033 | 0.368 | 0.27 | 2.84878241565925e-52 |
| STK17B | 1.34969181835268e-56 | 0.167531090465154 | 0.802 | 0.692 | 3.32860996242139e-52 |
| SIGLEC14 | 3.91951386653784e-56 | 0.860874543627816 | 0.095 | 0.048 | 9.66630509765563e-52 |
| ANKRD34B | 4.35637649884461e-56 | 0.646140944663996 | 0.142 | 0.083 | 1.07436957214506e-51 |
| CCNJL | 5.45581695310786e-56 | 0.592530511886311 | 0.126 | 0.069 | 1.34551357697546e-51 |
| CTSS | 9.8831083069979e-56 | 0.153451839127052 | 0.901 | 0.831 | 2.43737217067182e-51 |
| BIN2 | 1.22353955619643e-55 | 0.263008382844475 | 0.573 | 0.465 | 3.01749325349164e-51 |
| RGS18 | 3.78637659565369e-55 | 0.286299045820305 | 0.358 | 0.261 | 9.33796196020112e-51 |
| IQSEC1 | 6.16401439951341e-55 | 0.19018934484346 | 0.472 | 0.362 | 1.520169231208e-50 |
| BMX | 1.04400145697405e-54 | 0.564679214138299 | 0.159 | 0.097 | 2.57471639318939e-50 |
| NATD1 | 1.27866675978624e-54 | 0.681899180009413 | 0.116 | 0.063 | 3.15344796298483e-50 |
| H2AC6 | 1.66371055738348e-54 | 0.230864413219869 | 0.473 | 0.363 | 4.10304297661915e-50 |
| APOBEC3A | 2.7283486109303e-54 | 0.195204913157806 | 0.472 | 0.362 | 6.72865334427632e-50 |
| AATK | 3.25200306666856e-54 | 0.432226482548466 | 0.158 | 0.096 | 8.020089963018e-50 |
| FFAR2 | 5.70197912904468e-54 | 0.167671127029675 | 0.741 | 0.616 | 1.406222092805e-49 |

| Gene | P_val | Avg_log2FC | Pct. 1 | Pct. 2 | P_val_adj |
|---|---|---|---|---|---|
| OXER1 | 9.09713462558071e-54 | 1.33330199802488 | 0.052 | 0.019 | 2.24353534136071e-49 |
| ANP32A | 9.13905679267131e-54 | 0.158658365719268 | 0.728 | 0.607 | 2.2538741862086e-49 |
| ZDHHC18 | 1.00666051336337e-53 | 0.257091635288364 | 0.434 | 0.33 | 2.48262615805674e-49 |
| FCGR2A | 1.15352397735962e-53 | 0.195069579116512 | 0.836 | 0.72 | 2.84482083296429e-49 |
| PREX1 | 2.4629604570144e-53 | 0.203197532220854 | 0.559 | 0.444 | 6.0741530790889e-49 |
| ITPRIP | 3.04761474339143e-53 | 0.373590063182518 | 0.28 | 0.198 | 7.51602748015194e-49 |
| MARCKS | 3.74770066547403e-53 | 0.169953748003084 | 0.535 | 0.424 | 9.24257938119204e-49 |
| HIP1 | 3.94849897650395e-53 | 0.380296358767577 | 0.259 | 0.18 | 9.73778817585403e-49 |
| ATG16L2 | 3.98841371912927e-53 | 0.254027502125246 | 0.494 | 0.389 | 9.83622591411661e-49 |
| PSTPIP1 | 5.17399398607822e-53 | 0.471171078731659 | 0.212 | 0.142 | 1.27601039684661e-48 |
| MARC1 | 1.50936546732477e-52 | 0.653698740886454 | 0.114 | 0.063 | 3.72239711551634e-48 |
| IL18RAP | 1.82118892275826e-52 | 0.281532976294575 | 0.326 | 0.237 | 4.49141612130643e-48 |
| PHF12 | 2.39254442937266e-52 | 0.334328833604605 | 0.228 | 0.152 | 5.90049307171885e-48 |
| PTPRE | 3.65854236705058e-52 | 0.17200996055213 | 0.664 | 0.546 | 9.02269718562015e-48 |
| CYSTM1 | 3.68001686364725e-52 | 0.250316126557185 | 0.612 | 0.506 | 9.07565758912684e-48 |
| MXD1 | 4.9538580043014e-52 | 0.155062009201886 | 0.842 | 0.73 | 1.22172046102081e-47 |
| LTB4R | 5.63691952214579e-52 | 0.465479997532699 | 0.241 | 0.167 | 1.3901770925516e-47 |
| DENND3 | 8.38653189066793e-52 | 0.218223433188709 | 0.415 | 0.315 | 2.06828649487653e-47 |
| SRPK1 | 9.2024757636865e-52 | 0.2770040787903 | 0.351 | 0.26 | 2.26951457284036e-47 |
| TSEN34 | 1.40351545333534e-51 | 0.254623933082076 | 0.36 | 0.263 | 3.46134981101562e-47 |
| STX10 | 2.45172731302502e-51 | 0.352726338965861 | 0.282 | 0.202 | 6.0464498993823e-47 |
| NFIL3 | 3.19120758174925e-51 | 0.22526718486994 | 0.436 | 0.333 | 7.87015613811e-47 |
| IGF2R | 4.41076069915177e-51 | 0.154593206531592 | 0.523 | 0.41 | 1.08778180362481e-46 |
| PXN | 4.42423723240781e-51 | 0.230277217862683 | 0.402 | 0.304 | 1.09110538625641e-46 |
| LBR | 5.00928219799594e-51 | 0.215892249398498 | 0.511 | 0.405 | 1.23538917566976e-46 |
| ZBED1 | 5.60818310254527e-51 | 0.665108866480863 | 0.111 | 0.061 | 1.38309011674972e-46 |
| SLC15A3 | 7.34468330513636e-51 | 0.366443265449759 | 0.224 | 0.151 | 1.81134579671273e-46 |
| CCDC71L | 7.60741430944982e-51 | 0.450801475478362 | 0.214 | 0.144 | 1.87614051699652e-46 |
| UNC93B1 | 9.36512877161244e-51 | 0.348275829947938 | 0.285 | 0.206 | 2.30962805765506e-46 |
| S100A6 | 9.8817676502011e-51 | 0.137067749204951 | 0.981 | 0.928 | 2.4370415378926e-46 |
| SIGLEC9 | 1.29657079214686e-50 | 0.376786678161989 | 0.235 | 0.161 | 3.19760288759259e-46 |
| CFLAR | 2.10631108757309e-50 | 0.176270365380895 | 0.746 | 0.638 | 5.19458440417276e-46 |

| Gene | P_val | Avg_log2FC | Pct.1 | Pct.2 | P_val_adj |
|---|---|---|---|---|---|
| NFE2 | 2.322127840735e-50 | 0.278408701139024 | 0.363 | 0.272 | 5.72683168082065e-46 |
| PLPPR2 | 2.54456246331378e-50 | 0.465346168104643 | 0.147 | 0.089 | 6.27539994702444e-46 |
| STAT5B | 4.01481225602833e-50 | 0.231114536478181 | 0.372 | 0.276 | 9.90132998581708e-46 |
| ABCA7 | 4.98666628322097e-50 | 0.500930113649109 | 0.168 | 0.106 | 1.22981163876796e-45 |
| AL159141.1 | 9.57371598919429e-50 | 0.250010556196007 | 0.358 | 0.268 | 2.3610698372551e-45 |
| MVP | 1.85747944108999e-49 | 0.246077643518863 | 0.307 | 0.223 | 4.58091579761613e-45 |
| SBNO2 | 2.06513507009326e-49 | 0.287200523226166 | 0.312 | 0.227 | 5.09303610986399e-45 |
| SYNE1 | 3.16514753177003e-49 | 0.254959816621744 | 0.423 | 0.33 | 7.80588684285125e-45 |
| SASH3 | 1.12661951685716e-48 | 0.226900347210128 | 0.353 | 0.263 | 2.77846905247313e-44 |
| STX11 | 1.19252727380005e-48 | 0.252632954025428 | 0.382 | 0.29 | 2.94101076264567e-44 |
| CDC42EP3 | 1.19944223374716e-48 | 0.222981605120157 | 0.498 | 0.393 | 2.95806443686724e-44 |
| INPP5D | 1.29613134589435e-48 | 0.246219293332954 | 0.337 | 0.249 | 3.19651912524466e-44 |
| PSD4 | 1.65719262279648e-48 | 0.581671335472314 | 0.138 | 0.083 | 4.08696844634067e-44 |
| APLP2 | 2.06927995344092e-48 | 0.221535988517663 | 0.52 | 0.411 | 5.103258221176e-44 |
| CD37 | 2.34131591932411e-48 | 0.192244822405784 | 0.688 | 0.574 | 5.77415332023711e-44 |
| IL18R1 | 2.68255550113642e-48 | 0.309296015511276 | 0.27 | 0.191 | 6.61571837690264e-44 |
| MCTP2 | 2.7263081460487e-48 | 0.155247970629169 | 0.454 | 0.35 | 6.7236211497853e-44 |
| SERPINA1 | 3.23076531686953e-48 | 0.167770904978128 | 0.89 | 0.783 | 7.96771342446363e-44 |
| TPD52L2 | 5.86704527720314e-48 | 0.374939681919437 | 0.22 | 0.15 | 1.44693070626384e-43 |
| PTPRN2 | 6.67086330222265e-48 | 0.564100676529325 | 0.14 | 0.085 | 1.64516830759415e-43 |
| PADI2 | 7.43287737479317e-48 | 0.316835641408105 | 0.297 | 0.217 | 1.83309621817149e-43 |
| POR | 8.80928819593394e-48 | 0.457109645325401 | 0.212 | 0.145 | 2.17254665488123e-43 |
| FOSL2 | 8.92890734874556e-48 | 0.260820438952039 | 0.345 | 0.255 | 2.20204713034763e-43 |
| CYBC1 | 1.0991602707513e-47 | 0.315723181255449 | 0.293 | 0.213 | 2.71074905972687e-43 |
| SHISA5 | 1.19385118161976e-47 | 0.213131774438889 | 0.547 | 0.438 | 2.94427578411065e-43 |
| LINC01001 | 1.64948424646247e-47 | 0.19969522274902 | 0.421 | 0.321 | 4.06795804862575e-43 |
| LPAR2 | 1.7170462062768e-47 | 0.291469865093705 | 0.227 | 0.155 | 4.23457935391984e-43 |
| LAT2 | 2.151481735787e-47 | 0.255143686974649 | 0.348 | 0.26 | 5.30598425679789e-43 |
| SLC44A2 | 2.78094716345759e-47 | 0.266275740185163 | 0.314 | 0.23 | 6.85837189451912e-43 |
| BCL3 | 3.53631423090388e-47 | 0.224933007063027 | 0.409 | 0.31 | 8.72125815625516e-43 |
| PARP10 | 4.61138336077812e-47 | 0.397690462021617 | 0.164 | 0.104 | 1.1372593644351e-42 |
| PPP4R2 | 4.66385316767608e-47 | 0.27443794126643 | 0.342 | 0.255 | 1.15019946821228e-42 |

| Gene | P_val | Avg_log2FC | Pct. 1 | Pct. 2 | P_val_adj |
|---|---|---|---|---|---|
| TLN1 | 5.19715787588219e-47 | 0.218543165320655 | 0.574 | 0.471 | 1.28172307535006e-42 |
| AC008105.3 | 7.68133419142837e-47 | 0.42538813387247 | 0.163 | 0.104 | 1.89437063829006e-42 |
| SESN2 | 9.48517069220927e-47 | 0.380290860957099 | 0.209 | 0.141 | 2.33923279611265e-42 |
| MAP1LC3B | 1.03061632905458e-46 | 0.173585325967735 | 0.654 | 0.541 | 2.5417059907144e-42 |
| H3-3B | 1.11034145219994e-46 | 0.123135508470553 | 0.971 | 0.916 | 2.7383240894155e-42 |
| TMEM71 | 1.14772711246816e-46 | 0.265476381876847 | 0.311 | 0.227 | 2.83052460476897e-42 |
| GMIP | 1.19035426265437e-46 | 0.280797872243727 | 0.29 | 0.209 | 2.93565168255821e-42 |
| NADK | 1.30595024138127e-46 | 0.19772110983424 | 0.507 | 0.405 | 3.22073448529448e-42 |
| CD82 | 1.3709640036361e-46 | 0.265572509199188 | 0.392 | 0.303 | 3.38107142576734e-42 |
| FLOT1 | 2.31521474457335e-46 | 0.204709561445963 | 0.566 | 0.465 | 5.70978260306681e-42 |
| ATG2A | 2.35319632468766e-46 | 0.244345984958278 | 0.338 | 0.255 | 5.80345277594471e-42 |
| MAP7D1 | 3.84468024742393e-46 | 0.323821553746402 | 0.276 | 0.199 | 9.4817504261969e-42 |
| NTNG2 | 5.01193637783099e-46 | 0.389375751967652 | 0.216 | 0.148 | 1.23604374950068e-41 |
| LCP1 | 5.21966092997269e-46 | 0.132011097839251 | 0.946 | 0.885 | 1.28727277854986e-41 |
| ARAP1 | 9.49925219471193e-46 | 0.204402656300806 | 0.445 | 0.348 | 2.34270557625986e-41 |
| PINK1 | 1.31977611956688e-45 | 0.555035717175336 | 0.135 | 0.083 | 3.25483186607583e-41 |
| GNAI2 | 1.44135248037602e-45 | 0.15757766162589 | 0.812 | 0.72 | 3.55466348710334e-41 |
| AKNA | 1.91232144752586e-45 | 0.187624533732312 | 0.364 | 0.271 | 4.71616715388829e-41 |
| RAC2 | 2.14068670737541e-45 | 0.170380945918493 | 0.76 | 0.649 | 5.27936155772923e-41 |
| PPP2R5A | 3.9926425819699e-45 | 0.268480096008187 | 0.344 | 0.258 | 9.84665513565418e-41 |
| SULT1A2 | 4.98253354021199e-45 | 0.908292612973244 | 0.059 | 0.026 | 1.22879242168708e-40 |
| MX2 | 5.43209305475276e-45 | 0.155517040267835 | 0.559 | 0.448 | 1.33966278916313e-40 |
| N4BP1 | 6.11020345426139e-45 | 0.192756148503552 | 0.428 | 0.331 | 1.50689837588994e-40 |
| NIN | 7.17977758506506e-45 | 0.171760604534035 | 0.481 | 0.381 | 1.77067674802875e-40 |
| DEF6 | 1.38154713987773e-44 | 0.279819096835811 | 0.256 | 0.182 | 3.40717155636646e-40 |
| ARG1 | 1.42287761392323e-44 | 0.33668254204338 | 0.261 | 0.189 | 3.50910077145746e-40 |
| MAPKAPK2 | 2.15165282619499e-44 | 0.316235761193671 | 0.248 | 0.177 | 5.30640619996209e-40 |
| PLEC | 2.43043242190259e-44 | 0.603474356246672 | 0.127 | 0.077 | 5.99393243889618e-40 |
| DEF8 | 3.26531281279875e-44 | 0.354624788660074 | 0.203 | 0.139 | 8.05291445892428e-40 |
| CYFIP2 | 3.5565962229584e-44 | 0.383043317196122 | 0.239 | 0.171 | 8.77127760506001e-40 |
| TYMP | 3.57107544138431e-44 | 0.141019630098624 | 0.709 | 0.603 | 8.80698625354199e-40 |
| PRAM1 | 3.87206524133636e-44 | 0.321760178084129 | 0.301 | 0.226 | 9.54928729818374e-40 |

| Gene | P_val | Avg_log2FC | Pct.1 | Pct.2 | P_val_adj |
|---|---|---|---|---|---|
| PGD | 3.88860910073166e-44 | 0.196973446605389 | 0.601 | 0.489 | 9.59008776422442e-40 |
| MIIP | 4.48832350770277e-44 | 0.554610017162348 | 0.148 | 0.094 | 1.10691034346966e-39 |
| STAT6 | 5.40050057594373e-44 | 0.245075273515523 | 0.367 | 0.279 | 1.33187145203924e-39 |
| TOM1 | 5.66654897809073e-44 | 0.205196368498371 | 0.394 | 0.302 | 1.39748430897674e-39 |
| MAP2K6 | 1.00050947550545e-43 | 0.40488074999569 | 0.189 | 0.128 | 2.46745646849153e-39 |
| ZNF281 | 1.06227099805199e-43 | 0.296908150658756 | 0.297 | 0.22 | 2.61977273539581e-39 |
| SNX3 | 1.18832065260071e-43 | 0.24407611164396 | 0.454 | 0.36 | 2.93063639344388e-39 |
| MSL3 | 1.4152876919532e-43 | 0.281295808211309 | 0.277 | 0.202 | 3.49038250589498e-39 |
| R3HDM4 | 1.51158609332481e-43 | 0.165049750698907 | 0.609 | 0.494 | 3.72787362335764e-39 |
| BEST1 | 2.23029233551825e-43 | 0.313989708494927 | 0.182 | 0.12 | 5.50034695785512e-39 |
| IFNGR1 | 4.07435614029328e-43 | 0.15259967671656 | 0.54 | 0.43 | 1.00481771131913e-38 |
| BCL6 | 4.16065804843779e-43 | 0.137156935303791 | 0.596 | 0.486 | 1.02610148790573e-38 |
| DDAH2 | 5.08304028622925e-43 | 0.320124904845155 | 0.265 | 0.192 | 1.25357939538986e-38 |
| MIR22HG | 7.1536644967334e-43 | 0.28152832822573 | 0.283 | 0.206 | 1.76423673818439e-38 |
| FAM157C | 1.29116817174685e-42 | 0.144659935663813 | 0.45 | 0.35 | 3.18427894516209e-38 |
| GTF2I | 1.31325548553834e-42 | 0.24288519812951 | 0.364 | 0.28 | 3.23875067843465e-38 |
| SLFN5 | 1.40339478299854e-42 | 0.423074943553527 | 0.174 | 0.116 | 3.461052213831e-38 |
| SLC43A2 | 1.74874523629886e-42 | 0.206367607972617 | 0.306 | 0.224 | 4.31275550176025e-38 |
| OAS1 | 2.09310040382605e-42 | 0.308195695815665 | 0.309 | 0.232 | 5.1620042159158e-38 |
| LIMK2 | 2.20764219413433e-42 | 0.136703962988082 | 0.696 | 0.584 | 5.44448717917407e-38 |
| IGSF6 | 3.19412947920232e-42 | 0.13904228966366 | 0.739 | 0.62 | 7.87736212160876e-38 |
| TGFB1 | 3.69275664555468e-42 | 0.179316483840795 | 0.516 | 0.414 | 9.10707643926696e-38 |
| YPEL3 | 5.14643523769819e-42 | 0.171658217569936 | 0.552 | 0.447 | 1.26921385832113e-37 |
| PYCARD | 5.5386904685409e-42 | 0.208192141914835 | 0.488 | 0.393 | 1.36595184335156e-37 |
| SULT1B1 | 6.2975411509878e-42 | 0.466414265911776 | 0.133 | 0.083 | 1.55309959865661e-37 |
| MAEA | 6.3451590145929e-42 | 0.378649244009287 | 0.173 | 0.114 | 1.5648431161789e-37 |
| HOMER3 | 1.08464349226177e-41 | 0.908070863368862 | 0.066 | 0.032 | 2.67494778061597e-37 |
| EIF2AK2 | 1.13530148076637e-41 | 0.208154406919327 | 0.459 | 0.365 | 2.79988051186601e-37 |
| CARD19 | 1.44326679561861e-41 | 0.301716130296206 | 0.284 | 0.21 | 3.55938457135461e-37 |
| PLIN3 | 1.59430080650778e-41 | 0.240610997099589 | 0.344 | 0.261 | 3.93186464900948e-37 |
| LILRA2 | 1.61729928911316e-41 | 0.243691922956743 | 0.374 | 0.288 | 3.98858350681088e-37 |
| STEAP4 | 1.8872053178579e-41 | 0.15465644027199 | 0.463 | 0.359 | 4.65422575490115e-37 |

| Gene | P_val | Avg_log2FC | Pct.1 | Pct.2 | P_val_adj |
|---|---|---|---|---|---|
| ADAM19 | 1.91734360706795e-41 | 0.601797627262568 | 0.08 | 0.042 | 4.72855280375098e-37 |
| HMGB2 | 1.95711011894857e-41 | 0.208144078662424 | 0.551 | 0.455 | 4.82662497535096e-37 |
| MICAL1 | 2.09310923959426e-41 | 0.412163822925691 | 0.229 | 0.164 | 5.16202600668737e-37 |
| RFLNB | 2.29193175660937e-41 | 0.227295562230092 | 0.383 | 0.294 | 5.65236209815003e-37 |
| ULK1 | 2.73987421919807e-41 | 0.382035281569753 | 0.179 | 0.12 | 6.75707779938629e-37 |
| APBB1IP | 4.03197092019652e-41 | 0.157614657649467 | 0.611 | 0.505 | 9.94364668338867e-37 |
| WBP2 | 5.97830622665742e-41 | 0.249137389176336 | 0.348 | 0.267 | 1.47436988161825e-36 |
| HELZ2 | 1.1298722157574e-40 | 0.334053555783527 | 0.226 | 0.161 | 2.78649085850091e-36 |
| H2BC4 | 1.26050730013741e-40 | 0.339123091634035 | 0.205 | 0.141 | 3.10866310359889e-36 |
| PRKD2 | 1.26082657362956e-40 | 0.198366028433439 | 0.251 | 0.18 | 3.10945049588523e-36 |
| RXRA | 1.34162251035831e-40 | 0.296320201587489 | 0.212 | 0.148 | 3.30870943504567e-36 |
| DHX34 | 1.6190386609232e-40 | 0.212105550415548 | 0.257 | 0.183 | 3.9928731455688e-36 |
| ZBTB7B | 1.74472260183604e-40 | 0.468481099198446 | 0.142 | 0.09 | 4.30283488064805e-36 |
| HEBP2 | 1.92417798363052e-40 | 0.232198328995159 | 0.381 | 0.298 | 4.74540774322958e-36 |
| CTDSP1 | 3.0262135597238e-40 | 0.397510955914791 | 0.188 | 0.129 | 7.46324788099083e-36 |
| FBRSL1 | 3.03852019078535e-40 | 0.258112120420086 | 0.264 | 0.191 | 7.49359849451482e-36 |
| GNG2 | 4.85444791264697e-40 | 0.142751325758889 | 0.38 | 0.29 | 1.19720394421699e-35 |
| RARA-AS1 | 5.23063550061545e-40 | 0.463076649439547 | 0.133 | 0.083 | 1.28997932716178e-35 |
| DUSP1 | 7.00913391361944e-40 | 0.102286537614004 | 0.96 | 0.88 | 1.72859260577683e-35 |
| TYK2 | 7.13328157724062e-40 | 0.326008146745395 | 0.228 | 0.163 | 1.75920990257908e-35 |
| AL021707.1 | 7.22371649401765e-40 | 0.431411706918769 | 0.119 | 0.072 | 1.78151296175463e-35 |
| MBD2 | 8.06417458214019e-40 | 0.233872142403993 | 0.292 | 0.216 | 1.98878673544741e-35 |
| ARID3A | 1.01274721323223e-39 | 0.415957944023154 | 0.185 | 0.127 | 2.49763717727332e-35 |
| DNM2 | 1.04645459342949e-39 | 0.252247539607454 | 0.273 | 0.202 | 2.5807663183158e-35 |
| COTL1 | 1.17035323249309e-39 | 0.111172837833778 | 0.842 | 0.742 | 2.88632514197447e-35 |
| ACTN1 | 1.29977702700032e-39 | 0.178606489583157 | 0.616 | 0.515 | 3.20551010398819e-35 |
| PLOD1 | 1.66635426237296e-39 | 0.639773825923221 | 0.097 | 0.056 | 4.1095628818642e-35 |
| VAPA | 3.92433126863591e-39 | 0.170589372995396 | 0.475 | 0.38 | 9.67818577470987e-35 |
| ATP6V0D1 | 4.76980463233435e-39 | 0.233500803721689 | 0.362 | 0.28 | 1.1763292184263e-34 |
| MIDN | 1.10720584715221e-38 | 0.149168251572969 | 0.507 | 0.404 | 2.73059106024677e-34 |
| IL1B | 1.13047544283351e-38 | 0.674137824791059 | 0.093 | 0.053 | 2.787978537116e-34 |
| ZNF787 | 1.20523179825985e-38 | 0.528856208588786 | 0.102 | 0.06 | 2.97234266086844e-34 |

| Gene | P_val | Avg_log2FC | Pct.1 | Pct.2 | P_val_adj |
|---|---|---|---|---|---|
| ARRB2 | 1.36587632640838e-38 | 0.154114254303927 | 0.733 | 0.636 | 3.36852419618834e-34 |
| FLII | 1.66370822685103e-38 | 0.309342640938601 | 0.241 | 0.176 | 4.10303722906002e-34 |
| MYO9B | 1.95626528951908e-38 | 0.198413650891676 | 0.429 | 0.344 | 4.82454145701196e-34 |
| KLF2 | 2.54092868582845e-38 | 0.202336148438436 | 0.376 | 0.29 | 6.26643832499012e-34 |
| CARS2 | 2.88451437228272e-38 | 0.287835051837348 | 0.249 | 0.182 | 7.11378934492364e-34 |
| PLAUR | 3.78694666938847e-38 | 0.164919403547405 | 0.52 | 0.423 | 9.33936787604585e-34 |
| KREMEN1 | 4.54200244677512e-38 | 0.209097610387366 | 0.227 | 0.16 | 1.12014864342368e-33 |
| AL021707.6 | 5.20833907302939e-38 | 0.701041408949562 | 0.058 | 0.028 | 1.28448058219051e-33 |
| PLEK | 5.40442729237659e-38 | 0.123747041154092 | 0.861 | 0.767 | 1.33283985884591e-33 |
| GPAT3 | 5.56934102537769e-38 | 0.420543733115151 | 0.139 | 0.089 | 1.37351088367864e-33 |
| SRA1 | 6.54367227314787e-38 | 0.228416511165523 | 0.265 | 0.195 | 1.61380045600373e-33 |
| MRVI1 | 8.24561489200032e-38 | 0.441893896616605 | 0.11 | 0.066 | 2.03353354466512e-33 |
| RASGRP2 | 1.06431611536963e-37 | 0.281617793210005 | 0.274 | 0.204 | 2.62481640372457e-33 |
| AC116353.5 | 1.14598954650017e-37 | 0.378914152783547 | 0.164 | 0.109 | 2.82623941957873e-33 |
| PDE4B | 1.27433144374845e-37 | 0.162928100611171 | 0.301 | 0.223 | 3.14275620657243e-33 |
| GABARAPL1 | 1.37500010971945e-37 | 0.272695911121396 | 0.248 | 0.181 | 3.3910252705901e-33 |
| MAST3 | 1.56601614677164e-37 | 0.24850177294512 | 0.179 | 0.122 | 3.86210902116823e-33 |
| AL035661.1 | 1.67095015132389e-37 | 0.825321878785515 | 0.068 | 0.035 | 4.12089726319499e-33 |
| FERMT3 | 1.95686403647403e-37 | 0.223864995549654 | 0.414 | 0.334 | 4.82601808675224e-33 |
| DENND1C | 2.0300868474637e-37 | 0.329418605684002 | 0.169 | 0.114 | 5.00660018321498e-33 |
| IER2 | 3.35636858031785e-37 | 0.14399599722486 | 0.847 | 0.769 | 8.27747619277988e-33 |
| MYL12A | 3.73229928883639e-37 | 0.14917688840539 | 0.797 | 0.689 | 9.20459650612831e-33 |
| TOR1B | 4.11096483895847e-37 | 0.331713013990115 | 0.176 | 0.121 | 1.01384614858394e-32 |
| OSBPL2 | 6.69724449006262e-37 | 0.236767384985203 | 0.234 | 0.169 | 1.65167443613924e-32 |
| SIRPB2 | 7.28874850470583e-37 | 0.612558242183261 | 0.094 | 0.055 | 1.79755115623055e-32 |
| LITAF | 7.53298589728785e-37 | 0.109427472190792 | 0.888 | 0.789 | 1.85778498198913e-32 |
| ZNF516 | 7.89402660619539e-37 | 0.348999446800938 | 0.171 | 0.117 | 1.94682484161991e-32 |
| DIAPH1 | 1.10623350666231e-36 | 0.290629815334607 | 0.3 | 0.231 | 2.72819307413059e-32 |
| RGL2 | 1.26357833964444e-36 | 0.322010642233429 | 0.178 | 0.122 | 3.11623690123111e-32 |
| SNX18 | 1.28068156553226e-36 | 0.289991796813849 | 0.236 | 0.172 | 3.15841687691566e-32 |
| MS4A6A | 1.31910859192074e-36 | 0.227066309087014 | 0.344 | 0.266 | 3.25318560939493e-32 |
| PPP1R3B | 1.87176747551849e-36 | 0.29814926683791 | 0.197 | 0.137 | 4.6161529481237e-32 |

| Gene | P_val | Avg_log2FC | Pct.1 | Pct.2 | P_val_adj |
|---|---|---|---|---|---|
| CAPN1 | 1.97895705703921e-36 | 0.524862845072924 | 0.145 | 0.097 | 4.88050389407009e-32 |
| CEBPA | 2.12763268976497e-36 | 0.823315046411597 | 0.085 | 0.049 | 5.24716773949837e-32 |
| PTK2B | 2.18634528049965e-36 | 0.216910916293456 | 0.334 | 0.258 | 5.39196473076825e-32 |
| TNFSF8 | 2.23454690242839e-36 | 0.456182676412256 | 0.097 | 0.057 | 5.51083957076889e-32 |
| CKLF | 2.67574614507006e-36 | 0.116189724697088 | 0.624 | 0.513 | 6.59892514297177e-32 |
| MYO5A | 2.81323962605188e-36 | 0.323700396367738 | 0.176 | 0.121 | 6.93801156576916e-32 |
| VASP | 2.97479788216984e-36 | 0.144829454576734 | 0.671 | 0.569 | 7.33644653700725e-32 |
| CAMKK2 | 3.22889469632104e-36 | 0.394694003738579 | 0.175 | 0.121 | 7.96310010006694e-32 |
| KDM4B | 3.72409788772454e-36 | 0.272630957115784 | 0.176 | 0.12 | 9.18437021070625e-32 |
| PRKACA | 4.72502128809223e-36 | 0.243622353485169 | 0.214 | 0.153 | 1.16528475006931e-31 |
| HSF1 | 6.15927248617885e-36 | 0.437455786959627 | 0.121 | 0.076 | 1.51899978054143e-31 |
| ARHGDIA | 9.27095835265894e-36 | 0.301858543545488 | 0.337 | 0.266 | 2.28640374893275e-31 |
| PLBD1 | 1.11324516731385e-35 | 0.11758451448391 | 0.397 | 0.312 | 2.74548523162941e-31 |
| VNN1 | 1.35426031673085e-35 | 0.252096006264869 | 0.245 | 0.18 | 3.33987679312163e-31 |
| SH3BGRL3 | 1.43345771996023e-35 | 0.12814551498097 | 0.893 | 0.813 | 3.53519342896592e-31 |
| MAP3K3 | 1.65546673870265e-35 | 0.157739455937471 | 0.326 | 0.248 | 4.08271207098848e-31 |
| LRRC25 | 1.76009149946483e-35 | 0.215038953262199 | 0.386 | 0.303 | 4.34073765598017e-31 |
| MTF1 | 1.92526641913616e-35 | 0.283379037563837 | 0.202 | 0.144 | 4.7480920428736e-31 |
| OS9 | 2.21372974308129e-35 | 0.212467343963932 | 0.438 | 0.354 | 5.45950029238707e-31 |
| SKAP2 | 2.96536924001927e-35 | 0.164009618240335 | 0.518 | 0.429 | 7.31319361973553e-31 |
| IL1RN | 3.14208536141993e-35 | 0.154396217737751 | 0.4 | 0.315 | 7.74901091833383e-31 |
| GLIPR1 | 3.58639074072291e-35 | 0.205665362877869 | 0.268 | 0.198 | 8.84475684477084e-31 |
| XKR8 | 3.93804745450841e-35 | 0.318414744459319 | 0.14 | 0.092 | 9.71201263230863e-31 |
| UPB1 | 4.81416690396916e-35 | 0.845816041761226 | 0.057 | 0.028 | 1.18726984185687e-30 |
| RASAL3 | 4.88032618140427e-35 | 0.355851356438986 | 0.149 | 0.1 | 1.20358604285792e-30 |
| NFAM1 | 5.85079072448906e-35 | 0.227595159877373 | 0.247 | 0.181 | 1.44292200847349e-30 |
| OAS3 | 6.5305249997983e-35 | 0.288615855957652 | 0.273 | 0.206 | 1.61055807545026e-30 |
| BRI3 | 6.63032767978821e-35 | 0.12131461205946 | 0.841 | 0.745 | 1.63517141238937e-30 |
| MOB3A | 7.39601709688889e-35 | 0.114671446083922 | 0.367 | 0.283 | 1.82400573643474e-30 |
| COL18A1 | 7.73520067455468e-35 | 0.377765333073835 | 0.129 | 0.083 | 1.90765519035867e-30 |
| PHF1 | 1.17750610027644e-34 | 0.283269802726982 | 0.19 | 0.133 | 2.90396554450176e-30 |
| TBC1D1 | 1.42651765136625e-34 | 0.225931642104781 | 0.25 | 0.185 | 3.51807783179945e-30 |

| Gene | P_val | Avg_log2FC | Pct. 1 | Pct. 2 | P_val_adj |
|---|---|---|---|---|---|
| EXTL3 | 1.88606476488967e-34 | 0.414008293568029 | 0.127 | 0.082 | 4.65141292317091e-30 |
| NPEPL1 | 2.12288097463251e-34 | 0.363755015276324 | 0.121 | 0.077 | 5.23544905963871e-30 |
| PXK | 2.16346538938388e-34 | 0.35634440231783 | 0.157 | 0.106 | 5.33553834329853e-30 |
| RPS6KA1 | 2.677139017079e-34 | 0.177126150505925 | 0.329 | 0.253 | 6.60236024392024e-30 |
| STK17A | 2.77657719965125e-34 | 0.207096319264007 | 0.276 | 0.207 | 6.84759468977992e-30 |
| HLA-B | 2.8451879924937e-34 | 0.174389424076448 | 0.979 | 0.959 | 7.01680262708796e-30 |
| IFNGR2 | 3.09044403077694e-34 | 0.129740603375633 | 0.475 | 0.381 | 7.6216530687021e-30 |
| COA1 | 3.11999569320962e-34 | 0.545806961571817 | 0.101 | 0.062 | 7.69453337859355e-30 |
| CANT1 | 4.23977529223743e-34 | 0.380524718730775 | 0.135 | 0.088 | 1.04561338257159e-29 |
| CYBA | 4.41411599515852e-34 | 0.140556917727571 | 0.86 | 0.775 | 1.08860928672599e-29 |
| VPS37B | 4.98853443191845e-34 | 0.234367529590879 | 0.187 | 0.131 | 1.23027236159973e-29 |
| SYNE2 | 5.86352608487588e-34 | 0.160550955428426 | 0.274 | 0.206 | 1.44606280305209e-29 |
| MANSC1 | 6.38531979878773e-34 | 0.40514522161763 | 0.144 | 0.096 | 1.57474756877703e-29 |
| RASSF2 | 7.84189479891266e-34 | 0.146376706747401 | 0.446 | 0.357 | 1.93396809530784e-29 |
| NR1H2 | 8.92247307833152e-34 | 0.311159528540268 | 0.192 | 0.137 | 2.20046031057812e-29 |
| SDCBP | 1.06725541584853e-33 | 0.108919418439744 | 0.788 | 0.676 | 2.63206530656563e-29 |
| SH2D3C | 1.08716447676062e-33 | 0.264094234718043 | 0.196 | 0.138 | 2.68116503258705e-29 |
| ATG7 | 1.14303157289605e-33 | 0.220339869242291 | 0.169 | 0.117 | 2.81894446507623e-29 |
| MYD88 | 1.25701907166575e-33 | 0.201168478357074 | 0.359 | 0.281 | 3.10006043454208e-29 |
| LOXHD1 | 1.45946658497569e-33 | 0.625111848446247 | 0.067 | 0.036 | 3.59933649186704e-29 |
| EMB | 1.83792462522674e-33 | 0.163488945490885 | 0.409 | 0.325 | 4.53268971073418e-29 |
| ABCA2 | 2.47040283354058e-33 | 0.51925406155244 | 0.087 | 0.051 | 6.09250746807778e-29 |
| AGTRAP | 2.49521533247097e-33 | 0.213185182559217 | 0.47 | 0.389 | 6.15370005293989e-29 |
| GLT1D1 | 3.91490128897432e-33 | 0.106135109788686 | 0.442 | 0.351 | 9.65492955886847e-29 |
| TMUB2 | 5.91380518788339e-33 | 0.272334608900381 | 0.209 | 0.151 | 1.4584626354358e-28 |
| SCO2 | 8.05401564552916e-33 | 0.20245559023957 | 0.334 | 0.259 | 1.9862813385004e-28 |
| RIPK3 | 9.76818906696733e-33 | 0.897163020273271 | 0.051 | 0.024 | 2.40903078769548e-28 |
| TXN | 1.10851457134503e-32 | 0.148619686744808 | 0.618 | 0.518 | 2.73381863585112e-28 |
| WDFY4 | 1.40841744256148e-32 | 0.39552832117001 | 0.113 | 0.072 | 3.47343909684512e-28 |
| STK40 | 1.46844668633077e-32 | 0.128956627849521 | 0.281 | 0.21 | 3.62148321782896e-28 |
| ZBP1 | 1.48632435812993e-32 | 0.184893430413839 | 0.342 | 0.267 | 3.66557313202003e-28 |
| ZFP36 | 1.63884801200022e-32 | 0.131059458707042 | 0.743 | 0.642 | 4.04172696719494e-28 |

| Gene | P_val | Avg_log2FC | Pct.1 | Pct.2 | P_val_adj |
|---|---|---|---|---|---|
| PRKCD | 1.69643821271044e-32 | 0.174670157004077 | 0.312 | 0.24 | 4.18375592018649e-28 |
| SH3BP5 | 2.67107383593591e-32 | 0.294426093933423 | 0.191 | 0.137 | 6.58740229418515e-28 |
| IFIT1 | 3.21774848882222e-32 | 0.177964060265581 | 0.402 | 0.322 | 7.93561132313337e-28 |
| CORO7 | 3.78281988610695e-32 | 0.391168856895581 | 0.148 | 0.101 | 9.32919040311695e-28 |
| PPP1R12C | 4.66051181536233e-32 | 0.319475969948912 | 0.141 | 0.095 | 1.14937542390466e-27 |
| IFI44 | 4.66432184879935e-32 | 0.158675477736048 | 0.483 | 0.396 | 1.15031505435089e-27 |
| TRIOBP | 6.96554132513752e-32 | 0.3647657725347 | 0.16 | 0.111 | 1.71784180160541e-27 |
| PLEKHG3 | 7.71143890722941e-32 | 0.337563082797282 | 0.146 | 0.099 | 1.90179506330092e-27 |
| TMX4 | 8.26050271339375e-32 | 0.255055869195473 | 0.239 | 0.177 | 2.03720517917717e-27 |
| CSNK1D | 1.01256046984623e-31 | 0.191459745968421 | 0.283 | 0.216 | 2.49717663073478e-27 |
| CREBBP | 1.02077952428963e-31 | 0.143201850849967 | 0.348 | 0.271 | 2.51744646280308e-27 |
| RRP12 | 1.06412201823756e-31 | 0.272769504296616 | 0.209 | 0.152 | 2.62433772137746e-27 |
| NQO2 | 1.31464732120379e-31 | 0.28644923785053 | 0.226 | 0.168 | 3.2421832235528e-27 |
| HSH2D | 1.3582204401922e-31 | 0.136980616419308 | 0.423 | 0.341 | 3.349643249602e-27 |
| ITGB2-AS1 | 1.86685708975467e-31 | 0.256917838085724 | 0.193 | 0.138 | 4.60404295475298e-27 |
| ENTPD4 | 2.27100077681138e-31 | 0.344537211089123 | 0.12 | 0.078 | 5.60074211577224e-27 |
| PRDX5 | 2.3570169059563e-31 | 0.228161013879881 | 0.419 | 0.342 | 5.81287509346942e-27 |
| VPS9D1 | 3.10318187159334e-31 | 0.241747380909922 | 0.183 | 0.13 | 7.65306713172349e-27 |
| RASGRP4 | 3.11353486351894e-31 | 0.21561454443384 | 0.272 | 0.206 | 7.6785996804104e-27 |
| CSGALNACT2 | 3.66531739455778e-31 | 0.124985195653992 | 0.438 | 0.356 | 9.0394057584584e-27 |
| MTX1 | 3.97971518604752e-31 | 0.395393410223874 | 0.16 | 0.112 | 9.81477359183038e-27 |
| PPP1R12B | 4.29478029196705e-31 | 0.325619641994104 | 0.114 | 0.074 | 1.05917871560491e-26 |
| LAMTOR5 | 4.74198482930604e-31 | 0.218191114098186 | 0.323 | 0.253 | 1.16946829860346e-26 |
| SH3GLB1 | 5.25726317195516e-31 | 0.151133891670398 | 0.608 | 0.511 | 1.29654624346758e-26 |
| TMCC3 | 6.59943580097129e-31 | 0.156220172152307 | 0.192 | 0.136 | 1.62755285723554e-26 |
| HCK | 7.82067283464246e-31 | 0.111576776384311 | 0.751 | 0.653 | 1.92873433447952e-26 |
| DPEP2 | 8.86624995713907e-31 | 0.13314144677222 | 0.229 | 0.168 | 2.18659456442964e-26 |
| PTP4A3 | 9.67724037286372e-31 | 0.255037267227538 | 0.135 | 0.09 | 2.38660102075565e-26 |
| PLXNC1 | 9.99699272027915e-31 | 0.102594565065198 | 0.429 | 0.344 | 2.46545834467524e-26 |
| GTPBP1 | 1.39318752880739e-30 | 0.143606330527991 | 0.191 | 0.137 | 3.4358790835448e-26 |
| TMEM120A | 1.42866240894288e-30 | 0.303327236608141 | 0.164 | 0.115 | 3.52336723293493e-26 |
| HLA-F | 1.49605833208285e-30 | 0.163653937184019 | 0.469 | 0.388 | 3.68957905858272e-26 |

| Gene | P_val | Avg_log2FC | Pct.1 | Pct.2 | P_val_adj |
|---|---|---|---|---|---|
| AC108134.3 | 1.97247094773336e-30 | 0.541263881304046 | 0.08 | 0.047 | 4.86450785130001e-26 |
| OSTF1 | 2.62453629374394e-30 | 0.181543202423589 | 0.35 | 0.276 | 6.4726314076313e-26 |
| NDST1 | 3.33490192250423e-30 | 0.499281981939136 | 0.082 | 0.049 | 8.22453512127992e-26 |
| TECPR1 | 3.35511038009532e-30 | 0.373207702429994 | 0.112 | 0.072 | 8.27437321939109e-26 |
| ARHGAP27P1-BPTFP1-KPNA2P3 | 3.78192115579754e-30 | 0.311462322784112 | 0.167 | 0.118 | 9.3269739544279e-26 |
| LRRC4 | 5.55615527735087e-30 | 0.39991198547381 | 0.106 | 0.067 | 1.37025901450027e-25 |
| STX4 | 9.51519924562654e-30 | 0.29330379811161 | 0.15 | 0.104 | 2.34663843795642e-25 |
| DEDD2 | 1.03349673163918e-29 | 0.231839705464309 | 0.244 | 0.183 | 2.54880963956855e-25 |
| DHRS13 | 1.03887213200969e-29 | 0.659278761804744 | 0.069 | 0.039 | 2.5620664519623e-25 |
| NARF | 1.20395919040211e-29 | 0.188102712209749 | 0.248 | 0.187 | 2.96920415536969e-25 |
| DGKD | 2.74531506578079e-29 | 0.34804202063259 | 0.15 | 0.106 | 6.77049601522858e-25 |
| PPP1R18 | 3.4956723651327e-29 | 0.128689568548746 | 0.548 | 0.457 | 8.62102718689026e-25 |
| ACOX1 | 5.39191201387173e-29 | 0.234673764532479 | 0.182 | 0.131 | 1.32975334086105e-24 |
| PDLIM2 | 6.01897685354164e-29 | 0.327574405012579 | 0.15 | 0.105 | 1.48440007162044e-24 |
| AL031777.2 | 6.98688919843807e-29 | 0.436827749306823 | 0.111 | 0.073 | 1.7231066141188e-24 |
| MAU2 | 9.23281317479674e-29 | 0.300302332470062 | 0.156 | 0.109 | 2.27699638516837e-24 |
| AL034397.3 | 1.04476833861466e-28 | 0.247341603285663 | 0.331 | 0.265 | 2.57660767669147e-24 |
| ARHGAP27 | 1.18702105972941e-28 | 0.177949208976327 | 0.216 | 0.16 | 2.92743133750468e-24 |
| RALY | 1.28793223330475e-28 | 0.153072541923206 | 0.279 | 0.215 | 3.17629847377618e-24 |
| RAF1 | 1.33838174678679e-28 | 0.126799810111252 | 0.334 | 0.262 | 3.30071706392557e-24 |
| CCM2 | 1.9263758940987e-28 | 0.261161154123242 | 0.168 | 0.12 | 4.75082823002621e-24 |
| RIPK1 | 2.7501817164469e-28 | 0.231240801479333 | 0.167 | 0.118 | 6.78249814910133e-24 |
| SIRPB1 | 2.87513507619587e-28 | 0.200195461239732 | 0.306 | 0.24 | 7.09065812491427e-24 |
| ADAP1 | 3.34464422077258e-28 | 0.47238418458897 | 0.083 | 0.051 | 8.24856157726933e-24 |
| ADGRE3 | 3.43966201263893e-28 | 0.199844263677718 | 0.169 | 0.119 | 8.48289445557012e-24 |
| SEMA4A | 3.46431270540323e-28 | 0.17490204065658 | 0.272 | 0.21 | 8.54368799406545e-24 |
| CASP4 | 3.62517098168708e-28 | 0.121666288710676 | 0.462 | 0.377 | 8.94039667503669e-24 |
| PDXK | 4.51324217760595e-28 | 0.379600678521622 | 0.153 | 0.109 | 1.11305578584118e-23 |
| ITGAL | 4.77362906149207e-28 | 0.130769097753641 | 0.266 | 0.204 | 1.17727239914518e-23 |
| MAF1 | 8.43091202589677e-28 | 0.408422449934655 | 0.159 | 0.114 | 2.07923152382666e-23 |
| C1GALT1 | 8.98330971670465e-28 | 0.298517141334232 | 0.174 | 0.126 | 2.2154638423337e-23 |
| CBX4 | 9.28088166557679e-28 | 0.322428553935998 | 0.166 | 0.12 | 2.28885103636455e-23 |

| Gene | P_val | Avg_log2FC | Pct.1 | Pct.2 | P_val_adj |
|---|---|---|---|---|---|
| GTPBP2 | 9.40833605295717e-28 | 0.305254607077446 | 0.143 | 0.099 | 2.3202838373803e-23 |
| TNFSF13B | 1.25356684231882e-27 | 0.164119185031123 | 0.6 | 0.513 | 3.09154654652666e-23 |
| ANXA3 | 2.12466870921002e-27 | 0.114955158756642 | 0.49 | 0.402 | 5.23985797065376e-23 |
| MIR646HG | 2.40655276991282e-27 | 0.288585070936801 | 0.082 | 0.05 | 5.935040441159e-23 |
| IQGAP1 | 2.71789180457343e-27 | 0.112758421629992 | 0.733 | 0.643 | 6.702864768439e-23 |
| GPR132 | 2.8537651721438e-27 | 0.337399052530341 | 0.116 | 0.077 | 7.03795566754104e-23 |
| PLK3 | 3.26189387840214e-27 | 0.232573826608758 | 0.205 | 0.152 | 8.04448268291535e-23 |
| TNK2 | 3.46054283571001e-27 | 0.345627424864765 | 0.127 | 0.087 | 8.53439074142802e-23 |
| MLF2 | 4.38139947451693e-27 | 0.185124656419999 | 0.293 | 0.229 | 1.08054073840537e-22 |
| TRAF3IP3 | 4.41052742776604e-27 | 0.210099973784261 | 0.215 | 0.161 | 1.08772427423566e-22 |
| JUND | 5.66511588253984e-27 | 0.180141120380853 | 0.49 | 0.416 | 1.39713087895197e-22 |
| RABAC1 | 6.08978191215434e-27 | 0.246652789159399 | 0.248 | 0.191 | 1.5018620151755e-22 |
| MAPK8IP3 | 6.13846652563998e-27 | 0.235291931177353 | 0.147 | 0.103 | 1.51386861455333e-22 |
| TLR5 | 6.33545753686756e-27 | 0.464025291954036 | 0.106 | 0.069 | 1.56245053774228e-22 |
| JAK1 | 6.56105005147752e-27 | 0.105136165918304 | 0.469 | 0.389 | 1.61808616369539e-22 |
| MOSPD2 | 6.56176836062982e-27 | 0.259912098022309 | 0.172 | 0.125 | 1.61826331309853e-22 |
| ZDHHC19 | 9.1360034199661e-27 | 0.350044621638751 | 0.229 | 0.178 | 2.25312116343204e-22 |
| LINC02863 | 1.01318439883136e-26 | 0.6664123072665 | 0.053 | 0.028 | 2.49871536439789e-22 |
| AKT2 | 1.1279341772803e-26 | 0.594859414508993 | 0.076 | 0.046 | 2.78171126800868e-22 |
| IFI35 | 1.27376380386883e-26 | 0.172946148774154 | 0.331 | 0.265 | 3.14135629310131e-22 |
| AKT1 | 1.4455672900145e-26 | 0.435041885065022 | 0.077 | 0.047 | 3.56505805063375e-22 |
| GLIPR2 | 1.94571245223535e-26 | 0.10388693697926 | 0.515 | 0.429 | 4.79851604970281e-22 |
| IL6R | 2.00836233042507e-26 | 0.137915879199816 | 0.257 | 0.197 | 4.95302317929431e-22 |
| FRAT1 | 2.57785863431796e-26 | 0.414279726706543 | 0.111 | 0.074 | 6.35751496395494e-22 |
| AKAP17A | 2.66773689718886e-26 | 0.197470283363646 | 0.241 | 0.185 | 6.57917273584717e-22 |
| LGALS9 | 3.38587534056044e-26 | 0.128247072139844 | 0.294 | 0.229 | 8.35024576489017e-22 |
| MTHFS | 3.49151939967315e-26 | 0.28828108514603 | 0.168 | 0.121 | 8.61078514347393e-22 |
| SMIM25 | 5.57327036147616e-26 | 0.11192681953316 | 0.381 | 0.305 | 1.37447993654725e-21 |
| PHOSPHO1 | 5.61083257288048e-26 | 0.232476316288126 | 0.083 | 0.051 | 1.38374352912378e-21 |
| CD58 | 5.93516015050547e-26 | 0.138927633205657 | 0.295 | 0.232 | 1.46372919631766e-21 |
| HDAC7 | 6.43270324327111e-26 | 0.283201167049739 | 0.141 | 0.099 | 1.58643327385552e-21 |
| TIMP2 | 7.18788193542481e-26 | 0.145504830781077 | 0.378 | 0.307 | 1.77267544291447e-21 |

| Gene | P_val | Avg_log2FC | Pct.1 | Pct.2 | P_val_adj |
|---|---|---|---|---|---|
| MSRB1 | 7.23790102494481e-26 | 0.105630216035237 | 0.685 | 0.59 | 1.78501115077189e-21 |
| OSER1 | 7.78427317724536e-26 | 0.140891981912562 | 0.263 | 0.203 | 1.91975745097225e-21 |
| RALB | 8.51120810548221e-26 | 0.139713957311151 | 0.406 | 0.331 | 2.09903414297402e-21 |
| GAA | 8.54073624026619e-26 | 0.267038222643114 | 0.166 | 0.12 | 2.10631637157445e-21 |
| MXD3 | 9.69033761392649e-26 | 0.304808298250253 | 0.136 | 0.096 | 2.38983106234655e-21 |
| RARA | 9.98044914159703e-26 | 0.113439276048237 | 0.332 | 0.262 | 2.46137836730066e-21 |
| RSAD2 | 1.12117374302521e-25 | 0.102537333538542 | 0.471 | 0.395 | 2.76503868504878e-21 |
| SLC49A4 | 1.13140107716339e-25 | 0.140672393781601 | 0.162 | 0.116 | 2.79026133650035e-21 |
| TADA3 | 1.19536733512306e-25 | 0.266963977177515 | 0.163 | 0.118 | 2.94801492188048e-21 |
| HLX | 1.23878574623983e-25 | 0.336554373047814 | 0.172 | 0.127 | 3.05509340737666e-21 |
| NFE4 | 1.33558541566825e-25 | 0.281101800408287 | 0.126 | 0.086 | 3.29382075212104e-21 |
| ACSL4 | 1.42124207720849e-25 | 0.128486842047076 | 0.297 | 0.233 | 3.50506721081159e-21 |
| AC104530.1 | 1.43018887478653e-25 | 0.587180458414745 | 0.056 | 0.031 | 3.52713180299853e-21 |
| TMEM127 | 2.048778856596e-25 | 0.149747224969283 | 0.239 | 0.182 | 5.05269841613706e-21 |
| COPE | 2.65977490604451e-25 | 0.227167817071876 | 0.268 | 0.212 | 6.55953687328698e-21 |
| SLC26A8 | 2.83758578117483e-25 | 0.409646829846447 | 0.105 | 0.07 | 6.99805405353336e-21 |
| SNRK | 2.9142177768752e-25 | 0.112350362303902 | 0.247 | 0.189 | 7.18704388132962e-21 |
| NDEL1 | 3.00442854743633e-25 | 0.141709372808354 | 0.199 | 0.147 | 7.40952168368748e-21 |
| SLC16A3 | 3.43016259633494e-25 | 0.119205759212453 | 0.354 | 0.286 | 8.45946699508122e-21 |
| ADM | 3.76585282292256e-25 | 0.196434014568079 | 0.334 | 0.271 | 9.28734623189162e-21 |
| DNAJB1 | 4.03204198675755e-25 | 0.20189446937722 | 0.247 | 0.191 | 9.94382194774148e-21 |
| KIF21B | 4.14438133882848e-25 | 0.419713210907003 | 0.095 | 0.062 | 1.02208732578188e-20 |
| FGD4 | 4.22010993988888e-25 | 0.146576789778346 | 0.203 | 0.152 | 1.0407635133754e-20 |
| C1RL | 4.41815567498754e-25 | 0.262086759699388 | 0.196 | 0.147 | 1.08960555256543e-20 |
| TLR1 | 4.88574166380086e-25 | 0.108492899340779 | 0.374 | 0.299 | 1.20492160912657e-20 |
| DGAT1 | 4.90917687598321e-25 | 0.32062952247136 | 0.125 | 0.087 | 1.21070120115498e-20 |
| C11orf21 | 5.86299869704374e-25 | 0.434410341830346 | 0.101 | 0.067 | 1.44593273866493e-20 |
| FAM53C | 5.90479658915271e-25 | 0.193228869812699 | 0.195 | 0.145 | 1.45624093481684e-20 |
| SPSB3 | 6.60376847819968e-25 | 0.250485850368723 | 0.176 | 0.13 | 1.62862138209361e-20 |
| CLK3 | 7.038340304612e-25 | 0.279762077509059 | 0.136 | 0.096 | 1.73579548592341e-20 |
| TMEM259 | 7.68161557008149e-25 | 0.200501402225589 | 0.173 | 0.127 | 1.8944400318935e-20 |
| OSCAR | 9.14181289815137e-25 | 0.187332506253356 | 0.38 | 0.315 | 2.25455389694209e-20 |

| Gene | P_val | Avg_log2FC | Pct. 1 | Pct. 2 | P_val_adj |
|---|---|---|---|---|---|
| ADARB2 | 9.76419719445564e-25 | 0.604458168706258 | 0.082 | 0.052 | 2.40804631209665e-20 |
| UBE2M | 1.1382962115013e-24 | 0.329912523179608 | 0.127 | 0.088 | 2.8072661168045e-20 |
| SERPINB1 | 1.14858441520805e-24 | 0.154848562253165 | 0.699 | 0.606 | 2.8326388847861e-20 |
| FGR | 1.15000906195178e-24 | 0.11733409972557 | 0.686 | 0.601 | 2.83615234858547e-20 |
| UBE2S | 1.32462760825603e-24 | 0.257201772830657 | 0.186 | 0.139 | 3.26679660748102e-20 |
| RIT1 | 1.34443069234109e-24 | 0.162699394459289 | 0.227 | 0.174 | 3.3156349734516e-20 |
| ELL | 1.85014115265889e-24 | 0.154563248791856 | 0.164 | 0.119 | 4.56281811068736e-20 |
| CNPY3 | 1.87535527568566e-24 | 0.153030905482158 | 0.336 | 0.272 | 4.62500118089597e-20 |
| CSK | 2.56369964209841e-24 | 0.102324961006335 | 0.29 | 0.226 | 6.32259605734309e-20 |
| AC023157.3 | 2.76848764791718e-24 | 0.298727418431628 | 0.095 | 0.062 | 6.82764423729334e-20 |
| UNC119 | 3.65154228200045e-24 | 0.251172109751889 | 0.156 | 0.113 | 9.00543357586951e-20 |
| OPLAH | 3.96601227866646e-24 | 0.6635890228324 | 0.059 | 0.034 | 9.78097948164723e-20 |
| ORM1 | 4.86307709585695e-24 | 0.358754297692066 | 0.113 | 0.078 | 1.19933207338024e-19 |
| PPFIA1 | 6.9242429362316e-24 | 0.257607475550914 | 0.173 | 0.128 | 1.70765679293344e-19 |
| SLC40A1 | 8.01124009925396e-24 | 0.278424554200288 | 0.146 | 0.105 | 1.97573203327801e-19 |
| DGCR2 | 8.94633638425703e-24 | 0.228525248944583 | 0.109 | 0.074 | 2.20634547908547e-19 |
| ROPN1L | 1.00377216778228e-23 | 0.402616227304869 | 0.104 | 0.07 | 2.47550292018467e-19 |
| YWHAB | 1.01507808191778e-23 | 0.11346785233668 | 0.579 | 0.495 | 2.50338556562562e-19 |
| F8A1 | 1.14357184238642e-23 | 0.444503556169573 | 0.09 | 0.058 | 2.82027687769339e-19 |
| ZNF106 | 1.21429709377735e-23 | 0.204838635144019 | 0.264 | 0.21 | 2.9946994926737e-19 |
| HSD17B11 | 1.33309011379505e-23 | 0.101594826796853 | 0.446 | 0.367 | 3.28766683864135e-19 |
| PTPN6 | 1.53031677683976e-23 | 0.10442796927318 | 0.463 | 0.386 | 3.77406723504221e-19 |
| RCN3 | 1.80705663891672e-23 | 0.425021715580064 | 0.051 | 0.028 | 4.45656308289641e-19 |
| CCNI | 1.85325246089801e-23 | 0.119661167615638 | 0.483 | 0.407 | 4.57049121906667e-19 |
| JPT1 | 1.97437585405494e-23 | 0.119836967661533 | 0.406 | 0.334 | 4.86920573127029e-19 |
| CARD16 | 3.59292787282652e-23 | 0.155696430055635 | 0.565 | 0.483 | 8.86087871996476e-19 |
| ACOT9 | 5.83444439830916e-23 | 0.127149764179336 | 0.224 | 0.172 | 1.438890677511e-18 |
| C4orf3 | 6.42362435697449e-23 | 0.132687666607391 | 0.542 | 0.462 | 1.58419423891705e-18 |
| ARL8A | 6.47034199711099e-23 | 0.153464733429539 | 0.257 | 0.202 | 1.59571574332751e-18 |
| KCNQ1 | 7.01243328266962e-23 | 0.434668524895721 | 0.086 | 0.056 | 1.72940629617198e-18 |
| OGFRL1 | 7.73888037907413e-23 | 0.125892570258605 | 0.44 | 0.369 | 1.90856267908726e-18 |
| G6PD | 8.02324286082093e-23 | 0.218330074769241 | 0.274 | 0.22 | 1.97869215433566e-18 |

| Gene | P_val | Avg_log2FC | Pct.1 | Pct.2 | P_val_adj |
|---|---|---|---|---|---|
| TANC2 | 8.1577278623901e-23 | 0.313593891071629 | 0.078 | 0.049 | 2.01185884542265e-18 |
| TSSC4 | 8.41384604999787e-23 | 0.352640400978819 | 0.115 | 0.08 | 2.07502271285048e-18 |
| CHMP4B | 8.4893072099384e-23 | 0.122962090270316 | 0.361 | 0.294 | 2.09363294411501e-18 |
| ADD3 | 8.54709237578771e-23 | 0.127489355587458 | 0.368 | 0.301 | 2.10788392171677e-18 |
| SH2B2 | 8.77664861035838e-23 | 0.252627211873126 | 0.131 | 0.093 | 2.16449708028658e-18 |
| C5AR2 | 9.34158394623888e-23 | 0.184642263416659 | 0.146 | 0.105 | 2.30382143282143e-18 |
| RAB32 | 9.45345125814731e-23 | 0.224461950621245 | 0.264 | 0.211 | 2.33141014928429e-18 |
| GJB6 | 1.11150662863401e-22 | 0.692682833792942 | 0.051 | 0.029 | 2.7411976475372e-18 |
| B3GNT8 | 2.10836846556817e-22 | 0.402905029174451 | 0.098 | 0.066 | 5.19965830978421e-18 |
| MPP1 | 2.19201966833563e-22 | 0.132095668532075 | 0.287 | 0.228 | 5.40595890604933e-18 |
| FOSB | 2.36403357801302e-22 | 0.41242499536473 | 0.142 | 0.104 | 5.83017961009571e-18 |
| SYTL3 | 2.42210592382218e-22 | 0.200512159956535 | 0.14 | 0.101 | 5.97339762933026e-18 |
| ATP6V0B | 2.55004480493906e-22 | 0.116572306298842 | 0.632 | 0.553 | 6.28892049794071e-18 |
| PPP4R1 | 2.61192441747727e-22 | 0.135097345422118 | 0.193 | 0.146 | 6.44152799838245e-18 |
| CSAD | 2.61993205641899e-22 | 0.190066787760715 | 0.2 | 0.153 | 6.46127643754052e-18 |
| SYTL1 | 4.00410952563859e-22 | 0.347695251031708 | 0.133 | 0.096 | 9.87493491212989e-18 |
| RNF167 | 4.24341407290341e-22 | 0.214199061361926 | 0.171 | 0.128 | 1.04651077865944e-17 |
| AC136475.9 | 4.24799529610177e-22 | 0.492414631195849 | 0.061 | 0.037 | 1.04764059992462e-17 |
| AKIRIN2 | 4.36115020132617e-22 | 0.121318776604855 | 0.367 | 0.301 | 1.07554686265106e-17 |
| ANKRD55 | 4.78776046991292e-22 | 0.667534866197695 | 0.052 | 0.029 | 1.18075748708993e-17 |
| PLEKHM1 | 4.85430283031845e-22 | 0.180867875206456 | 0.199 | 0.152 | 1.19716816401314e-17 |
| ST3GAL4 | 4.91335895380713e-22 | 0.324263463049683 | 0.11 | 0.076 | 1.21173258518792e-17 |
| RNF141 | 7.21282399130122e-22 | 0.156739715493163 | 0.221 | 0.171 | 1.77882665273471e-17 |
| TRIM28 | 8.84254218185405e-22 | 0.325257421042301 | 0.122 | 0.087 | 2.18074775288885e-17 |
| DNTTIP1 | 9.04750099597483e-22 | 0.17603655259911 | 0.197 | 0.151 | 2.23129469562731e-17 |
| CCPG1 | 1.01526898012076e-21 | 0.194699209654213 | 0.24 | 0.19 | 2.50385635877383e-17 |
| TGOLN2 | 1.37271970047757e-21 | 0.117525515878534 | 0.351 | 0.289 | 3.38540132531778e-17 |
| KIAA0513 | 1.5129736812332e-21 | 0.24015456530212 | 0.141 | 0.103 | 3.73129569265731e-17 |
| PLEKHO2 | 1.92517552896403e-21 | 0.159500649492307 | 0.171 | 0.127 | 4.74786788953108e-17 |
| CHSY1 | 1.98756478649812e-21 | 0.10750123036869 | 0.224 | 0.174 | 4.90173227646166e-17 |
| TOR1AIP2 | 2.17763510872612e-21 | 0.211939482561953 | 0.164 | 0.123 | 5.37048370514036e-17 |
| MAP1LC3A | 2.64388774799348e-21 | 0.258844037004202 | 0.139 | 0.101 | 6.52035596410152e-17 |

| Gene | P_val | Avg_log2FC | Pct.1 | Pct.2 | P_val_adj |
| --- | --- | --- | --- | --- | --- |
| HSDL2 | 2.66638239692949e-21 | 0.133810079127311 | 0.208 | 0.159 | 6.57583226730751e-17 |
| NT5C3A | 2.97798541508456e-21 | 0.13257003602016 | 0.381 | 0.317 | 7.34430763068155e-17 |
| OAS2 | 2.98586210648384e-21 | 0.212027517637318 | 0.221 | 0.173 | 7.36373312701044e-17 |
| IL16 | 3.92487000803624e-21 | 0.23298386219178 | 0.162 | 0.121 | 9.67951441381898e-17 |
| PKN1 | 4.0767285840727e-21 | 0.159439899462203 | 0.191 | 0.146 | 1.00540280340401e-16 |
| TDP2 | 4.08198226980373e-21 | 0.101766281937878 | 0.28 | 0.223 | 1.006698467379e-16 |
| UBXN2B | 4.45653622941533e-21 | 0.154672372810244 | 0.206 | 0.159 | 1.09907096489841e-16 |
| CNPPD1 | 4.49664616421954e-21 | 0.329379679819605 | 0.12 | 0.085 | 1.10896287701982e-16 |
| MKRN1 | 4.99555189989233e-21 | 0.123855350243137 | 0.245 | 0.193 | 1.23200300955145e-16 |
| ZNF552 | 5.07112110803873e-21 | 0.421819903238889 | 0.074 | 0.047 | 1.25063988766451e-16 |
| AC020916.1 | 5.08922172410871e-21 | 0.272958175174304 | 0.097 | 0.066 | 1.25510386159969e-16 |
| SLC9A1 | 5.66994179323226e-21 | 0.253784520090358 | 0.097 | 0.066 | 1.39832104504694e-16 |
| RBM23 | 5.7055618844114e-21 | 0.106058733424013 | 0.209 | 0.16 | 1.40710567193354e-16 |
| ARSA | 6.12510614993767e-21 | 0.308403113480807 | 0.104 | 0.072 | 1.51057367869763e-16 |
| SOCS3 | 7.1447983216454e-21 | 0.157184202276687 | 0.359 | 0.297 | 1.76205016208419e-16 |
| AC005050.3 | 7.61761086784327e-21 | 0.214904028110779 | 0.151 | 0.111 | 1.87865519222751e-16 |
| GPR108 | 7.65660138781649e-21 | 0.283156741296723 | 0.145 | 0.108 | 1.8882710342633e-16 |
| ST3GAL2 | 9.4819118201065e-21 | 0.190998898799678 | 0.155 | 0.114 | 2.33842909307467e-16 |
| ARHGEF40 | 1.09720716814728e-20 | 0.2309513623968 | 0.097 | 0.066 | 2.70593231808483e-16 |
| MGRN1 | 1.19035151363915e-20 | 0.21056696664402 | 0.149 | 0.11 | 2.93564490293688e-16 |
| DYNLT1 | 1.26401643002303e-20 | 0.133022470687922 | 0.291 | 0.234 | 3.11731731972279e-16 |
| ARHGEF11 | 1.27543291280481e-20 | 0.337423248928083 | 0.087 | 0.058 | 3.14547264955922e-16 |
| KLF3 | 1.66255088511296e-20 | 0.106384855769365 | 0.287 | 0.229 | 4.10018299286557e-16 |
| SDF2 | 1.72149221617144e-20 | 0.196380861958686 | 0.179 | 0.137 | 4.24554410352201e-16 |
| AXIN1 | 1.92401411080603e-20 | 0.454596061381919 | 0.087 | 0.059 | 4.74500360006984e-16 |
| CDKN1B | 2.09766195411557e-20 | 0.147282124796408 | 0.174 | 0.131 | 5.17325391123981e-16 |
| MAP4K2 | 2.37265407827088e-20 | 0.364651318450925 | 0.078 | 0.051 | 5.85143948783166e-16 |
| CEACAM4 | 2.55286965619743e-20 | 0.23237251838125 | 0.162 | 0.122 | 6.29588714611411e-16 |
| AC011472.2 | 2.59352980273649e-20 | 0.156005574259214 | 0.177 | 0.134 | 6.39616319950873e-16 |
| TWF2 | 3.0459146223537e-20 | 0.221806263424909 | 0.206 | 0.162 | 7.51183464164869e-16 |
| FAM193B | 3.70400201695557e-20 | 0.284083104441978 | 0.099 | 0.068 | 9.13480977421582e-16 |
| HK2 | 4.21126557658135e-20 | 0.385461823462321 | 0.077 | 0.05 | 1.03858231649649e-15 |

| Gene | P_val | Avg_log2FC | Pct. 1 | Pct. 2 | P_val_adj |
|---|---|---|---|---|---|
| EVI2A | 4.50546120035322e-20 | 0.114365709973213 | 0.309 | 0.25 | 1.11113684123111e-15 |
| TPST2 | 4.87145519465114e-20 | 0.136302865285556 | 0.215 | 0.168 | 1.20139828010487e-15 |
| MYO1G | 4.97771689379524e-20 | 0.268917972843831 | 0.125 | 0.091 | 1.22760454034778e-15 |
| DEGS1 | 5.22681455983265e-20 | 0.341339031194047 | 0.122 | 0.089 | 1.28903700674593e-15 |
| PITPNM1 | 5.36139966791135e-20 | 0.239850561792153 | 0.094 | 0.064 | 1.3222283861003e-15 |
| TAGAP | 6.53120461295772e-20 | 0.149459893881172 | 0.262 | 0.21 | 1.61072568164763e-15 |
| MPPE1 | 6.59956284781443e-20 | 0.213400366919421 | 0.154 | 0.115 | 1.627584189528e-15 |
| PGM1 | 7.76510776348791e-20 | 0.533168081862224 | 0.076 | 0.05 | 1.91503087663139e-15 |
| CAPG | 7.9318471538922e-20 | 0.265310323026563 | 0.254 | 0.207 | 1.95615214509289e-15 |
| CUEDC1 | 8.30007985255544e-20 | 0.459791081766499 | 0.065 | 0.041 | 2.04696569323722e-15 |
| ETS2 | 8.5958450971136e-20 | 0.124520549963358 | 0.318 | 0.258 | 2.11990731785016e-15 |
| NSFL1C | 8.67459675402838e-20 | 0.256149586259147 | 0.138 | 0.102 | 2.13932905147848e-15 |
| MAP2K2 | 1.28252692620067e-19 | 0.198979001140386 | 0.191 | 0.148 | 3.16296790539608e-15 |
| VCP | 1.4987620119615e-19 | 0.110402643711541 | 0.234 | 0.186 | 3.69624687389945e-15 |
| MMP24OS | 1.57159967090885e-19 | 0.168035204638674 | 0.191 | 0.148 | 3.8758791083954e-15 |
| VPS28 | 1.59720841885878e-19 | 0.211566841510988 | 0.184 | 0.142 | 3.93903540258951e-15 |
| CCDC9 | 1.96905777942747e-19 | 0.283703348357695 | 0.095 | 0.065 | 4.85609029562403e-15 |
| MFN2 | 2.04320519405055e-19 | 0.211728856821212 | 0.141 | 0.105 | 5.03895264956747e-15 |
| PIK3CA | 2.20711647295848e-19 | 0.212365815208501 | 0.108 | 0.076 | 5.4431906456102e-15 |
| GPR84 | 2.35794297690898e-19 | 0.540166793669583 | 0.082 | 0.056 | 5.81515896965293e-15 |
| RFXANK | 2.93372014999429e-19 | 0.295159379299089 | 0.14 | 0.104 | 7.23514063391591e-15 |
| DCTN1 | 2.96048567596776e-19 | 0.255079908002491 | 0.127 | 0.092 | 7.30114977407169e-15 |
| SLCO4C1 | 3.08843184765179e-19 | 0.400457579271928 | 0.084 | 0.057 | 7.61669062267884e-15 |
| TFEB | 4.47465754186654e-19 | 0.153548265309609 | 0.125 | 0.09 | 1.10354004297513e-14 |
| MARK2 | 5.54935433564543e-19 | 0.126004680223203 | 0.205 | 0.16 | 1.36858176625688e-14 |
| NUAK2 | 5.70164537847891e-19 | 0.10742591777481 | 0.19 | 0.146 | 1.40613978324047e-14 |
| SNX20 | 5.90222583066246e-19 | 0.20588552690428 | 0.199 | 0.156 | 1.45560693435798e-14 |
| AP1M1 | 6.33034745026403e-19 | 0.347232264550048 | 0.098 | 0.068 | 1.56119028818412e-14 |
| AC004687.1 | 8.50211640512583e-19 | 0.115506459640766 | 0.231 | 0.183 | 2.09679194783213e-14 |
| TESC | 8.74360060614571e-19 | 0.394401989158673 | 0.14 | 0.106 | 2.15634678148765e-14 |
| PPP1R35 | 1.13258449178777e-18 | 0.289285455628735 | 0.1 | 0.07 | 2.79317987364701e-14 |
| TOB1 | 1.14722374713082e-18 | 0.106204681794123 | 0.213 | 0.166 | 2.82928320517402e-14 |

| Gene | P_val | Avg_log2FC | Pct.1 | Pct.2 | P_val_adj |
|---|---|---|---|---|---|
| ALAS2 | 1.16324220760703e-18 | 0.150169585128501 | 0.07 | 0.045 | 2.86878793240045e-14 |
| APOBEC3B | 1.20446615032913e-18 | 0.343696842840981 | 0.077 | 0.051 | 2.97045441994171e-14 |
| MAP3K11 | 1.48745353917735e-18 | 0.174636062274343 | 0.178 | 0.137 | 3.66835791831917e-14 |
| AKIRIN1 | 1.50618338988146e-18 | 0.102382935317521 | 0.234 | 0.185 | 3.71454947612566e-14 |
| ZFAND2B | 1.5927777773378e-18 | 0.360603580364681 | 0.095 | 0.067 | 3.92810855447047e-14 |
| TRABD | 1.62445688745501e-18 | 0.15776108945881 | 0.228 | 0.181 | 4.00623557584155e-14 |
| AGER | 1.73425883368336e-18 | 0.304477629706723 | 0.073 | 0.048 | 4.2770291356299e-14 |
| TOLLIP | 1.74498810637281e-18 | 0.327459761101156 | 0.104 | 0.075 | 4.30348966793662e-14 |
| IFITM3 | 1.84179660869381e-18 | 0.117198691716328 | 0.977 | 0.924 | 4.54223879636069e-14 |
| CCNDBP1 | 1.92516283956771e-18 | 0.145843500652979 | 0.282 | 0.231 | 4.74783659494189e-14 |
| ARAP3 | 2.14834313485229e-18 | 0.160824659421872 | 0.094 | 0.065 | 5.29824383917271e-14 |
| GRINA | 2.34247316815281e-18 | 0.112206562815187 | 0.491 | 0.422 | 5.77700732729846e-14 |
| SNN | 2.39107344707125e-18 | 0.115602920196138 | 0.134 | 0.099 | 5.89686533516711e-14 |
| WDTC1 | 3.01833041470445e-18 | 0.214785300661438 | 0.142 | 0.106 | 7.44380646874412e-14 |
| DENND4B | 3.49727326484645e-18 | 0.189854972702948 | 0.152 | 0.115 | 8.62497532576433e-14 |
| ARPC4 | 3.51253662511362e-18 | 0.107329719786731 | 0.304 | 0.249 | 8.66261782485521e-14 |
| APBB3 | 3.59755980276579e-18 | 0.367103393590468 | 0.067 | 0.043 | 8.87230198558099e-14 |
| PAF1 | 3.88270548815292e-18 | 0.345811252974127 | 0.099 | 0.07 | 9.57552827488274e-14 |
| TBC1D10B | 4.17307309775731e-18 | 0.31579314450013 | 0.091 | 0.064 | 1.02916328736891e-13 |
| PRKCZ | 4.18456988806668e-18 | 0.34125616215995 | 0.083 | 0.057 | 1.031998625795e-13 |
| EXOSC4 | 4.49467953614085e-18 | 0.334231152799793 | 0.104 | 0.075 | 1.10847786720306e-13 |
| MINDY1 | 7.75316368461086e-18 | 0.28150019193823 | 0.088 | 0.061 | 1.91208522789873e-13 |
| CTSZ | 8.91929462413621e-18 | 0.13041995105972 | 0.246 | 0.198 | 2.19967644020447e-13 |
| APOBEC3G | 9.97942423616326e-18 | 0.231294818193292 | 0.139 | 0.104 | 2.46112560512258e-13 |
| GBA | 1.02292744871888e-17 | 0.288711206888133 | 0.151 | 0.116 | 2.52274367403051e-13 |
| ATXN7L3 | 1.04490531790659e-17 | 0.339369982739383 | 0.098 | 0.069 | 2.57694549502124e-13 |
| UTS2 | 1.29012684025988e-17 | 0.256245146649545 | 0.095 | 0.067 | 3.18171081344891e-13 |
| OGFR | 1.37906878038008e-17 | 0.114973405186776 | 0.24 | 0.194 | 3.40105942617334e-13 |
| GRK5 | 1.59591339833298e-17 | 0.211263526411453 | 0.065 | 0.042 | 3.9358416229688e-13 |
| WDR1 | 1.71414520859606e-17 | 0.101200998259489 | 0.293 | 0.24 | 4.22742491343959e-13 |
| AC008763.3 | 2.03056880110048e-17 | 0.419958749457037 | 0.085 | 0.059 | 5.007788777274e-13 |
| TMC8 | 2.16584705570893e-17 | 0.19658306155904 | 0.145 | 0.111 | 5.34141200878937e-13 |

| Gene | P_val | Avg_log2FC | Pct. 1 | Pct. 2 | P_val_adj |
|---|---|---|---|---|---|
| ZNF839 | 2.17966320198433e-17 | 0.515638928337504 | 0.053 | 0.033 | 5.37548538873376e-13 |
| TMCO3 | 2.20761102730084e-17 | 0.267765479353544 | 0.129 | 0.096 | 5.44441031552934e-13 |
| ZBTB7A | 2.66018183346686e-17 | 0.199666885834562 | 0.147 | 0.112 | 6.56054043769598e-13 |
| PANX2 | 2.7846948457226e-17 | 0.184841372394077 | 0.093 | 0.065 | 6.86761442852108e-13 |
| GPR27 | 3.01086983767272e-17 | 0.30933340011378 | 0.086 | 0.06 | 7.42540719366846e-13 |
| CARHSP1 | 3.36783945588024e-17 | 0.341382497426665 | 0.088 | 0.062 | 8.30576566609185e-13 |
| FHL3 | 4.39810833003106e-17 | 0.288602428950334 | 0.111 | 0.082 | 1.08466147635226e-12 |
| ADD1 | 4.65391188086413e-17 | 0.139774059730454 | 0.203 | 0.162 | 1.14774774805871e-12 |
| INPP4A | 4.7947831421416e-17 | 0.232212517768085 | 0.098 | 0.07 | 1.18248941851496e-12 |
| SH3BP5L | 4.90168646660859e-17 | 0.532125172708489 | 0.05 | 0.03 | 1.20885391639501e-12 |
| PGAP6 | 4.98238751589288e-17 | 0.146469995262058 | 0.132 | 0.099 | 1.2287564091695e-12 |
| DPH3 | 5.93201736576401e-17 | 0.160916973299523 | 0.149 | 0.113 | 1.46295412274472e-12 |
| GLTP | 6.2102286508436e-17 | 0.206075134092445 | 0.142 | 0.108 | 1.53156658987105e-12 |
| TPRG1L | 6.39878044718865e-17 | 0.194446533614745 | 0.118 | 0.087 | 1.57806723388567e-12 |
| GDI1 | 6.52132123597781e-17 | 0.108095798657417 | 0.169 | 0.13 | 1.60828824321685e-12 |
| FES | 6.64693469021101e-17 | 0.203454080057411 | 0.196 | 0.157 | 1.63926703329984e-12 |
| CASC3 | 7.25000081534275e-17 | 0.102240864708215 | 0.156 | 0.119 | 1.78799520107983e-12 |
| SPATA13 | 7.68590320516807e-17 | 0.131597269970469 | 0.169 | 0.131 | 1.89549744845855e-12 |
| CDC34 | 8.11980221265441e-17 | 0.24872835 | 0.106 | 0.077 | 2.00250562168483e-12 |
| ATP2A3 | 8.20763466082505e-17 | 0.120602444861499 | 0.171 | 0.133 | 2.02416686005267e-12 |
| TTLL3 | 8.45578269852662e-17 | 0.301861592626047 | 0.064 | 0.042 | 2.08536512911064e-12 |
| TBKBP1 | 8.88690377984054e-17 | 0.392174342240905 | 0.06 | 0.039 | 2.19168821018427e-12 |
| SLC25A28 | 9.99500284864006e-17 | 0.144704310806375 | 0.161 | 0.124 | 2.46496760253161e-12 |
| AL049634.2 | 1.04062287154244e-16 | 0.205403901644959 | 0.124 | 0.093 | 2.56638412579796e-12 |
| ATM | 1.15492595470969e-16 | 0.108554043282574 | 0.131 | 0.098 | 2.84827838950504e-12 |
| ZNF592 | 1.180807927275e-16 | 0.304739214583738 | 0.091 | 0.065 | 2.9121085102456e-12 |
| SCARF1 | 1.19081969762113e-16 | 0.114726778988677 | 0.194 | 0.153 | 2.93679953827323e-12 |
| PPM1M | 1.19434849454806e-16 | 0.261356424839569 | 0.124 | 0.093 | 2.94550225725442e-12 |
| ARHGEF18 | 1.41776478535058e-16 | 0.489472283485322 | 0.053 | 0.033 | 3.49649151363161e-12 |
| TLK1 | 1.90596459134168e-16 | 0.103864352444281 | 0.176 | 0.138 | 4.70048987516685e-12 |
| ZDHHC17 | 1.93276972804762e-16 | 0.125630401431102 | 0.173 | 0.134 | 4.76659670331104e-12 |
| STARD3 | 2.4563561900861e-16 | 0.161314359434751 | 0.124 | 0.093 | 6.05786563599035e-12 |

| Gene | P_val | Avg_log2FC | Pct.1 | Pct.2 | P_val_adj |
|---|---|---|---|---|---|
| SLC9A3R1 | 2.5667888322523e-16 | 0.106713394214246 | 0.242 | 0.196 | 6.33021461810062e-12 |
| ABR | 2.83810445861516e-16 | 0.183747090173413 | 0.128 | 0.097 | 6.99933321583671e-12 |
| MSRA | 2.87829143121138e-16 | 0.18106247625011 | 0.149 | 0.114 | 7.09844232765351e-12 |
| GPAT4 | 3.04296037115233e-16 | 0.278599301265928 | 0.081 | 0.056 | 7.50454886733588e-12 |
| MRPL28 | 3.24184872432272e-16 | 0.266263110001618 | 0.119 | 0.089 | 7.9950473239247e-12 |
| UBXN6 | 3.53621233072799e-16 | 0.239628389481649 | 0.084 | 0.059 | 8.72100685004137e-12 |
| NCK2 | 3.61868275546818e-16 | 0.416851745308657 | 0.065 | 0.044 | 8.92439541153562e-12 |
| PLEKHM2 | 3.65819913652915e-16 | 0.112223217377149 | 0.175 | 0.137 | 9.02185071050819e-12 |
| ZNF276 | 3.70055348077984e-16 | 0.188649107627573 | 0.129 | 0.097 | 9.12630499429923e-12 |
| TMEM43 | 3.74172830493584e-16 | 0.232349298491599 | 0.099 | 0.072 | 9.22785034563277e-12 |
| CCDC159 | 3.82442370794025e-16 | 0.351151898555703 | 0.087 | 0.062 | 9.43179374852225e-12 |
| MACROH2A1 | 4.20947544776018e-16 | 0.11108411481631 | 0.37 | 0.314 | 1.03814083492662e-11 |
| RBM38 | 4.3582725824154e-16 | 0.242851743571846 | 0.122 | 0.092 | 1.07483718427529e-11 |
| PGAM1 | 4.4401614757188e-16 | 0.145857219503605 | 0.233 | 0.19 | 1.09503262314177e-11 |
| REPS2 | 4.90132724400462e-16 | 0.173277905747061 | 0.098 | 0.07 | 1.20876532491642e-11 |
| ALDOA | 4.97739226464109e-16 | 0.318024145877507 | 0.079 | 0.055 | 1.22752448030578e-11 |
| DSE | 5.39680315154122e-16 | 0.265579960120545 | 0.098 | 0.071 | 1.3309595932331e-11 |
| SIPA1L2 | 6.55426975051605e-16 | 0.107405384373223 | 0.157 | 0.122 | 1.61641400587227e-11 |
| PLSCR1 | 6.64985697574696e-16 | 0.108481597415855 | 0.527 | 0.461 | 1.63998772735872e-11 |
| TREML2 | 7.7711410363037e-16 | 0.264750390401101 | 0.093 | 0.067 | 1.91651880237322e-11 |
| LRCH4 | 8.39846874911169e-16 | 0.292388832557316 | 0.075 | 0.051 | 2.07123036290593e-11 |
| CMTM3 | 8.401885160349e-16 | 0.158936354789664 | 0.159 | 0.123 | 2.07207291824527e-11 |
| QSOX1 | 8.52717411906278e-16 | 0.197094032275231 | 0.188 | 0.151 | 2.10297168124326e-11 |
| FAM120AOS | 9.25635544031804e-16 | 0.24512077999022 | 0.127 | 0.096 | 2.28280237869123e-11 |
| TNNI2 | 9.60999705053078e-16 | 0.268001774744295 | 0.073 | 0.05 | 2.3700174726019e-11 |
| AGAP2 | 1.15694841195635e-15 | 0.501109556488693 | 0.054 | 0.035 | 2.85326617356676e-11 |
| USB1 | 1.29303180280498e-15 | 0.107136906163705 | 0.241 | 0.197 | 3.18887503207763e-11 |
| EIF4A1 | 1.50837321337183e-15 | 0.121320981558488 | 0.113 | 0.084 | 3.71995001881761e-11 |
| SERTAD1 | 1.76808739297625e-15 | 0.270103086795968 | 0.081 | 0.057 | 4.36045712855803e-11 |
| PGS1 | 1.95470403653808e-15 | 0.126511682970771 | 0.371 | 0.317 | 4.8206910949102e-11 |
| NFKBID | 2.03845470941903e-15 | 0.155832206640338 | 0.153 | 0.119 | 5.02723700436921e-11 |
| RIC8A | 2.2208434660032e-15 | 0.339115283299034 | 0.079 | 0.055 | 5.47704415585709e-11 |

| Gene | P_val | Avg_log2FC | Pct.1 | Pct.2 | P_val_adj |
|---|---|---|---|---|---|
| PPP1R3D | 2.30994257656697e-15 | 0.319039161056934 | 0.094 | 0.068 | 5.69678038232946e-11 |
| MAN2A2 | 2.3562938169428e-15 | 0.155000508823861 | 0.155 | 0.12 | 5.81109181134434e-11 |
| ING1 | 2.4024937064213e-15 | 0.276182099608357 | 0.09 | 0.065 | 5.92502997877621e-11 |
| CDC37 | 2.7645569601058e-15 | 0.116056460293179 | 0.254 | 0.21 | 6.81795037501293e-11 |
| TRIM8 | 2.78199585941711e-15 | 0.101568124604046 | 0.196 | 0.156 | 6.86095818849448e-11 |
| KCNE1 | 2.83329848389388e-15 | 0.209670298851206 | 0.127 | 0.097 | 6.98748072097908e-11 |
| AC079781.5 | 2.88198615141301e-15 | 0.252195259602007 | 0.069 | 0.047 | 7.10755424661476e-11 |
| NUMA1 | 3.19247189586135e-15 | 0.379351265393642 | 0.076 | 0.053 | 7.87327418957326e-11 |
| CAMK2G | 3.2135355886075e-15 | 0.125445578618413 | 0.142 | 0.11 | 7.92522146862381e-11 |
| IKBKG | 3.3303074254889e-15 | 0.160183096069318 | 0.107 | 0.079 | 8.21320417274073e-11 |
| IMPA2 | 3.66059645917779e-15 | 0.173297571669625 | 0.136 | 0.105 | 9.02776298762426e-11 |
| CHPT1 | 3.70313734016945e-15 | 0.187080405859412 | 0.139 | 0.107 | 9.13267730832589e-11 |
| TOR1A | 3.88395536243579e-15 | 0.251146617346885 | 0.116 | 0.088 | 9.57861071483913e-11 |
| FURIN | 3.88748665700046e-15 | 0.129197145580831 | 0.218 | 0.177 | 9.58731959349455e-11 |
| DNAJC4 | 4.23842137546219e-15 | 0.390198670195043 | 0.081 | 0.057 | 1.04527947961648e-10 |
| GSK3A | 4.39338081140405e-15 | 0.242202866631302 | 0.095 | 0.069 | 1.08349557570847e-10 |
| TMBIM1 | 5.05842195787175e-15 | 0.223486842937563 | 0.12 | 0.091 | 1.24750802325033e-10 |
| EPN1 | 5.08851497606139e-15 | 0.150131540052348 | 0.119 | 0.089 | 1.25492956339626e-10 |
| CYP1B1 | 5.22300199541604e-15 | 0.180474265227434 | 0.183 | 0.146 | 1.2880967521095e-10 |
| EZH1 | 5.31899824274752e-15 | 0.13542990062813 | 0.105 | 0.077 | 1.31177134662639e-10 |
| CIDEB | 5.51200118585391e-15 | 0.415777036048646 | 0.052 | 0.033 | 1.35936973245529e-10 |
| IFIT5 | 6.24344356709324e-15 | 0.177138938711818 | 0.124 | 0.094 | 1.53975805251653e-10 |
| NMRK1 | 6.94129917365341e-15 | 0.219693294273499 | 0.1 | 0.073 | 1.7118632022064e-10 |
| HSD11B1-AS1 | 7.04336113314052e-15 | 0.102868940460105 | 0.145 | 0.111 | 1.73703372265512e-10 |
| RAB11FIP4 | 7.26609620924674e-15 | 0.109797657276466 | 0.129 | 0.098 | 1.79196464712443e-10 |
| LMAN2 | 8.25250713758755e-15 | 0.15226791964055 | 0.184 | 0.147 | 2.03523331027184e-10 |
| ACAA1 | 8.29184634356311e-15 | 0.103425392136511 | 0.202 | 0.162 | 2.04493514524953e-10 |
| EIF4E3 | 8.59210320806644e-15 | 0.138963765666607 | 0.18 | 0.144 | 2.11898449317334e-10 |
| KIAA2013 | 9.3049001953402e-15 | 0.161952043170792 | 0.146 | 0.113 | 2.2947744861748e-10 |
| TRPM6 | 1.43572404155625e-14 | 0.162312265352433 | 0.111 | 0.083 | 3.54078263128603e-10 |
| PRRC2A | 1.48237894027187e-14 | 0.145477337140918 | 0.154 | 0.121 | 3.65584294249848e-10 |
| GMPR2 | 1.49156927982358e-14 | 0.250036288829552 | 0.099 | 0.073 | 3.67850815790091e-10 |

| Gene | P_val | Avg_log2FC | Pct.1 | Pct.2 | P_val_adj |
|---|---|---|---|---|---|
| ACTR1A | 1.63348716866617e-14 | 0.225726111277098 | 0.102 | 0.075 | 4.0285060553645e-10 |
| MAP1S | 1.65703978763105e-14 | 0.306907009609524 | 0.069 | 0.047 | 4.0865915242557e-10 |
| DDIT4 | 1.66878421511334e-14 | 0.168220785563062 | 0.151 | 0.118 | 4.11555563131253e-10 |
| ARRDC1 | 1.7158743870813e-14 | 0.28019398355633 | 0.1 | 0.074 | 4.23168941341991e-10 |
| SNRNP70 | 2.09384920384347e-14 | 0.149606005807487 | 0.164 | 0.13 | 5.16385090651877e-10 |
| PCYT1A | 2.12333146365889e-14 | 0.15543797531797 | 0.151 | 0.118 | 5.23656005567555e-10 |
| TMEM131L | 2.1302653685944e-14 | 0.100262641363383 | 0.174 | 0.139 | 5.25366045202751e-10 |
| MBOAT1 | 2.14002634209645e-14 | 0.455799413759752 | 0.064 | 0.044 | 5.27773296487827e-10 |
| NOSIP | 2.43529714897326e-14 | 0.189808032832319 | 0.122 | 0.093 | 6.00592982879786e-10 |
| GNG10 | 2.9142260376077e-14 | 0.240503937200883 | 0.128 | 0.098 | 7.18706425394812e-10 |
| JDP2 | 2.98779969688456e-14 | 0.198739743867724 | 0.125 | 0.096 | 7.36851161245671e-10 |
| CYB5R3 | 3.64429849072384e-14 | 0.276675512767127 | 0.11 | 0.083 | 8.98756893782314e-10 |
| CHMP1A | 3.64851957403259e-14 | 0.179406321203281 | 0.121 | 0.093 | 8.99797897347917e-10 |
| ZFAND2A | 3.83461275359194e-14 | 0.130741643796437 | 0.144 | 0.112 | 9.45692197290845e-10 |
| PRKAR2A | 3.90361467156129e-14 | 0.2437462052235 | 0.104 | 0.078 | 9.62709450300445e-10 |
| RELA | 4.21194918826537e-14 | 0.148114931836458 | 0.136 | 0.105 | 1.03875090881001e-09 |
| KLF16 | 4.27869332789524e-14 | 0.35504988082585 | 0.06 | 0.041 | 1.05521134852552e-09 |
| KAT2B | 4.32801921255785e-14 | 0.208587593008938 | 0.116 | 0.088 | 1.06737609820102e-09 |
| AC136475.5 | 4.37639006062089e-14 | 0.325263328280167 | 0.075 | 0.053 | 1.07930531675032e-09 |
| UBA1 | 5.10523703652333e-14 | 0.141003197621332 | 0.164 | 0.13 | 1.25905355794738e-09 |
| TRADD | 6.41948476865314e-14 | 0.28281128019848 | 0.073 | 0.051 | 1.58317333364524e-09 |
| CMPK2 | 6.80605486405143e-14 | 0.114165915783825 | 0.187 | 0.15 | 1.67850925057236e-09 |
| YIPF3 | 7.28454082980729e-14 | 0.120142918585394 | 0.17 | 0.135 | 1.79651345944707e-09 |
| CAMTA2 | 7.69308536560263e-14 | 0.30129364820792 | 0.081 | 0.059 | 1.89726871286492e-09 |
| SLC25A44 | 1.02496022112832e-13 | 0.335948731746564 | 0.069 | 0.048 | 2.52775689734666e-09 |
| CTDSP2 | 1.04704718648725e-13 | 0.170229912152388 | 0.117 | 0.089 | 2.58222777131484e-09 |
| TACC3 | 1.08380234144587e-13 | 0.128530133740108 | 0.17 | 0.136 | 2.67287333447381e-09 |
| GDE1 | 1.13497240709841e-13 | 0.181382530740873 | 0.113 | 0.086 | 2.79906895038609e-09 |
| WDR45 | 1.43716781972303e-13 | 0.19637785421356 | 0.088 | 0.064 | 3.54434327700094e-09 |
| PDZD8 | 1.47050537731271e-13 | 0.148256047170747 | 0.162 | 0.13 | 3.6265603615286e-09 |
| SLC38A10 | 1.54746800503137e-13 | 0.273001117339941 | 0.104 | 0.079 | 3.81636559400837e-09 |
| TAZ | 1.67296871972615e-13 | 0.263826502956591 | 0.095 | 0.071 | 4.12587545658862e-09 |

| Gene | P_val | Avg_log2FC | Pct. 1 | Pct. 2 | P_val_adj |
|---|---|---|---|---|---|
| ZNF354A | 2.20260691303541e-13 | 0.450143382852321 | 0.054 | 0.036 | 5.43206916892793e-09 |
| AP002807.1 | 2.28995953515749e-13 | 0.229190147214663 | 0.069 | 0.048 | 5.64749820560541e-09 |
| H2AJ | 2.32141625599418e-13 | 0.158974316131177 | 0.113 | 0.085 | 5.72507677053284e-09 |
| SH3GL1 | 2.74935729516825e-13 | 0.159784745193925 | 0.098 | 0.073 | 6.78046496134395e-09 |
| SEPTIN9 | 2.99257589183498e-13 | 0.112967210822421 | 0.14 | 0.11 | 7.38029066444343e-09 |
| DTX2 | 3.02978071534973e-13 | 0.359065368369328 | 0.064 | 0.044 | 7.47204520019551e-09 |
| NR4A2 | 3.43885511837705e-13 | 0.130851128004585 | 0.139 | 0.109 | 8.48090449294147e-09 |
| PMF1 | 3.84054779201532e-13 | 0.334127517356837 | 0.073 | 0.052 | 9.47155896466817e-09 |
| FCHO1 | 3.91137292619485e-13 | 0.189332460207252 | 0.081 | 0.059 | 9.64622791058174e-09 |
| PHYKPL | 3.92840117902138e-13 | 0.178334504529841 | 0.121 | 0.093 | 9.68822298770253e-09 |
| FAM217B | 4.06134535117047e-13 | 0.163102001570541 | 0.135 | 0.106 | 1.00160899050566e-08 |
| BRMS1 | 4.09690250967631e-13 | 0.199464448492044 | 0.093 | 0.069 | 1.01037809693637e-08 |
| FBXW5 | 4.55382554894609e-13 | 0.209347285018641 | 0.105 | 0.08 | 1.12306445688109e-08 |
| BCKDK | 4.90994719184291e-13 | 0.20309290619864 | 0.111 | 0.085 | 1.2108911764523e-08 |
| ERGIC2 | 5.27086706095647e-13 | 0.168716269921913 | 0.098 | 0.073 | 1.29990123457308e-08 |
| AKTIP | 5.37990947949519e-13 | 0.273167749953508 | 0.079 | 0.057 | 1.3267932758331e-08 |
| SNX27 | 5.55069633930901e-13 | 0.128481185915052 | 0.117 | 0.09 | 1.36891273120039e-08 |
| TNRC18 | 6.28468231025902e-13 | 0.153373690866764 | 0.151 | 0.121 | 1.54992835135608e-08 |
| SFXN5 | 6.4111148609319e-13 | 0.383738923673453 | 0.05 | 0.033 | 1.58110914700302e-08 |
| HVCN1 | 6.54808019331606e-13 | 0.244036708562864 | 0.09 | 0.067 | 1.61488753727561e-08 |
| NXF1 | 6.96956358636694e-13 | 0.107422400221466 | 0.201 | 0.165 | 1.71883377166982e-08 |
| EXOC7 | 7.49417938065194e-13 | 0.302369798135589 | 0.096 | 0.072 | 1.84821451885638e-08 |
| HACD4 | 1.002485812949e-12 | 0.11511927660397 | 0.218 | 0.182 | 2.47233051189481e-08 |
| SF3A1 | 1.07359496101268e-12 | 0.111406755581149 | 0.15 | 0.119 | 2.64769989284947e-08 |
| RBM42 | 1.09352424557064e-12 | 0.210671945970019 | 0.118 | 0.091 | 2.69684949442632e-08 |
| TOP3A | 1.09896212666982e-12 | 0.127541997233397 | 0.07 | 0.05 | 2.71026039679311e-08 |
| COMMD5 | 1.13886366410596e-12 | 0.291576809302384 | 0.08 | 0.058 | 2.80866556841811e-08 |
| DDX49 | 1.14825496970249e-12 | 0.314717820435378 | 0.066 | 0.047 | 2.83182640628029e-08 |
| WIPI1 | 1.2490826006185e-12 | 0.300082557834152 | 0.092 | 0.069 | 3.08048750964534e-08 |
| RHOB | 1.35624967792142e-12 | 0.131454908640423 | 0.151 | 0.12 | 3.34478295568981e-08 |
| ATG4D | 1.37367162048609e-12 | 0.266874133393716 | 0.07 | 0.05 | 3.3877489504428e-08 |
| SHFL | 1.42537656999118e-12 | 0.101677904333418 | 0.176 | 0.143 | 3.51526369691225e-08 |

| Gene | P_val | Avg_log2FC | Pct.1 | Pct.2 | P_val_adj |
|---|---|---|---|---|---|
| LRSAM1 | 1.48095425048518e-12 | 0.220631865374636 | 0.07 | 0.05 | 3.65232937254655e-08 |
| CYHR1 | 1.49220962721893e-12 | 0.224298737854639 | 0.065 | 0.046 | 3.68008738264733e-08 |
| ARHGAP18 | 1.65590393556051e-12 | 0.429490588475239 | 0.058 | 0.04 | 4.08379028587933e-08 |
| PHKG2 | 1.71279745777144e-12 | 0.241853341274427 | 0.078 | 0.057 | 4.22410109035592e-08 |
| PCMT1 | 1.75636545299035e-12 | 0.124534755658843 | 0.227 | 0.19 | 4.3315484801648e-08 |
| PLEKHG1 | 1.83562850945925e-12 | 0.162160253435529 | 0.081 | 0.059 | 4.52702703002841e-08 |
| IKBIP | 1.89017908995995e-12 | 0.155372118196704 | 0.088 | 0.065 | 4.66155967165922e-08 |
| ARL11 | 2.11142221071204e-12 | 0.201272064140844 | 0.1 | 0.076 | 5.20718945605803e-08 |
| HMOX2 | 2.11965709558752e-12 | 0.14539726521265 | 0.072 | 0.051 | 5.22749832913793e-08 |
| NFYA | 2.13897660697699e-12 | 0.14466434225598 | 0.068 | 0.048 | 5.27514410812665e-08 |
| MILR1 | 2.14916973249697e-12 | 0.230878272920302 | 0.11 | 0.085 | 5.30028239428403e-08 |
| FLNA | 2.33684290576498e-12 | 0.167953575740249 | 0.245 | 0.209 | 5.7631219741976e-08 |
| TADA2B | 2.59168818863331e-12 | 0.231478784795673 | 0.089 | 0.066 | 6.39162141080746e-08 |
| TLR6 | 2.6036292743776e-12 | 0.117161061477996 | 0.112 | 0.086 | 6.42107051647004e-08 |
| AC084871.2 | 2.60710385745038e-12 | 0.358583372235003 | 0.077 | 0.056 | 6.42963953324412e-08 |
| TSPYL2 | 2.65479933228039e-12 | 0.200708895383618 | 0.085 | 0.062 | 6.5472661132699e-08 |
| ARID3B | 2.71907691140938e-12 | 0.212382967707371 | 0.106 | 0.081 | 6.70578747891781e-08 |
| MIR3945HG | 3.05648907207413e-12 | 0.139882345857362 | 0.117 | 0.09 | 7.53791334954922e-08 |
| IFITM1 | 3.35021280767554e-12 | 0.117282433992514 | 0.754 | 0.692 | 8.2622948262894e-08 |
| WASF2 | 3.82300968156703e-12 | 0.101226702969231 | 0.228 | 0.19 | 9.42830647668061e-08 |
| CDK5RAP2 | 4.32583140613174e-12 | 0.257599527799565 | 0.095 | 0.072 | 1.06683654138021e-07 |
| POLR2C | 4.47442094057812e-12 | 0.24918776818456 | 0.075 | 0.054 | 1.10348169236538e-07 |
| DAPK3 | 5.10588223004973e-12 | 0.251907433431441 | 0.056 | 0.039 | 1.25921267557486e-07 |
| AKAP8L | 5.12312393389049e-12 | 0.115003984080135 | 0.116 | 0.09 | 1.26346482457607e-07 |
| WBP1L | 5.23065971589785e-12 | 0.157446817463085 | 0.074 | 0.053 | 1.28998529913473e-07 |
| AC007278.1 | 5.31684390249363e-12 | 0.263479856405775 | 0.052 | 0.036 | 1.31124004323298e-07 |
| GPR160 | 6.05728809492871e-12 | 0.17504008533821 | 0.156 | 0.126 | 1.49384838997132e-07 |
| TMEM175 | 6.15567630446366e-12 | 0.174009543289097 | 0.056 | 0.038 | 1.51811289020683e-07 |
| CMTM7 | 6.1889845135952e-12 | 0.219065524964934 | 0.11 | 0.085 | 1.52632736074285e-07 |
| STK16 | 6.25889851000168e-12 | 0.370882815907655 | 0.056 | 0.039 | 1.54356955053661e-07 |
| CHPF2 | 1.02014933631648e-11 | 0.24704573434213 | 0.078 | 0.057 | 2.5158922932237e-07 |
| OTUD1 | 1.18623930914243e-11 | 0.136715717280704 | 0.196 | 0.162 | 2.92550338420706e-07 |

| Gene | P_val | Avg_log2FC | Pct.1 | Pct.2 | P_val_adj |
|---|---|---|---|---|---|
| GALNS | 1.22259353471721e-11 | 0.317829998996603 | 0.075 | 0.055 | 3.01516017531959e-07 |
| MMP25-AS1 | 1.92814831392333e-11 | 0.276526344251981 | 0.057 | 0.04 | 4.75519937179773e-07 |
| UBTF | 2.23542644477353e-11 | 0.228218052770641 | 0.083 | 0.062 | 5.51300869810047e-07 |
| ID2 | 2.25777087551443e-11 | 0.175265442677628 | 0.229 | 0.194 | 5.56811453319369e-07 |
| MTG2 | 2.52937048084493e-11 | 0.142350842674862 | 0.069 | 0.05 | 6.23793347985978e-07 |
| DNMT3A | 2.64342832610908e-11 | 0.226254076740315 | 0.057 | 0.04 | 6.51922293785021e-07 |
| SGSH | 2.70101899195162e-11 | 0.316037848091659 | 0.055 | 0.038 | 6.66125303795109e-07 |
| CRACR2A | 2.80299209198097e-11 | 0.173667403423102 | 0.062 | 0.044 | 6.91273909724347e-07 |
| RETREG2 | 3.74993948133631e-11 | 0.119433567129478 | 0.126 | 0.1 | 9.24810074887162e-07 |
| KIF1C | 4.00933951366452e-11 | 0.298013079053184 | 0.056 | 0.039 | 9.88783310859943e-07 |
| SAP30L | 4.31910191455797e-11 | 0.206066645762911 | 0.072 | 0.053 | 1.06517691416829e-06 |
| DEDD | 4.96961321010353e-11 | 0.195942480905553 | 0.086 | 0.065 | 1.22560600987573e-06 |
| CYP27A1 | 6.59113824897866e-11 | 0.112297289902992 | 0.184 | 0.152 | 1.62550651496312e-06 |
| RENBP | 6.8986677063851e-11 | 0.319894868733107 | 0.05 | 0.034 | 1.70134942974869e-06 |
| ZDHHC7 | 6.92136169286239e-11 | 0.122429978231452 | 0.096 | 0.074 | 1.70694622069372e-06 |
| LINC01410 | 7.43174639283184e-11 | 0.236805924596414 | 0.074 | 0.055 | 1.83281729540019e-06 |
| SUCLG1 | 8.16654926960062e-11 | 0.223643515697951 | 0.104 | 0.082 | 2.01403438086891e-06 |
| SSH3 | 9.04095764732498e-11 | 0.238859309782893 | 0.051 | 0.036 | 2.22968097498329e-06 |
| SUPT20H | 9.28420275374061e-11 | 0.129481371224702 | 0.108 | 0.084 | 2.28967008312751e-06 |
| C1orf122 | 9.50198214292161e-11 | 0.250722736524094 | 0.087 | 0.066 | 2.34337883608733e-06 |
| TRPV2 | 1.00405358766687e-10 | 0.357741451979229 | 0.059 | 0.042 | 2.47619695790403e-06 |
| XAB2 | 1.00725782741178e-10 | 0.229792115014183 | 0.066 | 0.048 | 2.48409925396294e-06 |
| PURB | 1.05430253476361e-10 | 0.119110735202647 | 0.1 | 0.077 | 2.60012091123401e-06 |
| TSPAN14 | 1.22823734854343e-10 | 0.102930396224266 | 0.132 | 0.106 | 3.0290789489778e-06 |
| HAUS4 | 1.39852106717696e-10 | 0.228929915421048 | 0.067 | 0.049 | 3.44903265587183e-06 |
| ITGA5 | 1.54392476038112e-10 | 0.109786493120441 | 0.076 | 0.057 | 3.80762724405193e-06 |
| AGPAT1 | 1.5629527879404e-10 | 0.20712887183293 | 0.059 | 0.042 | 3.8545541656186e-06 |
| RNF216 | 1.76025648962518e-10 | 0.198805274997254 | 0.081 | 0.061 | 4.34114455471363e-06 |
| ADGRE1 | 1.91896134800462e-10 | 0.195075938272251 | 0.075 | 0.056 | 4.73254247644899e-06 |
| KRT23 | 2.52121346266976e-10 | 0.111972703730125 | 0.083 | 0.063 | 6.21781664163615e-06 |
| B3GNT2 | 3.13334041515058e-10 | 0.136044840284422 | 0.107 | 0.084 | 7.72744413184436e-06 |
| FXYD5 | 3.32710772148544e-10 | 0.108225767428139 | 0.202 | 0.17 | 8.2053130627274e-06 |

| Gene | P_val | Avg_log2FC | Pct. 1 | Pct. 2 | P_val_adj |
|---|---|---|---|---|---|
| TINF2 | 3.3981412195836e-10 | 0.252922525284174 | 0.092 | 0.071 | 8.38049587573707e-06 |
| TMEM184B | 3.47334666038621e-10 | 0.145166861458678 | 0.06 | 0.043 | 8.56596753384446e-06 |
| PPP6R1 | 3.55225304396871e-10 | 0.266119305730569 | 0.064 | 0.047 | 8.76056645703564e-06 |
| FBXO6 | 3.63111206253978e-10 | 0.165769531865155 | 0.152 | 0.126 | 8.95504856863559e-06 |
| KLHL18 | 3.75282667358518e-10 | 0.176015266889964 | 0.076 | 0.057 | 9.25522114239576e-06 |
| ARPC5L | 4.0859836422267e-10 | 0.154901496286436 | 0.129 | 0.104 | 1.00768528584595e-05 |
| UQCRFS1 | 4.35454051871303e-10 | 0.12036880655695 | 0.196 | 0.166 | 1.07391678272501e-05 |
| PKM | 4.5366519452242e-10 | 0.115318448498401 | 0.279 | 0.242 | 1.11882910273119e-05 |
| DHRS9 | 5.0135046943403e-10 | 0.197034776518034 | 0.126 | 0.102 | 1.2364305277182e-05 |
| CEP85L | 5.19928906966761e-10 | 0.10765980684009 | 0.089 | 0.068 | 1.28224867036143e-05 |
| AC044849.1 | 5.67014683164108e-10 | 0.180172071507303 | 0.053 | 0.038 | 1.39837161161932e-05 |
| WDR13 | 6.09410224931007e-10 | 0.251905837531091 | 0.055 | 0.04 | 1.50292749672485e-05 |
| SIRT7 | 6.87562757103898e-10 | 0.123146700781847 | 0.143 | 0.116 | 1.69566727156963e-05 |
| PPM1N | 8.0674384042073e-10 | 0.36982917806246 | 0.054 | 0.038 | 1.9895916592456e-05 |
| UBXN11 | 8.24656834195296e-10 | 0.262781406611088 | 0.064 | 0.047 | 2.03376868449244e-05 |
| TDRD9 | 8.82691007680025e-10 | 0.299184949726778 | 0.056 | 0.041 | 2.17689256314048e-05 |
| GPANK1 | 9.39718096742756e-10 | 0.183347064649312 | 0.076 | 0.058 | 2.31753277018698e-05 |
| DCAF10 | 9.57503586082125e-10 | 0.196904457332571 | 0.098 | 0.077 | 2.36139534399574e-05 |
| KAT5 | 1.04388770032359e-09 | 0.144833180429764 | 0.087 | 0.067 | 2.57443584653803e-05 |
| AC005632.3 | 1.04889109203143e-09 | 0.131947831703815 | 0.093 | 0.072 | 2.58677521116792e-05 |
| SDHAF3 | 1.25505300915647e-09 | 0.279594702532616 | 0.059 | 0.043 | 3.09521173118169e-05 |
| HAL | 1.28868062785694e-09 | 0.148610958458441 | 0.05 | 0.035 | 3.17814416442078e-05 |
| HS1BP3 | 1.38054370729531e-09 | 0.27647545125287 | 0.057 | 0.041 | 3.40469689093169e-05 |
| SMIM29 | 1.64239902465298e-09 | 0.162092319542055 | 0.074 | 0.056 | 4.05048447459918e-05 |
| YRDC | 1.64748771838162e-09 | 0.222997813938711 | 0.079 | 0.061 | 4.06303421107275e-05 |
| CBFA2T3 | 1.75630961475844e-09 | 0.199380279612568 | 0.053 | 0.038 | 4.33141077191725e-05 |
| EMILIN2 | 2.19958437914535e-09 | 0.126331466740724 | 0.148 | 0.122 | 5.42461499584826e-05 |
| SYVN1 | 2.27886524843345e-09 | 0.169733473157967 | 0.073 | 0.055 | 5.62013747568657e-05 |
| BAK1 | 2.5773400592331e-09 | 0.29788500923165 | 0.054 | 0.039 | 6.35623605408067e-05 |
| KHNYN | 2.88779688644144e-09 | 0.142737958302992 | 0.071 | 0.054 | 7.12188468134188e-05 |
| KIAA1958 | 3.03605670564285e-09 | 0.286740182276564 | 0.06 | 0.045 | 7.48752304745639e-05 |
| PRKCSH | 3.17579944448721e-09 | 0.110819426164544 | 0.13 | 0.106 | 7.83215658999435e-05 |

| Gene | P_val | Avg_log2FC | Pct.1 | Pct.2 | P_val_adj |
|---|---|---|---|---|---|
| PLIN5 | 3.32597214814968e-09 | 0.112154729282303 | 0.102 | 0.081 | 8.20251251176674e-05 |
| CDK5RAP3 | 3.4494761507275e-09 | 0.122280500167105 | 0.061 | 0.045 | 8.50709808292415e-05 |
| RAB37 | 3.76952741487079e-09 | 0.110430865890145 | 0.092 | 0.072 | 9.29640851055433e-05 |
| CYB5R1 | 3.96639748392942e-09 | 0.197640847095653 | 0.071 | 0.054 | 9.78192947486674e-05 |
| PCIF1 | 4.1337416543383e-09 | 0.158409251285389 | 0.094 | 0.074 | 0.000101946336679291 |
| ARSG | 4.19998754763276e-09 | 0.203975731513682 | 0.058 | 0.042 | 0.000103580092899719 |
| ARHGAP1 | 4.98651145192444e-09 | 0.195209080478644 | 0.075 | 0.058 | 0.00012297734542736 |
| MAP11 | 5.3517040091192e-09 | 0.213560398524824 | 0.053 | 0.039 | 0.000131983724272898 |
| AP2M1 | 6.18140789645535e-09 | 0.112524282616682 | 0.139 | 0.115 | 0.000152445881542382 |
| KMT5A | 6.29261786569021e-09 | 0.141151970380616 | 0.085 | 0.067 | 0.000155188541803652 |
| FAM89B | 6.72998183009978e-09 | 0.105899317444133 | 0.122 | 0.099 | 0.000165974811893921 |
| PGLS | 6.86977051786639e-09 | 0.100405153366118 | 0.106 | 0.085 | 0.000169422280511621 |
| CHD3 | 7.12357906835834e-09 | 0.207421566453441 | 0.109 | 0.088 | 0.000175681706983853 |
| SEMA4B | 7.1613184819058e-09 | 0.204648008681786 | 0.062 | 0.046 | 0.000176612436400761 |
| TP53I3 | 7.55076461870128e-09 | 0.269727329237468 | 0.056 | 0.042 | 0.000186216957026411 |
| NXPE3 | 8.36448362461193e-09 | 0.26163980805445 | 0.057 | 0.042 | 0.000206284895150179 |
| FOLR3 | 9.52243370726777e-09 | 0.264825747962522 | 0.089 | 0.071 | 0.000234842260088638 |
| GUSBP11 | 1.00739438220577e-08 | 0.118885177239743 | 0.084 | 0.065 | 0.000248443602539587 |
| RAB35 | 1.02322824505464e-08 | 0.100735218687335 | 0.092 | 0.073 | 0.000252348549795375 |
| KLC1 | 1.19471487574553e-08 | 0.112721148270959 | 0.077 | 0.059 | 0.000294640582656364 |
| PEX16 | 1.30942513979612e-08 | 0.188695558619032 | 0.063 | 0.048 | 0.000322930427976518 |
| S100Z | 1.36516150366258e-08 | 0.143579073176125 | 0.059 | 0.044 | 0.000336676130033266 |
| ZNF316 | 1.42688790980103e-08 | 0.106741762424116 | 0.052 | 0.038 | 0.00035189909631513 |
| ZNF865 | 1.47403791773542e-08 | 0.222466065601366 | 0.051 | 0.037 | 0.000363527231271909 |
| KCNE3 | 1.65163065214237e-08 | 0.132914366496936 | 0.082 | 0.064 | 0.000407325151431351 |
| ZNF687 | 1.6763225026659e-08 | 0.158723372096237 | 0.063 | 0.048 | 0.000413414655607465 |
| GTF2F1 | 1.74237594693867e-08 | 0.108638339700929 | 0.109 | 0.088 | 0.000429704756034015 |
| SMIM3 | 1.7758977469391e-08 | 0.307988530184415 | 0.05 | 0.036 | 0.000437971902350122 |
| PSMD2 | 1.81796216018947e-08 | 0.146459411720144 | 0.122 | 0.101 | 0.000448345827945927 |
| SLC3A2 | 2.02116266567841e-08 | 0.108614496453803 | 0.124 | 0.102 | 0.000498459136609609 |
| C9orf16 | 2.98405300730824e-08 | 0.125416180585581 | 0.141 | 0.119 | 0.000735927152662357 |
| CARD6 | 3.78024340189804e-08 | 0.205059293945292 | 0.063 | 0.048 | 0.000932283627776096 |

| Gene | P_val | Avg_log2FC | Pct.1 | Pct.2 | P_val_adj |
|---|---|---|---|---|---|
| BAG6 | 4.07191207212631e-08 | 0.146041181873757 | 0.107 | 0.087 | 0.00100421495522779 |
| RNASEL | 4.54629313481048e-08 | 0.211598204289819 | 0.05 | 0.037 | 0.00112120681290696 |
| POFUT2 | 4.5578480280565e-08 | 0.1008996413507 | 0.061 | 0.046 | 0.00112405648067929 |
| EMD | 4.97996970392094e-08 | 0.117659853484774 | 0.077 | 0.06 | 0.00122816012838098 |
| FBXL12 | 6.49476989462048e-08 | 0.110153714787203 | 0.058 | 0.044 | 0.0016017401514113 |
| AC093673.1 | 6.96026393745339e-08 | 0.326194595672224 | 0.051 | 0.038 | 0.00171654029225475 |
| ZNF296 | 7.23430587835116e-08 | 0.210097494790496 | 0.068 | 0.052 | 0.00178412451571896 |
| DVL3 | 9.01815731178795e-08 | 0.110595817851087 | 0.078 | 0.062 | 0.00222405795623314 |
| KDM5C | 1.01709004080427e-07 | 0.109504124049608 | 0.092 | 0.074 | 0.00250834745863148 |
| LINC00963 | 1.03875577766595e-07 | 0.10215713142668 | 0.091 | 0.073 | 0.00256177949887978 |
| MOAP1 | 1.12903905718767e-07 | 0.197364140000652 | 0.055 | 0.041 | 0.00278443612283622 |
| SSH1 | 1.13018283147543e-07 | 0.124502942523379 | 0.087 | 0.07 | 0.0027872568989847 |
| GUCD1 | 1.17661886372214e-07 | 0.152582215635257 | 0.072 | 0.056 | 0.00290177744171155 |
| SIAH2 | 1.32681123033909e-07 | 0.101456226089113 | 0.095 | 0.077 | 0.00327218185626226 |
| IPCEF1 | 1.49102151352677e-07 | 0.1214387118939 | 0.068 | 0.053 | 0.00367715725665972 |
| SKIV2L | 1.59698800536148e-07 | 0.261223387392447 | 0.055 | 0.042 | 0.00393849181882249 |
| AL109809.4 | 2.12143022274984e-07 | 0.170178094902675 | 0.055 | 0.042 | 0.00523187121534564 |
| RPS19BP1 | 2.23390741767239e-07 | 0.116152289110825 | 0.112 | 0.093 | 0.00550926247346366 |
| CORO1C | 2.70822861859428e-07 | 0.14791406120799 | 0.089 | 0.072 | 0.00667903341917721 |
| SNX11 | 2.74702261525113e-07 | 0.127621196990646 | 0.059 | 0.045 | 0.00677470717373233 |
| NPLOC4 | 2.8920490747515e-07 | 0.100472692802729 | 0.071 | 0.056 | 0.00713237142815215 |
| MED16 | 2.9006378389786e-07 | 0.217366651268457 | 0.055 | 0.042 | 0.00715355303848902 |
| CBX1 | 3.16677810551233e-07 | 0.159740123676171 | 0.086 | 0.07 | 0.00780990816381452 |
| AC097376.3 | 3.44493704883719e-07 | 0.137207280690236 | 0.081 | 0.065 | 0.00849590374984229 |
| EVA1B | 3.95030542739776e-07 | 0.244427695302134 | 0.053 | 0.04 | 0.00974224324504835 |
| USP20 | 3.97330403597885e-07 | 0.145009289855049 | 0.066 | 0.052 | 0.00979896241353104 |
| CACNA1E | 4.4358777730195e-07 | 0.130415423493314 | 0.069 | 0.054 | 0.0109397617638207 |
| SLC22A18 | 5.5225739168862e-07 | 0.145072759847546 | 0.062 | 0.049 | 0.0136197717938247 |
| TRPM2 | 6.80477351604118e-07 | 0.315493025005681 | 0.056 | 0.043 | 0.0167819324452608 |
| MOSPD3 | 8.46792365816784e-07 | 0.247872734071788 | 0.054 | 0.042 | 0.0208835933257735 |
| TANGO2 | 9.24881647602076e-07 | 0.102871299773153 | 0.063 | 0.05 | 0.0228094311931624 |
| ALDH3B1 | 1.19649419211838e-06 | 0.136828623901302 | 0.105 | 0.088 | 0.0295079397660234 |

| Gene | P_val | Avg_log2FC | Pct.1 | Pct.2 | P_val_adj |
|---|---|---|---|---|---|
| ATP6V1C1 | 1.25913306228918e-06 | 0.159098961905108 | 0.111 | 0.093 | 0.0310527395821758 |
| ATP2B4 | 1.26269966122663e-06 | 0.192479460402238 | 0.064 | 0.051 | 0.0311406990451713 |
| AP3B2 | 1.36336215989428e-06 | 0.164110841354407 | 0.052 | 0.04 | 0.0336232375873128 |
| SCAF1 | 1.38259385059274e-06 | 0.139575931206753 | 0.05 | 0.038 | 0.0340975295433181 |
| TSPAN32 | 1.3906827521249e-06 | 0.276566672847888 | 0.055 | 0.043 | 0.0342970180329044 |
| NAA60 | 1.62530192054433e-06 | 0.113192114695263 | 0.09 | 0.074 | 0.0400831959644642 |
| TRIM14 | 1.86028654039421e-06 | 0.207249303313646 | 0.057 | 0.045 | 0.0458783866592019 |
| AMN1 | 1.96897373040485e-06 | 0.188961424453118 | 0.052 | 0.04 | 0.0485588301392443 |
| PFKFB2 | 2.05823643631546e-06 | 0.141210203905955 | 0.069 | 0.055 | 0.0507602269924118 |
| GGA3 | 2.33070846163785e-06 | 0.130179830133699 | 0.085 | 0.07 | 0.0574799320809126 |
| CLEC5A | 3.64688278164045e-06 | 0.193448585705553 | 0.058 | 0.046 | 0.0899394231608168 |
| SLC26A6 | 3.68430246027876e-06 | 0.224765515397341 | 0.05 | 0.039 | 0.0908622672753949 |
| DHX58 | 4.05724577996846e-06 | 0.119643436651597 | 0.055 | 0.043 | 0.100059795425582 |
| NME6 | 5.91037344964772e-06 | 0.216942747860982 | 0.059 | 0.047 | 0.145761630015212 |
| LYL1 | 6.16858714533152e-06 | 0.179770481631355 | 0.062 | 0.05 | 0.152129696178166 |
| SF3A3 | 6.2505563230124e-06 | 0.171449578627116 | 0.069 | 0.056 | 0.154151220038132 |
| CLP1 | 6.27205987335064e-06 | 0.112910077816482 | 0.06 | 0.047 | 0.154681540596573 |
| CA5B | 6.66614993037203e-06 | 0.138211500049845 | 0.087 | 0.072 | 0.164400589582835 |
| TIPARP | 8.10070247716892e-06 | 0.136579108445782 | 0.088 | 0.073 | 0.19977952449194 |
| PGM2 | 9.36614629629678e-06 | 0.217495693636934 | 0.059 | 0.048 | 0.230987899959271 |
| CYTH2 | 9.45199538931461e-06 | 0.105496580547272 | 0.053 | 0.042 | 0.233105110291277 |
| RPS6KB2 | 1.15215792558511e-05 | 0.129825627444133 | 0.055 | 0.044 | 0.2841451876078 |
| AGPAT2 | 1.22040146041319e-05 | 0.158306213308242 | 0.093 | 0.079 | 0.3009754081671 |
| SCAMP2 | 1.27077780469676e-05 | 0.104094271653287 | 0.099 | 0.083 | 0.313399222194315 |
| PRKDC | 2.19503414010141e-05 | 0.107538628387484 | 0.076 | 0.063 | 0.541339319631811 |
| IRF2BPL | 2.42040629207928e-05 | 0.115480773203524 | 0.068 | 0.055 | 0.596920599752592 |
| TMEM63A | 2.45745936081259e-05 | 0.153428822103757 | 0.051 | 0.041 | 0.606058627563601 |
| PGP | 2.49928716263848e-05 | 0.174226071371186 | 0.081 | 0.068 | 0.616374200049902 |
| SHARPIN | 3.28841302014301e-05 | 0.10792575455455 | 0.063 | 0.052 | 0.810988419027668 |
| INTS3 | 3.51048415878009e-05 | 0.193111966515467 | 0.053 | 0.042 | 0.865755603238345 |
| KIAA0930 | 3.5706585636507e-05 | 0.105875675131424 | 0.09 | 0.076 | 0.880595814967534 |
| C18orf21 | 5.91703710238623e-05 | 0.195067355424833 | 0.059 | 0.048 | 1 |

| Gene | P_val | Avg_log2FC | Pct.1 | Pct.2 | P_val_adj |
|---|---|---|---|---|---|
| METTL23 | 6.8841111904632e-05 | 0.117600087433205 | 0.051 | 0.041 | 1 |
| PHF23 | 7.53974977921268e-05 | 0.13420185337794 | 0.056 | 0.046 | 1 |
| LSM6 | 0.000108483641833143 | 0.171580436465335 | 0.061 | 0.05 | 1 |
| MINDY2 | 0.000800589172677951 | 0.127444069346592 | 0.054 | 0.045 | 1 |
| SPTSSA | 0.00100152943338789 | 0.105443102947546 | 0.068 | 0.059 | 1 |
| CITED4 | 0.00111992080340764 | 0.142478780627395 | 0.112 | 0.1 | 1 |
| TMUB1 | 0.021785049546535 | 0.101247034601154 | 0.05 | 0.044 | 1 |

Supplementary Table S5：Differentially expressed genes in T cells between NET-high and NET-low groups

| Gene | P_val | Avg_log2FC | Pct.1 | Pct.2 | P_val_adj |
|---|---|---|---|---|---|
| MALAT1 | 0 | 0.361707972278855 | 0.997 | 0.996 | 0 |
| S100A12 | 1.44390445167072e-157 | 1.93193022397184 | 0.211 | 0.138 | 3.56095715871032e-153 |
| HBB | 2.54621903955671e-139 | 0.731741899957218 | 0.715 | 0.668 | 6.27948539535477e-135 |
| S100A9 | 2.02360673931177e-129 | 1.46365468335494 | 0.509 | 0.473 | 4.99061894049069e-125 |
| S100A8 | 5.26595719189331e-124 | 1.64820997666129 | 0.466 | 0.426 | 1.29869036266473e-119 |
| FTH1 | 1.53072977841224e-120 | 0.491240901080258 | 0.894 | 0.913 | 3.77508577952028e-116 |
| HBA2 | 5.10100341342479e-101 | 0.687926586867529 | 0.627 | 0.597 | 1.25800946181882e-96 |
| HBA1 | 8.85364221365686e-96 | 0.702435719428278 | 0.583 | 0.552 | 2.18348524273205e-91 |
| DUSP1 | 9.71182637358177e-85 | 0.907041864377868 | 0.327 | 0.281 | 2.39513062025274e-80 |
| FPR1 | 1.12374230433145e-82 | 1.4234915550104 | 0.13 | 0.085 | 2.77137327094223e-78 |
| SERPINB1 | 7.69154936980941e-77 | 0.105079853233751 | 0.263 | 0.362 | 1.8968899055824e-72 |
| SERPINA1 | 1.09252748173179e-76 | 1.31056254324606 | 0.146 | 0.101 | 2.69439127544694e-72 |
| NAMPT | 1.41696726687631e-73 | 1.12087359921441 | 0.251 | 0.207 | 3.49452467357035e-69 |
| CD14 | 4.48199282772286e-72 | 1.38532582363565 | 0.116 | 0.076 | 1.10534907117301e-67 |
| MNDA | 2.3734067093005e-69 | 1.26515473581774 | 0.16 | 0.117 | 5.85329562647689e-65 |
| SAT1 | 1.14774418558136e-67 | 0.734495416300592 | 0.511 | 0.516 | 2.83056671048076e-63 |
| SLC11A1 | 3.44559006756689e-67 | 1.23944357796096 | 0.091 | 0.055 | 8.49751422463346e-63 |
| IL7R | 1.44390834123935e-64 | 1.02332933731241 | 0.111 | 0.072 | 3.56096675116448e-60 |
| TCF7 | 1.316353298338e-61 | 1.1279465233277 | 0.12 | 0.082 | 3.24639050436117e-57 |
| CSF3R | 2.08269068639259e-59 | 1.24025626146322 | 0.127 | 0.089 | 5.13633177078141e-55 |
| HBD | 1.17129272465647e-58 | 0.965836526768672 | 0.106 | 0.069 | 2.88864211754778e-54 |
| SPI1 | 1.80319854140772e-58 | 0.991995049210642 | 0.141 | 0.102 | 4.44704824281972e-54 |
| NCOA4 | 7.67464008190543e-58 | 0.121427386859112 | 0.178 | 0.248 | 1.89271973699952e-53 |
| CXCL8 | 2.5313386217098e-57 | 1.84742791254973 | 0.054 | 0.029 | 6.2427873088607e-53 |
| SUCLA2 | 6.98595854585356e-57 | 0.117632452701287 | 0.063 | 0.102 | 1.72287709657841e-52 |
| LAMP2 | 3.60514482056206e-55 | 0.108265210743389 | 0.136 | 0.194 | 8.89100815647016e-51 |
| LAP3 | 7.54202105281189e-55 | 0.224008729616829 | 0.158 | 0.222 | 1.86001323204447e-50 |
| VCAN | 1.74926795080698e-52 | 1.18052495043494 | 0.139 | 0.102 | 4.31404462028017e-48 |
| PLP2 | 8.65984501605746e-52 | 0.206565311358438 | 0.109 | 0.159 | 2.13569097786009e-47 |
| TUBA1A | 3.15332889841399e-51 | 0.171405615476751 | 0.177 | 0.242 | 7.77673972926859e-47 |
| JUNB | 1.28486048204397e-50 | 0.515024233952875 | 0.499 | 0.502 | 3.16872292081685e-46 |
| G0S2 | 2.67813392370598e-50 | 1.51148654642075 | 0.085 | 0.055 | 6.60481388264369e-46 |

| Gene | P_val | Avg_log2FC | Pct. 1 | Pct. 2 | P_val_adj |
|---|---|---|---|---|---|
| FCN1 | 3.04592573920761e-50 | 1.0114126211556 | 0.197 | 0.16 | 7.51186205803382e-46 |
| FOS | 3.30612295510952e-50 | 1.06730830979014 | 0.218 | 0.183 | 8.1535604318911e-46 |
| LYZ | 7.36441165903769e-50 | 0.91252351297188 | 0.299 | 0.266 | 1.81621120335188e-45 |
| IFI44L | 2.99251056351258e-49 | 1.20201687695234 | 0.077 | 0.048 | 7.38012955173473e-45 |
| HEXB | 4.79858512457178e-49 | 0.132582581551462 | 0.108 | 0.156 | 1.18342706342189e-44 |
| RTN3 | 2.35584793170442e-48 | 0.234704502720177 | 0.148 | 0.206 | 5.80999216916944e-44 |
| GABARAP | 3.14699285714225e-47 | 0.117118657294353 | 0.134 | 0.186 | 7.76111378428421e-43 |
| IFNAR1 | 3.96633014309109e-47 | 0.11938825793633 | 0.111 | 0.158 | 9.78176339889125e-43 |
| NFYC | 1.65912478659301e-46 | 0.103590552771837 | 0.078 | 0.117 | 4.09173354869568e-42 |
| PDLIM7 | 2.67796661759174e-46 | 0.111043948386976 | 0.026 | 0.05 | 6.60440127230474e-42 |
| BASP1 | 5.347437754758e-46 | 1.67474222900477 | 0.054 | 0.031 | 1.31878509907842e-41 |
| ZFAS1 | 1.11918388519281e-45 | 0.118478650060923 | 0.183 | 0.246 | 2.7601312976625e-41 |
| AGPS | 1.95414207197739e-45 | 0.109839709188222 | 0.071 | 0.108 | 4.81930517791064e-41 |
| CAPZA2 | 3.15005034737678e-45 | 0.156919939980315 | 0.171 | 0.232 | 7.76865416670062e-41 |
| PPT1 | 3.69820042351227e-45 | 0.323134985372815 | 0.109 | 0.156 | 9.12050188446597e-41 |
| GTF2F2 | 4.06371576391477e-45 | 0.104954114886924 | 0.07 | 0.107 | 1.00219358169666e-40 |
| METTL9 | 9.15640788249205e-45 | 0.201143727778286 | 0.172 | 0.234 | 2.25815331198019e-40 |
| DNTTIP1 | 1.04264073884912e-44 | 0.116677999573804 | 0.047 | 0.077 | 2.57136059014969e-40 |
| LILRB2 | 2.79366692033853e-44 | 1.06892688048818 | 0.073 | 0.046 | 6.88974135893888e-40 |
| ADSS2 | 1.42465728431558e-43 | 0.105440570729127 | 0.145 | 0.198 | 3.51348979457907e-39 |
| TLR2 | 2.03885318039847e-43 | 1.28979149051235 | 0.052 | 0.03 | 5.0282197134987e-39 |
| CA1 | 3.69966886299945e-43 | 0.986121240599902 | 0.088 | 0.06 | 9.12412334992924e-39 |
| PLEKHB2 | 4.81444696486982e-42 | 0.107126242030938 | 0.117 | 0.163 | 1.1873389104762e-37 |
| LGALS3 | 2.62197599718028e-41 | 0.194659296020822 | 0.127 | 0.175 | 6.46631720424601e-37 |
| SEC23B | 4.34130786870245e-40 | 0.130237064324769 | 0.081 | 0.118 | 1.0706533465794e-35 |
| SLC8A1 | 5.35218916832945e-40 | 0.954661964753915 | 0.059 | 0.036 | 1.31995689269341e-35 |
| ILKAP | 5.85095121501576e-40 | 0.104476671600581 | 0.106 | 0.149 | 1.44296158864719e-35 |
| C5AR1 | 6.56699577955115e-40 | 1.25846670484789 | 0.059 | 0.036 | 1.6195524991529e-35 |
| DPYD | 9.01646924246375e-40 | 0.861834152870292 | 0.181 | 0.148 | 2.22364164457641e-35 |
| PTTG1IP | 1.55212362489115e-39 | 0.166615700071809 | 0.096 | 0.136 | 3.82784728370656e-35 |
| NT5C3A | 1.64492015025279e-39 | 0.132658910977224 | 0.166 | 0.221 | 4.05670207455342e-35 |
| NQO2 | 1.88111253164869e-39 | 0.103331325215349 | 0.049 | 0.077 | 4.639199725552e-35 |

| Gene | P_val | Avg_log2FC | Pct.1 | Pct.2 | P_val_adj |
|---|---|---|---|---|---|
| FOXN2 | 1.95994034766041e-39 | 0.106251855475145 | 0.117 | 0.161 | 4.8336048854001e-35 |
| KRAS | 2.71917477735165e-39 | 0.123471901813832 | 0.146 | 0.197 | 6.70602883590463e-35 |
| NUP88 | 4.63707004012044e-39 | 0.177024444784039 | 0.074 | 0.109 | 1.1435942132945e-34 |
| IFNGR2 | 6.19910431408959e-39 | 1.07996447565581 | 0.069 | 0.045 | 1.52882310594078e-34 |
| DLEU2 | 7.42248042859777e-39 | 0.304387728099652 | 0.124 | 0.171 | 1.83053212330078e-34 |
| CLEC7A | 8.10886812765206e-39 | 1.20974712527063 | 0.059 | 0.036 | 1.99980905764155e-34 |
| R3HDM1 | 9.32764464484778e-39 | 0.118353753705054 | 0.099 | 0.139 | 2.30038372231236e-34 |
| TNFSF13B | 1.38097875764944e-38 | 1.30010736020072 | 0.069 | 0.045 | 3.40576981211505e-34 |
| WDR33 | 1.50810526147726e-38 | 0.13263802946571 | 0.146 | 0.197 | 3.71928919585522e-34 |
| SEC63 | 1.79378343887773e-38 | 0.148609991065987 | 0.124 | 0.17 | 4.42382871696026e-34 |
| CYBB | 1.97065622311099e-38 | 1.10837467525727 | 0.089 | 0.062 | 4.86003237743633e-34 |
| BLVRA | 2.09334341190649e-38 | 0.330661795725872 | 0.087 | 0.125 | 5.16260352244377e-34 |
| LPGAT1 | 2.43078520091144e-38 | 0.121317120415466 | 0.15 | 0.2 | 5.99480246248779e-34 |
| RARS1 | 3.20621710431164e-38 | 0.117060133230873 | 0.069 | 0.102 | 7.90717262265336e-34 |
| COA1 | 4.96578114182539e-38 | 0.163109898502557 | 0.086 | 0.123 | 1.22466094519698e-33 |
| MAP2K3 | 5.34819930120277e-38 | 0.128145569047101 | 0.125 | 0.171 | 1.31897291166263e-33 |
| PREP | 7.94476256522325e-38 | 0.125517748463871 | 0.07 | 0.102 | 1.95933734383536e-33 |
| ELOVL5 | 1.14501509995127e-37 | 0.132342934350712 | 0.167 | 0.222 | 2.82383623949983e-33 |
| RBMS1 | 1.39218713540276e-37 | 0.135177171336545 | 0.205 | 0.267 | 3.4334119133303e-33 |
| STK39 | 1.89317549725831e-37 | 0.151472388225601 | 0.132 | 0.179 | 4.66894941133845e-33 |
| S100A11 | 3.10881592333134e-37 | 0.18794043671555 | 0.495 | 0.596 | 7.66696183011975e-33 |
| BNIP2 | 3.27001099016163e-37 | 0.129817018877753 | 0.21 | 0.275 | 8.06450110393662e-33 |
| IDI1 | 4.39884705424208e-37 | 0.146222870701068 | 0.179 | 0.236 | 1.08484366051718e-32 |
| OGDH | 7.03519331710881e-37 | 0.104931712817468 | 0.183 | 0.239 | 1.73501937586538e-32 |
| FCGR2A | 8.43490682728139e-37 | 1.37011473822291 | 0.078 | 0.053 | 2.08021672174414e-32 |
| DDX56 | 9.25465183049059e-37 | 0.13791044602975 | 0.057 | 0.087 | 2.28238223443559e-32 |
| PARP9 | 9.49357619337994e-37 | 0.13594615233188 | 0.2 | 0.261 | 2.34130576081136e-32 |
| ASXL1 | 1.22644508745437e-36 | 0.125526981957209 | 0.155 | 0.207 | 3.02465887467997e-32 |
| ZNF326 | 1.23912885101552e-36 | 0.130927770061031 | 0.12 | 0.163 | 3.05593957237446e-32 |
| GTF2H1 | 1.24336683916349e-36 | 0.100915936427463 | 0.075 | 0.108 | 3.06639129874499e-32 |
| STX11 | 2.01423458178414e-36 | 0.352214102823079 | 0.085 | 0.122 | 4.96750532559604e-32 |
| GAB2 | 2.1119293077377e-36 | 0.793407236488706 | 0.055 | 0.034 | 5.20844005874272e-32 |

| Gene | P_val | Avg_log2FC | Pct. 1 | Pct. 2 | P_val_adj |
|---|---|---|---|---|---|
| MS4A6A | 2.30795640188767e-36 | 1.29379815210291 | 0.069 | 0.046 | 5.69188207833537e-32 |
| DNAJC1 | 2.609303370889e-36 | 0.105564674088661 | 0.189 | 0.247 | 6.43506397328646e-32 |
| TAF1D | 3.09708243335106e-36 | 0.130668809647405 | 0.166 | 0.219 | 7.63802469713039e-32 |
| MYADM | 3.49379099596826e-36 | 0.193204723458158 | 0.094 | 0.131 | 8.61638735425692e-32 |
| CTDSPL2 | 4.2068584810271e-36 | 0.13410535023405 | 0.089 | 0.126 | 1.0374954385909e-31 |
| FCER1G | 4.50603671863519e-36 | 1.02399606218849 | 0.192 | 0.164 | 1.11127877554981e-31 |
| NAA50 | 7.94108251651643e-36 | 0.103574422102357 | 0.206 | 0.268 | 1.95842977022328e-31 |
| GCH1 | 9.84264088469575e-36 | 0.13121237608886 | 0.128 | 0.173 | 2.42739209498367e-31 |
| HCK | 9.92252449805846e-36 | 0.964367540971366 | 0.087 | 0.061 | 2.44709299171118e-31 |
| PLXDC2 | 1.27247470108765e-35 | 0.94384876532083 | 0.059 | 0.037 | 3.13817710782237e-31 |
| FAM133B | 1.3130835011806e-35 | 0.163736055183435 | 0.136 | 0.183 | 3.2383265306116e-31 |
| AP1S2 | 1.59771780292865e-35 | 0.277388240634055 | 0.125 | 0.169 | 3.94029164558264e-31 |
| XRCC4 | 2.16746040885875e-35 | 0.128355946661798 | 0.035 | 0.058 | 5.34539086032744e-31 |
| TACC1 | 2.68248620145458e-35 | 0.134635416525838 | 0.17 | 0.224 | 6.61554747002728e-31 |
| CRNKL1 | 2.94451442437699e-35 | 0.113142180236409 | 0.088 | 0.124 | 7.26176147339854e-31 |
| FLOT1 | 4.69038739095923e-35 | 0.128911376946101 | 0.13 | 0.174 | 1.15674333835837e-30 |
| GALM | 4.71613725937344e-35 | 0.109539710418659 | 0.117 | 0.159 | 1.16309377090668e-30 |
| FCGR3B | 4.85885050569576e-35 | 1.49937142287237 | 0.082 | 0.058 | 1.19828971171469e-30 |
| JAML | 4.86915626215052e-35 | 0.943462841297026 | 0.09 | 0.064 | 1.20083131737156e-30 |
| ETF1 | 5.32468820255833e-35 | 0.201359948619697 | 0.121 | 0.164 | 1.31317460451494e-30 |
| CEP135 | 6.30045578235396e-35 | 0.107436357108166 | 0.058 | 0.087 | 1.55381840504413e-30 |
| NEAT1 | 9.14526048130855e-35 | 0.399087950713767 | 0.489 | 0.496 | 2.25540413990031e-30 |
| DCUN1D1 | 1.09674805591247e-34 | 0.10335453574832 | 0.061 | 0.09 | 2.70480005549133e-30 |
| CNIH4 | 1.28133053506372e-34 | 0.237040112903734 | 0.075 | 0.108 | 3.16001736557416e-30 |
| RB1 | 1.76071308270122e-34 | 0.167397889486381 | 0.136 | 0.182 | 4.34227060455775e-30 |
| CHP1 | 2.12775791459932e-34 | 0.125051060293631 | 0.081 | 0.114 | 5.24747656898484e-30 |
| USP10 | 2.36170481525847e-34 | 0.223908123759914 | 0.101 | 0.14 | 5.82443641539043e-30 |
| NOL10 | 2.38372049119633e-34 | 0.119562945180798 | 0.067 | 0.097 | 5.8787314753884e-30 |
| CYB5R4 | 2.77679509749622e-34 | 0.208221576887498 | 0.121 | 0.163 | 6.84813206944517e-30 |
| MORF4L2 | 4.83640939248383e-34 | 0.138045303846335 | 0.109 | 0.148 | 1.19275528437436e-29 |
| RIF1 | 4.91664384767985e-34 | 0.146193172967631 | 0.096 | 0.133 | 1.21254270571481e-29 |
| ACSL4 | 4.94757689919899e-34 | 0.177038130385312 | 0.099 | 0.137 | 1.22017141488046e-29 |

| Gene | P_val | Avg_log2FC | Pct.1 | Pct.2 | P_val_adj |
|---|---|---|---|---|---|
| SPTLC1 | 6.84063458708981e-34 | 0.10195095161068 | 0.088 | 0.123 | 1.68703730186809e-29 |
| TMEM33 | 6.86764121582095e-34 | 0.161621227168692 | 0.06 | 0.089 | 1.69369767664576e-29 |
| TNPO3 | 7.57991990786952e-34 | 0.135074209451735 | 0.083 | 0.117 | 1.86935984767878e-29 |
| COMMD1 | 8.41504164297467e-34 | 0.113973320588527 | 0.099 | 0.136 | 2.07531756999041e-29 |
| NF2 | 8.68407585515857e-34 | 0.102773254110132 | 0.05 | 0.076 | 2.14166678739921e-29 |
| PPP4R3B | 1.01711158269195e-33 | 0.100709494636996 | 0.195 | 0.252 | 2.50840058523488e-29 |
| STXBP2 | 1.08748184239037e-33 | 0.116578614150492 | 0.2 | 0.256 | 2.68194771970312e-29 |
| CUL4A | 1.50628991326098e-33 | 0.117307955513811 | 0.152 | 0.2 | 3.71481218408422e-29 |
| MSL3 | 1.67318281291708e-33 | 0.117413575480855 | 0.135 | 0.18 | 4.1264034532161e-29 |
| SLC38A2 | 1.98765069979648e-33 | 0.165775491109579 | 0.117 | 0.158 | 4.90194415583808e-29 |
| INSIG1 | 2.09914618522174e-33 | 0.129934860921637 | 0.123 | 0.164 | 5.17691432199385e-29 |
| NAGA | 2.17617520001114e-33 | 0.193324372963263 | 0.032 | 0.053 | 5.36688327826748e-29 |
| UPF2 | 3.4987884556688e-33 | 0.174258823359069 | 0.133 | 0.177 | 8.62871208937039e-29 |
| PLBD1 | 4.04158550346114e-33 | 1.43037166341075 | 0.057 | 0.037 | 9.96735816863588e-29 |
| DYSF | 4.32939568298429e-33 | 0.998304041771791 | 0.055 | 0.035 | 1.06771556333759e-28 |
| RTN4 | 4.55643349906766e-33 | 0.191572788272034 | 0.236 | 0.303 | 1.12370762954007e-28 |
| CCDC6 | 4.63488756303304e-33 | 0.102898445498246 | 0.073 | 0.104 | 1.14305597079521e-28 |
| SNAP23 | 4.83134684560175e-33 | 0.157447587638849 | 0.121 | 0.162 | 1.1915067590623e-28 |
| OAS1 | 6.16627560718455e-33 | 0.132209173912165 | 0.101 | 0.137 | 1.52072689024385e-28 |
| MFSD6 | 6.32974272709984e-33 | 0.166920636464086 | 0.092 | 0.127 | 1.56104115135736e-28 |
| N4BP2 | 7.90509477202096e-33 | 0.102701057657211 | 0.089 | 0.123 | 1.94955447267581e-28 |
| GALNT1 | 1.07025708462469e-32 | 0.118733226372738 | 0.135 | 0.178 | 2.63946802210141e-28 |
| PPP1R15B | 1.18899884256278e-32 | 0.108669158133649 | 0.094 | 0.129 | 2.93230894552834e-28 |
| FTL | 1.6885756809805e-32 | 0.439523838509173 | 0.888 | 0.914 | 4.16436534443412e-28 |
| THEMIS | 1.77045346513838e-32 | 0.139885431597533 | 0.287 | 0.363 | 4.36629233572428e-28 |
| OASL | 1.99054039633619e-32 | 0.180418464877564 | 0.101 | 0.138 | 4.90907072544431e-28 |
| USP48 | 3.09924500808814e-32 | 0.171158518197843 | 0.132 | 0.176 | 7.64335803894697e-28 |
| CARS2 | 4.43389623945445e-32 | 0.147955511926595 | 0.086 | 0.12 | 1.09348749057426e-27 |
| PDCD6IP | 5.51336653517676e-32 | 0.118187523404587 | 0.17 | 0.221 | 1.35970645490529e-27 |
| SMCHD1 | 5.89737759214898e-32 | 0.448678517903799 | 0.51 | 0.534 | 1.45441126177578e-27 |
| TNPO1 | 5.90507655323973e-32 | 0.196622864344532 | 0.082 | 0.116 | 1.45630997955998e-27 |
| IRAK3 | 6.59473525145574e-32 | 1.01263805187579 | 0.054 | 0.034 | 1.62639360771402e-27 |

| Gene | P_val | Avg_log2FC | Pct. 1 | Pct. 2 | P_val_adj |
|---|---|---|---|---|---|
| CTSA | 7.01966560677612e-32 | 0.240313102971544 | 0.135 | 0.179 | 1.73118993194313e-27 |
| GNA13 | 1.59809602223412e-31 | 0.147650689907184 | 0.134 | 0.178 | 3.94122441003377e-27 |
| IFI6 | 2.60115980776552e-31 | 0.35600174035896 | 0.207 | 0.268 | 6.41498031791133e-27 |
| ST8SIA4 | 3.8147618409113e-31 | 0.191199148498121 | 0.128 | 0.169 | 9.40796565205546e-27 |
| PPP3CB | 4.08650644766516e-31 | 0.111080586393642 | 0.111 | 0.15 | 1.00781422012318e-26 |
| ZFR | 4.86651679009568e-31 | 0.142979048190218 | 0.2 | 0.257 | 1.2001803707734e-26 |
| ATP6V1B2 | 5.07573518503446e-31 | 0.169186925395302 | 0.08 | 0.111 | 1.2517778113332e-26 |
| RAB5A | 5.13538877454641e-31 | 0.131510715277285 | 0.123 | 0.163 | 1.26648957957864e-26 |
| OAZ2 | 6.45812946189925e-31 | 0.139365962711213 | 0.066 | 0.094 | 1.59270388789359e-26 |
| DMXL2 | 7.96573423444625e-31 | 0.977546591310154 | 0.056 | 0.036 | 1.96450937689914e-26 |
| TM9SF3 | 9.29414012019958e-31 | 0.130478375848718 | 0.18 | 0.232 | 2.29212083644362e-26 |
| PTPN22 | 1.11336956703032e-30 | 0.126178071288688 | 0.221 | 0.281 | 2.7457920262101 8e-26 |
| RAB31 | 1.16506038715054e-30 | 0.974366521547028 | 0.067 | 0.046 | 2.87327192679066e-26 |
| SHLD2 | 1.63042406182674e-30 | 0.119663061160676 | 0.073 | 0.103 | 4.02095182127712e-26 |
| NUP58 | 2.41011477966681e-30 | 0.123034924703641 | 0.055 | 0.081 | 5.94382506961429e-26 |
| LAPTM4A | 2.69928425723416e-30 | 0.198661920859377 | 0.169 | 0.219 | 6.6569748351909e-26 |
| NUP153 | 2.73788870037115e-30 | 0.16697302726937 | 0.109 | 0.147 | 6.75218111285533e-26 |
| ALOX5 | 3.03565058832688e-30 | 1.07466439623719 | 0.06 | 0.04 | 7.48652148093176e-26 |
| PAFAH1B1 | 3.73456352192513e-30 | 0.109737045421302 | 0.25 | 0.317 | 9.21018055777175e-26 |
| GABPB1 | 5.24520750674473e-30 | 0.156655869197265 | 0.064 | 0.091 | 1.29357307531339e-25 |
| CHMP2B | 5.97731261448745e-30 | 0.137600075642784 | 0.098 | 0.132 | 1.47412483698489e-25 |
| BTBD9 | 6.52789723163726e-30 | 0.823023648897814 | 0.068 | 0.047 | 1.60991001526638e-25 |
| SLC25A37 | 6.91676802324316e-30 | 1.01320326121523 | 0.132 | 0.107 | 1.70581332989223e-25 |
| NCF2 | 9.96903139275065e-30 | 0.936831859913218 | 0.08 | 0.057 | 2.45856252208017e-25 |
| PPP1CB | 1.11726573676444e-29 | 0.269306666210845 | 0.122 | 0.163 | 2.75540076000845e-25 |
| LGALS8 | 1.14718540646165e-29 | 0.104699479962927 | 0.136 | 0.177 | 2.82918864941571e-25 |
| MAML2 | 1.19867441298959e-29 | 0.959942808136157 | 0.066 | 0.045 | 2.95617083731492e-25 |
| PPP4R3A | 1.34693872181336e-29 | 0.100192030269085 | 0.197 | 0.252 | 3.3218202757361e-25 |
| ZFAND6 | 1.66451939412102e-29 | 0.161630209914261 | 0.19 | 0.244 | 4.10503772978127e-25 |
| OSBPL11 | 1.69674171427308e-29 | 0.106897659547752 | 0.053 | 0.077 | 4.18450441574026e-25 |
| YME1L1 | 2.09736807706223e-29 | 0.1181483549417 | 0.25 | 0.316 | 5.17252915165086e-25 |
| NUP54 | 2.36880875436556e-29 | 0.101019548877111 | 0.081 | 0.113 | 5.84195615001635e-25 |

| Gene | P_val | Avg_log2FC | Pct. 1 | Pct. 2 | P_val_adj |
|---|---|---|---|---|---|
| LARP1 | 2.84549386886158e-29 | 0.121055567058789 | 0.168 | 0.215 | 7.01755697938643e-25 |
| DIS3L | 2.89310680751929e-29 | 0.111215953334072 | 0.064 | 0.091 | 7.13498000870408e-25 |
| PIK3CG | 3.87434370135693e-29 | 0.105935083827948 | 0.083 | 0.114 | 9.55490643628646e-25 |
| HBG2 | 4.7924480220212e-29 | 0.910785540689977 | 0.068 | 0.047 | 1.18191353119087e-24 |
| RDX | 5.10612161347796e-29 | 0.117819577738693 | 0.053 | 0.078 | 1.25927171231594e-24 |
| PRIM2 | 5.23993217592595e-29 | 0.323562789394418 | 0.039 | 0.061 | 1.29227207322686e-24 |
| GLIPR1 | 9.39256512132082e-29 | 0.136331878257493 | 0.229 | 0.288 | 2.31639441022014e-24 |
| LRRK2 | 1.21985213276138e-28 | 0.912899804179121 | 0.072 | 0.05 | 3.00839932981611e-24 |
| FAM200B | 1.23928164148997e-28 | 0.107732721535749 | 0.07 | 0.098 | 3.05631638424256e-24 |
| TRADD | 1.26087299764467e-28 | 0.130723617010546 | 0.116 | 0.154 | 3.10956498679128e-24 |
| RNF145 | 1.30009834302472e-28 | 0.173782402662495 | 0.154 | 0.2 | 3.20630253356757e-24 |
| RAP1GDS1 | 1.30687451575355e-28 | 0.161730869588949 | 0.111 | 0.147 | 3.2230139307514e-24 |
| RAB10 | 1.33806612386012e-28 | 0.253377786314156 | 0.154 | 0.2 | 3.29993867466384e-24 |
| CWF19L1 | 1.39569543017782e-28 | 0.109573265813619 | 0.076 | 0.106 | 3.44206406990453e-24 |
| SMAD2 | 1.40987571135643e-28 | 0.118722132309561 | 0.122 | 0.161 | 3.47703547934724e-24 |
| RAP1A | 1.54622192557401e-28 | 0.160451689722027 | 0.277 | 0.348 | 3.81329251285062e-24 |
| ABHD3 | 1.64318698455131e-28 | 0.150395695956739 | 0.101 | 0.137 | 4.05242774130044e-24 |
| RAB7A | 1.66308241635983e-28 | 0.162632923434214 | 0.302 | 0.381 | 4.10149385522661e-24 |
| RPS6KB1 | 1.67076280081102e-28 | 0.131405902681859 | 0.07 | 0.098 | 4.12043521936015e-24 |
| CDC40 | 2.15081703863257e-28 | 0.136578375249386 | 0.133 | 0.173 | 5.30434498067564e-24 |
| EIF4A1 | 2.49811571437156e-28 | 0.137169708537637 | 0.077 | 0.107 | 6.16085297478314e-24 |
| KPNA4 | 2.58763801163863e-28 | 0.129631326188856 | 0.121 | 0.159 | 6.3816328643032e-24 |
| JPX | 2.84120504393458e-28 | 0.110851768719595 | 0.089 | 0.121 | 7.00697987935146e-24 |
| AL159141.1 | 2.94706770483444e-28 | 0.179924518535724 | 0.165 | 0.211 | 7.26805837366269e-24 |
| PCM1 | 2.96614903584724e-28 | 0.128721456681518 | 0.236 | 0.298 | 7.31511675220645e-24 |
| FECH | 3.19470494559228e-28 | 0.135404211376973 | 0.033 | 0.052 | 7.87878133681968e-24 |
| STAU1 | 3.44338674327587e-28 | 0.178394825963273 | 0.142 | 0.185 | 8.49208038626695e-24 |
| WBP4 | 3.755544220257e-28 | 0.147845113570391 | 0.035 | 0.055 | 9.26192315599782e-24 |
| CITED2 | 4.6389232262055e-28 | 0.107037950667607 | 0.123 | 0.161 | 1.1440512460468e-23 |
| CUL1 | 4.67962843413738e-28 | 0.127516105644907 | 0.14 | 0.182 | 1.15408996442696e-23 |
| FAM76B | 5.06256525686209e-28 | 0.157317750907405 | 0.117 | 0.154 | 1.24852984364733e-23 |
| SNX18 | 5.17482701497595e-28 | 0.167743895274308 | 0.052 | 0.075 | 1.27621583843337e-23 |

| Gene | P_val | Avg_log2FC | Pct. 1 | Pct. 2 | P_val_adj |
|---|---|---|---|---|---|
| UHRF2 | 6.14758793314766e-28 | 0.111891745820034 | 0.14 | 0.182 | 1.51611813607288e-23 |
| LIMS1 | 6.39137076974727e-28 | 0.154909593972744 | 0.159 | 0.205 | 1.57623985923507e-23 |
| NPTN | 7.60091148484996e-28 | 0.167432531888373 | 0.092 | 0.125 | 1.8745367903937e-23 |
| RNF10 | 7.93511179436668e-28 | 0.129543561516638 | 0.227 | 0.286 | 1.95695727072671e-23 |
| OXSR1 | 1.01419977521797e-27 | 0.14731908844263 | 0.097 | 0.13 | 2.50121948564255e-23 |
| PRMT3 | 1.03417236325585e-27 | 0.174558657594354 | 0.036 | 0.056 | 2.55047588226158e-23 |
| YIPF4 | 1.05040584140676e-27 | 0.192695449000535 | 0.067 | 0.094 | 2.59051088607736e-23 |
| CHUK | 1.16034909491481e-27 | 0.129236240108969 | 0.058 | 0.083 | 2.86165293787889e-23 |
| GCA | 1.18593137091094e-27 | 1.47763909667012 | 0.071 | 0.051 | 2.92474394694055e-23 |
| PDE4B | 1.36620652548995e-27 | 0.806617933724093 | 0.108 | 0.084 | 3.36933853316331e-23 |
| TSG101 | 1.56708377953146e-27 | 0.25479290682359 | 0.119 | 0.157 | 3.86474201708048e-23 |
| MCPH1 | 1.6615283090729e-27 | 0.104501428359622 | 0.105 | 0.139 | 4.09766111583558e-23 |
| RANBP9 | 1.77675722157273e-27 | 0.14591489349226 | 0.119 | 0.156 | 4.38183865984266e-23 |
| PRKACA | 1.82863804875973e-27 | 0.10646192517164 | 0.074 | 0.103 | 4.50978715585126e-23 |
| NGLY1 | 2.0983322580783e-27 | 0.115423101540064 | 0.106 | 0.141 | 5.1749070148727e-23 |
| CD36 | 3.13539352200733e-27 | 1.07066781684469 | 0.061 | 0.042 | 7.73250750397447e-23 |
| SPPL2A | 3.45709922292973e-27 | 0.201417997054745 | 0.159 | 0.205 | 8.52589810358929e-23 |
| FAM177A1 | 3.68058014578809e-27 | 0.165217367985109 | 0.109 | 0.144 | 9.07704675554259e-23 |
| BRD9 | 4.64388202726674e-27 | 0.116815518815821 | 0.088 | 0.119 | 1.14527418556452e-22 |
| SP140L | 5.71973431238922e-27 | 0.139870280887362 | 0.177 | 0.225 | 1.41060087612143e-22 |
| PLAUR | 6.10688802582945e-27 | 0.85284270591195 | 0.058 | 0.039 | 1.50608072493006e-22 |
| NCF1 | 7.1502583645949e-27 | 0.941962775868823 | 0.153 | 0.128 | 1.76339671787639e-22 |
| CAAP1 | 7.4159587660013e-27 | 0.123526774021709 | 0.049 | 0.071 | 1.82892375087124e-22 |
| YWHAG | 8.35703206357274e-27 | 0.271700467296552 | 0.062 | 0.088 | 2.06101124751831e-22 |
| ABCB10 | 1.0241058651713e-26 | 0.139776594780343 | 0.04 | 0.06 | 2.52564988468546e-22 |
| PHF10 | 1.27814172720988e-26 | 0.114806434039712 | 0.076 | 0.104 | 3.15215312764501e-22 |
| NAIP | 1.37858283581973e-26 | 0.999638456796958 | 0.099 | 0.077 | 3.39986098969861e-22 |
| SEC62 | 1.58385969736891e-26 | 0.166317016987548 | 0.204 | 0.258 | 3.9061147856512e-22 |
| LDLR | 1.91763048831071e-26 | 0.111136977768544 | 0.068 | 0.094 | 4.72926031027186e-22 |
| EIF4G3 | 2.01416744285479e-26 | 0.139608017987871 | 0.215 | 0.269 | 4.96733974756849e-22 |
| COLGALT1 | 2.14662817751021e-26 | 0.175830820821799 | 0.046 | 0.068 | 5.29401441137568e-22 |
| TPD52L2 | 2.18551485417488e-26 | 0.201699573851263 | 0.059 | 0.084 | 5.38991673336608e-22 |

| Gene | P_val | Avg_log2FC | Pct.1 | Pct.2 | P_val_adj |
|---|---|---|---|---|---|
| RAB2A | 2.8321993926426e-26 | 0.196958098662491 | 0.236 | 0.297 | 6.98477014213517e-22 |
| CAB39 | 3.05484585600288e-26 | 0.120229177437808 | 0.205 | 0.259 | 7.5338608500743e-22 |
| UBE3C | 3.11501858860683e-26 | 0.169994600759991 | 0.09 | 0.121 | 7.68225884322215e-22 |
| PLAA | 3.17641383989757e-26 | 0.182339993726305 | 0.074 | 0.102 | 7.83367181195538e-22 |
| SNX2 | 3.52658310945784e-26 | 0.220066716186152 | 0.137 | 0.178 | 8.69725926454492e-22 |
| RNF138 | 3.6718123197217e-26 | 0.244805049616844 | 0.09 | 0.122 | 9.05542354289765e-22 |
| XPC | 4.16839901418635e-26 | 0.100930307222555 | 0.103 | 0.136 | 1.02801056487864e-21 |
| NOL8 | 4.47331558997319e-26 | 0.116498609224908 | 0.132 | 0.17 | 1.10320909079919e-21 |
| STRN | 4.54677726540866e-26 | 0.102538321939696 | 0.088 | 0.118 | 1.12132620919508e-21 |
| ZSWIM6 | 6.15831995181205e-26 | 0.860432111060523 | 0.082 | 0.06 | 1.51876486651589e-21 |
| CLINT1 | 6.2167996181187e-26 | 0.119595607204342 | 0.209 | 0.263 | 1.53318712182043e-21 |
| TPP2 | 6.5972959087418e-26 | 0.136041707875815 | 0.202 | 0.255 | 1.6270251170139e-21 |
| DOCK11 | 7.14326296072455e-26 | 0.135494285330024 | 0.221 | 0.278 | 1.76167151137389e-21 |
| STX8 | 8.34350348560086e-26 | 0.187802875194031 | 0.124 | 0.161 | 2.05767482961888e-21 |
| CASC3 | 8.90019006840434e-26 | 0.109836361994141 | 0.096 | 0.128 | 2.19496487466988e-21 |
| HPS3 | 9.00994085912306e-26 | 0.11975814863212 | 0.075 | 0.102 | 2.22203161467693e-21 |
| RARS2 | 9.23966800842541e-26 | 0.103260703120128 | 0.084 | 0.113 | 2.27868692423787e-21 |
| TFG | 9.64762275251582e-26 | 0.192734726354184 | 0.072 | 0.099 | 2.37929672322545e-21 |
| HSD17B11 | 1.0468401148928e-25 | 0.213022081919468 | 0.145 | 0.187 | 2.58171709134862e-21 |
| RFTN1 | 1.09578446397733e-25 | 0.148109720901358 | 0.13 | 0.169 | 2.70242364506088e-21 |
| CDC73 | 1.16985569072375e-25 | 0.202708394470492 | 0.123 | 0.16 | 2.88509810446292e-21 |
| CST3 | 1.29473228780079e-25 | 0.790395782429166 | 0.184 | 0.161 | 3.19306876817432e-21 |
| MKNK1 | 1.44905384064523e-25 | 0.203938609819738 | 0.078 | 0.106 | 3.57365658179927e-21 |
| UBXN2A | 1.46511912690944e-25 | 0.145383433923091 | 0.056 | 0.079 | 3.61327679078407e-21 |
| PPP2R2B | 1.52042523718215e-25 | 0.214973909982711 | 0.094 | 0.125 | 3.74967271993863e-21 |
| TUBGCP3 | 1.52116673844151e-25 | 0.2196525015068 | 0.05 | 0.072 | 3.75150141034445e-21 |
| MTREX | 1.57811937690498e-25 | 0.156994023615236 | 0.088 | 0.119 | 3.89195800732307e-21 |
| ARFIP1 | 1.58542644986011e-25 | 0.176663009088482 | 0.041 | 0.061 | 3.909978710645e-21 |
| FAF2 | 1.6214609075892e-25 | 0.109744257163749 | 0.081 | 0.11 | 3.99884689029648e-21 |
| ADIPOR2 | 2.04435425184124e-25 | 0.113474456482193 | 0.109 | 0.142 | 5.04178645589086e-21 |
| PLSCR1 | 2.08218290829689e-25 | 0.401987421101847 | 0.142 | 0.184 | 5.13507948844179e-21 |
| INPP4A | 2.25678790407379e-25 | 0.116232026005896 | 0.173 | 0.219 | 5.56569032902679e-21 |

| Gene | P_val | Avg_log2FC | Pct.1 | Pct.2 | P_val_adj |
|---|---|---|---|---|---|
| ISCA1 | 2.37178907218623e-25 | 0.117957149867028 | 0.106 | 0.14 | 5.84930620982568e-21 |
| ZBTB2 | 2.89983848810193e-25 | 0.117441006518225 | 0.053 | 0.075 | 7.15158167935698e-21 |
| NECAP2 | 3.05568324327617e-25 | 0.149805780639874 | 0.143 | 0.183 | 7.5359260145677e-21 |
| ATP6V1H | 3.75123978365541e-25 | 0.216159869548158 | 0.085 | 0.115 | 9.25130755445098e-21 |
| FAM126A | 3.8148527834796e-25 | 0.139112715524162 | 0.054 | 0.077 | 9.40818993461738e-21 |
| TKT | 4.96942743034585e-25 | 0.295690348553807 | 0.239 | 0.301 | 1.22556019287189e-20 |
| ASCC3 | 5.49973680003395e-25 | 0.197726541395699 | 0.129 | 0.168 | 1.35634508962437e-20 |
| TBC1D1 | 6.08775733750005e-25 | 0.224081499862969 | 0.142 | 0.183 | 1.50136271457426e-20 |
| TTN | 6.33667460731321e-25 | 1.10500366733852 | 0.079 | 0.058 | 1.56275069165558e-20 |
| FMR1 | 7.41664839478387e-25 | 0.14443834613764 | 0.099 | 0.13 | 1.8290938271216e-20 |
| ICOS | 7.5364484313293e-25 | 0.210063391979522 | 0.036 | 0.055 | 1.85863891213443e-20 |
| RFLNB | 7.58838649295443e-25 | 0.173343295648923 | 0.111 | 0.144 | 1.87144787689242e-20 |
| TBK1 | 9.03447014805537e-25 | 0.20201926711026 | 0.115 | 0.15 | 2.22808102791341e-20 |
| FGR | 9.48206844946925e-25 | 0.158241951489337 | 0.281 | 0.348 | 2.33846772100811e-20 |
| ARID3B | 1.06797094140861e-24 | 0.154009901099182 | 0.081 | 0.109 | 2.63382993570191e-20 |
| OAS3 | 1.09566222561751e-24 | 0.252373962330236 | 0.058 | 0.082 | 2.7021221808179e-20 |
| HTT | 1.11955995426973e-24 | 0.110276744061249 | 0.138 | 0.176 | 2.76105875922e-20 |
| IREB2 | 1.12849356236654e-24 | 0.10177280613085 | 0.116 | 0.151 | 2.78309082350837e-20 |
| AKIRIN1 | 1.14558535464837e-24 | 0.114502432456058 | 0.155 | 0.196 | 2.82524260163381e-20 |
| MARCHF7 | 1.19944051519779e-24 | 0.152906203601559 | 0.193 | 0.242 | 2.9580601985808e-20 |
| TMEM87A | 1.37839958042932e-24 | 0.130821963691709 | 0.161 | 0.204 | 3.39940904525478e-20 |
| KCMF1 | 1.7126593766878e-24 | 0.13485242939313 | 0.132 | 0.169 | 4.22376055478745e-20 |
| ESF1 | 1.75215630257542e-24 | 0.129610851163517 | 0.052 | 0.074 | 4.3211678734115e-20 |
| RABL3 | 1.78382616364327e-24 | 0.115078877683422 | 0.065 | 0.089 | 4.39927208477704e-20 |
| NAA16 | 1.94711986094243e-24 | 0.163714094169643 | 0.115 | 0.15 | 4.80198700105622e-20 |
| ARMC8 | 2.44604501758879e-24 | 0.140874706347008 | 0.097 | 0.128 | 6.03243622237746e-20 |
| DCAF12 | 2.72652981310634e-24 | 0.188974974415928 | 0.039 | 0.058 | 6.72416782508286e-20 |
| TMEM243 | 2.75572166739756e-24 | 0.120946310617522 | 0.068 | 0.094 | 6.79616077613586e-20 |
| GNAQ | 2.78355509869059e-24 | 0.737181220884552 | 0.061 | 0.043 | 6.86480358439073e-20 |
| PFKFB3 | 2.86606882820449e-24 | 0.904891671527755 | 0.062 | 0.043 | 7.06829894411791e-20 |
| PTPN2 | 2.86862444002803e-24 | 0.188068536561042 | 0.177 | 0.225 | 7.07460159399712e-20 |
| NCKAP1L | 3.1485767062018e-24 | 0.107829084016568 | 0.257 | 0.318 | 7.76501987283488e-20 |

| Gene | P_val | Avg_log2FC | Pct.1 | Pct.2 | P_val_adj |
|---|---|---|---|---|---|
| LINC01619 | 3.44724649493806e-24 | 0.658832638653341 | 0.12 | 0.097 | 8.50159930581625e-20 |
| UBE4A | 3.47579352516957e-24 | 0.133001461122086 | 0.107 | 0.14 | 8.57200199177318e-20 |
| SDE2 | 3.94474266935226e-24 | 0.1082657695408 | 0.082 | 0.11 | 9.72852437115654e-20 |
| SAFB2 | 4.54888710160705e-24 | 0.117375029190991 | 0.125 | 0.161 | 1.12184653699833e-19 |
| CORO7 | 5.2120379914118e-24 | 0.13006734045114 | 0.141 | 0.179 | 1.28539280944198e-19 |
| SRSF4 | 6.71899398747996e-24 | 0.209451476585817 | 0.21 | 0.265 | 1.65703829719231e-19 |
| GLYR1 | 6.78264396845437e-24 | 0.143846622176367 | 0.122 | 0.158 | 1.67273565550022e-19 |
| ETFDH | 7.10674239221987e-24 | 0.146028234988624 | 0.053 | 0.075 | 1.75266480876926e-19 |
| CRTAP | 7.20839102729396e-24 | 0.146059809494613 | 0.053 | 0.075 | 1.77773339515124e-19 |
| IGSF6 | 7.64279222721226e-24 | 0.969320930292647 | 0.074 | 0.055 | 1.88486541907509e-19 |
| RIOK3 | 8.05181370544355e-24 | 0.11462042294242 | 0.348 | 0.428 | 1.98573829603649e-19 |
| ZNF101 | 9.81574610190043e-24 | 0.109592809589319 | 0.114 | 0.147 | 2.42075930365068e-19 |
| CWC22 | 1.17138549677741e-23 | 0.146267346635902 | 0.08 | 0.108 | 2.88887091215244e-19 |
| ARL14EP | 1.25145546227175e-23 | 0.113612605465639 | 0.107 | 0.139 | 3.0863394610546e-19 |
| DENND5A | 1.29444608777746e-23 | 0.715430427515539 | 0.05 | 0.034 | 3.19236294167677e-19 |
| FBXL3 | 1.43970798211512e-23 | 0.10756975358321 | 0.075 | 0.102 | 3.55060782549231e-19 |
| ACSL1 | 1.62050917152788e-23 | 1.06864476133133 | 0.087 | 0.067 | 3.99649971882205e-19 |
| DLEU1 | 1.65227078759447e-23 | 0.293088574197737 | 0.046 | 0.066 | 4.07483021636548e-19 |
| TMEM167B | 1.72974795694629e-23 | 0.16744056220989 | 0.088 | 0.117 | 4.26590441142095e-19 |
| GADD45B | 1.7583677111789e-23 | 0.10350902961679 | 0.216 | 0.267 | 4.33648644930941e-19 |
| DYNC1H1 | 1.76993820280652e-23 | 0.118954286958204 | 0.21 | 0.262 | 4.36502159576143e-19 |
| MAP2K1 | 1.81019959690461e-23 | 0.215844245193323 | 0.105 | 0.137 | 4.46431424588614e-19 |
| VCPIP1 | 1.90220064093269e-23 | 0.109064854711378 | 0.13 | 0.165 | 4.69120722066819e-19 |
| SCAF4 | 2.1921302980002e-23 | 0.122622027556168 | 0.092 | 0.122 | 5.40623174092809e-19 |
| FKBP15 | 2.31160219966557e-23 | 0.114115357150414 | 0.077 | 0.103 | 5.70087334481523e-19 |
| ATF2 | 2.43053445418977e-23 | 0.143211669115468 | 0.077 | 0.104 | 5.99418407092281e-19 |
| PPTC7 | 2.55342063475388e-23 | 0.122871863446298 | 0.08 | 0.107 | 6.29724596943002e-19 |
| GPN2 | 2.91888027428739e-23 | 0.158759709106306 | 0.045 | 0.065 | 7.19854253244757e-19 |
| SAMD9L | 3.09992595132716e-23 | 0.122325850962052 | 0.24 | 0.297 | 7.64503738116305e-19 |
| SMG7 | 3.14960576035246e-23 | 0.219283098196987 | 0.104 | 0.136 | 7.76755772618123e-19 |
| KPNA1 | 3.56986986706312e-23 | 0.141419070364052 | 0.073 | 0.098 | 8.80401306615106e-19 |
| NADK | 3.81169449262926e-23 | 0.290087291572082 | 0.095 | 0.125 | 9.40040095772229e-19 |

| Gene | P_val | Avg_log2FC | Pct.1 | Pct.2 | P_val_adj |
|---|---|---|---|---|---|
| DHX8 | 3.98845377497544e-23 | 0.145819097243201 | 0.129 | 0.165 | 9.83632469984444e-19 |
| CSNK1A1 | 3.99182890566936e-23 | 0.174628615219247 | 0.194 | 0.244 | 9.84464844716177e-19 |
| CCM2 | 4.82976545604803e-23 | 0.102031941323735 | 0.182 | 0.228 | 1.19111675677057e-18 |
| GGA2 | 4.85227430633928e-23 | 0.18465399517484 | 0.107 | 0.139 | 1.19666788942939e-18 |
| TNFSF8 | 4.99418988301084e-23 | 0.764144800416533 | 0.059 | 0.042 | 1.23166710894813e-18 |
| SLC25A12 | 5.36873155322865e-23 | 0.178118545682718 | 0.052 | 0.074 | 1.32403657565725e-18 |
| WDFY1 | 5.58868229729348e-23 | 0.110644448880756 | 0.072 | 0.097 | 1.37828082815852e-18 |
| LRRC40 | 5.67264295940006e-23 | 0.191397689216152 | 0.039 | 0.057 | 1.39898720664724e-18 |
| MKNK2 | 5.77448595742135e-23 | 0.122135805805994 | 0.155 | 0.195 | 1.42410372681925e-18 |
| SYAP1 | 6.21834020839612e-23 | 0.137397310731521 | 0.186 | 0.232 | 1.53356706219465e-18 |
| MAP3K20 | 8.02844353159538e-23 | 0.32252769520942 | 0.037 | 0.055 | 1.97997474376205e-18 |
| PTK2B | 8.50392501961547e-23 | 0.10210928729902 | 0.211 | 0.261 | 2.09723798833757e-18 |
| CDC27 | 9.83220349094656e-23 | 0.167616766157596 | 0.112 | 0.145 | 2.42481802493724e-18 |
| CDS2 | 1.20882939071427e-22 | 0.11009781709362 | 0.078 | 0.104 | 2.98121504337953e-18 |
| NAPG | 1.27737272483648e-22 | 0.175894592064826 | 0.092 | 0.121 | 3.15025661399173e-18 |
| ITPRID2 | 1.33298216043641e-22 | 0.142931014482636 | 0.055 | 0.076 | 3.28740060406827e-18 |
| ABCD3 | 1.34060112522356e-22 | 0.214469792134443 | 0.041 | 0.059 | 3.30619049502635e-18 |
| POM121 | 1.36671608687806e-22 | 0.105315037565409 | 0.108 | 0.139 | 3.37059521345868e-18 |
| VPS35L | 1.37606415030404e-22 | 0.157978110377069 | 0.055 | 0.077 | 3.39364940747982e-18 |
| TMEM165 | 1.37765168730946e-22 | 0.185119777378299 | 0.111 | 0.144 | 3.39756459124258e-18 |
| NUFIP2 | 1.38711655648579e-22 | 0.184242249548345 | 0.159 | 0.2 | 3.42090685160525e-18 |
| STK17A | 1.41983740845629e-22 | 0.120798468526816 | 0.281 | 0.347 | 3.5016030167349e-18 |
| SLC39A8 | 1.54769110897638e-22 | 0.13942781902522 | 0.038 | 0.056 | 3.81691581295755e-18 |
| JUN | 1.57419577174477e-22 | 0.381806576588731 | 0.388 | 0.378 | 3.88228161227695e-18 |
| MAN1A1 | 1.59328313844466e-22 | 0.195221168935554 | 0.101 | 0.132 | 3.92935487603222e-18 |
| RNF125 | 1.71229522941207e-22 | 0.104471013614016 | 0.174 | 0.218 | 4.22286249477605e-18 |
| SHOC2 | 1.75463297758898e-22 | 0.186524077857608 | 0.171 | 0.215 | 4.32727584932994e-18 |
| PDE3B | 1.76830382846358e-22 | 0.964162230288857 | 0.187 | 0.166 | 4.36099090175688e-18 |
| LYPLAL1 | 2.18867909147664e-22 | 0.106025476859866 | 0.043 | 0.062 | 5.39772037539968e-18 |
| SGPP1 | 2.37034286480203e-22 | 0.171744760454556 | 0.044 | 0.064 | 5.84573957317475e-18 |
| TBXAS1 | 2.64259716437169e-22 | 0.768105063105243 | 0.073 | 0.054 | 6.51717312677345e-18 |
| EDRF1 | 2.81372140703018e-22 | 0.125132494345533 | 0.094 | 0.123 | 6.93919973401784e-18 |

| Gene | P_val | Avg_log2FC | Pct. 1 | Pct. 2 | P_val_adj |
|---|---|---|---|---|---|
| CHFR | 2.88817236154617e-22 | 0.117190860044049 | 0.117 | 0.15 | 7.12281067804516e-18 |
| MARCHF5 | 3.11518546560944e-22 | 0.126587367159385 | 0.053 | 0.074 | 7.68267039528601e-18 |
| TTC19 | 3.38733185058383e-22 | 0.106939413072158 | 0.064 | 0.087 | 8.35383780990985e-18 |
| VAMP3 | 4.17926213086431e-22 | 0.131832652660486 | 0.081 | 0.107 | 1.03068962671376e-17 |
| PPP4R1 | 4.28222846593735e-22 | 0.167382759736163 | 0.053 | 0.074 | 1.05608318426947e-17 |
| H2AC6 | 4.29761650243806e-22 | 0.306104458927832 | 0.059 | 0.081 | 1.05987818183127e-17 |
| DGLUCY | 4.43925352920244e-22 | 0.128183140002834 | 0.089 | 0.116 | 1.0948087053719e-17 |
| NVL | 4.49376261780122e-22 | 0.134021388795743 | 0.046 | 0.066 | 1.10825173680214e-17 |
| MAPK6 | 4.59746003738982e-22 | 0.265352323791722 | 0.06 | 0.083 | 1.13382559442108e-17 |
| MTG2 | 4.79463950818888e-22 | 0.103518797676796 | 0.035 | 0.052 | 1.18245399550954e-17 |
| PRCP | 4.87570975627089e-22 | 0.246051136314269 | 0.055 | 0.076 | 1.20244754009153e-17 |
| TMCO3 | 5.16046831943693e-22 | 0.109210313030569 | 0.036 | 0.053 | 1.27267469693954e-17 |
| PPP1R2 | 5.17509515039702e-22 | 0.146156031736455 | 0.191 | 0.238 | 1.27628196599091e-17 |
| SLC16A7 | 5.51982989391858e-22 | 0.139863920343545 | 0.072 | 0.097 | 1.3613004484382e-17 |
| UBE2J1 | 5.58059072733406e-22 | 0.258005830229357 | 0.151 | 0.192 | 1.37628528517513e-17 |
| COPA | 6.35384454476562e-22 | 0.21496215294784 | 0.194 | 0.242 | 1.5669851416301e-17 |
| NCOR1 | 6.75215752919192e-22 | 0.153104713852938 | 0.271 | 0.333 | 1.66521708984931e-17 |
| LEPROT | 6.91069331206199e-22 | 0.116675148772993 | 0.063 | 0.086 | 1.70431518462073e-17 |
| SUCLG2 | 7.12024839302655e-22 | 0.175377479545146 | 0.132 | 0.168 | 1.75599565868821e-17 |
| AMBRA1 | 8.46808331538681e-22 | 0.236603276932961 | 0.091 | 0.119 | 2.0883987072407e-17 |
| PHF20L1 | 8.70436252400104e-22 | 0.160393652800978 | 0.154 | 0.193 | 2.14666988566914e-17 |
| SLC66A2 | 9.38428496542354e-22 | 0.120716390172192 | 0.075 | 0.1 | 2.31435235817275e-17 |
| INTS4 | 9.88498174799889e-22 | 0.104893393811239 | 0.053 | 0.074 | 2.43783419869149e-17 |
| ACBD6 | 9.92184466464883e-22 | 0.218660565873686 | 0.088 | 0.116 | 2.44692533119569e-17 |
| PPM1B | 9.94453215524618e-22 | 0.107419132753115 | 0.051 | 0.072 | 2.45252052012681e-17 |
| SFSWAP | 1.01567370966858e-21 | 0.128147162538603 | 0.15 | 0.188 | 2.50485450278466e-17 |
| IWS1 | 1.09907035296414e-21 | 0.19192600045761 | 0.106 | 0.137 | 2.71052730448017e-17 |
| GLB1 | 1.14480905947255e-21 | 0.13496217130437 | 0.067 | 0.09 | 2.82332810247121e-17 |
| GFM2 | 1.21968292690651e-21 | 0.129374827017966 | 0.039 | 0.057 | 3.00798203433683e-17 |
| MOB3A | 1.28607811014446e-21 | 0.1073991822923 | 0.149 | 0.186 | 3.17172583523828e-17 |
| FCGR1A | 1.51002895059249e-21 | 1.14343205791199 | 0.053 | 0.037 | 3.72403339795121e-17 |
| MAP1S | 1.56100319513278e-21 | 0.100654612956152 | 0.038 | 0.055 | 3.84974607983647e-17 |

| Gene | P_val | Avg_log2FC | Pct.1 | Pct.2 | P_val_adj |
|---|---|---|---|---|---|
| LINC01578 | 1.57054235591039e-21 | 0.100045957477606 | 0.342 | 0.418 | 3.8732715581462e-17 |
| HECTD1 | 1.6026118592241e-21 | 0.149904726098665 | 0.155 | 0.194 | 3.95236136721848e-17 |
| SPEN | 1.65136025272829e-21 | 0.132685156808444 | 0.157 | 0.197 | 4.0725846552785e-17 |
| OSER1 | 1.68517843206088e-21 | 0.106998560299549 | 0.109 | 0.14 | 4.15598704914854e-17 |
| TRIM27 | 1.77105586937785e-21 | 0.146460711805434 | 0.053 | 0.074 | 4.36777798505966e-17 |
| RASA2 | 1.87416582784343e-21 | 0.209497548146185 | 0.247 | 0.306 | 4.62206776462747e-17 |
| RAB8B | 2.03515188828171e-21 | 0.206535287747862 | 0.202 | 0.251 | 5.01909158688037e-17 |
| PGD | 2.04710942931954e-21 | 0.501414282636159 | 0.09 | 0.119 | 5.04858127458785e-17 |
| APOL3 | 2.18739085889568e-21 | 0.146080925240571 | 0.115 | 0.147 | 5.39454333620853e-17 |
| BIN3 | 2.26917418572602e-21 | 0.235950759056499 | 0.066 | 0.089 | 5.59623737683751e-17 |
| REEP3 | 2.27356235394122e-21 | 0.196456197840068 | 0.045 | 0.065 | 5.60705947728983e-17 |
| LPCAT3 | 2.31304231135587e-21 | 0.136740870831946 | 0.06 | 0.082 | 5.70442494826584e-17 |
| ELF2 | 2.41846344698443e-21 | 0.199557204341183 | 0.197 | 0.245 | 5.96441455295299e-17 |
| PEX14 | 2.47346055335497e-21 | 0.1793453613925 | 0.089 | 0.116 | 6.10004841668403e-17 |
| STIM2 | 2.54842967844847e-21 | 0.138118068547175 | 0.161 | 0.201 | 6.28493727298962e-17 |
| VAC14 | 2.62679661997115e-21 | 0.125118387545325 | 0.041 | 0.059 | 6.47820582417284e-17 |
| VSIR | 2.76926273462022e-21 | 0.163879080789424 | 0.349 | 0.427 | 6.8295557561204e-17 |
| CDK5RAP1 | 3.08371061723606e-21 | 0.117243066735393 | 0.064 | 0.087 | 7.60504712422757e-17 |
| GALK2 | 3.4151587590394e-21 | 0.149955570059438 | 0.04 | 0.058 | 8.42246453154297e-17 |
| PARP4 | 3.56887486687433e-21 | 0.200182693222063 | 0.17 | 0.212 | 8.80155919668547e-17 |
| EPC1 | 3.97267087558746e-21 | 0.108307017673211 | 0.284 | 0.347 | 9.79740091337379e-17 |
| THEM4 | 3.99267181631977e-21 | 0.135544637414847 | 0.037 | 0.054 | 9.84672723340782e-17 |
| RAP2C | 4.00007579373189e-21 | 0.166752358781526 | 0.061 | 0.083 | 9.8649869225016e-17 |
| MAX | 4.0989471614473e-21 | 0.191118756271185 | 0.191 | 0.238 | 1.01088234895613e-16 |
| PRKAA1 | 4.37166447208114e-21 | 0.145937579355231 | 0.09 | 0.118 | 1.07813989210465e-16 |
| TBRG1 | 4.65330073253702e-21 | 0.110979196548148 | 0.221 | 0.272 | 1.14759702665828e-16 |
| SETX | 4.93822704331567e-21 | 0.127365677428056 | 0.231 | 0.283 | 1.21786555342251e-16 |
| NUDT4 | 5.15809072122304e-21 | 0.141571252593259 | 0.074 | 0.098 | 1.27208833366803e-16 |
| OTULIN | 6.2189084174078e-21 | 0.146794777923074 | 0.066 | 0.089 | 1.53370719390111e-16 |
| BPGM | 6.76219669203302e-21 | 0.145926141170807 | 0.117 | 0.15 | 1.66769294818918e-16 |
| HACL1 | 8.85018509332618e-21 | 0.171763774501478 | 0.048 | 0.068 | 2.1826326477161e-16 |
| RNF130 | 9.11984275829676e-21 | 0.86742417047327 | 0.088 | 0.069 | 2.24913562105115e-16 |

| Gene | P_val | Avg_log2FC | Pct. 1 | Pct. 2 | P_val_adj |
|---|---|---|---|---|---|
| CUL2 | 1.12583613619568e-20 | 0.14101955833249 | 0.087 | 0.113 | 2.77653707908579e-16 |
| R3HDM4 | 1.12622801902794e-20 | 0.22688793450725 | 0.145 | 0.183 | 2.77750354052671e-16 |
| CTNNA1 | 1.22648729425534e-20 | 0.136014256685222 | 0.07 | 0.094 | 3.02476296509252e-16 |
| IFNAR2 | 1.40364095526778e-20 | 0.111663420123217 | 0.052 | 0.072 | 3.46165932388141e-16 |
| STX5 | 1.45858163138161e-20 | 0.131899714246185 | 0.092 | 0.12 | 3.59715401931333e-16 |
| CORO1C | 1.58006108409092e-20 | 0.366893912932332 | 0.034 | 0.05 | 3.89674664558502e-16 |
| ZBTB20 | 1.6971399299231e-20 | 0.675041388724527 | 0.082 | 0.063 | 4.18548649517634e-16 |
| TXLNG | 1.83671118648137e-20 | 0.146684141002362 | 0.041 | 0.058 | 4.52969712810036e-16 |
| ANKRD13A | 1.88318613256952e-20 | 0.232965954344762 | 0.114 | 0.145 | 4.64431364014295e-16 |
| SAP130 | 2.24182295505527e-20 | 0.105187990773677 | 0.052 | 0.072 | 5.5287837717573e-16 |
| USP22 | 2.35623310696686e-20 | 0.185438526632118 | 0.069 | 0.093 | 5.81094208840166e-16 |
| CREB1 | 2.39275237814004e-20 | 0.138821089337382 | 0.135 | 0.169 | 5.90100591496897e-16 |
| AKAP17A | 2.812814028749e-20 | 0.136596765450447 | 0.17 | 0.21 | 6.93696195770078e-16 |
| ARL4A | 3.35584911707488e-20 | 0.350251992940564 | 0.043 | 0.062 | 8.27619509253007e-16 |
| PURB | 3.5712784040115e-20 | 0.117772362636667 | 0.068 | 0.09 | 8.80748679997317e-16 |
| TRAPPC13 | 3.70283283660302e-20 | 0.134055582722223 | 0.043 | 0.061 | 9.13192634163036e-16 |
| PRKRIP1 | 4.41475151565765e-20 | 0.100308451487851 | 0.123 | 0.155 | 1.08876601879149e-15 |
| IST1 | 4.65455061011592e-20 | 0.168386460843335 | 0.166 | 0.207 | 1.14790527146679e-15 |
| MTMR6 | 4.96526624092855e-20 | 0.20021075109979 | 0.085 | 0.111 | 1.2245339603378e-15 |
| ZNF131 | 5.8028909838228e-20 | 0.163571599863967 | 0.13 | 0.164 | 1.43110897443038e-15 |
| YLPM1 | 5.8425121170667e-20 | 0.100456998229404 | 0.074 | 0.098 | 1.44088033831099e-15 |
| LPIN2 | 6.13837458149868e-20 | 0.1221708371411 | 0.111 | 0.142 | 1.5138459392892e-15 |
| WDR45B | 6.73550460747431e-20 | 0.148695441352422 | 0.079 | 0.104 | 1.66111014629531e-15 |
| BRAP | 6.77080692612935e-20 | 0.149920426752846 | 0.073 | 0.097 | 1.66981640412202e-15 |
| CARD11 | 6.86267341902925e-20 | 0.117009338796155 | 0.182 | 0.223 | 1.69247251860099e-15 |
| DRAM2 | 6.88520579430484e-20 | 0.235289769131287 | 0.073 | 0.096 | 1.69802945299146e-15 |
| FBXO21 | 7.05397370983846e-20 | 0.159831805362585 | 0.057 | 0.078 | 1.73965099632036e-15 |
| UBP1 | 7.08471658885876e-20 | 0.125853998200628 | 0.073 | 0.096 | 1.74723280514435e-15 |
| APAF1 | 7.70805215760991e-20 | 0.179466070358844 | 0.053 | 0.072 | 1.90095982310976e-15 |
| HEATR1 | 7.76109797415516e-20 | 0.15539466317452 | 0.059 | 0.081 | 1.91404198238614e-15 |
| NAA35 | 8.20506470069615e-20 | 0.167388264172234 | 0.052 | 0.072 | 2.02353305648568e-15 |
| TMEM205 | 1.05755518873159e-19 | 0.147527539850484 | 0.047 | 0.065 | 2.60814260644985e-15 |

| Gene | P_val | Avg_log2FC | Pct.1 | Pct.2 | P_val_adj |
|---|---|---|---|---|---|
| ASXL2 | 1.0621970303521e-19 | 0.104550107447311 | 0.074 | 0.098 | 2.61959031625435e-15 |
| ADNP | 1.10043916434358e-19 | 0.20356812575042 | 0.102 | 0.131 | 2.71390306710413e-15 |
| STXBP3 | 1.17900419225935e-19 | 0.253318538739347 | 0.086 | 0.112 | 2.90766013895001e-15 |
| TMEM161B | 1.26856217993257e-19 | 0.131337217326918 | 0.058 | 0.079 | 3.1285280481497e-15 |
| PRPF39 | 1.31300967005524e-19 | 0.186136761367583 | 0.079 | 0.104 | 3.23814444829024e-15 |
| HIBCH | 1.47877757637324e-19 | 0.259739823811961 | 0.042 | 0.059 | 3.64696125885168e-15 |
| CHD8 | 1.58926323381847e-19 | 0.151957640992349 | 0.18 | 0.222 | 3.91944098724312e-15 |
| STX17 | 1.75857812488605e-19 | 0.164792153312712 | 0.06 | 0.081 | 4.33700537159399e-15 |
| ERICH1 | 1.87214312727441e-19 | 0.200006262788113 | 0.142 | 0.178 | 4.61707938048415e-15 |
| TSEN34 | 2.02513550369025e-19 | 0.124325304915192 | 0.047 | 0.065 | 4.9943891792009e-15 |
| GNL1 | 2.66480906290586e-19 | 0.132765525471055 | 0.077 | 0.101 | 6.57195211093844e-15 |
| TOX | 3.1579196781246e-19 | 0.206255775412523 | 0.092 | 0.118 | 7.78806151019089e-15 |
| CDC42EP3 | 3.6463179236071e-19 | 0.35537163988695 | 0.109 | 0.139 | 8.99254926319982e-15 |
| GLMP | 3.94511867122269e-19 | 0.158647535809784 | 0.04 | 0.056 | 9.72945166696939e-15 |
| EEFSEC | 4.10759170792547e-19 | 0.12996207757868 | 0.054 | 0.073 | 1.01301426700858e-14 |
| HMGCR | 4.21754648552335e-19 | 0.182497183592797 | 0.038 | 0.055 | 1.04013131425977e-14 |
| ADAR | 4.29030723300666e-19 | 0.150774968788199 | 0.288 | 0.351 | 1.0580755698041e-14 |
| EXT2 | 4.35584542122297e-19 | 0.118494844095838 | 0.057 | 0.077 | 1.07423859778201e-14 |
| RAB21 | 4.79216202694663e-19 | 0.229426346611186 | 0.113 | 0.143 | 1.18184299908558e-14 |
| TRPM7 | 5.14420017291871e-19 | 0.211755063127219 | 0.139 | 0.174 | 1.26866264664521e-14 |
| PPIL2 | 5.29205774835302e-19 | 0.112531475216384 | 0.074 | 0.098 | 1.30512728189882e-14 |
| RNF13 | 5.46654801303483e-19 | 0.313434192202785 | 0.13 | 0.164 | 1.34816007097465e-14 |
| CIR1 | 5.62501264689433e-19 | 0.191059390529856 | 0.182 | 0.224 | 1.38724061897708e-14 |
| PTPN12 | 6.9120889578217e-19 | 0.158940325869276 | 0.132 | 0.165 | 1.70465937877799e-14 |
| ZNF766 | 7.00203318549282e-19 | 0.14106802952491 | 0.053 | 0.072 | 1.72684142420624e-14 |
| DNAJC13 | 7.8597562462888e-19 | 0.229146555010739 | 0.07 | 0.093 | 1.93837308545974e-14 |
| CBWD5 | 8.18312841423279e-19 | 0.148070345608911 | 0.043 | 0.061 | 2.01812312951809e-14 |
| OTULINL | 8.21042397345589e-19 | 0.268722544172254 | 0.06 | 0.08 | 2.02485476033369e-14 |
| GSPT1 | 8.24376540431563e-19 | 0.141883474528757 | 0.248 | 0.304 | 2.03307742401232e-14 |
| GPRIN3 | 1.12746545148595e-18 | 0.230344711686435 | 0.204 | 0.251 | 2.78055529645464e-14 |
| ZCCHC8 | 1.18147554346498e-18 | 0.262080242675252 | 0.076 | 0.1 | 2.91375498529332e-14 |
| SCFD1 | 1.2200421674677e-18 | 0.250930128018871 | 0.18 | 0.224 | 3.00886799340884e-14 |

| Gene | P_val | Avg_log2FC | Pct. 1 | Pct. 2 | P_val_adj |
|---|---|---|---|---|---|
| EHBP1L1 | 1.23808724126577e-18 | 0.134794911780946 | 0.181 | 0.221 | 3.05337075440964e-14 |
| SLC7A6OS | 1.28162994427506e-18 | 0.152121509253064 | 0.06 | 0.081 | 3.16075576857114e-14 |
| TRANK1 | 1.2840215483901e-18 | 0.122060740153311 | 0.132 | 0.164 | 3.16665394263966e-14 |
| RABEP1 | 1.31103461249185e-18 | 0.182765777466687 | 0.132 | 0.166 | 3.23327356132741e-14 |
| PILRA | 1.48734117648088e-18 | 0.865637221184128 | 0.071 | 0.055 | 3.66808080943715e-14 |
| BTN2A1 | 1.49028416976494e-18 | 0.128274983713397 | 0.1 | 0.127 | 3.6753388194743e-14 |
| TRAT1 | 1.57569498094534e-18 | 0.164999900325966 | 0.104 | 0.132 | 3.88597896200741e-14 |
| GIT2 | 1.77487463127582e-18 | 0.219308736400773 | 0.143 | 0.177 | 4.37719581565243e-14 |
| PPM1A | 1.97600252222885e-18 | 0.159967931820658 | 0.092 | 0.118 | 4.8732174203208e-14 |
| SEMA4D | 2.0212432470562e-18 | 0.168567041890473 | 0.21 | 0.257 | 4.98479009589e-14 |
| BDP1 | 2.05815099189652e-18 | 0.162788067954397 | 0.166 | 0.204 | 5.0758119762152e-14 |
| TM2D3 | 2.29212509356111e-18 | 0.15810236587897 | 0.068 | 0.09 | 5.65283890574042e-14 |
| TIGIT | 2.32804640276109e-18 | 0.130385520573843 | 0.126 | 0.157 | 5.7414280384894e-14 |
| ALAS2 | 2.47399899749765e-18 | 0.701836286674022 | 0.084 | 0.066 | 6.1013763276287e-14 |
| TTC7A | 2.56076886901369e-18 | 0.275517355495516 | 0.077 | 0.101 | 6.31536818476155e-14 |
| APOBEC3A | 2.91799799037011e-18 | 0.88529834665175 | 0.059 | 0.044 | 7.19636664385076e-14 |
| EMSY | 3.00311626625081e-18 | 0.142574779651598 | 0.075 | 0.098 | 7.40628533582776e-14 |
| DNAJC11 | 3.13966663778864e-18 | 0.114040752646656 | 0.048 | 0.066 | 7.74304586211435e-14 |
| CBR4 | 3.29535898113083e-18 | 0.118589205166679 | 0.035 | 0.051 | 8.12701431926486e-14 |
| GTDC1 | 3.31023977585705e-18 | 0.240873760247756 | 0.083 | 0.108 | 8.16371333521867e-14 |
| PANK3 | 3.65664402079759e-18 | 0.101577712130876 | 0.049 | 0.067 | 9.01801548409101e-14 |
| UXS1 | 4.23814156523692e-18 | 0.18359175026766 | 0.047 | 0.065 | 1.04521047281873e-13 |
| THUMPD2 | 4.58266411243177e-18 | 0.177591355435736 | 0.066 | 0.088 | 1.13017662340792e-13 |
| ACOX1 | 4.59646335197401e-18 | 0.153021832221668 | 0.049 | 0.067 | 1.13357979186383e-13 |
| MCM3AP | 4.65224298705159e-18 | 0.107466704762525 | 0.106 | 0.133 | 1.14733616546666e-13 |
| VPS45 | 5.02337419265481e-18 | 0.130642639444366 | 0.049 | 0.067 | 1.23886454339253e-13 |
| TOP1 | 5.02830120855328e-18 | 0.27875704402815 | 0.173 | 0.215 | 1.24007964405341e-13 |
| DUSP2 | 5.0682011142044e-18 | 0.35082928046831 | 0.39 | 0.382 | 1.24991975878509e-13 |
| GGNBP2 | 5.12550609328717e-18 | 0.169323931457436 | 0.196 | 0.239 | 1.26405231272648e-13 |
| NFIL3 | 5.19731528388828e-18 | 0.263246288409317 | 0.083 | 0.107 | 1.28176189531253e-13 |
| OPA1 | 5.21558366305353e-18 | 0.276004750654026 | 0.104 | 0.132 | 1.28626724298226e-13 |
| PMAIP1 | 5.50322245416816e-18 | 0.186705749143059 | 0.151 | 0.188 | 1.35720472164695e-13 |

| Gene | P_val | Avg_log2FC | Pct. 1 | Pct. 2 | P_val_adj |
|---|---|---|---|---|---|
| PHC2 | 5.89830490164738e-18 | 0.280258480564444 | 0.096 | 0.122 | 1.45463995484428e-13 |
| OSBPL8 | 5.95960415489704e-18 | 0.241510420650632 | 0.284 | 0.346 | 1.46975757668071e-13 |
| TGOLN2 | 6.33741269618714e-18 | 0.119559525196891 | 0.313 | 0.378 | 1.56293271913367e-13 |
| CCNH | 6.63243970718911e-18 | 0.22278779064515 | 0.151 | 0.187 | 1.63569228058698e-13 |
| TRAF6 | 6.74591075212267e-18 | 0.148672867805986 | 0.06 | 0.08 | 1.66367650968849e-13 |
| TIMP1 | 6.88326845952042e-18 | 0.906840875629418 | 0.116 | 0.098 | 1.69755166748693e-13 |
| STIM1 | 7.51568603441419e-18 | 0.194106035287008 | 0.154 | 0.19 | 1.85351848980723e-13 |
| EXOC2 | 8.13937637724143e-18 | 0.193397409215684 | 0.08 | 0.104 | 2.00733300215528e-13 |
| NUTM2A-AS1 | 8.18900297521107e-18 | 0.162861506712518 | 0.091 | 0.116 | 2.01957191374655e-13 |
| TRIP12 | 8.22466014542311e-18 | 0.206048885926334 | 0.227 | 0.278 | 2.02836568506425e-13 |
| ZDHHC18 | 8.47282425181835e-18 | 0.141611076252975 | 0.071 | 0.093 | 2.08956791698344e-13 |
| KLHDC2 | 8.49199127787538e-18 | 0.121618951328375 | 0.153 | 0.189 | 2.09429488894963e-13 |
| MFSD1 | 9.98797353824272e-18 | 0.304151218960716 | 0.072 | 0.094 | 2.46323403400142e-13 |
| TSPOAP1-AS1 | 1.03009794094906e-17 | 0.267174601226936 | 0.081 | 0.105 | 2.54042754196858e-13 |
| TOR1AIP2 | 1.08325315451953e-17 | 0.187032871498881 | 0.07 | 0.092 | 2.67151892967606e-13 |
| ROCK2 | 1.18165318141792e-17 | 0.123944245338977 | 0.065 | 0.086 | 2.91419307601286e-13 |
| ELP2 | 1.25965970405627e-17 | 0.180536243300672 | 0.072 | 0.093 | 3.10657276214358e-13 |
| EXOC6 | 1.32740974544679e-17 | 0.205686947178256 | 0.059 | 0.079 | 3.27365791422088e-13 |
| EIF2AK2 | 1.3373223845066e-17 | 0.259774025789568 | 0.187 | 0.23 | 3.29810446467019e-13 |
| GLS | 1.35509749201509e-17 | 0.20695096297623 | 0.161 | 0.199 | 3.34194143480761e-13 |
| RNF115 | 1.35678565063527e-17 | 0.179876865120776 | 0.133 | 0.165 | 3.34610477159669e-13 |
| LTA4H | 1.37686966073857e-17 | 0.513093219043705 | 0.115 | 0.146 | 3.39563595731346e-13 |
| TP53BP2 | 1.52913574721928e-17 | 0.103260753335129 | 0.072 | 0.094 | 3.77115457979219e-13 |
| TEX14 | 1.55746686059283e-17 | 0.261959275276534 | 0.049 | 0.067 | 3.84102477159405e-13 |
| ADIPOR1 | 1.61414514016648e-17 | 0.32326202202978 | 0.123 | 0.155 | 3.98080474467857e-13 |
| LRRC41 | 1.62150926905064e-17 | 0.14977884273588 | 0.042 | 0.058 | 3.9989661593327e-13 |
| BTBD10 | 1.62643368914615e-17 | 0.263219568064759 | 0.056 | 0.075 | 4.01111076417224e-13 |
| NEU1 | 1.65855041059127e-17 | 0.115551445440882 | 0.038 | 0.054 | 4.09031702260018e-13 |
| PRPF3 | 1.67658203980574e-17 | 0.198865003847638 | 0.094 | 0.12 | 4.13478662656892e-13 |
| RBM19 | 1.79593549217923e-17 | 0.168328917229546 | 0.071 | 0.092 | 4.42913611081242e-13 |
| ARL15 | 1.83014794419175e-17 | 0.753969586378569 | 0.091 | 0.074 | 4.5135108599657e-13 |
| ETV6 | 2.02473146518044e-17 | 0.815259548331578 | 0.107 | 0.089 | 4.99339273942801e-13 |

| Gene | P_val | Avg_log2FC | Pct.1 | Pct.2 | P_val_adj |
|---|---|---|---|---|---|
| LINC00649 | 2.03692681893548e-17 | 0.121603228165494 | 0.045 | 0.062 | 5.02346892085867e-13 |
| UBE2R2 | 2.09644248483036e-17 | 0.228023960201288 | 0.173 | 0.213 | 5.17024645608864e-13 |
| DCAF1 | 2.10540663650451e-17 | 0.14552014789938 | 0.056 | 0.075 | 5.19235384694743e-13 |
| CWC25 | 2.28608492259036e-17 | 0.169009866678509 | 0.101 | 0.128 | 5.63794263609235e-13 |
| SBDS | 2.32118053604552e-17 | 0.14757896524345 | 0.191 | 0.233 | 5.72449543799545e-13 |
| UTRN | 2.32951853312128e-17 | 0.507749118652874 | 0.371 | 0.376 | 5.7450586063837e-13 |
| EP400 | 2.79694826077464e-17 | 0.116149505474908 | 0.089 | 0.114 | 6.89783380072241e-13 |
| SLC25A26 | 2.82690521426108e-17 | 0.226013336872668 | 0.067 | 0.088 | 6.97171363941068e-13 |
| SLFN11 | 2.93346310324548e-17 | 0.159744050808687 | 0.047 | 0.064 | 7.234506705224e-13 |
| WNK1 | 2.96112236102101e-17 | 0.128552930780773 | 0.301 | 0.364 | 7.30271996675001e-13 |
| SIK3 | 3.00283742700149e-17 | 0.673439473297066 | 0.173 | 0.155 | 7.40559766247108e-13 |
| TYROBP | 3.10285243780205e-17 | 0.760359863428532 | 0.233 | 0.22 | 7.6522546821074e-13 |
| BIRC2 | 3.14894949664314e-17 | 0.185537013737849 | 0.096 | 0.121 | 7.76593924862131e-13 |
| SPTLC2 | 3.26820308791905e-17 | 0.294507769152646 | 0.09 | 0.115 | 8.06004245542596e-13 |
| GLMN | 3.43205163294069e-17 | 0.120918175438849 | 0.051 | 0.069 | 8.46412573715833e-13 |
| LATS1 | 3.53840775672018e-17 | 0.12034665663351 | 0.091 | 0.115 | 8.72642120962332e-13 |
| HCFC2 | 3.81434867137968e-17 | 0.136799287390462 | 0.038 | 0.053 | 9.40694669335656e-13 |
| SPOCK2 | 3.83305168860517e-17 | 0.412750625037647 | 0.221 | 0.202 | 9.45307207443806e-13 |
| SEC23A | 4.32820973607865e-17 | 0.21148617313028 | 0.053 | 0.072 | 1.06742308511172e-12 |
| CNOT6 | 4.34360908283255e-17 | 0.151943290409285 | 0.049 | 0.066 | 1.07122087200816e-12 |
| MARCKSL1 | 4.38584577782323e-17 | 0.191305721816914 | 0.057 | 0.076 | 1.08163728572677e-12 |
| USP33 | 4.46881641312358e-17 | 0.181461990580535 | 0.159 | 0.196 | 1.10209950380454e-12 |
| RAF1 | 4.60191587332637e-17 | 0.28038366526238 | 0.131 | 0.163 | 1.13492449267975e-12 |
| FLI1 | 4.68796287103016e-17 | 0.203259602517787 | 0.141 | 0.174 | 1.15614540325346e-12 |
| ECD | 4.71428261545798e-17 | 0.194830702784887 | 0.089 | 0.113 | 1.16263637862425e-12 |
| USP8 | 4.82777396381355e-17 | 0.201782120231366 | 0.164 | 0.201 | 1.1906256149557e-12 |
| PPWD1 | 4.83649574089592e-17 | 0.140086345343703 | 0.113 | 0.141 | 1.19277657961975e-12 |
| KIF21A | 5.20394816520798e-17 | 0.130476082968964 | 0.068 | 0.088 | 1.28339769650359e-12 |
| UBXN2B | 5.260316097333e-17 | 0.258929680406891 | 0.044 | 0.061 | 1.29729915592426e-12 |
| AC015911.11 | 5.41964579274669e-17 | 0.10523217908215 | 0.046 | 0.063 | 1.33659304540719e-12 |
| UBE2D1 | 5.95493376964355e-17 | 0.36967896413645 | 0.064 | 0.085 | 1.46860576626949e-12 |
| ATF6 | 7.08480143539516e-17 | 0.320072128433148 | 0.134 | 0.167 | 1.74725372999716e-12 |

| Gene | P_val | Avg_log2FC | Pct.1 | Pct.2 | P_val_adj |
|---|---|---|---|---|---|
| DDX3X | 8.25060829424855e-17 | 0.176190470825643 | 0.261 | 0.317 | 2.03476501752758e-12 |
| HS2ST1 | 8.73161381696633e-17 | 0.192970788346333 | 0.035 | 0.05 | 2.15339059954024e-12 |
| MFSD14B | 9.63564961559109e-17 | 0.215371823204489 | 0.063 | 0.084 | 2.37634390819707e-12 |
| TMEM87B | 1.02142287011794e-16 | 0.151313462912803 | 0.044 | 0.061 | 2.51903308228486e-12 |
| CDK10 | 1.02826953700986e-16 | 0.136155768561062 | 0.064 | 0.084 | 2.53591833217371e-12 |
| FTX | 1.07053260005963e-16 | 0.645722428213795 | 0.076 | 0.06 | 2.64014749826707e-12 |
| BAG4 | 1.10237435717535e-16 | 0.218898967657064 | 0.044 | 0.06 | 2.71867563966586e-12 |
| MTMR4 | 1.28909744486207e-16 | 0.119331671920994 | 0.037 | 0.052 | 3.17917211851884e-12 |
| NELFA | 1.2914252858461e-16 | 0.150976563505469 | 0.038 | 0.053 | 3.18491303995366e-12 |
| SERINC1 | 1.41683669284273e-16 | 0.168525822850714 | 0.194 | 0.236 | 3.49420265188875e-12 |
| NORAD | 1.42464752270918e-16 | 0.173947523942287 | 0.157 | 0.193 | 3.51346572050539e-12 |
| JOSD1 | 1.45742958086092e-16 | 0.107447272429269 | 0.15 | 0.184 | 3.59431283231921e-12 |
| RNMT | 1.60973524599005e-16 | 0.284058061096052 | 0.123 | 0.154 | 3.96992906366067e-12 |
| CENPT | 1.70577468171955e-16 | 0.121570548661827 | 0.075 | 0.096 | 4.20678152005675e-12 |
| DENND1A | 1.97342100905903e-16 | 0.651597654832263 | 0.074 | 0.058 | 4.86685089254138e-12 |
| IRF9 | 2.10064133565433e-16 | 0.116468083671696 | 0.133 | 0.164 | 5.18060166199071e-12 |
| ACSL3 | 2.11989201506938e-16 | 0.173039848483761 | 0.061 | 0.08 | 5.2280776875641e-12 |
| VPS37B | 2.15187832117757e-16 | 0.143754404610443 | 0.22 | 0.265 | 5.30696231568812e-12 |
| AIF1 | 2.22085007859522e-16 | 0.945424230948994 | 0.184 | 0.17 | 5.47706046383154e-12 |
| WDR75 | 2.26857484220294e-16 | 0.177772875503553 | 0.058 | 0.077 | 5.59475927584089e-12 |
| SLC38A1 | 2.31923232480552e-16 | 0.181253538141696 | 0.318 | 0.386 | 5.71969075943537e-12 |
| PIK3R1 | 2.35644731159421e-16 | 0.406524238743635 | 0.389 | 0.397 | 5.81147035985365e-12 |
| ABR | 2.49317576849023e-16 | 0.159303489485824 | 0.11 | 0.137 | 6.14867008025061e-12 |
| SP1 | 2.54363697049078e-16 | 0.179460384509131 | 0.041 | 0.056 | 6.27311749662436e-12 |
| GMIP | 2.75343425247804e-16 | 0.146705213478719 | 0.127 | 0.156 | 6.79051955346135e-12 |
| APOL6 | 2.80707312387245e-16 | 0.124280173352184 | 0.289 | 0.347 | 6.92280373809422e-12 |
| AC118549.1 | 3.03467316945022e-16 | 0.135708816058889 | 0.064 | 0.084 | 7.48411097049814e-12 |
| BCL2A1 | 3.21478527149321e-16 | 1.22799341543581 | 0.095 | 0.079 | 7.92830343655655e-12 |
| FOXJ3 | 3.26230990822182e-16 | 0.158933057755847 | 0.117 | 0.145 | 8.04550869565666e-12 |
| VPS41 | 3.57398841960982e-16 | 0.208471937801961 | 0.084 | 0.108 | 8.81417024044173e-12 |
| SUN1 | 3.61101931204565e-16 | 0.200320010490476 | 0.041 | 0.057 | 8.90549582736698e-12 |
| IFIH1 | 3.68339903982554e-16 | 0.202888754318947 | 0.088 | 0.111 | 9.08399871201776e-12 |

| Gene | P_val | Avg_log2FC | Pct.1 | Pct.2 | P_val_adj |
|---|---|---|---|---|---|
| GAK | 3.70307044923522e-16 | 0.120094995442087 | 0.166 | 0.201 | 9.13251234190389e-12 |
| TRMT11 | 3.72449932601473e-16 | 0.184522472688621 | 0.04 | 0.055 | 9.18536023781752e-12 |
| RSBN1 | 3.77405774260337e-16 | 0.143549001138372 | 0.096 | 0.121 | 9.30758120480843e-12 |
| C1orf162 | 4.10471405885351e-16 | 0.983215061357418 | 0.082 | 0.066 | 1.01230458119445e-11 |
| CD4 | 4.37687216862913e-16 | 0.726729239844424 | 0.058 | 0.043 | 1.07942421422732e-11 |
| FUBP3 | 4.57553282750796e-16 | 0.180528678308582 | 0.064 | 0.083 | 1.12841790592001e-11 |
| SRGN | 4.67903441466577e-16 | 0.254842160148352 | 0.756 | 0.811 | 1.15394346734487e-11 |
| WAC | 4.98493161693173e-16 | 0.147750511874121 | 0.301 | 0.364 | 1.2293838353677e-11 |
| LONP2 | 5.41502894080879e-16 | 0.144272234377187 | 0.113 | 0.141 | 1.33545443738226e-11 |
| PNPLA2 | 6.18174373380909e-16 | 0.108934034923629 | 0.156 | 0.19 | 1.524541639632e-11 |
| TATDN3 | 6.51124620663228e-16 | 0.205950631256841 | 0.056 | 0.074 | 1.60580353947965e-11 |
| TOP3A | 6.62015031527545e-16 | 0.152848329055672 | 0.037 | 0.051 | 1.63266147075323e-11 |
| IDE | 7.12352082353415e-16 | 0.236264321490394 | 0.044 | 0.06 | 1.75680270549999e-11 |
| GSE1 | 7.46000723958137e-16 | 0.102164812340805 | 0.075 | 0.096 | 1.83978698542556e-11 |
| PJA2 | 7.49423370852022e-16 | 0.267815410676782 | 0.143 | 0.177 | 1.84822791719526e-11 |
| NXF1 | 8.67764715522946e-16 | 0.138063722818658 | 0.142 | 0.173 | 2.14008134142269e-11 |
| RHOU | 8.74245103484408e-16 | 0.167896436156686 | 0.039 | 0.054 | 2.15606327421325e-11 |
| ABHD13 | 9.14013990782043e-16 | 0.142547020710192 | 0.061 | 0.079 | 2.25414130406667e-11 |
| GOSR1 | 9.34681049307798e-16 | 0.253689414277138 | 0.083 | 0.106 | 2.30511040380289e-11 |
| CEP95 | 9.54809973822655e-16 | 0.171475142950001 | 0.107 | 0.133 | 2.35475235744143e-11 |
| MICU2 | 9.91427442232097e-16 | 0.265170365572968 | 0.131 | 0.163 | 2.4450583580328e-11 |
| LCP2 | 1.007100400212e-15 | 0.162472441670966 | 0.319 | 0.385 | 2.48371100700282e-11 |
| PTS | 1.03003078800656e-15 | 0.210595225378631 | 0.105 | 0.131 | 2.54026192938178e-11 |
| RPRD1A | 1.08385007885744e-15 | 0.173894486404867 | 0.067 | 0.087 | 2.67299106447823e-11 |
| PRR14L | 1.10329322817669e-15 | 0.117650745419652 | 0.074 | 0.095 | 2.72094175932936e-11 |
| P4HA1 | 1.1081846649267e-15 | 0.185317303557523 | 0.038 | 0.053 | 2.73300502064222e-11 |
| DMTF1 | 1.11552933477947e-15 | 0.181606025236647 | 0.132 | 0.162 | 2.75111844543313e-11 |
| SNX14 | 1.12125125821611e-15 | 0.117756902588894 | 0.093 | 0.117 | 2.76522985301258e-11 |
| RIT1 | 1.1442769342417e-15 | 0.158517988722905 | 0.067 | 0.087 | 2.82201577522687e-11 |
| PRKCH | 1.15183066707112e-15 | 0.254153782581697 | 0.376 | 0.455 | 2.84064479113078e-11 |
| MBTPS1 | 1.19200623189024e-15 | 0.179292205414665 | 0.089 | 0.112 | 2.9397257690877e-11 |
| BCOR | 1.20106527513071e-15 | 0.108885243467594 | 0.036 | 0.05 | 2.96206718152736e-11 |

| Gene | P_val | Avg_log2FC | Pct. 1 | Pct. 2 | P_val_adj |
|---|---|---|---|---|---|
| PDP1 | 1.21600606269235e-15 | 0.139938792666302 | 0.056 | 0.074 | 2.99891415181188e-11 |
| CCDC57 | 1.24415207573348e-15 | 0.195631539557807 | 0.071 | 0.092 | 3.06832784917392e-11 |
| EPM2AIP1 | 1.26254504514912e-15 | 0.128151288697811 | 0.066 | 0.085 | 3.11368859034676e-11 |
| LEMD3 | 1.35694040917835e-15 | 0.1628863953641 | 0.087 | 0.11 | 3.34648643711564e-11 |
| AKAP8L | 1.60020885216775e-15 | 0.107996605106188 | 0.179 | 0.216 | 3.94643507121612e-11 |
| AL118516.1 | 1.67962009556076e-15 | 0.145384027091002 | 0.039 | 0.053 | 4.14227907967195e-11 |
| NSUN6 | 1.77372177348441e-15 | 0.160466904551115 | 0.06 | 0.078 | 4.37435263776725e-11 |
| UBN1 | 1.81238969627995e-15 | 0.262410087637014 | 0.122 | 0.151 | 4.46971546896562e-11 |
| RPRD2 | 1.81493331398045e-15 | 0.203926339773353 | 0.1 | 0.126 | 4.47598853893859e-11 |
| SETBP1 | 1.81582053126279e-15 | 0.208751395789015 | 0.077 | 0.099 | 4.47817659420029e-11 |
| CERS5 | 1.89177878103241e-15 | 0.149902781497024 | 0.049 | 0.065 | 4.66550482978213e-11 |
| EVI2B | 1.90190380084968e-15 | 0.218169762762411 | 0.316 | 0.382 | 4.69047515365548e-11 |
| HMGXB3 | 1.92112102053287e-15 | 0.136707562479617 | 0.088 | 0.111 | 4.73786866083816e-11 |
| EAF1 | 1.95211556573954e-15 | 0.168410600917483 | 0.047 | 0.063 | 4.81430740822684e-11 |
| IRF7 | 1.95785171676496e-15 | 0.18285499290153 | 0.137 | 0.167 | 4.82845390388575e-11 |
| GDI1 | 1.98494718175861e-15 | 0.100485608782632 | 0.121 | 0.148 | 4.89527673965309e-11 |
| USF3 | 2.01029379936943e-15 | 0.124606689513338 | 0.116 | 0.142 | 4.95778656800488e-11 |
| G2E3 | 2.0491623835245e-15 | 0.182843070627776 | 0.089 | 0.112 | 5.05364427024813e-11 |
| LGALS9 | 2.06669936536441e-15 | 0.19886412973959 | 0.176 | 0.213 | 5.09689397486170e-11 |
| FOCAD | 2.17714258058218e-15 | 0.24997591278148 | 0.038 | 0.052 | 5.36926903223178e-11 |
| BABAM2 | 2.20765580799213e-15 | 0.289122039935177 | 0.124 | 0.154 | 5.44452075367019e-11 |
| PHRF1 | 2.22893640208326e-15 | 0.128161925577431 | 0.062 | 0.081 | 5.49700295481774e-11 |
| ELP4 | 2.26002386880781e-15 | 0.351840693275379 | 0.064 | 0.084 | 5.57367086525381e-11 |
| ZNF430 | 2.56901641922281e-15 | 0.111278146227786 | 0.096 | 0.12 | 6.33570829308729e-11 |
| RAB1A | 2.96587613314543e-15 | 0.278524458968729 | 0.162 | 0.199 | 7.31444371956326e-11 |
| PAFAH1B2 | 3.02135830982368e-15 | 0.232377206271088 | 0.074 | 0.095 | 7.45127386368716e-11 |
| UBA6 | 3.27336543978498e-15 | 0.256314385725072 | 0.105 | 0.131 | 8.07277384759772e-11 |
| TMUB2 | 3.59796389159952e-15 | 0.151369988756606 | 0.091 | 0.114 | 8.87329854946274e-11 |
| MFHAS1 | 3.77008711319605e-15 | 0.12513131632917 | 0.036 | 0.05 | 9.29778883856409e-11 |
| UBE2E1 | 3.86456440877692e-15 | 0.367597866355202 | 0.086 | 0.11 | 9.53078874492563e-11 |
| RBM25 | 3.89831046493255e-15 | 0.147110757658995 | 0.309 | 0.372 | 9.61401326861666e-11 |
| TBL1XR1 | 3.92521672166643e-15 | 0.332131056198852 | 0.107 | 0.134 | 9.68036947897375e-11 |

| Gene | P_val | Avg_log2FC | Pct. 1 | Pct. 2 | P_val_adj |
|---|---|---|---|---|---|
| SP3 | 3.96876664074248e-15 | 0.221203910505623 | 0.193 | 0.235 | 9.78777228939911e-11 |
| TNFAIP2 | 4.03186827536723e-15 | 0.66276625356747 | 0.058 | 0.044 | 9.94339354071066e-11 |
| CYP20A1 | 4.1139563302074e-15 | 0.16893654958605 | 0.089 | 0.112 | 1.01458391015575e-10 |
| LINS1 | 4.4046113619658e-15 | 0.110903461700285 | 0.069 | 0.088 | 1.08626525408801e-10 |
| LST1 | 4.49283228312652e-15 | 0.932985657906745 | 0.129 | 0.114 | 1.10802229766466e-10 |
| RNPC3 | 4.56944112926023e-15 | 0.206924769932363 | 0.149 | 0.182 | 1.12691557129816e-10 |
| TJAP1 | 4.78615134835766e-15 | 0.115553204582436 | 0.045 | 0.061 | 1.18036064553197e-10 |
| KDM3A | 4.88744945011322e-15 | 0.154034568516541 | 0.112 | 0.138 | 1.20534278338692e-10 |
| ZNF83 | 5.01384413390014e-15 | 0.113144301875175 | 0.065 | 0.084 | 1.23651424030245e-10 |
| U2AF1L4 | 5.19793965276008e-15 | 0.106214864227182 | 0.09 | 0.112 | 1.28191587716369e-10 |
| LDB1 | 5.34756268100306e-15 | 0.133548176096123 | 0.093 | 0.116 | 1.31881590838898e-10 |
| IL16 | 5.37347461935046e-15 | 0.113748114495795 | 0.222 | 0.265 | 1.32520631062421e-10 |
| UPF1 | 5.63471440535493e-15 | 0.107766421528444 | 0.075 | 0.096 | 1.38963326664863e-10 |
| SYTL2 | 5.74712870839524e-15 | 0.155036979312799 | 0.119 | 0.146 | 1.41735688206443e-10 |
| MAP4K5 | 5.81903010409e-15 | 0.18315335749681 | 0.111 | 0.137 | 1.43508920427068e-10 |
| UIMC1 | 5.8539583968417e-15 | 0.298741487290676 | 0.094 | 0.119 | 1.4437032198291e-10 |
| AMMECR1 | 5.98453688593069e-15 | 0.139363297207509 | 0.045 | 0.06 | 1.47590648680823e-10 |
| FAM172A | 6.03738405369274e-15 | 0.298102983360351 | 0.175 | 0.214 | 1.4889396553217e-10 |
| METTL14 | 6.1051628949582e-15 | 0.131239975417199 | 0.072 | 0.092 | 1.50565527315459e-10 |
| PSMA3-AS1 | 7.02892678607197e-15 | 0.117468356403903 | 0.195 | 0.234 | 1.73347392398107e-10 |
| RASSF3 | 7.55380564089332e-15 | 0.27897226542061 | 0.12 | 0.148 | 1.86291954715711e-10 |
| GADD45A | 8.46846759863988e-15 | 0.184602052530055 | 0.065 | 0.084 | 2.08849347917657e-10 |
| REXO1 | 9.19060007111886e-15 | 0.112761403897426 | 0.087 | 0.108 | 2.26658578953933e-10 |
| AQR | 9.30265589482406e-15 | 0.259196016725809 | 0.095 | 0.12 | 2.29422099678151e-10 |
| CNOT2 | 9.59416679857e-15 | 0.243351474319107 | 0.18 | 0.218 | 2.36611341586333e-10 |
| LYSMD2 | 9.76148716991361e-15 | 0.31322608378431 | 0.042 | 0.057 | 2.40737796584409e-10 |
| TTC13 | 1.04280868120504e-14 | 0.116063880336551 | 0.089 | 0.112 | 2.57177476958788e-10 |
| SETDB2 | 1.20170373557839e-14 | 0.129549397935921 | 0.048 | 0.064 | 2.96364175268341e-10 |
| NFAT5 | 1.23239233325564e-14 | 0.6539829979354 | 0.126 | 0.109 | 3.03932597227505e-10 |
| SCAI | 1.26694846568378e-14 | 0.14621497027729 | 0.036 | 0.05 | 3.12454830606935e-10 |
| HIPK1 | 1.31046768112361e-14 | 0.162671671020916 | 0.099 | 0.123 | 3.23187539518705e-10 |
| EXOG | 1.32780356423709e-14 | 0.130523999395599 | 0.043 | 0.057 | 3.27462915012151e-10 |

| Gene | P_val | Avg_log2FC | Pct. 1 | Pct. 2 | P_val_adj |
|---|---|---|---|---|---|
| CEP120 | 1.37659779657515e-14 | 0.106697392053388 | 0.085 | 0.107 | 3.39496548591364e-10 |
| EDEM1 | 1.38738733807396e-14 | 0.207306583425289 | 0.048 | 0.064 | 3.42157465315799e-10 |
| TRIM14 | 1.49118719386309e-14 | 0.172207167861338 | 0.103 | 0.127 | 3.67756585750515e-10 |
| PPP1R12A | 1.49945318317083e-14 | 0.137671409609063 | 0.342 | 0.411 | 3.6979514403359e-10 |
| MED21 | 1.50820421400136e-14 | 0.175335532091248 | 0.045 | 0.06 | 3.71953323257016e-10 |
| SHPRH | 1.62877865320578e-14 | 0.136292723464121 | 0.062 | 0.08 | 4.0168939145361e-10 |
| SKIV2L | 1.68158903155145e-14 | 0.131530533838169 | 0.053 | 0.07 | 4.14713486961218e-10 |
| KIZ | 1.80045000597695e-14 | 0.133621794838732 | 0.073 | 0.093 | 4.44026980474035e-10 |
| PIK3C3 | 1.84133700802899e-14 | 0.154203964516704 | 0.075 | 0.094 | 4.5411053292011e-10 |
| BTG1 | 1.90365403911816e-14 | 0.219230774876332 | 0.62 | 0.675 | 4.69479159127321e-10 |
| NUMA1 | 2.0798194407635e-14 | 0.107413835090105 | 0.144 | 0.174 | 5.12925070481094e-10 |
| FBXO28 | 2.25580947051679e-14 | 0.104634866716328 | 0.061 | 0.078 | 5.56327731618851e-10 |
| NIPAL3 | 2.26860929123387e-14 | 0.150871245601023 | 0.084 | 0.105 | 5.59484423404097e-10 |
| SLC25A40 | 2.30239850929574e-14 | 0.210902178583835 | 0.036 | 0.05 | 5.67817520362515e-10 |
| CNTRL | 2.486356743123e-14 | 0.198901073883321 | 0.209 | 0.251 | 6.13185299988995e-10 |
| SETD5 | 2.56173819030112e-14 | 0.176062551971876 | 0.13 | 0.159 | 6.31775872492063e-10 |
| GRAMD2B | 2.58517097010015e-14 | 0.151463952508922 | 0.037 | 0.05 | 6.37554864646099e-10 |
| IL15RA | 2.63568439542036e-14 | 0.184249218849574 | 0.046 | 0.062 | 6.50012485598569e-10 |
| NR2C1 | 2.65049160085885e-14 | 0.158670736001508 | 0.078 | 0.099 | 6.5366423860381e-10 |
| AEBP2 | 2.74005544567466e-14 | 0.210046408948261 | 0.093 | 0.116 | 6.75752474012284e-10 |
| TSPAN5 | 3.02128976279421e-14 | 0.398999916863075 | 0.077 | 0.098 | 7.45110481300309e-10 |
| PHF3 | 3.35907112212124e-14 | 0.151506195118237 | 0.224 | 0.268 | 8.28414120137541e-10 |
| APOBR | 3.43321994485707e-14 | 0.152722936306345 | 0.08 | 0.1 | 8.46700702800651e-10 |
| PHF2 | 3.58709457720092e-14 | 0.138349125341856 | 0.056 | 0.073 | 8.84649264629292e-10 |
| SYK | 3.98008014840439e-14 | 0.675881233855582 | 0.06 | 0.047 | 9.81567366199491e-10 |
| ZNF487 | 4.30613110326593e-14 | 0.204614793051479 | 0.04 | 0.054 | 1.06197805268744e-09 |
| PDK3 | 4.47853734636064e-14 | 0.226899187759499 | 0.062 | 0.08 | 1.10449688035946e-09 |
| NFATC1 | 4.62293669377515e-14 | 0.103092880725816 | 0.05 | 0.066 | 1.14010864741883e-09 |
| DHX40 | 5.17099917469062e-14 | 0.22970888525362 | 0.06 | 0.078 | 1.2752718164622e-09 |
| DICER1 | 5.23536507346108e-14 | 0.25855672786447 | 0.123 | 0.15 | 1.29114573441697e-09 |
| ZNF638 | 5.40881067510817e-14 | 0.137561293 | 0.181 | 0.217 | 1.33392088869518e-09 |
| USP24 | 5.96842114629146e-14 | 0.200113205899747 | 0.129 | 0.157 | 1.4719320230984e-09 |

| Gene | P_val | Avg_log2FC | Pct. 1 | Pct. 2 | P_val_adj |
|---|---|---|---|---|---|
| RNF149 | 5.99369979348667e-14 | 0.472399169711297 | 0.4 | 0.416 | 1.47816624306968e-09 |
| ADPGK | 6.07399749809413e-14 | 0.190591638321307 | 0.129 | 0.157 | 1.49796926297997e-09 |
| WWP1 | 6.69917183216685e-14 | 0.194436247887817 | 0.153 | 0.185 | 1.65214975724899e-09 |
| PPP6R3 | 7.09284332946385e-14 | 0.251909730504486 | 0.181 | 0.219 | 1.74923702191237e-09 |
| FEZ2 | 7.1709204215765e-14 | 0.259741868205482 | 0.043 | 0.058 | 1.7684923943692e-09 |
| PHACTR4 | 7.53141602302643e-14 | 0.189688347276415 | 0.091 | 0.114 | 1.85739781959878e-09 |
| UBE2O | 7.66151412486847e-14 | 0.107709710173769 | 0.052 | 0.068 | 1.88948261347506e-09 |
| CWF19L2 | 7.68783537483157e-14 | 0.24037205191176 | 0.081 | 0.102 | 1.89597396014096e-09 |
| LYSMD3 | 8.04785048404358e-14 | 0.105594215935589 | 0.065 | 0.083 | 1.98476088637483e-09 |
| CARD19 | 8.75975439370755e-14 | 0.251243233531443 | 0.073 | 0.092 | 2.16033062857615e-09 |
| TPK1 | 8.92893791095966e-14 | 0.271516895453586 | 0.06 | 0.078 | 2.20205466760087e-09 |
| SKAP2 | 9.19823630444642e-14 | 0.351482822582275 | 0.079 | 0.1 | 2.26846903740258e-09 |
| RFX3-AS1 | 9.24853454733421e-14 | 0.124696204313311 | 0.095 | 0.118 | 2.28087359006356e-09 |
| MTERF4 | 9.32589247000137e-14 | 0.125693684637799 | 0.092 | 0.114 | 2.29995160095174e-09 |
| ORC2 | 9.42794200705945e-14 | 0.294475963638601 | 0.048 | 0.063 | 2.325119057781e-09 |
| DCAF10 | 9.45140829987247e-14 | 0.124231243013911 | 0.067 | 0.085 | 2.33090631491455e-09 |
| WIPF2 | 9.84968076870215e-14 | 0.101603678481137 | 0.074 | 0.093 | 2.42912827117733e-09 |
| KIF21B | 1.01904436220244e-13 | 0.168815280429365 | 0.105 | 0.129 | 2.51316720606366e-09 |
| FBXL5 | 1.05120208374858e-13 | 0.312973659749902 | 0.203 | 0.246 | 2.59247457894074e-09 |
| RHBDF2 | 1.13048442493496e-13 | 0.106379586602101 | 0.176 | 0.209 | 2.7880006887746e-09 |
| NCOA7 | 1.1668553223617e-13 | 0.213329555106392 | 0.122 | 0.15 | 2.87769859600842e-09 |
| SUPT6H | 1.24305670982376e-13 | 0.161211696142276 | 0.159 | 0.191 | 3.06562645776735e-09 |
| TESPA1 | 1.28496433520682e-13 | 0.170940945639652 | 0.087 | 0.109 | 3.16897904348707e-09 |
| SLC44A2 | 1.37562687558193e-13 | 0.104401239851705 | 0.195 | 0.232 | 3.39257100056015e-09 |
| FKBP5 | 1.48837213386803e-13 | 0.305542066576395 | 0.164 | 0.199 | 3.67062335654535e-09 |
| ZNF331 | 1.49142545187849e-13 | 0.586915618033109 | 0.068 | 0.054 | 3.67815344942274e-09 |
| NOL9 | 1.51879665718952e-13 | 0.265414351941665 | 0.059 | 0.076 | 3.74565631596078e-09 |
| NEMF | 1.67116011134067e-13 | 0.256884023987627 | 0.162 | 0.196 | 4.12141506658835e-09 |
| VPS39 | 1.78302729992034e-13 | 0.143221591621045 | 0.102 | 0.124 | 4.39730192706353e-09 |
| KAT2B | 1.80982294833481e-13 | 0.163954023738879 | 0.163 | 0.195 | 4.4633853551833e-09 |
| PPP3CA | 1.9332876964148e-13 | 0.253710950170288 | 0.152 | 0.184 | 4.76787411689818e-09 |
| GOLGA2 | 1.93760169265627e-13 | 0.151622692525214 | 0.091 | 0.112 | 4.77851329442889e-09 |

| Gene | P_val | Avg_log2FC | Pct. 1 | Pct. 2 | P_val_adj |
|---|---|---|---|---|---|
| OFD1 | 1.99991161061268e-13 | 0.132943883299119 | 0.172 | 0.206 | 4.93218201409298e-09 |
| RASA3 | 2.0078593092113e-13 | 0.219472834043835 | 0.218 | 0.261 | 4.9517826283769e-09 |
| ANKRD36B | 2.02085818767764e-13 | 0.243906448155803 | 0.075 | 0.095 | 4.9838404624506e-09 |
| STK4 | 2.02788044358152e-13 | 0.110150367617332 | 0.364 | 0.435 | 5.00115874996074e-09 |
| DENND6A | 2.23341492579174e-13 | 0.165713177313858 | 0.086 | 0.107 | 5.50804788998758e-09 |
| PACSIN2 | 2.26202989964762e-13 | 0.346910752502375 | 0.069 | 0.087 | 5.57861813851095e-09 |
| CSNK1D | 2.27757564832227e-13 | 0.125039154422876 | 0.188 | 0.223 | 5.61695706389238e-09 |
| BAZ1A | 2.37189621640866e-13 | 0.278623207729428 | 0.216 | 0.259 | 5.84957044890705e-09 |
| CHMP7 | 2.50603301347849e-13 | 0.153168704936834 | 0.037 | 0.05 | 6.18037861784066e-09 |
| LETMD1 | 2.51484499084859e-13 | 0.164267622681422 | 0.065 | 0.082 | 6.20211071643079e-09 |
| LPP | 2.82294474095273e-13 | 0.565155758973927 | 0.145 | 0.129 | 6.96194632013761e-09 |
| MLLT3 | 3.11277446016585e-13 | 0.182777865737177 | 0.086 | 0.108 | 7.67672437366102e-09 |
| RPAP2 | 3.40428117964714e-13 | 0.104917406764241 | 0.089 | 0.11 | 8.39563824524577e-09 |
| TDRD3 | 3.47718252975748e-13 | 0.173059746942265 | 0.059 | 0.076 | 8.57542755488789e-09 |
| EBLN3P | 3.76743195863288e-13 | 0.123259351564722 | 0.105 | 0.128 | 9.2912406963804e-09 |
| AMFR | 3.88811356917581e-13 | 0.155306585528976 | 0.049 | 0.064 | 9.58886568430139e-09 |
| RRN3 | 3.99780014623657e-13 | 0.260128470721622 | 0.073 | 0.092 | 9.85937472064863e-09 |
| SIPA1L1 | 4.05896669191902e-13 | 0.712713949142018 | 0.124 | 0.109 | 1.00102236556107e-08 |
| EVI2A | 4.16777539016775e-13 | 0.281486961909239 | 0.104 | 0.128 | 1.02785676672317e-08 |
| CSNK2A2 | 4.28939239040006e-13 | 0.118650715059657 | 0.066 | 0.084 | 1.05784995132046e-08 |
| PIEZO1 | 4.38380338197798e-13 | 0.138021258341332 | 0.109 | 0.133 | 1.08113359006341e-08 |
| PAN2 | 4.48296192294942e-13 | 0.133650444249207 | 0.037 | 0.05 | 1.10558806943779e-08 |
| PAN3 | 4.54793393955985e-13 | 0.594903887801759 | 0.142 | 0.127 | 1.12161146817425e-08 |
| UBE3A | 4.64526078293619e-13 | 0.262750177452003 | 0.135 | 0.164 | 1.14561421428772e-08 |
| PGGT1B | 4.81645155568049e-13 | 0.191628043173317 | 0.056 | 0.073 | 1.18783328266192e-08 |
| UHRF1BP1L | 4.9891917728316e-13 | 0.313107585105688 | 0.082 | 0.102 | 1.23043447501573e-08 |
| DDX6 | 5.37341209934509e-13 | 0.121685714189156 | 0.345 | 0.41 | 1.32519089194049e-08 |
| WHAMM | 5.45103376006565e-13 | 0.185127076078343 | 0.168 | 0.201 | 1.34433394590739e-08 |
| ECPAS | 6.02649113652454e-13 | 0.221555663530069 | 0.113 | 0.138 | 1.48625324408968e-08 |
| AKAP11 | 6.12359287695014e-13 | 0.136160096704655 | 0.059 | 0.075 | 1.51020047531344e-08 |
| DIDO1 | 6.14444005929508e-13 | 0.175514459680898 | 0.153 | 0.184 | 1.51534180742335e-08 |
| ZNF350 | 6.1460621878172e-13 | 0.119824232920947 | 0.064 | 0.081 | 1.51574185675948e-08 |

| Gene | P_val | Avg_log2FC | Pct.1 | Pct.2 | P_val_adj |
|---|---|---|---|---|---|
| LRMP | 6.68046893306296e-13 | 0.227378685678267 | 0.108 | 0.132 | 1.64753724827199e-08 |
| SRBD1 | 7.0697478685276e-13 | 0.291445233374556 | 0.048 | 0.063 | 1.74354121933628e-08 |
| BAZ2B | 7.11107485603782e-13 | 0.671626438744632 | 0.084 | 0.07 | 1.75373328099605e-08 |
| FAM91A1 | 7.18635511374092e-13 | 0.250860222281077 | 0.103 | 0.127 | 1.77229889815078e-08 |
| PAK1 | 7.20727012985655e-13 | 0.33869054949245 | 0.059 | 0.076 | 1.77745695942522e-08 |
| CRADD | 7.22227572267214e-13 | 0.251250839448387 | 0.042 | 0.055 | 1.7811576387254e-08 |
| CDK16 | 7.29512849053641e-13 | 0.127937659024645 | 0.048 | 0.062 | 1.79912458833609e-08 |
| CTBS | 7.39638109069483e-13 | 0.279365390133493 | 0.055 | 0.071 | 1.82409550458716e-08 |
| DOCK9 | 7.54995067266147e-13 | 0.269231199880632 | 0.047 | 0.062 | 1.86196883489177e-08 |
| SLC25A28 | 8.10302487554837e-13 | 0.135169245632025 | 0.085 | 0.105 | 1.99836799480774e-08 |
| POR | 8.37339192995083e-13 | 0.14946369196777 | 0.085 | 0.105 | 2.06504591776447e-08 |
| ADAT2 | 8.44005480657311e-13 | 0.120173388172952 | 0.061 | 0.077 | 2.08148631639706e-08 |
| MSRB1 | 9.21262512989361e-13 | 0.507391141118539 | 0.062 | 0.079 | 2.27201760953436e-08 |
| MAP2K7 | 9.26497466009944e-13 | 0.157433332789763 | 0.087 | 0.108 | 2.28492805067372e-08 |
| TRIM13 | 9.34571889778642e-13 | 0.120452249896061 | 0.047 | 0.062 | 2.30484119457209e-08 |
| TNFRSF1A | 9.90551472348187e-13 | 0.193546983266182 | 0.055 | 0.071 | 2.4428980411051e-08 |
| GTF2I | 9.9821000640742e-13 | 0.138276987666756 | 0.277 | 0.33 | 2.46178551780198e-08 |
| TANK | 1.01835882263323e-12 | 0.293374386567079 | 0.194 | 0.234 | 2.51147652837808e-08 |
| PARG | 1.01843985842312e-12 | 0.115071096867475 | 0.04 | 0.053 | 2.51167637884309e-08 |
| DHRSX | 1.03138881435794e-12 | 0.156168284143238 | 0.057 | 0.073 | 2.54361109396955e-08 |
| CCNT2 | 1.04405134133431e-12 | 0.155415998047785 | 0.09 | 0.11 | 2.57483941799867e-08 |
| WASHC2A | 1.05243291552205e-12 | 0.172834969738201 | 0.122 | 0.147 | 2.59551005626048e-08 |
| NDUFAF5 | 1.0647089473971e-12 | 0.203833976195587 | 0.046 | 0.06 | 2.62578520607073e-08 |
| C2orf68 | 1.12637906714929e-12 | 0.125558619168155 | 0.048 | 0.062 | 2.77787605540358e-08 |
| DCAF16 | 1.15849121844298e-12 | 0.112298999743988 | 0.085 | 0.105 | 2.85707104292407e-08 |
| SNX19 | 1.21520869451836e-12 | 0.228052514769579 | 0.039 | 0.052 | 2.99694768242117e-08 |
| SERGEF | 1.23177670736563e-12 | 0.162525247514644 | 0.041 | 0.055 | 3.03780771570512e-08 |
| LRRFIP2 | 1.41676147089633e-12 | 0.388841796717434 | 0.094 | 0.116 | 3.49401713952454e-08 |
| CSPP1 | 1.43270001735973e-12 | 0.118806050935464 | 0.04 | 0.053 | 3.53332478281257e-08 |
| CMTM7 | 1.52487283813126e-12 | 0.22395228234066 | 0.172 | 0.206 | 3.76064139339931e-08 |
| CD2AP | 1.59642091963372e-12 | 0.299168004297206 | 0.086 | 0.107 | 3.93709327200068e-08 |
| LILRB1 | 1.60486890926529e-12 | 0.158004079945925 | 0.058 | 0.074 | 3.95792770403006e-08 |

| Gene | P_val | Avg_log2FC | Pct.1 | Pct.2 | P_val_adj |
|---|---|---|---|---|---|
| XAF1 | 1.62180222119441e-12 | 0.122427440680084 | 0.387 | 0.455 | 3.99968863790965e-08 |
| PHTF2 | 1.65362911533372e-12 | 0.303376426204392 | 0.085 | 0.106 | 4.07818012423602e-08 |
| SELENOI | 1.71392935116044e-12 | 0.238995260305173 | 0.05 | 0.065 | 4.22689256583188e-08 |
| GNS | 1.74528059606746e-12 | 0.244216950160203 | 0.083 | 0.103 | 4.30421100602156e-08 |
| ATXN3 | 1.84738759494643e-12 | 0.141503331788895 | 0.052 | 0.067 | 4.55602728665689e-08 |
| TM9SF4 | 1.85331595782072e-12 | 0.262684815447052 | 0.064 | 0.081 | 4.57064781517747e-08 |
| LARP4B | 1.86946060401009e-12 | 0.277172250962492 | 0.095 | 0.117 | 4.61046374160968e-08 |
| ABHD17B | 1.94339335205324e-12 | 0.171461187302056 | 0.058 | 0.074 | 4.79279668483369e-08 |
| ZNF263 | 1.95256597519795e-12 | 0.122959750079843 | 0.041 | 0.054 | 4.81541820803319e-08 |
| TLK1 | 1.97278556095902e-12 | 0.24970338053515 | 0.154 | 0.185 | 4.86528375043713e-08 |
| CTSS | 1.9901735780472e-12 | 0.641426549948316 | 0.483 | 0.529 | 4.90816607818002e-08 |
| PLCG2 | 2.33874390859185e-12 | 0.302476260073065 | 0.106 | 0.13 | 5.76781022736923e-08 |
| YOD1 | 2.42330389089847e-12 | 0.120495257597445 | 0.037 | 0.05 | 5.97635205573382e-08 |
| GRB2 | 2.45168509840064e-12 | 0.261034151869397 | 0.314 | 0.38 | 6.04634578967565e-08 |
| FBRSL1 | 2.52595305788419e-12 | 0.104518578373984 | 0.107 | 0.13 | 6.22950543135399e-08 |
| PABPC1L | 2.7804136014916e-12 | 0.103272859826754 | 0.041 | 0.054 | 6.85705602399859e-08 |
| ELP1 | 2.78172425925252e-12 | 0.238486935246801 | 0.046 | 0.06 | 6.86028836816855e-08 |
| MMS19 | 2.84994214079767e-12 | 0.18464826843281 | 0.056 | 0.072 | 7.02852730763521e-08 |
| RBM41 | 3.0134365982731e-12 | 0.207616549208896 | 0.049 | 0.063 | 7.43173733866111e-08 |
| LCOR | 3.03925014080493e-12 | 0.214463629299898 | 0.127 | 0.154 | 7.49539869725311e-08 |
| WRN | 3.08251598332294e-12 | 0.247781581697144 | 0.043 | 0.057 | 7.60210091807105e-08 |
| ANKRD13C | 3.20491792338362e-12 | 0.213890497624162 | 0.079 | 0.098 | 7.90396858264868e-08 |
| GLCCI1 | 3.3824949708728e-12 | 0.245769869990879 | 0.103 | 0.125 | 8.34190909716649e-08 |
| DPP8 | 3.46114560455193e-12 | 0.212942278154143 | 0.131 | 0.158 | 8.53587728994598e-08 |
| ZNF281 | 3.49352863193891e-12 | 0.232656166653262 | 0.089 | 0.11 | 8.61574031208774e-08 |
| PLEKHA2 | 3.51216398006423e-12 | 0.203485316362461 | 0.157 | 0.189 | 8.6616988076344e-08 |
| RGS2 | 3.54996089894681e-12 | 0.731354602068669 | 0.155 | 0.141 | 8.75491356898262e-08 |
| METTL16 | 3.56725417495666e-12 | 0.153510910733576 | 0.058 | 0.074 | 8.79756224627812e-08 |
| TBC1D15 | 3.61221216047585e-12 | 0.264932834447347 | 0.122 | 0.148 | 8.90843763016553e-08 |
| NUTM2B-AS1 | 3.62080111502689e-12 | 0.247812546449088 | 0.103 | 0.126 | 8.9296197098793e-08 |
| OXR1 | 3.74540876266322e-12 | 0.297928116957232 | 0.071 | 0.09 | 9.23692709048004e-08 |
| MCCC1 | 3.77362711002004e-12 | 0.164135433520019 | 0.038 | 0.05 | 9.30651917873142e-08 |

| Gene | P_val | Avg_log2FC | Pct.1 | Pct.2 | P_val_adj |
|---|---|---|---|---|---|
| ADK | 3.91274531566839e-12 | 0.459379814272734 | 0.099 | 0.123 | 9.64961249750138e-08 |
| FBXW11 | 3.99106592124402e-12 | 0.200908739347088 | 0.067 | 0.085 | 9.84276677497199e-08 |
| DDIT3 | 4.14686289063071e-12 | 0.193217870808865 | 0.057 | 0.073 | 1.02269932608735e-07 |
| KBTBD2 | 4.32021256863588e-12 | 0.238501391993014 | 0.115 | 0.139 | 1.06545082367698e-07 |
| MED13L | 4.48640895764903e-12 | 0.595162867900148 | 0.178 | 0.165 | 1.1064381771354e-07 |
| EEA1 | 4.5163514209069e-12 | 0.203652502974339 | 0.052 | 0.067 | 1.11382258742406e-07 |
| LUZP1 | 4.58071627003416e-12 | 0.137556119577562 | 0.05 | 0.064 | 1.12969624651582e-07 |
| CORO1B | 4.68462511996869e-12 | 0.12741655452736 | 0.12 | 0.144 | 1.15532224708668e-07 |
| FBXO33 | 4.99938711827584e-12 | 0.314983912102523 | 0.14 | 0.169 | 1.23294885110919e-07 |
| DYNC1LI2 | 5.01501421795324e-12 | 0.156884330168268 | 0.095 | 0.116 | 1.23680280643163e-07 |
| ZMIZ1 | 5.17766143067867e-12 | 0.225038392012454 | 0.057 | 0.073 | 1.27691486203397e-07 |
| CCDC186 | 5.26804525225954e-12 | 0.147686751876094 | 0.163 | 0.192 | 1.29920532011225e-07 |
| MDN1 | 5.32924282613933e-12 | 0.171464205876698 | 0.063 | 0.079 | 1.31429786578248e-07 |
| GRIPAP1 | 5.55975214574416e-12 | 0.20432751230414 | 0.115 | 0.139 | 1.37114607418342e-07 |
| TAOK3 | 5.66303283055526e-12 | 0.190940282294311 | 0.314 | 0.373 | 1.39661715667154e-07 |
| AC005747.1 | 5.79911022105662e-12 | 0.826316449609889 | 0.062 | 0.05 | 1.43017656271698e-07 |
| ZC3H7A | 6.70657182784345e-12 | 0.19818430154428 | 0.177 | 0.212 | 1.65397474418275e-07 |
| MTR | 6.85387338587656e-12 | 0.226697547684935 | 0.067 | 0.084 | 1.69030225442488e-07 |
| ABHD2 | 6.9179552027509e-12 | 0.391537194820029 | 0.105 | 0.129 | 1.70610611210243e-07 |
| DCAF6 | 7.60584833340108e-12 | 0.293788502939953 | 0.046 | 0.06 | 1.87575431598337e-07 |
| TAF2 | 7.8552324530219e-12 | 0.214725221156494 | 0.086 | 0.106 | 1.93725742756426e-07 |
| ALMS1 | 8.50853617277695e-12 | 0.202641161787622 | 0.039 | 0.051 | 2.09837519093025e-07 |
| PPP1R21 | 8.76413643612284e-12 | 0.236903119150859 | 0.072 | 0.089 | 2.16141132787661e-07 |
| FURIN | 9.37932996104958e-12 | 0.137991175739744 | 0.084 | 0.103 | 2.31313035499405e-07 |
| PDXDC1 | 9.4197176764732e-12 | 0.212153092904842 | 0.098 | 0.12 | 2.32309077337182e-07 |
| PDLIM5 | 9.64608631436786e-12 | 0.20241346718156 | 0.061 | 0.077 | 2.3789178068494e-07 |
| HSPA1A | 9.67139871734911e-12 | 0.328371087402053 | 0.06 | 0.076 | 2.38516035167264e-07 |
| CTDSP2 | 9.84930386274409e-12 | 0.156653267092932 | 0.076 | 0.094 | 2.42903531862995e-07 |
| LINC00910 | 9.99582891120336e-12 | 0.220171464474371 | 0.055 | 0.07 | 2.46517132608097e-07 |
| NFKBIZ | 1.00045530693905e-11 | 0.657205710846313 | 0.162 | 0.149 | 2.46732287797309e-07 |
| CUX1 | 1.04859222682759e-11 | 0.656276828156178 | 0.08 | 0.067 | 2.58603814980221e-07 |
| MSL1 | 1.04981116711139e-11 | 0.158297001947486 | 0.107 | 0.129 | 2.58904430033011e-07 |

| Gene | P_val | Avg_log2FC | Pct.1 | Pct.2 | P_val_adj |
|---|---|---|---|---|---|
| TGFBR3 | 1.05041312898678e-11 | 0.121055188146654 | 0.267 | 0.313 | 2.5905288587072e-07 |
| AC125232.2 | 1.06341824724655e-11 | 0.159777036218633 | 0.068 | 0.085 | 2.62260208135944e-07 |
| SYNRG | 1.14713165929182e-11 | 0.145044714708949 | 0.185 | 0.219 | 2.82905609814549e-07 |
| CD5 | 1.19795396133184e-11 | 0.148091406366598 | 0.249 | 0.293 | 2.95439405943659e-07 |
| STX7 | 1.25136516125655e-11 | 0.332032160958017 | 0.073 | 0.091 | 3.0861167606909e-07 |
| MKRN1 | 1.26434602488503e-11 | 0.260874027924337 | 0.116 | 0.14 | 3.11813016657146e-07 |
| CLIP1 | 1.31493441697203e-11 | 0.24598998211888 | 0.183 | 0.217 | 3.24289125913643e-07 |
| RHOT1 | 1.31520953946105e-11 | 0.273965478903399 | 0.078 | 0.097 | 3.24356976621885e-07 |
| P2RY10 | 1.32566990158796e-11 | 0.171058296319123 | 0.109 | 0.132 | 3.26936711129623e-07 |
| CRLF3 | 1.38282087348225e-11 | 0.224643942759438 | 0.09 | 0.111 | 3.41031283818191e-07 |
| S100PBP | 1.38871875078911e-11 | 0.200499049110376 | 0.063 | 0.079 | 3.4248581831961e-07 |
| SETD2 | 1.39497167559026e-11 | 0.211824924552185 | 0.215 | 0.255 | 3.44027914634071e-07 |
| VPS52 | 1.46832449248623e-11 | 0.181971403064546 | 0.053 | 0.067 | 3.62118186336955e-07 |
| SCAF11 | 1.64981117777021e-11 | 0.159684433807787 | 0.336 | 0.4 | 4.06876432661688e-07 |
| HIVEP3 | 1.66621026060865e-11 | 0.253074093851046 | 0.145 | 0.173 | 4.10920774471304e-07 |
| RCOR3 | 1.68807452668361e-11 | 0.207150287747443 | 0.085 | 0.104 | 4.16312939770711e-07 |
| MBD6 | 1.72885876340985e-11 | 0.106762466079444 | 0.066 | 0.082 | 4.26371148232138e-07 |
| CRTC2 | 1.7493490532334e-11 | 0.206995962637354 | 0.098 | 0.118 | 4.31424463508421e-07 |
| PTAR1 | 1.75148965712089e-11 | 0.198243160209693 | 0.108 | 0.131 | 4.31952379239153e-07 |
| GPR107 | 1.99882720537744e-11 | 0.102083976463652 | 0.046 | 0.059 | 4.92950765390183e-07 |
| FOXK2 | 2.13268089093082e-11 | 0.191814304205617 | 0.06 | 0.075 | 5.25961761321359e-07 |
| PSMD5 | 2.48086675749037e-11 | 0.321893566358051 | 0.061 | 0.076 | 6.11831359732276e-07 |
| CUL5 | 2.51410592614425e-11 | 0.195875674728067 | 0.101 | 0.123 | 6.20028803505694e-07 |
| DGAT1 | 2.51891405262078e-11 | 0.136043501737688 | 0.066 | 0.082 | 6.21214583657337e-07 |
| MSL2 | 2.6175871606624e-11 | 0.185682964579114 | 0.123 | 0.147 | 6.45549345562562e-07 |
| NEDD9 | 2.63336243084091e-11 | 0.357707118723058 | 0.124 | 0.149 | 6.49439842693985e-07 |
| MTOR | 2.69867530399427e-11 | 0.120073642637841 | 0.056 | 0.071 | 6.65547303471066e-07 |
| RANBP2 | 2.71767705764371e-11 | 0.26631072835582 | 0.144 | 0.172 | 6.70233515956092e-07 |
| PIBF1 | 2.89249969783179e-11 | 0.238755871118729 | 0.08 | 0.098 | 7.13348275479276e-07 |
| PHF20 | 2.92066854133358e-11 | 0.227415731419297 | 0.163 | 0.194 | 7.20295275663687e-07 |
| STAT3 | 2.95410772364055e-11 | 0.224455408649416 | 0.202 | 0.239 | 7.28542046804233e-07 |
| ABI1 | 3.13366022920657e-11 | 0.224189331463321 | 0.195 | 0.231 | 7.72823285726925e-07 |

| Gene | P_val | Avg_log2FC | Pct.1 | Pct.2 | P_val_adj |
| --- | --- | --- | --- | --- | --- |
| RALGAPA2 | 3.16109116488633e-11 | 0.797673364992773 | 0.066 | 0.054 | 7.79588303084267e-07 |
| PEX1 | 3.18918278212013e-11 | 0.106486920875976 | 0.055 | 0.07 | 7.86516257726466e-07 |
| PRKAR2A | 3.19392658271615e-11 | 0.19110336165708 | 0.093 | 0.114 | 7.87686173829457e-07 |
| HAPLN3 | 3.24848085527721e-11 | 0.24600251649504 | 0.048 | 0.062 | 8.01140348528466e-07 |
| NFRKB | 3.4308930180659e-11 | 0.137218184209931 | 0.06 | 0.075 | 8.46126836115411e-07 |
| SENP2 | 3.55936245201323e-11 | 0.201819657170678 | 0.059 | 0.074 | 8.77809967915503e-07 |
| IFNGR1 | 3.6066980543806e-11 | 0.770417995994067 | 0.109 | 0.095 | 8.89483874171343e-07 |
| ZNF592 | 3.6869062858666e-11 | 0.191783715260152 | 0.056 | 0.071 | 9.0926482822042e-07 |
| ZBTB25 | 3.7435360245784e-11 | 0.109076941579241 | 0.056 | 0.07 | 9.23230854381526e-07 |
| SENP6 | 3.74533520303516e-11 | 0.224206852803473 | 0.182 | 0.215 | 9.23674567772531e-07 |
| USP12 | 3.92656481391876e-11 | 0.289840634732897 | 0.071 | 0.088 | 9.68369414408644e-07 |
| TMEM245 | 3.9287571070185e-11 | 0.158431386574302 | 0.055 | 0.07 | 9.68910077732903e-07 |
| TRIM56 | 4.54086638598129e-11 | 0.114695166540636 | 0.121 | 0.144 | 1.11986846811071e-06 |
| RAB28 | 4.59217229941134e-11 | 0.371231692020682 | 0.045 | 0.058 | 1.13252153248082e-06 |
| WDR20 | 4.63122089346999e-11 | 0.140957675975439 | 0.047 | 0.061 | 1.14215169674757e-06 |
| SAT2 | 4.84723571036575e-11 | 0.106195648324366 | 0.102 | 0.123 | 1.1954252708904e-06 |
| APBB1IP | 4.95670431163821e-11 | 0.145851155500238 | 0.412 | 0.491 | 1.22242241733622e-06 |
| IKZF5 | 5.07532865839695e-11 | 0.126201215492964 | 0.07 | 0.086 | 1.25167755373386e-06 |
| STAM | 5.19391122561427e-11 | 0.203469701946426 | 0.045 | 0.059 | 1.28092238646099e-06 |
| IRF2BP2 | 5.57307267061859e-11 | 0.2242448416162 | 0.162 | 0.193 | 1.37443118202796e-06 |
| SLC30A7 | 6.20613247379885e-11 | 0.144248752532082 | 0.061 | 0.076 | 1.53055639068827e-06 |
| STX16 | 6.52999952142093e-11 | 0.120741877321765 | 0.134 | 0.158 | 1.61042848197283e-06 |
| KLHL28 | 6.848637960775e-11 | 0.117132854948474 | 0.088 | 0.107 | 1.68901109388633e-06 |
| CCDC88C | 7.0135208335266e-11 | 0.134052732446605 | 0.292 | 0.343 | 1.72967450796433e-06 |
| NPC2 | 7.25149343618563e-11 | 0.552892219570199 | 0.156 | 0.188 | 1.7883633112321e-06 |
| NUP214 | 7.68531756870093e-11 | 0.289648471508484 | 0.102 | 0.124 | 1.89535301879302e-06 |
| VTI1A | 7.74607322856896e-11 | 0.247163217969111 | 0.153 | 0.183 | 1.91033657962968e-06 |
| TAF1C | 7.7970995244058e-11 | 0.10102943136329 | 0.065 | 0.08 | 1.92292068470896e-06 |
| MAN1A2 | 7.89250356525926e-11 | 0.235965492878402 | 0.152 | 0.18 | 1.94644922926424e-06 |
| TLK2 | 8.06148726384681e-11 | 0.188415778080505 | 0.1 | 0.12 | 1.9881239890099e-06 |
| TSC1 | 8.0991542296824e-11 | 0.113365055684749 | 0.056 | 0.07 | 1.99741341612427e-06 |
| MECP2 | 8.1411437624975e-11 | 0.241720506204199 | 0.164 | 0.196 | 2.00776887470713e-06 |

| Gene | P_val | Avg_log2FC | Pct. 1 | Pct. 2 | P_val_adj |
|---|---|---|---|---|---|
| TRMT1 | 8.81238251684789e-11 | 0.247484447287246 | 0.058 | 0.072 | 2.17330977630503e-06 |
| OSBP | 9.05345248163487e-11 | 0.255958844923579 | 0.061 | 0.076 | 2.23276245102079e-06 |
| CDADC1 | 9.34598377247039e-11 | 0.148315532484528 | 0.051 | 0.064 | 2.30490651796665e-06 |
| MUC20-OT1 | 9.57782227060166e-11 | 0.239915225190914 | 0.105 | 0.127 | 2.36208252837578e-06 |
| UBE4B | 1.01768150735961e-10 | 0.306319477760274 | 0.063 | 0.078 | 2.50980613345026e-06 |
| ZNF652 | 1.06644002801907e-10 | 0.313776314253832 | 0.086 | 0.105 | 2.63005439710062e-06 |
| ADAM10 | 1.07145467654094e-10 | 0.256901697651177 | 0.213 | 0.253 | 2.64242152328528e-06 |
| GLUL | 1.15125637962034e-10 | 0.488796398188863 | 0.137 | 0.165 | 2.83922848341967e-06 |
| ANAPC2 | 1.24536095303311e-10 | 0.126127243128338 | 0.044 | 0.056 | 3.07130918237026e-06 |
| ZDHHC7 | 1.24628421978441e-10 | 0.275151519727363 | 0.064 | 0.079 | 3.07358614283231e-06 |
| GRK5 | 1.24744379524896e-10 | 0.29626840171666 | 0.092 | 0.111 | 3.07644588784299e-06 |
| STK38 | 1.26696498558614e-10 | 0.158917642258998 | 0.29 | 0.341 | 3.12458904745255e-06 |
| PAPSS1 | 1.31452360670284e-10 | 0.355869642338709 | 0.042 | 0.055 | 3.24187811885055e-06 |
| ROCK1 | 1.34894121065118e-10 | 0.180272580332071 | 0.305 | 0.36 | 3.32675881370794e-06 |
| CTDP1 | 1.41831286116438e-10 | 0.160465058916994 | 0.06 | 0.075 | 3.49784317820359e-06 |
| ABL1 | 1.479157378127e-10 | 0.115591126576603 | 0.051 | 0.064 | 3.6478979259368e-06 |
| IQCB1 | 1.5419477841398e-10 | 0.259358478469809 | 0.059 | 0.074 | 3.80275162524557e-06 |
| DNAJC5 | 1.58532805803867e-10 | 0.148116414883811 | 0.091 | 0.11 | 3.90973605673497e-06 |
| RAB11FIP2 | 1.58964222356792e-10 | 0.184887000501124 | 0.069 | 0.085 | 3.9203756517632e-06 |
| UBXN7 | 1.60002604922918e-10 | 0.221980193497858 | 0.098 | 0.118 | 3.945984242609e-06 |
| RNF11 | 1.60732730744837e-10 | 0.190598687651878 | 0.091 | 0.11 | 3.96399060562917e-06 |
| PRRC2B | 1.61171620494843e-10 | 0.118693976394856 | 0.191 | 0.224 | 3.97481450464382e-06 |
| RBM6 | 1.74108281358632e-10 | 0.172915340599813 | 0.179 | 0.211 | 4.29385843486658e-06 |
| EFR3A | 1.88601370368103e-10 | 0.283590810247064 | 0.134 | 0.16 | 4.65128699601815e-06 |
| TRPS1 | 2.0095001383568e-10 | 0.829015208752665 | 0.057 | 0.047 | 4.95582924121554e-06 |
| DDI2 | 2.07436327946349e-10 | 0.25408568674256 | 0.048 | 0.061 | 5.11579471981286e-06 |
| TOR1B | 2.1086356473666e-10 | 0.180698509150047 | 0.042 | 0.054 | 5.20031723353552e-06 |
| PPP1R10 | 2.15848221549274e-10 | 0.107482046600964 | 0.071 | 0.087 | 5.3232488398482e-06 |
| AP4E1 | 2.18597048275184e-10 | 0.207591172597843 | 0.049 | 0.062 | 5.39104040456259e-06 |
| CASP8 | 2.26541259877637e-10 | 0.112185344343565 | 0.448 | 0.53 | 5.58696055110228e-06 |
| SIL1 | 2.29842942403018e-10 | 0.332978277534803 | 0.06 | 0.075 | 5.66838664554323e-06 |
| ARHGAP26 | 2.3290049908925e-10 | 0.684166699630506 | 0.226 | 0.22 | 5.74379210853909e-06 |

| Gene | P_val | Avg_log2FC | Pct.1 | Pct.2 | P_val_adj |
|---|---|---|---|---|---|
| UBASH3B | 2.35590265050857e-10 | 0.342013598811954 | 0.053 | 0.067 | 5.81012711668425e-06 |
| ACAP1 | 2.38323516651997e-10 | 0.135360626692044 | 0.328 | 0.385 | 5.87753456767155e-06 |
| MYSM1 | 2.38650195405135e-10 | 0.222429351533778 | 0.095 | 0.115 | 5.88559111908145e-06 |
| SIAH2 | 2.43467504823077e-10 | 0.191616233078821 | 0.098 | 0.118 | 6.00439560394672e-06 |
| TNPO2 | 2.51742463517018e-10 | 0.184270103033803 | 0.039 | 0.05 | 6.20847263525669e-06 |
| TRAPPC10 | 2.55056148790747e-10 | 0.164477988652574 | 0.191 | 0.225 | 6.2901947414774e-06 |
| SEPSECS | 2.76952129682601e-10 | 0.110007293941508 | 0.047 | 0.059 | 6.83019342223231e-06 |
| PARP11 | 2.93218620743052e-10 | 0.220572303678906 | 0.047 | 0.06 | 7.23135762476516e-06 |
| DMXL1 | 2.94686449573842e-10 | 0.218789523361204 | 0.098 | 0.118 | 7.26755721939009e-06 |
| FAM157C | 3.00703049978385e-10 | 0.791986365621618 | 0.06 | 0.049 | 7.41593861856693e-06 |
| FBXO42 | 3.06267335738135e-10 | 0.132914983753712 | 0.054 | 0.068 | 7.55316503397389e-06 |
| TRA2A | 3.13181399978671e-10 | 0.213383843155308 | 0.198 | 0.235 | 7.72367968627398e-06 |
| ARRDC3 | 3.22645271500988e-10 | 0.121290972165133 | 0.139 | 0.163 | 7.95707768575737e-06 |
| AC006369.1 | 3.38884906735822e-10 | 0.145913810414395 | 0.04 | 0.052 | 8.35757956991885e-06 |
| PARP12 | 3.42848858611506e-10 | 0.17227645056307 | 0.131 | 0.155 | 8.45533855107696e-06 |
| JUND | 3.54628793437638e-10 | 0.11904933852719 | 0.383 | 0.45 | 8.74585530375902e-06 |
| CREM | 3.7255058355685e-10 | 0.277325609420437 | 0.146 | 0.173 | 9.18784249167903e-06 |
| NDRG1 | 3.9408819739425e-10 | 0.200041067028702 | 0.092 | 0.111 | 9.71900312413699e-06 |
| AP1G1 | 4.20751279086843e-10 | 0.257121955508486 | 0.104 | 0.125 | 1.03765680448397e-05 |
| CELF2 | 4.22182051306999e-10 | 0.187277769184123 | 0.392 | 0.468 | 1.04118537493332e-05 |
| PPP2R2A | 4.3714625705939e-10 | 0.236025933073303 | 0.109 | 0.131 | 1.07809009915987e-05 |
| PDPK1 | 4.44448075838011e-10 | 0.238456417502309 | 0.053 | 0.067 | 1.0960978446317e-05 |
| DUSP10 | 4.66424373573329e-10 | 0.134932813459709 | 0.054 | 0.067 | 1.15029579010654e-05 |
| RSF1 | 4.71766438019674e-10 | 0.148568885893419 | 0.214 | 0.25 | 1.16347038944412e-05 |
| HPCAL1 | 4.80936733384773e-10 | 0.237875781418515 | 0.105 | 0.126 | 1.18608617187353e-05 |
| ABCB7 | 4.8570671265362e-10 | 0.243420225432188 | 0.051 | 0.065 | 1.19784989474636e-05 |
| DGKE | 4.8982608610166e-10 | 0.155120348107416 | 0.05 | 0.063 | 1.20800909354391e-05 |
| AFTPH | 5.07694339473328e-10 | 0.264798798232764 | 0.117 | 0.14 | 1.25207578000912e-05 |
| FUT8 | 5.19304226026362e-10 | 0.324022627804669 | 0.071 | 0.087 | 1.28070808222621e-05 |
| CCS | 5.21053636375478e-10 | 0.154078066190966 | 0.074 | 0.09 | 1.2850224780292e-05 |
| RESF1 | 5.41392117834724e-10 | 0.111636069582438 | 0.379 | 0.444 | 1.335181241004e-05 |
| OSBPL2 | 6.05151751600641e-10 | 0.243888553148216 | 0.075 | 0.091 | 1.4924252497975e-05 |

| Gene | P_val | Avg_log2FC | Pct. 1 | Pct. 2 | P_val_adj |
|---|---|---|---|---|---|
| GAPVD1 | 6.20078749660832e-10 | 0.254325180668453 | 0.091 | 0.11 | 1.52923821241354e-05 |
| TRIM25 | 6.45377761775477e-10 | 0.25828477477287 | 0.078 | 0.095 | 1.59163063609068e-05 |
| LUC7L3 | 6.47621514026405e-10 | 0.177990172331922 | 0.354 | 0.419 | 1.59716417789192e-05 |
| NDFIP1 | 6.71851387151815e-10 | 0.243785758360393 | 0.153 | 0.181 | 1.65691989099381e-05 |
| SREBF2 | 6.85226744784935e-10 | 0.192451030880236 | 0.109 | 0.13 | 1.68990619798861e-05 |
| SMAD4 | 6.97681336109878e-10 | 0.197704267481074 | 0.058 | 0.072 | 1.72062171111418e-05 |
| COQ8A | 7.30362211917451e-10 | 0.162088905464049 | 0.044 | 0.056 | 1.80121928703082e-05 |
| ZFAND5 | 7.90457523009395e-10 | 0.322576732018947 | 0.154 | 0.183 | 1.94942634324577e-05 |
| RIPOR2 | 8.37330752319001e-10 | 0.323680258276228 | 0.474 | 0.511 | 2.06502510136912e-05 |
| SLFN12L | 8.42753717664954e-10 | 0.190583186648721 | 0.212 | 0.247 | 2.07839921850531e-05 |
| HOTAIRM1 | 8.61517518735558e-10 | 0.142873240023385 | 0.058 | 0.072 | 2.12467450470563e-05 |
| EPS15 | 8.80588628507748e-10 | 0.276619109127579 | 0.174 | 0.206 | 2.17170767562581e-05 |
| ERCC6L2 | 8.83631833297895e-10 | 0.208891292738304 | 0.082 | 0.099 | 2.17921282727927e-05 |
| RIPK1 | 8.90265634027664e-10 | 0.214704097210455 | 0.101 | 0.12 | 2.19557310663903e-05 |
| ETV3 | 9.10130709067007e-10 | 0.127264135133845 | 0.048 | 0.06 | 2.24456435470105e-05 |
| IER5 | 9.24422674220015e-10 | 0.254825493653462 | 0.128 | 0.152 | 2.2798111991614e-05 |
| ABHD18 | 1.025629855629e-09 | 0.230901096859588 | 0.073 | 0.089 | 2.52940834995223e-05 |
| PIK3AP1 | 1.03683661426716e-09 | 0.31673769169633 | 0.12 | 0.143 | 2.55704645810568e-05 |
| POLK | 1.06417697700211e-09 | 0.259428261175637 | 0.069 | 0.085 | 2.62447326068262e-05 |
| AGPAT3 | 1.0756680405493e-09 | 0.162477542212614 | 0.089 | 0.107 | 2.65281252160268e-05 |
| ATRX | 1.07741536223362e-09 | 0.165154414612313 | 0.318 | 0.372 | 2.65712176634055e-05 |
| ABRAXAS2 | 1.09884869221864e-09 | 0.25167796813617 | 0.041 | 0.053 | 2.70998064474962e-05 |
| AFF1 | 1.11465655639404e-09 | 0.25659069695563 | 0.108 | 0.13 | 2.74896599937898e-05 |
| RBM33 | 1.14625093812594e-09 | 0.203377509223937 | 0.171 | 0.201 | 2.82688406360618e-05 |
| EZR | 1.15021251034341e-09 | 0.158295550427667 | 0.389 | 0.461 | 2.83665409300891e-05 |
| MT1E | 1.1562015954633e-09 | 0.210496571091935 | 0.449 | 0.524 | 2.8514243747316e-05 |
| GNB5 | 1.18880633868642e-09 | 0.267766383703322 | 0.055 | 0.069 | 2.93183419246846e-05 |
| MCUB | 1.19629088526371e-09 | 0.218662690854675 | 0.239 | 0.281 | 2.95029258123736e-05 |
| ATP6V1A | 1.2461523273447e-09 | 0.262390903394349 | 0.08 | 0.096 | 3.07326086969751e-05 |
| SLC12A9 | 1.26147292204059e-09 | 0.156832057439706 | 0.11 | 0.13 | 3.11104452033649e-05 |
| ZSWIM8 | 1.31122957617484e-09 | 0.13246881787913 | 0.111 | 0.131 | 3.23375438076239e-05 |
| ST3GAL1 | 1.31325601342519e-09 | 0.126941302800573 | 0.155 | 0.182 | 3.23875198030919e-05 |

| Gene | P_val | Avg_log2FC | Pct.1 | Pct.2 | P_val_adj |
|---|---|---|---|---|---|
| C9orf72 | 1.31379045162459e-09 | 0.770878440958446 | 0.064 | 0.053 | 3.24007001179655e-05 |
| AKAP10 | 1.37439768077227e-09 | 0.187517684249422 | 0.072 | 0.088 | 3.38953956032056e-05 |
| SEC24D | 1.44149353596398e-09 | 0.381335723516687 | 0.058 | 0.072 | 3.55501135839436e-05 |
| SUPT3H | 1.57708114599592e-09 | 0.294404493831946 | 0.073 | 0.089 | 3.88939752225514e-05 |
| EMILIN2 | 1.62360321215513e-09 | 0.730705480579413 | 0.058 | 0.048 | 4.00413024181697e-05 |
| AAK1 | 1.63364864757779e-09 | 0.23600237105268 | 0.247 | 0.291 | 4.02890429465634e-05 |
| FAF1 | 1.6445841483595e-09 | 0.273867145566032 | 0.124 | 0.148 | 4.0558734266842e-05 |
| NDE1 | 1.67371922295092e-09 | 0.177105524031989 | 0.082 | 0.099 | 4.12772634764157e-05 |
| AP2A2 | 1.67383608688974e-09 | 0.246687693324866 | 0.061 | 0.075 | 4.12801455748746e-05 |
| LINC01138 | 1.71088656541488e-09 | 0.11392973346164 | 0.136 | 0.159 | 4.21938844762617e-05 |
| KLHL2 | 1.72579235010744e-09 | 0.867394419617934 | 0.065 | 0.054 | 4.25614909383497e-05 |
| PCNX4 | 1.80222030323242e-09 | 0.234430000311755 | 0.051 | 0.064 | 4.44463571183179e-05 |
| PTGER4 | 1.80874961916358e-09 | 0.17848810098558 | 0.134 | 0.158 | 4.46073831078122e-05 |
| ATP2C1 | 1.89947384192333e-09 | 0.30720113681605 | 0.088 | 0.106 | 4.68448238895132e-05 |
| NR2C2 | 1.91104318972798e-09 | 0.119607338590012 | 0.066 | 0.081 | 4.71301471450714e-05 |
| NAGK | 2.00789640190719e-09 | 0.372743498418715 | 0.069 | 0.084 | 4.95187410638351e-05 |
| MGRN1 | 2.10129310176897e-09 | 0.179382825480385 | 0.071 | 0.087 | 5.18220904758264e-05 |
| PRKX | 2.17477216685441e-09 | 0.142180817173046 | 0.148 | 0.174 | 5.36342311789633e-05 |
| KLF10 | 2.42091390002499e-09 | 0.320148603485357 | 0.093 | 0.112 | 5.97045786024163e-05 |
| CPVL | 2.52409788440099e-09 | 0.746940401631608 | 0.063 | 0.052 | 6.22493020250972e-05 |
| KPNA5 | 2.64594881640224e-09 | 0.163072299276857 | 0.066 | 0.08 | 6.52543897101121e-05 |
| ITCH | 2.70929563396794e-09 | 0.312300876766274 | 0.181 | 0.214 | 6.68166489249173e-05 |
| SCO2 | 2.81207898338682e-09 | 0.390082811224958 | 0.104 | 0.124 | 6.93514918882858e-05 |
| YAF2 | 3.02458708215101e-09 | 0.369875646955622 | 0.091 | 0.11 | 7.45923666200082e-05 |
| RAPGEF2 | 3.02683484953845e-09 | 0.62279107451909 | 0.061 | 0.05 | 7.46478010593172e-05 |
| MT2A | 3.03486694453512e-09 | 0.260857278368495 | 0.719 | 0.777 | 7.4845888586125e-05 |
| GMDS | 3.04118207102287e-09 | 0.405697486247223 | 0.074 | 0.091 | 7.5001632235566e-05 |
| GMEB2 | 3.1749760959563e-09 | 0.154384556979386 | 0.05 | 0.063 | 7.83012604784743e-05 |
| ARHGEF2 | 3.24459702866651e-09 | 0.232395201128738 | 0.056 | 0.069 | 8.00182519209735e-05 |
| B4GALT5 | 3.27349935872501e-09 | 0.337839881130131 | 0.093 | 0.112 | 8.07310411848762e-05 |
| CHKB | 3.34220966791319e-09 | 0.138020106863364 | 0.068 | 0.082 | 8.2425574830075e-05 |
| KDM7A | 3.43398731963096e-09 | 0.521157319461564 | 0.136 | 0.124 | 8.46889952767388e-05 |

| Gene | P_val | Avg_log2FC | Pct. 1 | Pct. 2 | P_val_adj |
|---|---|---|---|---|---|
| YTHDC2 | 3.50273826288757e-09 | 0.245174218366023 | 0.083 | 0.099 | 8.63845310393333e-05 |
| TRRAP | 3.61051336767478e-09 | 0.240113811397486 | 0.104 | 0.124 | 8.90424806735955e-05 |
| ZDHHC20 | 3.63013791659126e-09 | 0.378233896009949 | 0.178 | 0.211 | 8.95264612989735e-05 |
| PPP6R2 | 3.73871015746079e-09 | 0.196854600398249 | 0.155 | 0.182 | 9.2204069903298e-05 |
| CENPC | 3.82858820310218e-09 | 0.274018955995228 | 0.144 | 0.169 | 9.4420642264906e-05 |
| GABARAPL1 | 3.86004199737687e-09 | 0.120010749593044 | 0.119 | 0.139 | 9.51963557393083e-05 |
| PCBP1-AS1 | 3.87737163369701e-09 | 0.117508363441721 | 0.058 | 0.071 | 9.56237392302358e-05 |
| HIF1A | 3.99746949161785e-09 | 0.442356348059565 | 0.159 | 0.19 | 9.85855926022793e-05 |
| HEATR3 | 4.00572300956948e-09 | 0.232516285758783 | 0.045 | 0.057 | 9.87891408620026e-05 |
| PBX2 | 4.17152902622315e-09 | 0.183667269219756 | 0.059 | 0.072 | 0.000102878248844715 |
| GSK3B | 4.17901257401719e-09 | 0.336416854254564 | 0.123 | 0.146 | 0.000103062808100412 |
| TAF13 | 4.30451868724508e-09 | 0.19979590700642 | 0.056 | 0.069 | 0.000106158039864838 |
| GPATCH8 | 4.35024606412151e-09 | 0.249516906154362 | 0.151 | 0.177 | 0.000107285768433365 |
| SMG1 | 4.36938836257018e-09 | 0.203710476377752 | 0.239 | 0.28 | 0.000107757855797706 |
| GCC2 | 4.42935368058662e-09 | 0.165771628870971 | 0.27 | 0.314 | 0.000109236720470627 |
| CPPED1 | 4.45171888573634e-09 | 0.539929324448843 | 0.094 | 0.113 | 0.00010978829116003 |
| PHF8 | 4.50246890107026e-09 | 0.181554002476665 | 0.042 | 0.054 | 0.000111039888038195 |
| HNRNPLL | 4.58219067059608e-09 | 0.248339990152778 | 0.149 | 0.176 | 0.00011300598631824 |
| CRTC3 | 4.64213730791248e-09 | 0.193305610728872 | 0.081 | 0.098 | 0.000114484390287738 |
| GBP2 | 4.78785783595482e-09 | 0.205664216042129 | 0.346 | 0.408 | 0.000118078149950318 |
| NECAP1 | 4.90614194959117e-09 | 0.144642219465184 | 0.058 | 0.071 | 0.000120995272760817 |
| TGFBR2 | 4.95715902603528e-09 | 0.270655765706576 | 0.162 | 0.19 | 0.000122253455900082 |
| EXOC1 | 4.97296819637844e-09 | 0.285283630620496 | 0.073 | 0.088 | 0.000122643341659085 |
| SPG7 | 5.11094057119837e-09 | 0.230622054815784 | 0.105 | 0.124 | 0.000126046016366894 |
| MIDN | 5.12500915458382e-09 | 0.231316212563669 | 0.176 | 0.205 | 0.000126392975770346 |
| LPIN1 | 5.1420150426945e-09 | 0.171810786524691 | 0.098 | 0.116 | 0.000126812374982932 |
| ARIH1 | 5.15437077102033e-09 | 0.585363812605007 | 0.142 | 0.131 | 0.000127117091954903 |
| LRIG1 | 5.20719563628524e-09 | 0.711813202658441 | 0.057 | 0.047 | 0.000128419858782067 |
| SYTL3 | 5.23735693893031e-09 | 0.167215767580761 | 0.201 | 0.232 | 0.000129163696827899 |
| PIGG | 5.38803755522989e-09 | 0.174496161319745 | 0.058 | 0.071 | 0.00013287978218708 |
| TNFSF10 | 5.43245387999359e-09 | 0.535323663880767 | 0.137 | 0.163 | 0.000133975177588402 |
| GBF1 | 5.53136307503209e-09 | 0.261994707195993 | 0.126 | 0.149 | 0.000136414476156441 |

| Gene | P_val | Avg_log2FC | Pct. 1 | Pct. 2 | P_val_adj |
|---|---|---|---|---|---|
| STMN3 | 5.55339786907207e-09 | 0.101710631566952 | 0.061 | 0.075 | 0.000136957898247055 |
| ATG16L2 | 5.6059473709044e-09 | 0.266749822668225 | 0.108 | 0.128 | 0.000138253874061244 |
| LINC01184 | 5.63632262033759e-09 | 0.303175622659003 | 0.039 | 0.05 | 0.000139002988462766 |
| ZNF518A | 5.72678559469313e-09 | 0.193055237190519 | 0.061 | 0.074 | 0.000141233986336322 |
| UTY | 5.92095105268464e-09 | 0.699114915747295 | 0.091 | 0.08 | 0.000146022494861308 |
| ACOT9 | 5.93913630512269e-09 | 0.307238168506477 | 0.09 | 0.108 | 0.000146470979556936 |
| CD28 | 5.94477676602352e-09 | 0.730966881332076 | 0.075 | 0.064 | 0.000146610084603672 |
| PDPR | 6.31938923570215e-09 | 0.107520560530527 | 0.062 | 0.076 | 0.000155848777330887 |
| TIPARP | 6.60755552063786e-09 | 0.285248727521833 | 0.154 | 0.181 | 0.000162955534249971 |
| ITGA1 | 6.64580198286097e-09 | 0.166993385894204 | 0.077 | 0.092 | 0.000163898768501317 |
| STK40 | 6.65628780500142e-09 | 0.249783239014608 | 0.082 | 0.098 | 0.000164157369846945 |
| USP4 | 6.73756521009506e-09 | 0.220082949828679 | 0.142 | 0.166 | 0.000166161833211364 |
| DHX36 | 6.75529310599182e-09 | 0.241248919036777 | 0.217 | 0.254 | 0.00016659903857997 |
| ANKRD27 | 6.78482361635392e-09 | 0.134934872102627 | 0.042 | 0.053 | 0.00016732732002652 |
| CCNL2 | 7.05857372140511e-09 | 0.174410546157795 | 0.167 | 0.194 | 0.000174078545117293 |
| ITPR2 | 7.61439658989592e-09 | 0.542814841891983 | 0.123 | 0.111 | 0.000187786248700013 |
| DENND1B | 8.10763934198489e-09 | 0.287990563103346 | 0.101 | 0.12 | 0.000199950601452031 |
| RBM26 | 8.16060950164135e-09 | 0.20896275473977 | 0.161 | 0.189 | 0.000201256951529479 |
| RIPK2 | 8.23415333106186e-09 | 0.272853305934531 | 0.054 | 0.067 | 0.000203070689450648 |
| ANKRD36C | 8.50515456120886e-09 | 0.401854593871348 | 0.125 | 0.148 | 0.000209754121788533 |
| WDR11 | 8.58055772585182e-09 | 0.170191493472377 | 0.061 | 0.075 | 0.000211613714634958 |
| ZBTB43 | 8.58393703612062e-09 | 0.228357789744566 | 0.071 | 0.085 | 0.000211697055184807 |
| ADAT1 | 9.60794029206951e-09 | 0.211418191650742 | 0.066 | 0.08 | 0.000236951023483018 |
| ATAD2B | 9.61898716045273e-09 | 0.564462707268911 | 0.096 | 0.085 | 0.000237223461351085 |
| MICU1 | 9.73542545405804e-09 | 0.352786995804371 | 0.075 | 0.09 | 0.000240095062547979 |
| TRAPPC11 | 9.74993251320146e-09 | 0.292424271548783 | 0.062 | 0.076 | 0.000240452835640574 |
| AOAH | 9.75793056656348e-09 | 0.475385246595602 | 0.163 | 0.152 | 0.000240650083632589 |
| FCHO1 | 9.78074047994907e-09 | 0.243961159298719 | 0.061 | 0.074 | 0.000241212621716504 |
| CD46 | 1.00531507400383e-08 | 0.230484949280305 | 0.214 | 0.249 | 0.000247930803550825 |
| CSNK1G3 | 1.0285665269837e-08 | 0.275625475015087 | 0.091 | 0.109 | 0.000253665076884721 |
| NCOA6 | 1.03570475234765e-08 | 0.13964623279489 | 0.085 | 0.101 | 0.000255425506023979 |
| WBP1L | 1.06048486339676e-08 | 0.11747124393689 | 0.066 | 0.08 | 0.000261536777010909 |

| Gene | P_val | Avg_log2FC | Pct.1 | Pct.2 | P_val_adj |
|---|---|---|---|---|---|
| AC012615.1 | 1.07089107024859e-08 | 0.104701922226744 | 0.091 | 0.108 | 0.000264103155744708 |
| ANKZF1 | 1.10165916675103e-08 | 0.129651138126982 | 0.074 | 0.089 | 0.000271691183704138 |
| ZNF791 | 1.12320151414334e-08 | 0.111195916358398 | 0.073 | 0.087 | 0.000277003957418032 |
| MFN1 | 1.18765291231488e-08 | 0.219354320833248 | 0.047 | 0.059 | 0.000292898961235097 |
| GGA3 | 1.20045243399962e-08 | 0.287843085869353 | 0.093 | 0.111 | 0.000296055579272987 |
| HLA-DRB5 | 1.22135126011127e-08 | 0.208150276466679 | 0.112 | 0.132 | 0.000301209647768643 |
| SBF2 | 1.23296718598692e-08 | 0.800157554678537 | 0.083 | 0.073 | 0.000304074367408094 |
| EDEM3 | 1.25021420056936e-08 | 0.210358691652026 | 0.05 | 0.062 | 0.000308327826144415 |
| EHD4 | 1.27227881108721e-08 | 0.236909293935746 | 0.044 | 0.055 | 0.000313769400390329 |
| ALG13 | 1.3009855435855e-08 | 0.250750984733262 | 0.079 | 0.095 | 0.000320849054759055 |
| STAG1 | 1.32736044105697e-08 | 0.317137343654878 | 0.173 | 0.204 | 0.000327353631973469 |
| STAMBPL1 | 1.40024870831106e-08 | 0.247723092114371 | 0.048 | 0.06 | 0.000345329336443673 |
| FAM49A | 1.54842994987105e-08 | 0.344316902601838 | 0.077 | 0.093 | 0.000381873794237199 |
| SLC39A11 | 1.66841802110165e-08 | 0.681915244164374 | 0.059 | 0.049 | 0.000411465252364089 |
| FBXO3 | 1.68998311616162e-08 | 0.206822608140689 | 0.062 | 0.076 | 0.000416783636107778 |
| TMEM175 | 1.77894526412342e-08 | 0.237077529628797 | 0.074 | 0.089 | 0.000438723481038118 |
| USPL1 | 1.82248591840531e-08 | 0.261214026892633 | 0.079 | 0.095 | 0.000449461477197117 |
| ITGB2-AS1 | 1.89530895897122e-08 | 0.137581267551946 | 0.214 | 0.245 | 0.000467421095461482 |
| C2CD5 | 1.94388701662572e-08 | 0.342383004361398 | 0.061 | 0.074 | 0.000479401416040234 |
| RLIM | 1.94539593207478e-08 | 0.291141514768793 | 0.063 | 0.077 | 0.000479773544768283 |
| RNF214 | 1.94823477938535e-08 | 0.204399771579744 | 0.053 | 0.065 | 0.000480473661292015 |
| MCOLN2 | 1.95286389735369e-08 | 0.248226262000733 | 0.102 | 0.121 | 0.000481615294365366 |
| NLRC3 | 2.12158712727978e-08 | 0.169861170309845 | 0.108 | 0.127 | 0.000523225817329741 |
| GPATCH2 | 2.25777008628527e-08 | 0.275063240217394 | 0.047 | 0.059 | 0.000556811258679672 |
| ITFG2 | 2.36329151148353e-08 | 0.164833545291168 | 0.069 | 0.083 | 0.000582834952562067 |
| PRDM2 | 2.44016528694252e-08 | 0.265093622664678 | 0.162 | 0.189 | 0.000601793563065764 |
| CPD | 2.44175797259227e-08 | 0.277507979160724 | 0.078 | 0.093 | 0.000602186351200705 |
| MCU | 2.52561535503437e-08 | 0.288065163911013 | 0.048 | 0.06 | 0.000622867258858575 |
| DDX58 | 2.53350561310647e-08 | 0.251355055679196 | 0.088 | 0.104 | 0.000624813154304317 |
| IL18RAP | 2.61378139704537e-08 | 0.211041297619865 | 0.068 | 0.082 | 0.000644610768139329 |
| ZFAND3 | 2.76667624627935e-08 | 0.556712758726738 | 0.177 | 0.169 | 0.000682317695857413 |
| MARCHF2 | 2.79710127360146e-08 | 0.163307091795547 | 0.07 | 0.084 | 0.000689821116095592 |

| Gene | P_val | Avg_log2FC | Pct.1 | Pct.2 | P_val_adj |
|---|---|---|---|---|---|
| RC3H2 | 2.80630493997691e-08 | 0.224306276538268 | 0.113 | 0.133 | 0.000692090924297107 |
| DGKD | 2.8668892129202e-08 | 0.133008702069922 | 0.121 | 0.141 | 0.000707032217690379 |
| AC079781.5 | 2.86708524951963e-08 | 0.131560713062093 | 0.08 | 0.095 | 0.000707080564236531 |
| ARRDC2 | 2.8837101730456e-08 | 0.120900115802597 | 0.113 | 0.131 | 0.000711180602876506 |
| CDK13 | 2.9656572383729e-08 | 0.188790360393992 | 0.169 | 0.196 | 0.000731390388127524 |
| STRIP1 | 3.01515512823937e-08 | 0.179550324782767 | 0.052 | 0.064 | 0.000743597557726394 |
| KMT2B | 3.06077324596682e-08 | 0.104682156244084 | 0.094 | 0.11 | 0.000754847897920338 |
| AGPAT4 | 3.08472572729875e-08 | 0.204350315300317 | 0.09 | 0.106 | 0.000760755058866418 |
| BANP | 3.18198238533278e-08 | 0.272900502415383 | 0.052 | 0.064 | 0.000784740495870771 |
| GIPC1 | 3.21161189952147e-08 | 0.189820058212243 | 0.07 | 0.084 | 0.000792047726659984 |
| PRDM4 | 3.45207358163999e-08 | 0.272978219889826 | 0.055 | 0.067 | 0.000851350386704055 |
| DEF8 | 3.46146717068929e-08 | 0.261852143931824 | 0.054 | 0.065 | 0.000853667033635393 |
| GABPB1-AS1 | 3.5630967934076e-08 | 0.2123639495546 | 0.061 | 0.074 | 0.000878730931190183 |
| HERC2 | 3.58556948304112e-08 | 0.314697748166664 | 0.127 | 0.149 | 0.0008842731459076 |
| PHIP | 3.60645044495688e-08 | 0.26520042749963 | 0.201 | 0.235 | 0.000889422808735265 |
| FAM8A1 | 3.82484837972118e-08 | 0.239569860247384 | 0.076 | 0.09 | 0.000943284107406838 |
| MAP3K1 | 3.88134056458115e-08 | 0.620824485583144 | 0.104 | 0.093 | 0.000957216210037004 |
| SLF1 | 3.96437952352998e-08 | 0.316728604913633 | 0.066 | 0.08 | 0.000977695278092962 |
| CCDC174 | 3.96723471170531e-08 | 0.278162587811783 | 0.074 | 0.089 | 0.000978399424600764 |
| ESCO1 | 3.98502684983734e-08 | 0.204487925867492 | 0.123 | 0.144 | 0.000982787321706884 |
| NSMAF | 4.21372815221389e-08 | 0.302865458723286 | 0.082 | 0.098 | 0.00103918963689899 |
| ST6GAL1 | 4.33539047365912e-08 | 0.25161486153718 | 0.125 | 0.147 | 0.00106919399861381 |
| LFNG | 4.40106682473834e-08 | 0.275781553728826 | 0.043 | 0.053 | 0.00108539110031697 |
| LYST | 4.68003672403821e-08 | 0.407087692489468 | 0.361 | 0.378 | 0.0011541906568823 |
| FAM160B1 | 4.8010018003602e-08 | 0.227543091678144 | 0.118 | 0.138 | 0.00118402306400483 |
| THOC1 | 4.93449350493942e-08 | 0.37950555568818 | 0.068 | 0.083 | 0.00121694478818816 |
| PNISR | 4.98712291679924e-08 | 0.11460657206461 | 0.406 | 0.473 | 0.00122992425374103 |
| KIDINS220 | 5.32579119453053e-08 | 0.229336136292749 | 0.127 | 0.148 | 0.00131344662439512 |
| SLC25A36 | 5.35417918807621e-08 | 0.220490917611835 | 0.098 | 0.115 | 0.00132044767136335 |
| TUBGCP6 | 5.41528154714849e-08 | 0.128869444315157 | 0.07 | 0.084 | 0.00133551673515776 |
| DNMT3A | 5.44578098728209e-08 | 0.232984147450883 | 0.051 | 0.063 | 0.00134303850708351 |
| COP1 | 5.5287245280264e-08 | 0.671474587166487 | 0.13 | 0.121 | 0.00136349404310187 |

| Gene | P_val | Avg_log2FC | Pct. 1 | Pct. 2 | P_val_adj |
|---|---|---|---|---|---|
| GTPBP1 | 5.53447620137613e-08 | 0.248114400476139 | 0.086 | 0.102 | 0.00136491252078338 |
| ITK | 5.66103585869232e-08 | 0.189307396086971 | 0.23 | 0.267 | 0.0013961246634707 |
| TOP3B | 5.7239825370815e-08 | 0.122833568420762 | 0.042 | 0.052 | 0.00141164857329504 |
| SLC39A10 | 5.80971511228945e-08 | 0.217355553313049 | 0.069 | 0.083 | 0.00143279194099282 |
| PNPLA8 | 6.07391670717163e-08 | 0.345165567134204 | 0.087 | 0.103 | 0.00149794933832267 |
| KMT2C | 6.17536409819456e-08 | 0.455058043279992 | 0.238 | 0.234 | 0.00152296829389674 |
| FOXK1 | 6.23582901744628e-08 | 0.257965599970016 | 0.056 | 0.068 | 0.0015378801522826 |
| MAML1 | 6.60824703374444e-08 | 0.26422621820618 | 0.05 | 0.061 | 0.00162972588346205 |
| KHNYN | 6.73244953861268e-08 | 0.145611348372176 | 0.065 | 0.078 | 0.00166035670521266 |
| RFNG | 6.85815380999417e-08 | 0.166112935053432 | 0.066 | 0.079 | 0.00169135789262076 |
| KLHL18 | 8.15175803290181e-08 | 0.190177210691643 | 0.04 | 0.05 | 0.00201038656607424 |
| RAB2B | 8.36690118757669e-08 | 0.154248571576153 | 0.04 | 0.05 | 0.00206344517088016 |
| HCG18 | 8.5879166857957e-08 | 0.142872926913806 | 0.068 | 0.081 | 0.00211795201305094 |
| PPM1K | 8.7174278766057e-08 | 0.174885915528809 | 0.097 | 0.113 | 0.0021498920629285 |
| VPS13B | 8.78244555160932e-08 | 0.555411212773211 | 0.173 | 0.164 | 0.00216592672193789 |
| GALNT7 | 8.83395971082743e-08 | 0.190298015559071 | 0.045 | 0.056 | 0.00217863114388426 |
| SMYD3 | 9.57439090816242e-08 | 0.807766131945747 | 0.08 | 0.07 | 0.00236123628577102 |
| KANSL3 | 9.63389743408007e-08 | 0.228073488188344 | 0.059 | 0.071 | 0.00237591178519283 |
| RNF166 | 1.02334818446524e-07 | 0.147451142269828 | 0.339 | 0.392 | 0.00252378129252817 |
| AP3B1 | 1.05561048555035e-07 | 0.378893394002379 | 0.155 | 0.182 | 0.00260334657946427 |
| PLEKHG2 | 1.09802300629449e-07 | 0.206172578931161 | 0.057 | 0.069 | 0.00270794433812347 |
| MARK3 | 1.09992582359821e-07 | 0.267850126202147 | 0.207 | 0.24 | 0.00271263706615791 |
| JMJD6 | 1.12523812336847e-07 | 0.229312364470787 | 0.122 | 0.141 | 0.00277506225985131 |
| YTHDC1 | 1.13402793934125e-07 | 0.222397655960568 | 0.245 | 0.284 | 0.00279673970400339 |
| WWP2 | 1.16811722231119e-07 | 0.24849561733465 | 0.096 | 0.113 | 0.00288081069366385 |
| PDSS2 | 1.18639288030195e-07 | 0.345998939684571 | 0.049 | 0.06 | 0.00292588212140068 |
| CLK4 | 1.23149946482546e-07 | 0.181440011712471 | 0.06 | 0.072 | 0.00303712398015254 |
| ZNF75A | 1.25082993251834e-07 | 0.174368313917855 | 0.062 | 0.074 | 0.00308479677957674 |
| SUCO | 1.30717928375647e-07 | 0.322894457419987 | 0.098 | 0.115 | 0.00322376554960022 |
| RGL2 | 1.30996490351364e-07 | 0.107565082011445 | 0.06 | 0.072 | 0.00323063544504534 |
| EML4 | 1.31181017892469e-07 | 0.449333115371391 | 0.315 | 0.325 | 0.00323518626326406 |
| MIATNB | 1.35560838467789e-07 | 0.186447608740831 | 0.045 | 0.056 | 0.00334320139829262 |

| Gene | P_val | Avg_log2FC | Pct.1 | Pct.2 | P_val_adj |
|---|---|---|---|---|---|
| BRF1 | 1.37021858790794e-07 | 0.180356786780731 | 0.057 | 0.068 | 0.00337923308149855 |
| ZFP36 | 1.3794930298069e-07 | 0.393774356747499 | 0.397 | 0.418 | 0.00340210571010978 |
| POLB | 1.38358755814446e-07 | 0.271224127696937 | 0.057 | 0.069 | 0.00341220363589586 |
| BRAF | 1.39929589999342e-07 | 0.628879955090159 | 0.086 | 0.076 | 0.00345094354856378 |
| SUMF1 | 1.44210270793188e-07 | 0.751516379889501 | 0.063 | 0.054 | 0.0035565136983016 |
| MARK4 | 1.51926112226867e-07 | 0.225023459179939 | 0.072 | 0.086 | 0.003746801779739 |
| SAMHD1 | 1.54538850277086e-07 | 0.137396104688936 | 0.454 | 0.533 | 0.0038112371255335 |
| CTSB | 1.57050118352272e-07 | 0.408010460229933 | 0.174 | 0.204 | 0.00387317001880373 |
| GALNT3 | 1.63958936906066e-07 | 0.176126781190973 | 0.05 | 0.061 | 0.0040435553019774 |
| SNIP1 | 1.65304465058433e-07 | 0.268425075693971 | 0.058 | 0.07 | 0.00407673871727106 |
| ERC1 | 1.69935441647039e-07 | 0.58994987482246 | 0.05 | 0.042 | 0.00419094786189927 |
| C18orf25 | 1.70459064086707e-07 | 0.290802096911763 | 0.057 | 0.069 | 0.00420386143850636 |
| LIMK2 | 1.71515728265061e-07 | 1.00541278720972 | 0.075 | 0.066 | 0.00422992089047292 |
| PRKCA | 1.72929119732094e-07 | 0.728068370725417 | 0.058 | 0.049 | 0.00426477795083291 |
| USP9X | 1.7424541558519e-07 | 0.248670431519104 | 0.125 | 0.146 | 0.00429724043916195 |
| TRAPPC6A | 1.75862440619854e-07 | 0.151134973775565 | 0.088 | 0.103 | 0.00433711951056684 |
| ATP9B | 1.8767249039868e-07 | 0.662299157051385 | 0.098 | 0.088 | 0.00462837895821226 |
| PI4KA | 1.89319223856606e-07 | 0.195872922092067 | 0.143 | 0.165 | 0.00466899069875163 |
| RNF216 | 1.94540480805466e-07 | 0.172858512208163 | 0.166 | 0.19 | 0.00479775733762441 |
| SOCS3 | 1.97226998367397e-07 | 0.915824775930939 | 0.054 | 0.045 | 0.00486401223373674 |
| ARFGAP1 | 1.9975055545887e-07 | 0.199145301062938 | 0.052 | 0.063 | 0.00492624819872664 |
| PLK3 | 2.03374815008433e-07 | 0.804554899500143 | 0.055 | 0.047 | 0.00501562968773798 |
| TUG1 | 2.0358768319939e-07 | 0.253544880421657 | 0.116 | 0.135 | 0.00502087944306335 |
| ZEB1 | 2.06468771332286e-07 | 0.862069539992138 | 0.08 | 0.071 | 0.00509193283859684 |
| RILPL2 | 2.13331346581285e-07 | 0.391789581334257 | 0.125 | 0.146 | 0.00526117766938765 |
| BAZ2A | 2.16546520960167e-07 | 0.203430113950945 | 0.193 | 0.222 | 0.00534047029991964 |
| IKBKB | 2.22242045081586e-07 | 0.206588799791908 | 0.096 | 0.113 | 0.00548093331580208 |
| L3MBTL3 | 2.29120219889731e-07 | 0.166242360172381 | 0.047 | 0.057 | 0.00565056286292055 |
| TMEM71 | 2.3287789210401e-07 | 0.378557780367762 | 0.063 | 0.076 | 0.0057432345750691 |
| COMMD10 | 2.34215425723728e-07 | 0.40636030686859 | 0.083 | 0.098 | 0.00577622082919858 |
| CCDC93 | 2.38138350652425e-07 | 0.263450558445657 | 0.076 | 0.09 | 0.0058729680037901 |
| FAM228B | 2.50825881489776e-07 | 0.12843297975595 | 0.052 | 0.062 | 0.00618586788930085 |

| Gene | P_val | Avg_log2FC | Pct. 1 | Pct. 2 | P_val_adj |
|---|---|---|---|---|---|
| MAP4K2 | 2.51518881559967e-07 | 0.230997851412362 | 0.071 | 0.085 | 0.00620295865703191 |
| TTC39C | 2.54234984094127e-07 | 0.283814734588307 | 0.101 | 0.119 | 0.00626994317772936 |
| SESN2 | 2.63239737763157e-07 | 0.143549192780933 | 0.051 | 0.062 | 0.00649201841271497 |
| ACSF3 | 2.78072278075719e-07 | 0.204083871291049 | 0.069 | 0.082 | 0.00685781852190338 |
| MX2 | 2.86139537565015e-07 | 0.628011831047187 | 0.128 | 0.118 | 0.0070567732754284 |
| TRMO | 2.87819536921828e-07 | 0.22624208909105 | 0.047 | 0.057 | 0.00709820541956613 |
| RGL4 | 3.02571263043234e-07 | 0.401610676280764 | 0.07 | 0.083 | 0.00746201248917224 |
| MALT1 | 3.0381586337286e-07 | 0.237444659357723 | 0.127 | 0.148 | 0.00749270682250147 |
| AUH | 3.04413365853748e-07 | 0.288114655149196 | 0.066 | 0.079 | 0.00750744242868514 |
| CERK | 3.0663852557587e-07 | 0.24283840497666 | 0.133 | 0.154 | 0.00756231931775211 |
| YEATS2 | 3.09388292035902e-07 | 0.287150573370909 | 0.053 | 0.064 | 0.00763013405818942 |
| BPTF | 3.16694195049637e-07 | 0.196596619945912 | 0.284 | 0.327 | 0.00781031223831414 |
| ACAP2 | 3.26034842624121e-07 | 0.265502110685551 | 0.266 | 0.31 | 0.00804067128879607 |
| ANKRD28 | 3.27566938935487e-07 | 0.330917906334416 | 0.042 | 0.051 | 0.00807845584802697 |
| ALOX5AP | 3.34397835271249e-07 | 0.682314945230451 | 0.162 | 0.154 | 0.00824691941345955 |
| MARF1 | 3.38370485071427e-07 | 0.241368962548911 | 0.112 | 0.13 | 0.00834489290283154 |
| ASB7 | 3.46363763375025e-07 | 0.280322409031902 | 0.04 | 0.05 | 0.00854202313235487 |
| CEP164 | 3.52194048830144e-07 | 0.248433501471861 | 0.055 | 0.066 | 0.00868580963224901 |
| ZNF217 | 3.53529438103525e-07 | 0.217372942117891 | 0.142 | 0.164 | 0.00871874300250914 |
| RAPGEF1 | 3.57952909588152e-07 | 0.2052854341552 | 0.256 | 0.295 | 0.00882783465626301 |
| ASB3 | 3.77020723591355e-07 | 0.303198395896684 | 0.062 | 0.074 | 0.00929808508520999 |
| FAM160A2 | 3.86041949489465e-07 | 0.129631783012995 | 0.053 | 0.064 | 0.00952056655830918 |
| PSTPIP2 | 3.9904959704841e-07 | 0.259313432052513 | 0.089 | 0.104 | 0.00984136116240788 |
| ARHGAP21 | 4.00380955346722e-07 | 0.261296132245127 | 0.053 | 0.064 | 0.00987419512076086 |
| ELMOD3 | 4.56546869933055e-07 | 0.138575626934057 | 0.054 | 0.065 | 0.011259358906289 |
| TARS3 | 4.64453387266428e-07 | 0.160521438366143 | 0.059 | 0.071 | 0.0114543494367646 |
| SLC2A3 | 4.73613844538574e-07 | 0.519352139553502 | 0.236 | 0.234 | 0.0116802646340103 |
| ZNF644 | 4.92907749546631e-07 | 0.299125697484723 | 0.166 | 0.193 | 0.012156090919319 |
| AREL1 | 4.98174286490844e-07 | 0.273476010052943 | 0.062 | 0.074 | 0.0122859742534372 |
| WASHC2C | 5.04326207201268e-07 | 0.23074035318962 | 0.137 | 0.157 | 0.0124376929219977 |
| PIK3CD | 5.25753925489758e-07 | 0.255321078792002 | 0.18 | 0.208 | 0.0129661433104284 |
| MED13 | 5.44382447765685e-07 | 0.209412091483426 | 0.158 | 0.182 | 0.0134255599267973 |

| Gene | P_val | Avg_log2FC | Pct.1 | Pct.2 | P_val_adj |
|---|---|---|---|---|---|
| SCML4 | 5.46349341607356e-07 | 0.213259203876138 | 0.092 | 0.107 | 0.0134740674627206 |
| TRIM44 | 5.50945742195931e-07 | 0.230187658821875 | 0.092 | 0.108 | 0.013587423894036 |
| INPP5D | 5.55380510977458e-07 | 0.171937915646587 | 0.329 | 0.384 | 0.0136967941617261 |
| MYBL1 | 5.5699476124743e-07 | 0.115588331267009 | 0.094 | 0.11 | 0.0137366048018841 |
| NPEPPS | 5.6739425645325e-07 | 0.283995795936739 | 0.143 | 0.165 | 0.0139930771526501 |
| VPS53 | 5.96338823095837e-07 | 0.268119076104339 | 0.102 | 0.119 | 0.0147069080551895 |
| NT5C2 | 6.10222276595522e-07 | 0.365470411664758 | 0.129 | 0.151 | 0.0150493017853988 |
| GIGYF2 | 6.14575494908055e-07 | 0.225215659748577 | 0.112 | 0.13 | 0.0151566608554225 |
| CD86 | 6.69964490038731e-07 | 0.358891813617862 | 0.041 | 0.05 | 0.0165226642533352 |
| METTL15 | 6.76172957448695e-07 | 0.309272641293133 | 0.044 | 0.054 | 0.0166757774765997 |
| PTEN | 7.11578843916746e-07 | 0.388398050243926 | 0.188 | 0.218 | 0.0175489574486748 |
| STAT6 | 7.23867746940769e-07 | 0.132280341397035 | 0.177 | 0.202 | 0.0178520263750532 |
| DZIP3 | 7.81355267336187e-07 | 0.272428436601752 | 0.052 | 0.063 | 0.019269783603045 |
| ANKRD44 | 7.83928982982561e-07 | 0.451491767028797 | 0.279 | 0.286 | 0.0193332565783159 |
| RUNX2 | 7.92497324876279e-07 | 0.188852447820204 | 0.043 | 0.052 | 0.0195445690260988 |
| MLKL | 7.97534728319581e-07 | 0.383278925246011 | 0.072 | 0.085 | 0.0196688014698175 |
| SLC35E2B | 8.01945035550643e-07 | 0.107770840314804 | 0.068 | 0.08 | 0.01977756846675 |
| NRDE2 | 8.2684199254338e-07 | 0.216733211889603 | 0.043 | 0.053 | 0.0203915772201048 |
| ZMYM4 | 8.47451265141989e-07 | 0.27781800007687 | 0.065 | 0.077 | 0.0208998431009317 |
| KDM2B | 9.0434130664927e-07 | 0.269963824013679 | 0.077 | 0.091 | 0.0223028653045843 |
| KIF13B | 9.12957284388271e-07 | 0.194809419170312 | 0.075 | 0.088 | 0.0225153525475836 |
| APPBP2 | 9.17189137079337e-07 | 0.314184836874258 | 0.075 | 0.088 | 0.0226197184986506 |
| E2F3 | 9.21811154287225e-07 | 0.310238809345119 | 0.087 | 0.103 | 0.0227337066870315 |
| SLC7A5 | 9.72258339847449e-07 | 0.395180905455011 | 0.045 | 0.055 | 0.0239778351773178 |
| RRN3P2 | 9.79126017019816e-07 | 0.124917247821315 | 0.051 | 0.062 | 0.0241472058317427 |
| SERTAD2 | 1.01595499646353e-06 | 0.361688169905898 | 0.051 | 0.061 | 0.0250554821227835 |
| FAM102B | 1.02812384187732e-06 | 0.264597634646551 | 0.045 | 0.055 | 0.0253555901883785 |
| TOB1 | 1.04384907352156e-06 | 0.162818959612574 | 0.103 | 0.119 | 0.0257434058511888 |
| TNRC18 | 1.04497306082851e-06 | 0.177884748269574 | 0.099 | 0.114 | 0.0257711256261528 |
| YBX3 | 1.08087457500136e-06 | 0.336444223904619 | 0.118 | 0.137 | 0.0266565287686835 |
| LY9 | 1.0853902407521e-06 | 0.124584630744417 | 0.084 | 0.097 | 0.0267678941174284 |
| CRACR2A | 1.10905944642446e-06 | 0.263444353325101 | 0.077 | 0.09 | 0.02735162406772 |

| Gene | P_val | Avg_log2FC | Pct.1 | Pct.2 | P_val_adj |
|---|---|---|---|---|---|
| FAM193B | 1.14436577194719e-06 | 0.198371849409426 | 0.096 | 0.111 | 0.0282223486677615 |
| CCNT1 | 1.24736622361952e-06 | 0.189675271848014 | 0.083 | 0.097 | 0.0307625458069046 |
| ARRB1 | 1.25029590104741e-06 | 0.178868828397203 | 0.048 | 0.058 | 0.0308347975116312 |
| NMT2 | 1.28653719898449e-06 | 0.308625075036312 | 0.051 | 0.061 | 0.0317285804013556 |
| CEP192 | 1.30921805053583e-06 | 0.323950899548275 | 0.073 | 0.086 | 0.0322879355623146 |
| VAV3 | 1.34306368572115e-06 | 0.705377894888758 | 0.057 | 0.048 | 0.0331226366172551 |
| MCM9 | 1.34870403143327e-06 | 0.355231233528605 | 0.07 | 0.083 | 0.0332617388232074 |
| RAB3GAP2 | 1.4137526065908e-06 | 0.332231791106562 | 0.092 | 0.107 | 0.0348659667837423 |
| YTHDF3 | 1.42901274186063e-06 | 0.28570722230714 | 0.089 | 0.104 | 0.0352423122397668 |
| PKD2 | 1.43433818340719e-06 | 0.233591511700159 | 0.043 | 0.052 | 0.0353736482791882 |
| TRIM33 | 1.4521929008893le-06 | 0.28086729602262 | 0.147 | 0.169 | 0.0358139813217322 |
| LUC7L | 1.48027256781931e-06 | 0.258548129680532 | 0.132 | 0.152 | 0.0365064820675598 |
| CAPN7 | 1.51687335418002e-06 | 0.281132037072614 | 0.096 | 0.111 | 0.0374091306607878 |
| CDYL | 1.5210937479022e-06 | 0.306138944203086 | 0.059 | 0.07 | 0.037513214010764 |
| BCL6 | 1.55971193693458e-06 | 0.830603128934966 | 0.055 | 0.047 | 0.0384656157886807 |
| SIRT1 | 1.56641259202413e-06 | 0.21959269253949 | 0.053 | 0.063 | 0.038630867344499 |
| RNF213 | 1.57886432024579e-06 | 0.15962365918513 | 0.667 | 0.732 | 0.0389379518659016 |
| RPS6KA3 | 1.63793646936302e-06 | 0.254064406638106 | 0.188 | 0.216 | 0.0403947892074307 |
| HOOK3 | 1.63909982548458e-06 | 0.344135630088009 | 0.123 | 0.142 | 0.0404234798961006 |
| PDZD8 | 1.70780617216286e-06 | 0.257625173211686 | 0.099 | 0.114 | 0.0421179158178804 |
| R3HCC1L | 1.72850166727148e-06 | 0.271084612901642 | 0.082 | 0.096 | 0.0426283081182491 |
| UBXN11 | 1.73104573575856e-06 | 0.270232447829767 | 0.071 | 0.083 | 0.0426910499352775 |
| UBR2 | 1.73476153220876e-06 | 0.182030502175901 | 0.262 | 0.3 | 0.0427826889073325 |
| CDKN1B | 1.74487862325651e-06 | 0.181318348460456 | 0.217 | 0.247 | 0.043032196606752 |
| MDM4 | 1.75247359037978e-06 | 0.246829351069455 | 0.157 | 0.181 | 0.0432195036859462 |
| ARHGAP17 | 1.77614497160607e-06 | 0.297003453787752 | 0.071 | 0.083 | 0.0438032872897489 |
| ESRRA | 1.78663502762581e-06 | 0.219604150033372 | 0.043 | 0.052 | 0.0440619930513076 |
| PCMTD2 | 1.94322346212208e-06 | 0.219812463198406 | 0.078 | 0.091 | 0.0479237770228546 |
| KDM5A | 1.96863544724722e-06 | 0.241061084054004 | 0.246 | 0.283 | 0.0485504874000109 |
| FRAT2 | 1.99114067056246e-06 | 0.407309096359372 | 0.054 | 0.064 | 0.0491055112174114 |
| USP32 | 2.02879491301377e-06 | 0.730521623049152 | 0.077 | 0.069 | 0.0500341401447456 |
| STAG3L5P-PVRIG2P-PILRB | 2.05823766375822e-06 | 0.233474331204184 | 0.056 | 0.066 | 0.0507602572636051 |

| Gene | P_val | Avg_log2FC | Pct.1 | Pct.2 | P_val_adj |
|---|---|---|---|---|---|
| MTMR1 | 2.07493984514311e-06 | 0.331363750790059 | 0.055 | 0.066 | 0.0511721664609193 |
| RIPK3 | 2.21736764481885e-06 | 0.104837772335402 | 0.045 | 0.054 | 0.0546847208565224 |
| MEF2D | 2.25182989602027e-06 | 0.163673131344596 | 0.153 | 0.175 | 0.0555346288956519 |
| MSH3 | 2.26745142211245e-06 | 0.336144551016251 | 0.078 | 0.092 | 0.0559198869721372 |
| PVT1 | 2.27599485963212e-06 | 0.314373647222116 | 0.066 | 0.078 | 0.0561305852282474 |
| PRRC2C | 2.28004027191841e-06 | 0.154150985521043 | 0.443 | 0.515 | 0.0562303531860518 |
| TYW1 | 2.34756035626256e-06 | 0.282587459648026 | 0.048 | 0.058 | 0.0578955335061472 |
| PPM1L | 2.36389351511758e-06 | 0.183842469204895 | 0.049 | 0.059 | 0.0582983418698298 |
| MAP3K4 | 2.36739404780661e-06 | 0.317003544843821 | 0.056 | 0.066 | 0.0583846720070067 |
| MANBA | 2.37126314720156e-06 | 0.643066961487441 | 0.082 | 0.073 | 0.0584800917362849 |
| LINC01550 | 2.40629423129557e-06 | 0.243147701605488 | 0.045 | 0.055 | 0.0593440283322113 |
| MAPK8 | 2.51610723715078e-06 | 0.76081050442222 | 0.067 | 0.059 | 0.0620522366826126 |
| AC022966.1 | 2.60623858818943e-06 | 0.528608557142992 | 0.151 | 0.143 | 0.0642750560619277 |
| DYM | 2.61144482911463e-06 | 0.261820284143363 | 0.102 | 0.119 | 0.0644034523756251 |
| BRWD1 | 2.61501243278167e-06 | 0.294197779110059 | 0.173 | 0.199 | 0.0644914366172616 |
| D2HGDH | 2.61676994213761e-06 | 0.11153787211598 | 0.064 | 0.075 | 0.0645347803129978 |
| DIAPH2 | 2.64387701316591e-06 | 0.597899498056147 | 0.11 | 0.101 | 0.0652032948986977 |
| FYB1 | 2.65480644520882e-06 | 0.186233596310626 | 0.618 | 0.681 | 0.06547283655174 |
| MINDY3 | 2.66153237510586e-06 | 0.245364220188869 | 0.07 | 0.082 | 0.0656387114348606 |
| IFITM2 | 2.66237896044461e-06 | 0.126225722542892 | 0.727 | 0.788 | 0.0656595899224849 |
| RORA | 2.6803993000152e-06 | 0.23481003482135 | 0.258 | 0.298 | 0.066104007536975 |
| RASGRP1 | 2.68407743977678e-06 | 0.204676372347723 | 0.234 | 0.269 | 0.0661947178197748 |
| CUL9 | 2.76491804590547e-06 | 0.191252444699718 | 0.05 | 0.06 | 0.0681884088481207 |
| MAP2K5 | 2.8021252883484e-06 | 0.281684454599914 | 0.056 | 0.066 | 0.0691060138612481 |
| SCAF8 | 2.85395817656042e-06 | 0.314513738094686 | 0.12 | 0.138 | 0.0703843165503331 |
| ZRANB1 | 3.10016317815008e-06 | 0.269256051012085 | 0.053 | 0.063 | 0.0764562242995373 |
| TLE3 | 3.10720391852586e-06 | 0.389566572684338 | 0.051 | 0.061 | 0.0766298630386848 |
| NSUN3 | 3.11385936334113e-06 | 0.333827658798374 | 0.051 | 0.061 | 0.0767939996187191 |
| TCF20 | 3.15337445742118e-06 | 0.196314605760816 | 0.092 | 0.106 | 0.0777685208689212 |
| GIGYF1 | 3.22455297201636e-06 | 0.117572001695639 | 0.079 | 0.091 | 0.0795239253958674 |
| MYO5A | 3.28036619920691e-06 | 0.364162614063093 | 0.108 | 0.125 | 0.0809003912048408 |
| NAPRT | 3.30723005326258e-06 | 0.169452443601153 | 0.053 | 0.063 | 0.0815629075735617 |

| Gene | P_val | Avg_log2FC | Pct.1 | Pct.2 | P_val_adj |
|---|---|---|---|---|---|
| ELMO2 | 3.34778473434883e-06 | 0.251359424648284 | 0.073 | 0.085 | 0.0825630671185108 |
| DHRS1 | 3.46598204405286e-06 | 0.181935944650633 | 0.053 | 0.063 | 0.0854780491704317 |
| RABEP2 | 3.51979230393882e-06 | 0.229246545890031 | 0.059 | 0.07 | 0.0868051177997392 |
| NSMCE2 | 3.52080060555895e-06 | 0.385543994717151 | 0.093 | 0.109 | 0.0868299845342947 |
| PTBP2 | 3.60632958054578e-06 | 0.630239607318249 | 0.07 | 0.062 | 0.0889393001154199 |
| TBC1D4 | 3.71690894799152e-06 | 0.652804148332771 | 0.084 | 0.076 | 0.0916664084753669 |
| STAT5B | 3.75381010593528e-06 | 0.223136787346551 | 0.155 | 0.178 | 0.0925764648325759 |
| SEC14L1 | 3.87017843503962e-06 | 0.431754938811926 | 0.106 | 0.123 | 0.095446340564947 |
| XIST | 4.07799571147726e-06 | 0.268495138686666 | 0.077 | 0.09 | 0.100571530236452 |
| UBR4 | 4.2529319695255e-06 | 0.226024953810394 | 0.123 | 0.141 | 0.104885808232438 |
| TSPO | 4.49315593154317e-06 | 0.35614803326272 | 0.316 | 0.367 | 0.110810211583718 |
| FGL2 | 4.60255714818065e-06 | 0.714770139636914 | 0.134 | 0.127 | 0.113508264388431 |
| AGO1 | 4.7364921338613e-06 | 0.225855536289957 | 0.041 | 0.05 | 0.116811369005287 |
| CTSH | 4.74120120678672e-06 | 0.360944737014377 | 0.076 | 0.088 | 0.116927504161774 |
| KIAA2026 | 4.78939521436847e-06 | 0.155564783514659 | 0.13 | 0.148 | 0.118116064776755 |
| PAG1 | 4.84917837045552e-06 | 0.592299742105578 | 0.191 | 0.188 | 0.119590436972174 |
| ODR4 | 4.9654678023053e-06 | 0.215664994352857 | 0.056 | 0.067 | 0.122458366940453 |
| DLGAP4 | 4.9706460875824e-06 | 0.149255102180088 | 0.063 | 0.073 | 0.122586073811957 |
| FBXL17 | 5.01459039744273e-06 | 0.560763217971806 | 0.083 | 0.075 | 0.123669828381733 |
| AGTPBP1 | 5.1185596592013e-06 | 0.56028154989829 | 0.204 | 0.202 | 0.126233918315222 |
| DYNLT1 | 5.19675606550285e-06 | 0.155689852027186 | 0.325 | 0.372 | 0.128162398087431 |
| PRKCB | 5.69335364897808e-06 | 0.219538037821407 | 0.411 | 0.482 | 0.140409487691097 |
| RALB | 6.05926939445685e-06 | 0.51972277721963 | 0.051 | 0.061 | 0.149433701806095 |
| CD69 | 6.28219671496709e-06 | 0.194722562352691 | 0.306 | 0.35 | 0.154931535384518 |
| NCOA2 | 6.31218943294765e-06 | 0.499280521049481 | 0.145 | 0.138 | 0.155671215795355 |
| DLG1 | 6.36646358419677e-06 | 0.33906449169076 | 0.116 | 0.133 | 0.157009724913461 |
| NUP98 | 6.52143307807418e-06 | 0.385551658600601 | 0.12 | 0.138 | 0.160831582571465 |
| SLC30A1 | 6.64283265091093e-06 | 0.16746592928132 | 0.102 | 0.117 | 0.163825538836765 |
| SLC17A5 | 7.00591004393229e-06 | 0.124083316556265 | 0.055 | 0.065 | 0.172779753503458 |
| ZNF431 | 7.11351578480057e-06 | 0.205293272086415 | 0.047 | 0.056 | 0.175433526284752 |
| VPS13A | 7.5077319829062e-06 | 0.229062875008945 | 0.153 | 0.174 | 0.185155686162433 |
| MLLT10 | 7.65399853395419e-06 | 0.256845269423554 | 0.082 | 0.095 | 0.188762911844378 |

| Gene | P_val | Avg_log2FC | Pct.1 | Pct.2 | P_val_adj |
|---|---|---|---|---|---|
| INO80 | 7.83271541945346e-06 | 0.227292905944082 | 0.088 | 0.102 | 0.193170427674561 |
| AL590764.2 | 7.90113081275729e-06 | 0.111301904510334 | 0.043 | 0.052 | 0.19485768810422 |
| CLK1 | 8.03918490160838e-06 | 0.231017151467367 | 0.268 | 0.308 | 0.198262378043466 |
| RAD51B | 8.21541477292021e-06 | 0.712810989068264 | 0.054 | 0.047 | 0.202608559129758 |
| ANO6 | 8.57184010729133e-06 | 0.343636784429597 | 0.07 | 0.082 | 0.211398720726019 |
| PLEC | 8.91541320848847e-06 | 0.146866268907252 | 0.098 | 0.112 | 0.219871920547743 |
| PUM1 | 8.99174516907136e-06 | 0.290955478983074 | 0.133 | 0.152 | 0.221754419359638 |
| HERC5 | 9.00287866333983e-06 | 0.407672587661329 | 0.049 | 0.058 | 0.222028993595287 |
| MAU2 | 9.02397714084798e-06 | 0.215406635390262 | 0.089 | 0.102 | 0.222549324247593 |
| SRPK2 | 9.05904797639196e-06 | 0.254432325019911 | 0.249 | 0.287 | 0.223414241193779 |
| ZNF266 | 9.18519064401046e-06 | 0.194110091050381 | 0.074 | 0.085 | 0.226525171662586 |
| PHF12 | 9.38144955292143e-06 | 0.24887987917961 | 0.122 | 0.139 | 0.231365308874148 |
| TRPC4AP | 9.51703054328867e-06 | 0.215043318342125 | 0.194 | 0.22 | 0.234709007258585 |
| JMJD1C | 9.57236084023093e-06 | 0.502069697006206 | 0.226 | 0.226 | 0.236073563041775 |
| MBNL2 | 9.65916085675017e-06 | 0.308813656150779 | 0.066 | 0.077 | 0.238214225049173 |
| MBNL1 | 9.8298972136432e-06 | 0.104808774679241 | 0.563 | 0.654 | 0.242424925082869 |
| BRD1 | 1.00466670114394e-05 | 0.405319724466103 | 0.121 | 0.113 | 0.247770901836117 |
| FNDC3A | 1.0059267731568e-05 | 0.365200135072875 | 0.12 | 0.138 | 0.248081660795931 |
| CTNNB1 | 1.02502244938036e-05 | 0.307363391410503 | 0.113 | 0.13 | 0.252791036466184 |
| KMT5B | 1.07115911510111e-05 | 0.186698353761365 | 0.095 | 0.109 | 0.264169260966236 |
| SNRK | 1.09914745770449e-05 | 0.253686427488879 | 0.175 | 0.199 | 0.271071746019082 |
| AGO4 | 1.11253893408311e-05 | 0.519904158191854 | 0.095 | 0.087 | 0.274374351923577 |
| SKAP1 | 1.11355206827428e-05 | 0.300836238402757 | 0.372 | 0.435 | 0.274624211077804 |
| LIMD1 | 1.15554111877778e-05 | 0.251945485194599 | 0.047 | 0.056 | 0.284979550712976 |
| RBAK | 1.19342172786214e-05 | 0.20205350576544 | 0.042 | 0.051 | 0.29432166652536 |
| PPARA | 1.21139530288002e-05 | 0.132155490218967 | 0.062 | 0.072 | 0.298754309596272 |
| WDR48 | 1.26252161298814e-05 | 0.284097717732254 | 0.087 | 0.1 | 0.311363080195135 |
| ITPRIP | 1.26891032607024e-05 | 0.191281988874486 | 0.088 | 0.101 | 0.312938664615442 |
| YPEL3 | 1.27521280409974e-05 | 0.121074550130922 | 0.298 | 0.337 | 0.314492981747078 |
| GPR89B | 1.28060033633915e-05 | 0.25252783027235 | 0.044 | 0.052 | 0.315821654947962 |
| SETD1B | 1.32377614706274e-05 | 0.121197661721715 | 0.092 | 0.104 | 0.326469673388614 |
| PGS1 | 1.38946891113585e-05 | 0.418469654072732 | 0.062 | 0.073 | 0.342670822864323 |

| Gene | P_val | Avg_log2FC | Pct. 1 | Pct. 2 | P_val_adj |
|---|---|---|---|---|---|
| SLMAP | 1.43721064897882e-05 | 0.355949916102127 | 0.107 | 0.123 | 0.354444890251156 |
| TMEM123 | 1.48439716507774e-05 | 0.369564409456534 | 0.204 | 0.236 | 0.366082028851472 |
| PPFIA1 | 1.56553594856014e-05 | 0.209194966957447 | 0.085 | 0.098 | 0.386092475633901 |
| NRF1 | 1.56586304879001e-05 | 0.275862389366939 | 0.063 | 0.074 | 0.386173145092593 |
| APC | 1.57616741322335e-05 | 0.191422502544174 | 0.086 | 0.098 | 0.388714407449143 |
| SBNO2 | 1.58797639960989e-05 | 0.106294626950881 | 0.104 | 0.118 | 0.391626739671792 |
| PCED1B | 1.59715313085701e-05 | 0.225034419804017 | 0.136 | 0.155 | 0.393889905131956 |
| TBC1D23 | 1.62121649732535e-05 | 0.320677944071681 | 0.064 | 0.075 | 0.399824412570378 |
| ODF2L | 1.65433645574526e-05 | 0.220704499162648 | 0.157 | 0.179 | 0.407992456715897 |
| ZNF160 | 1.65606358916815e-05 | 0.140073663180649 | 0.049 | 0.058 | 0.40841840236065 |
| SIN3B | 1.71954576788915e-05 | 0.231260374439481 | 0.063 | 0.074 | 0.424074377276823 |
| PLCG1 | 1.72824431754442e-05 | 0.254009907026648 | 0.1 | 0.115 | 0.426219613592805 |
| ERGIC1 | 1.7487228152344e-05 | 0.280741799748752 | 0.08 | 0.092 | 0.431270020693108 |
| NEK7 | 1.81743854351929e-05 | 0.418787558944596 | 0.097 | 0.112 | 0.448216693602726 |
| FRYL | 1.85722407726758e-05 | 0.256147692458261 | 0.165 | 0.188 | 0.458028601935731 |
| NR1D2 | 1.8703402597136e-05 | 0.119692481519439 | 0.074 | 0.085 | 0.461263314850569 |
| PDE4DIP | 1.90243490220557e-05 | 0.226421179518691 | 0.043 | 0.051 | 0.469178495581938 |
| RNF169 | 1.91054754828972e-05 | 0.288467363160444 | 0.083 | 0.095 | 0.47117923635921 |
| TMEM131 | 1.94679000626716e-05 | 0.311180041093084 | 0.12 | 0.137 | 0.480117351345607 |
| SMAD7 | 1.97082396461167e-05 | 0.150020196064902 | 0.045 | 0.053 | 0.48604460615253 |
| TNKS2 | 1.99048447804199e-05 | 0.249202088744311 | 0.153 | 0.174 | 0.490893281974716 |
| PATL2 | 1.99523648885989e-05 | 0.15641829786304 | 0.2 | 0.224 | 0.492065222882625 |
| NSF | 2.00594948827655e-05 | 0.551180002247336 | 0.2 | 0.198 | 0.494707262798762 |
| SECISBP2 | 2.0104327283377e-05 | 0.292617087056839 | 0.126 | 0.144 | 0.495812919462644 |
| RALGAPB | 2.01570271270937e-05 | 0.291454964864397 | 0.08 | 0.092 | 0.497112603008385 |
| RERE | 2.12412911347126e-05 | 0.547772743015206 | 0.074 | 0.067 | 0.523852721964282 |
| PLEKHM2 | 2.20720392656374e-05 | 0.251573301383813 | 0.116 | 0.131 | 0.54434063236915 |
| ATG7 | 2.26839354482848e-05 | 0.628563923024273 | 0.089 | 0.082 | 0.559431216025599 |
| TUT7 | 2.30616299084906e-05 | 0.298499690863687 | 0.208 | 0.238 | 0.568745916803196 |
| CCNY | 2.31557117154103e-05 | 0.328589183569004 | 0.122 | 0.14 | 0.57106616232545 |
| TMEM62 | 2.36488057772966e-05 | 0.394387356786272 | 0.048 | 0.056 | 0.583226848079688 |
| CACUL1 | 2.41048881251855e-05 | 0.451702472472395 | 0.072 | 0.083 | 0.594474750943326 |

| Gene | P_val | Avg_log2FC | Pct.1 | Pct.2 | P_val_adj |
|---|---|---|---|---|---|
| TBC1D14 | 2.44497772147543e-05 | 0.341459201301817 | 0.075 | 0.087 | 0.602980405670271 |
| RC3H1 | 2.50295605094181e-05 | 0.32128652509878 | 0.118 | 0.135 | 0.617279021283269 |
| RNGTT | 2.56861639079872e-05 | 0.403404369892848 | 0.068 | 0.08 | 0.63347217429878 |
| SPPL3 | 2.56932822013768e-05 | 0.325025397447939 | 0.081 | 0.094 | 0.633647725650355 |
| LRCH1 | 2.5715096728906e-05 | 0.309950665153599 | 0.044 | 0.052 | 0.63418571552828 |
| ADA2 | 2.64781339794648e-05 | 0.255744450362213 | 0.137 | 0.155 | 0.653003740201562 |
| ZBED5 | 2.74945332082154e-05 | 0.184403606199976 | 0.045 | 0.053 | 0.678070177981009 |
| ZNF302 | 2.94858457552441e-05 | 0.231650530488731 | 0.043 | 0.051 | 0.727179928015831 |
| GOLGA1 | 3.22866212384463e-05 | 0.298169628979894 | 0.055 | 0.064 | 0.796252652982563 |
| ATG2B | 3.24140331441605e-05 | 0.172459636866237 | 0.05 | 0.059 | 0.799394885401286 |
| SMURF1 | 3.53346038769215e-05 | 0.718363610417975 | 0.051 | 0.044 | 0.871422000812638 |
| DUSP5 | 3.58095803014008e-05 | 0.277924102334762 | 0.073 | 0.085 | 0.883135869393145 |
| ARHGAP15 | 3.6288800310188e-05 | 0.558068957497025 | 0.351 | 0.376 | 0.894954393249856 |
| NAA25 | 3.67712839596821e-05 | 0.426040477922482 | 0.044 | 0.053 | 0.906853405013681 |
| OSBPL7 | 3.68674531447052e-05 | 0.125864861002087 | 0.109 | 0.123 | 0.90922512945472 |
| TAF1 | 3.74966565522793e-05 | 0.281173204324729 | 0.083 | 0.095 | 0.924742543892311 |
| CHD9 | 3.76618795687919e-05 | 0.276426835553847 | 0.128 | 0.145 | 0.928817273925547 |
| BORCS5 | 3.93122000333913e-05 | 0.302474152785974 | 0.053 | 0.063 | 0.969517477223496 |
| ZFYVE27 | 3.9400880790716e-05 | 0.121207368322856 | 0.042 | 0.05 | 0.971704522060638 |
| SELENOO | 4.02319280377063e-05 | 0.170363626764592 | 0.076 | 0.087 | 0.992199809265913 |
| PRKCD | 4.03369938192732e-05 | 0.39704633957422 | 0.057 | 0.067 | 0.994790941570916 |
| PLXNC1 | 4.0818384077878e-05 | 0.817456868181441 | 0.065 | 0.058 | 1 |
| POGZ | 4.1131090887586e-05 | 0.238583539792618 | 0.059 | 0.069 | 1 |
| SZT2 | 4.14611163950508e-05 | 0.132673352381924 | 0.056 | 0.065 | 1 |
| PLCL2 | 4.15155612658936e-05 | 0.281820754147183 | 0.161 | 0.183 | 1 |
| MAP3K14 | 4.17556277881451e-05 | 0.262480044733809 | 0.066 | 0.076 | 1 |
| ARHGAP4 | 4.20887060148984e-05 | 0.114170402543851 | 0.352 | 0.393 | 1 |
| PLAC8 | 4.2871255702986e-05 | 0.462128342757685 | 0.226 | 0.259 | 1 |
| PAPOLG | 4.29471115074314e-05 | 0.310510309601329 | 0.049 | 0.058 | 1 |
| WDR59 | 4.3290166597561e-05 | 0.196511253778281 | 0.056 | 0.065 | 1 |
| ZHX2 | 4.34885416990766e-05 | 0.6095754708606 | 0.058 | 0.052 | 1 |
| PHYKPL | 4.35593462931903e-05 | 0.176489888599484 | 0.142 | 0.159 | 1 |

| Gene | P_val | Avg_log2FC | Pct. 1 | Pct. 2 | P_val_adj |
|---|---|---|---|---|---|
| PCSK7 | 4.50700822306059e-05 | 0.198805355884498 | 0.211 | 0.239 | 1 |
| AUTS2 | 4.52873627363825e-05 | 0.21400149553081 | 0.076 | 0.087 | 1 |
| NDEL1 | 4.67560428063643e-05 | 0.342200402860765 | 0.09 | 0.103 | 1 |
| CRY1 | 4.83729756757781e-05 | 0.434906511476512 | 0.048 | 0.057 | 1 |
| FOXN3 | 4.90521264446508e-05 | 0.221863892694967 | 0.223 | 0.252 | 1 |
| ARID4A | 4.97223032631133e-05 | 0.238316196870578 | 0.129 | 0.146 | 1 |
| SP100 | 5.00850725335998e-05 | 0.12745234338122 | 0.487 | 0.565 | 1 |
| ANKRD13D | 5.20891645316893e-05 | 0.148218892470476 | 0.146 | 0.163 | 1 |
| GPBP1L1 | 5.28095937571239e-05 | 0.259243551174072 | 0.144 | 0.163 | 1 |
| DDX3Y | 5.35261241952107e-05 | 0.290376360625132 | 0.122 | 0.139 | 1 |
| IFI44 | 5.35432477577221e-05 | 0.679042606557539 | 0.102 | 0.095 | 1 |
| CEMIP2 | 5.4224765927835e-05 | 0.371900407063682 | 0.263 | 0.266 | 1 |
| ATP2B1 | 5.60799257231765e-05 | 0.563560343564765 | 0.185 | 0.183 | 1 |
| WDR37 | 5.62442068475145e-05 | 0.259964233176245 | 0.095 | 0.108 | 1 |
| TENT2 | 5.84460743941442e-05 | 0.28852753420006 | 0.149 | 0.169 | 1 |
| SCFD2 | 5.84630603469843e-05 | 0.580486874046044 | 0.044 | 0.052 | 1 |
| OGFRL1 | 5.85061807331434e-05 | 0.40148463365437 | 0.069 | 0.08 | 1 |
| NABP1 | 5.89984249018339e-05 | 0.758752330059452 | 0.057 | 0.05 | 1 |
| PIM3 | 5.95629774269741e-05 | 0.316632958506774 | 0.14 | 0.159 | 1 |
| PARP6 | 6.04787801048596e-05 | 0.197657294008954 | 0.057 | 0.067 | 1 |
| FNBP4 | 6.09550352798085e-05 | 0.235058334383367 | 0.271 | 0.309 | 1 |
| MAP2K4 | 6.59569662838768e-05 | 0.27159619481784 | 0.072 | 0.083 | 1 |
| ENTPD4 | 6.63413360592704e-05 | 0.25290534282337 | 0.129 | 0.145 | 1 |
| LINC00426 | 6.84522638790772e-05 | 0.201388105630874 | 0.052 | 0.061 | 1 |
| KLF12 | 7.07071547474735e-05 | 0.750331231056394 | 0.15 | 0.146 | 1 |
| PISD | 7.52962105914159e-05 | 0.323711180611252 | 0.046 | 0.055 | 1 |
| NR4A2 | 7.86864089331451e-05 | 0.463997173274271 | 0.107 | 0.099 | 1 |
| AMPD2 | 8.11816896399752e-05 | 0.350817372929226 | 0.049 | 0.058 | 1 |
| RBM5 | 8.1913774853775e-05 | 0.254201306350165 | 0.151 | 0.17 | 1 |
| IFI27 | 8.29515097006861e-05 | 0.668963636002627 | 0.095 | 0.088 | 1 |
| NPIPB5 | 8.42950172164194e-05 | 0.552953407210361 | 0.07 | 0.064 | 1 |
| AGO3 | 8.652721447906e-05 | 0.241476567285521 | 0.078 | 0.089 | 1 |

| Gene | P_val | Avg_log2FC | Pct. 1 | Pct. 2 | P_val_adj |
|---|---|---|---|---|---|
| SH2B3 | 8.75329128987698e-05 | 0.546436788904091 | 0.067 | 0.06 | 1 |
| C16orf72 | 9.09093757093513e-05 | 0.313808722493472 | 0.061 | 0.071 | 1 |
| ANTXR2 | 9.22284202833214e-05 | 0.422542177120051 | 0.068 | 0.078 | 1 |
| MPP1 | 9.22418144840049e-05 | 0.552531444524119 | 0.052 | 0.06 | 1 |
| NFKB1 | 9.28660769936042e-05 | 0.44732986520157 | 0.12 | 0.138 | 1 |
| TAGAP | 9.29213515242242e-05 | 0.255728020939302 | 0.114 | 0.129 | 1 |
| MAN2A1 | 9.41347012841785e-05 | 0.317586213519051 | 0.122 | 0.138 | 1 |
| CXCR4 | 9.48795418008259e-05 | 0.228887519725099 | 0.299 | 0.342 | 1 |
| TCAF2 | 9.64236922350331e-05 | 0.273688543776663 | 0.101 | 0.114 | 1 |
| TRIM4 | 0.000102391049494999 | 0.230361000150195 | 0.069 | 0.079 | 1 |
| SPAG9 | 0.000112317962193702 | 0.367928334101873 | 0.136 | 0.154 | 1 |
| CCNL1 | 0.000112510192794265 | 0.368280413768342 | 0.453 | 0.501 | 1 |
| UBAP1 | 0.000114858939197396 | 0.397289137419073 | 0.096 | 0.11 | 1 |
| AGFG1 | 0.000115338818034245 | 0.514427128854533 | 0.098 | 0.113 | 1 |
| TARBP1 | 0.000115802559792737 | 0.178295275904826 | 0.062 | 0.071 | 1 |
| PGPEP1 | 0.000119959779780393 | 0.188901316532239 | 0.045 | 0.053 | 1 |
| AP3M2 | 0.000126462123887269 | 0.427432296955688 | 0.047 | 0.055 | 1 |
| MTF1 | 0.000133748757519667 | 0.356504328063908 | 0.043 | 0.05 | 1 |
| POLM | 0.000134107641090568 | 0.258739156387619 | 0.043 | 0.05 | 1 |
| MCL1 | 0.000134279462505957 | 0.174776275020531 | 0.431 | 0.501 | 1 |
| WDFY2 | 0.000134536953032512 | 0.671209424771371 | 0.06 | 0.053 | 1 |
| LEPROTL1 | 0.000135508412366153 | 0.234674472867756 | 0.277 | 0.317 | 1 |
| GDAP2 | 0.000136355096137973 | 0.35369717736113 | 0.047 | 0.055 | 1 |
| TMEM39A | 0.000137543379730295 | 0.307830901063318 | 0.052 | 0.06 | 1 |
| ZNF277 | 0.000138041205560986 | 0.337148012362101 | 0.068 | 0.078 | 1 |
| ZNF236 | 0.00014281083869862 | 0.318209261884328 | 0.046 | 0.054 | 1 |
| VMP1 | 0.000144779265369535 | 0.458007621254931 | 0.176 | 0.202 | 1 |
| RUBCN | 0.000150465911939213 | 0.260510515129586 | 0.053 | 0.062 | 1 |
| MAP4K4 | 0.000150614192265378 | 0.355452234131402 | 0.182 | 0.207 | 1 |
| B4GALT1 | 0.000150787884735331 | 0.286776079290465 | 0.144 | 0.163 | 1 |
| LINC01278 | 0.000153615961193411 | 0.276398945500862 | 0.049 | 0.057 | 1 |
| RFX7 | 0.00016147991104263 | 0.326686158078274 | 0.133 | 0.151 | 1 |

| Gene | P_val | Avg_log2FC | Pct. 1 | Pct. 2 | P_val_adj |
|---|---|---|---|---|---|
| MARCHF8 | 0.000162059738452182 | 0.15829799939074 | 0.055 | 0.064 | 1 |
| CAMK1D | 0.000162889623936182 | 0.488595536417279 | 0.113 | 0.106 | 1 |
| NIN | 0.00016314722054067 | 0.219415878213505 | 0.291 | 0.332 | 1 |
| NFKBIA | 0.000165691166782401 | 0.549575928923079 | 0.334 | 0.356 | 1 |
| SRGAP2 | 0.000167469990957877 | 0.401554520167428 | 0.076 | 0.087 | 1 |
| ZNF708 | 0.000168524296436046 | 0.250099290400913 | 0.052 | 0.06 | 1 |
| ARMH3 | 0.000172914864857267 | 0.230991555718154 | 0.081 | 0.092 | 1 |
| ELMO1 | 0.000173652463259912 | 0.626348828075684 | 0.145 | 0.142 | 1 |
| PBXIP1 | 0.000175670879174018 | 0.372175499477532 | 0.206 | 0.204 | 1 |
| TEPSIN | 0.000181729304757959 | 0.232476300706599 | 0.072 | 0.082 | 1 |
| CD82 | 0.000187741127335826 | 0.360919966494954 | 0.085 | 0.097 | 1 |
| TCF12 | 0.00018997078473772 | 0.409894339142678 | 0.114 | 0.13 | 1 |
| TTC3 | 0.000191705099196759 | 0.271234238940098 | 0.16 | 0.18 | 1 |
| SOS1 | 0.000196529795673114 | 0.321065187254511 | 0.198 | 0.224 | 1 |
| MIGA1 | 0.000197782562992552 | 0.269509102120049 | 0.048 | 0.056 | 1 |
| UBE2B | 0.000205022338828424 | 0.36103763717298 | 0.168 | 0.191 | 1 |
| LTB | 0.000205272432281618 | 0.476919186562955 | 0.209 | 0.207 | 1 |
| RHOH | 0.00021156143618018 | 0.296319608943722 | 0.191 | 0.217 | 1 |
| RICTOR | 0.000211984987564485 | 0.445963577012249 | 0.203 | 0.203 | 1 |
| SYNE1 | 0.000214163953767486 | 0.137712042573942 | 0.483 | 0.548 | 1 |
| FAM193A | 0.000219759332391033 | 0.268008751629415 | 0.064 | 0.073 | 1 |
| SSH1 | 0.00022151724901607 | 0.334904726481797 | 0.061 | 0.07 | 1 |
| ANKIB1 | 0.0002230733917557 | 0.325479352214747 | 0.123 | 0.139 | 1 |
| IL21R | 0.000227969507209066 | 0.166199397455089 | 0.077 | 0.087 | 1 |
| ZBTB11 | 0.000231176064720626 | 0.312715365936707 | 0.087 | 0.098 | 1 |
| FCGRT | 0.000232614370831056 | 0.56634489650469 | 0.082 | 0.075 | 1 |
| PLIN3 | 0.000232717038559776 | 0.401687157406873 | 0.059 | 0.067 | 1 |
| CNST | 0.000232991881489168 | 0.320713172527107 | 0.102 | 0.116 | 1 |
| TOM1 | 0.000234627385471422 | 0.48773808737937 | 0.095 | 0.089 | 1 |
| FAM126B | 0.000235075300516516 | 0.360702723186155 | 0.082 | 0.093 | 1 |
| FAM13B | 0.000237419601590402 | 0.287715592944489 | 0.124 | 0.14 | 1 |
| SORL1 | 0.000238768690092233 | 0.497733697565954 | 0.141 | 0.136 | 1 |

| Gene | P_val | Avg_log2FC | Pct.1 | Pct.2 | P_val_adj |
|---|---|---|---|---|---|
| ECE1 | 0.000239513655552453 | 0.398706664070419 | 0.113 | 0.128 | 1 |
| ATR | 0.000241178344735982 | 0.3484173504235 | 0.119 | 0.135 | 1 |
| EPC2 | 0.000241422882315827 | 0.383636162041757 | 0.077 | 0.088 | 1 |
| PIP5K1A | 0.000241713686705758 | 0.30420155005431 | 0.042 | 0.05 | 1 |
| ATP10A | 0.000243457062387777 | 0.209871202579249 | 0.081 | 0.092 | 1 |
| N4BP1 | 0.000250136077785973 | 0.335231827865965 | 0.159 | 0.179 | 1 |
| ANKRD17 | 0.000251611838308055 | 0.336432169515665 | 0.148 | 0.167 | 1 |
| DYRK1A | 0.000252914365990532 | 0.34280214673395 | 0.146 | 0.165 | 1 |
| CREG1 | 0.000254307297871735 | 0.640841112282251 | 0.043 | 0.05 | 1 |
| ADAM19 | 0.000256365931124551 | 0.384521179962905 | 0.051 | 0.06 | 1 |
| NFX1 | 0.000263316375265301 | 0.269687499635395 | 0.082 | 0.093 | 1 |
| PPIL4 | 0.000268816084720931 | 0.303303259298993 | 0.059 | 0.068 | 1 |
| PUM2 | 0.000269505398652376 | 0.324266145485849 | 0.165 | 0.187 | 1 |
| BTG2 | 0.000279115671291264 | 0.462188487043665 | 0.139 | 0.133 | 1 |
| CYLD | 0.000280357645756179 | 0.253465807463886 | 0.251 | 0.285 | 1 |
| PLEKHM1 | 0.000287696986664728 | 0.165408030916178 | 0.097 | 0.109 | 1 |
| ZNF654 | 0.000289951599946073 | 0.289681470107927 | 0.057 | 0.065 | 1 |
| EP300 | 0.000292339962222017 | 0.317409415814807 | 0.174 | 0.196 | 1 |
| ATXN2 | 0.000299958785356441 | 0.31400460554223 | 0.055 | 0.063 | 1 |
| OXNAD1 | 0.000306188907514894 | 0.295969881585963 | 0.136 | 0.153 | 1 |
| ZNF148 | 0.000317973264385516 | 0.297781086893379 | 0.155 | 0.174 | 1 |
| BSDC1 | 0.000319086902105496 | 0.169563885683966 | 0.057 | 0.065 | 1 |
| CCDC84 | 0.000321276540009523 | 0.31215304727976 | 0.051 | 0.059 | 1 |
| FBXO31 | 0.000321420111380175 | 0.166071050265408 | 0.044 | 0.051 | 1 |
| STAT4 | 0.000328646096491607 | 0.244485160105398 | 0.204 | 0.23 | 1 |
| GSAP | 0.000329090196440374 | 0.280425660357593 | 0.094 | 0.106 | 1 |
| CNOT6L | 0.000335030674877634 | 0.385291316316081 | 0.281 | 0.29 | 1 |
| PDE4D | 0.000336867711274967 | 0.285408258075101 | 0.216 | 0.244 | 1 |
| MADD | 0.000337050890343858 | 0.301402620597713 | 0.118 | 0.132 | 1 |
| FAM160B2 | 0.000337820360979546 | 0.125667084360769 | 0.052 | 0.059 | 1 |
| ZZEF1 | 0.000338763730572946 | 0.240661923442601 | 0.132 | 0.147 | 1 |
| NLRC5 | 0.000357122339041076 | 0.133015677955552 | 0.312 | 0.349 | 1 |

| Gene | P_val | Avg_log2FC | Pct.1 | Pct.2 | P_val_adj |
|---|---|---|---|---|---|
| REV3L | 0.000373373068779398 | 0.331991058079429 | 0.095 | 0.107 | 1 |
| USP47 | 0.000373398748318215 | 0.364474218314327 | 0.169 | 0.191 | 1 |
| FLYWCH1 | 0.000390280456095817 | 0.166333201551372 | 0.064 | 0.073 | 1 |
| UBR5 | 0.000406358399768555 | 0.334570642556781 | 0.153 | 0.172 | 1 |
| LPAR2 | 0.000420098038050584 | 0.166426180039688 | 0.044 | 0.051 | 1 |
| KIF1B | 0.000420310608557731 | 0.567492793856987 | 0.069 | 0.063 | 1 |
| FBXO38 | 0.000422287017382306 | 0.379529013174694 | 0.055 | 0.063 | 1 |
| IL17RA | 0.000430203772643354 | 0.620244152256276 | 0.108 | 0.102 | 1 |
| IL18R1 | 0.000431705538550502 | 0.371272062334149 | 0.053 | 0.061 | 1 |
| TCP11L2 | 0.000446281745525662 | 0.477229573843475 | 0.055 | 0.049 | 1 |
| HERC4 | 0.000452502376957338 | 0.327622107045022 | 0.083 | 0.094 | 1 |
| ZNF394 | 0.000458045996853012 | 0.216543728078898 | 0.201 | 0.225 | 1 |
| ARHGAP25 | 0.000459971034870703 | 0.21853431177944 | 0.275 | 0.311 | 1 |
| AL772307.1 | 0.000467182213112996 | 0.223131442037723 | 0.075 | 0.084 | 1 |
| MTMR3 | 0.000476500732422598 | 0.665477417264065 | 0.058 | 0.052 | 1 |
| SNTB1 | 0.000498785077806748 | 0.329334171345378 | 0.065 | 0.074 | 1 |
| GRN | 0.000508192992313161 | 0.70507851585822 | 0.162 | 0.161 | 1 |
| AHCTF1 | 0.000512341586350586 | 0.406520282525011 | 0.09 | 0.101 | 1 |
| FOSL2 | 0.000530462078476336 | 0.17522902551319 | 0.138 | 0.153 | 1 |
| FBXW4 | 0.000558942059313789 | 0.249269634632109 | 0.067 | 0.076 | 1 |
| CBL | 0.000584166787259783 | 0.197010890660655 | 0.077 | 0.087 | 1 |
| TECPR1 | 0.00058799223209549 | 0.197656777982788 | 0.112 | 0.124 | 1 |
| AC006978.2 | 0.000592603656744915 | 0.174991533093126 | 0.082 | 0.092 | 1 |
| TTLL3 | 0.000595581137628786 | 0.120088384120873 | 0.049 | 0.056 | 1 |
| RCOR1 | 0.000630027421768954 | 0.676531915062972 | 0.073 | 0.067 | 1 |
| CHD1 | 0.000645804699490821 | 0.435809480231484 | 0.256 | 0.263 | 1 |
| TADA2B | 0.000650245893698665 | 0.256625518274267 | 0.057 | 0.065 | 1 |
| RLF | 0.00066415320624695 | 0.226937346287303 | 0.108 | 0.121 | 1 |
| SFI1 | 0.000668636942986915 | 0.330876119439555 | 0.127 | 0.142 | 1 |
| XPO6 | 0.000681434738989245 | 0.371595125238925 | 0.178 | 0.201 | 1 |
| CDC14A | 0.000687877301725504 | 0.576283400563686 | 0.079 | 0.073 | 1 |
| KIAA1109 | 0.000697135649880051 | 0.463193122144655 | 0.181 | 0.179 | 1 |

| Gene | P_val | Avg_log2FC | Pct.1 | Pct.2 | P_val_adj |
|---|---|---|---|---|---|
| SRRM2 | 0.000707720413748012 | 0.114131794341921 | 0.461 | 0.528 | 1 |
| ZCCHC7 | 0.00071702929777443 | 0.327244319889878 | 0.115 | 0.129 | 1 |
| AL096870.2 | 0.00072024794088254 | 0.252418565181062 | 0.058 | 0.066 | 1 |
| DOCK10 | 0.000750079262443605 | 0.299553907511518 | 0.265 | 0.303 | 1 |
| DIP2B | 0.000788187013633317 | 0.621146576304313 | 0.064 | 0.058 | 1 |
| CBLB | 0.000799617657531815 | 0.320792640750525 | 0.282 | 0.321 | 1 |
| AF165147.1 | 0.000815331385956327 | 0.525455030721516 | 0.066 | 0.061 | 1 |
| NKTR | 0.000824363734099099 | 0.418104097253758 | 0.267 | 0.275 | 1 |
| HELZ | 0.000846925233394629 | 0.33926957130044 | 0.209 | 0.237 | 1 |
| DOCK2 | 0.000863848833736097 | 0.259104394115815 | 0.334 | 0.385 | 1 |
| CLEC16A | 0.000866639871345111 | 0.261706327600163 | 0.047 | 0.054 | 1 |
| ASAP1 | 0.000884807870540942 | 0.665198482207213 | 0.142 | 0.14 | 1 |
| BIRC3 | 0.000896334865541102 | 0.32129109107383 | 0.07 | 0.079 | 1 |
| LINC-PINT | 0.000902768343354462 | 0.301945623222898 | 0.067 | 0.075 | 1 |
| MRTFA | 0.000936799823603995 | 0.299252263035817 | 0.084 | 0.094 | 1 |
| FBXO32 | 0.000953770692516254 | 0.143670013386798 | 0.085 | 0.095 | 1 |
| SLC16A3 | 0.000956896411033767 | 0.49606249723006 | 0.05 | 0.057 | 1 |
| UNC119 | 0.000973336969397483 | 0.384816712097999 | 0.055 | 0.062 | 1 |
| TENT4B | 0.00101704033549571 | 0.244993978071499 | 0.109 | 0.121 | 1 |
| MACF1 | 0.00101749527141976 | 0.12770711072118 | 0.477 | 0.542 | 1 |
| JAZF1 | 0.0010522854654811 | 0.292009293239622 | 0.111 | 0.124 | 1 |
| NPC1 | 0.00106897973461675 | 0.229664023108323 | 0.138 | 0.153 | 1 |
| PRKCQ | 0.00108966270822454 | 0.379034865617823 | 0.193 | 0.217 | 1 |
| PCMTD1 | 0.00110903310048001 | 0.247594201758239 | 0.108 | 0.12 | 1 |
| BBX | 0.00111099424968231 | 0.283553209271448 | 0.182 | 0.202 | 1 |
| EIF2AK3 | 0.00114033268034652 | 0.442277712948565 | 0.052 | 0.059 | 1 |
| MED23 | 0.00114179755835597 | 0.171094678826102 | 0.072 | 0.08 | 1 |
| VPS8 | 0.00115370088470657 | 0.338218459170588 | 0.091 | 0.102 | 1 |
| KDM3B | 0.00119336345682495 | 0.29815291159579 | 0.118 | 0.131 | 1 |
| BTN3A1 | 0.001197175762972 | 0.168643437198567 | 0.271 | 0.301 | 1 |
| POLR3E | 0.00120883043146037 | 0.332136442571867 | 0.056 | 0.064 | 1 |
| CAPG | 0.00121713523290055 | 0.504090057317012 | 0.05 | 0.057 | 1 |

| Gene | P_val | Avg_log2FC | Pct.1 | Pct.2 | P_val_adj |
|---|---|---|---|---|---|
| VEZT | 0.0012220313414883 | 0.35065242327233 | 0.056 | 0.064 | 1 |
| GALNT11 | 0.00122756398744654 | 0.252344249269401 | 0.081 | 0.091 | 1 |
| RABGAP1L | 0.00123408872064682 | 0.550024201283294 | 0.256 | 0.265 | 1 |
| KDM1B | 0.00124171891057072 | 0.459866987562245 | 0.045 | 0.052 | 1 |
| ARHGAP35 | 0.00124661147015972 | 0.23083176210732 | 0.093 | 0.103 | 1 |
| ABTB1 | 0.00132226636719344 | 0.25989387678963 | 0.13 | 0.144 | 1 |
| TRAF5 | 0.00133369184355379 | 0.255835530135136 | 0.095 | 0.106 | 1 |
| ASH1L | 0.00134131666204238 | 0.422415230380629 | 0.185 | 0.185 | 1 |
| CD27 | 0.00135361248016824 | 0.182715885114854 | 0.201 | 0.222 | 1 |
| GPCPD1 | 0.00137409477345883 | 0.462771276562976 | 0.207 | 0.208 | 1 |
| MACO1 | 0.00139827907687222 | 0.259065633004468 | 0.065 | 0.073 | 1 |
| TNK2 | 0.00143900217879607 | 0.126544310790468 | 0.062 | 0.069 | 1 |
| FOXO3 | 0.00147158957533297 | 0.582420433812065 | 0.083 | 0.077 | 1 |
| GSDMB | 0.00152024666738973 | 0.112649978615797 | 0.044 | 0.05 | 1 |
| ANKMY1 | 0.00153102049930525 | 0.280731328117549 | 0.044 | 0.05 | 1 |
| THADA | 0.00153240573526141 | 0.392957039018396 | 0.067 | 0.075 | 1 |
| ZMYND8 | 0.00156274991687493 | 0.284823659332752 | 0.097 | 0.108 | 1 |
| TTBK2 | 0.00157024552887357 | 0.194339452867815 | 0.044 | 0.05 | 1 |
| MYO9B | 0.00159217769372179 | 0.190936454639897 | 0.288 | 0.321 | 1 |
| TXNDC11 | 0.00159278266134607 | 0.397783722690136 | 0.095 | 0.106 | 1 |
| ZNFX1 | 0.00165015542635798 | 0.238235856340753 | 0.162 | 0.179 | 1 |
| FBXW7 | 0.00165178459899989 | 0.325968760605314 | 0.183 | 0.205 | 1 |
| TRIM38 | 0.00166930130838802 | 0.362700091559987 | 0.146 | 0.163 | 1 |
| RREB1 | 0.0016793267080737 | 0.523076647047196 | 0.06 | 0.055 | 1 |
| EHBP1 | 0.0017212037309868 | 0.314951864042296 | 0.045 | 0.051 | 1 |
| AC008105.3 | 0.00175424539722452 | 0.460954714341282 | 0.066 | 0.06 | 1 |
| ABCA7 | 0.00188976516523079 | 0.17666714810606 | 0.085 | 0.094 | 1 |
| MIR181A1HG | 0.00189415036892313 | 0.484735367023815 | 0.044 | 0.05 | 1 |
| RUFY2 | 0.00189514522129992 | 0.401139742727926 | 0.05 | 0.056 | 1 |
| NCOA3 | 0.00191090885479444 | 0.300089723555085 | 0.145 | 0.161 | 1 |
| CHIC2 | 0.00191429989458443 | 0.337394397640648 | 0.082 | 0.092 | 1 |
| PICALM | 0.00191798337303956 | 0.396364414560942 | 0.253 | 0.291 | 1 |

| Gene | P_val | Avg_log2FC | Pct.1 | Pct.2 | P_val_adj |
|---|---|---|---|---|---|
| TTC17 | 0.00193477732839777 | 0.311134820305939 | 0.151 | 0.168 | 1 |
| CAMKMT | 0.00201514796326782 | 0.588755331020362 | 0.058 | 0.053 | 1 |
| PHC3 | 0.00204187244243491 | 0.304027427437426 | 0.13 | 0.145 | 1 |
| CEBPB | 0.00204667689595174 | 0.562143540667951 | 0.261 | 0.271 | 1 |
| MYLIP | 0.00208431271381032 | 0.224402243442259 | 0.096 | 0.106 | 1 |
| SLC12A6 | 0.002097422091469 | 0.719361904316463 | 0.069 | 0.064 | 1 |
| PCGF3 | 0.00211501930931922 | 0.18029592732919 | 0.07 | 0.078 | 1 |
| SNX29 | 0.00220430685561594 | 0.476658921248179 | 0.058 | 0.053 | 1 |
| PITPNC1 | 0.00225726781888797 | 0.472490298279514 | 0.174 | 0.173 | 1 |
| SYNE2 | 0.00231664525604631 | 0.137875586519842 | 0.566 | 0.647 | 1 |
| AGO2 | 0.00240178853543264 | 0.408390764429787 | 0.063 | 0.071 | 1 |
| CASD1 | 0.00242047674131769 | 0.312828595131873 | 0.052 | 0.059 | 1 |
| SOS2 | 0.00244249030790395 | 0.49202509455953 | 0.095 | 0.09 | 1 |
| IL10RA | 0.00245056395020778 | 0.166192976250181 | 0.367 | 0.417 | 1 |
| CAMK4 | 0.00250216782891663 | 0.501383439908134 | 0.148 | 0.166 | 1 |
| CHST11 | 0.00266700322802724 | 0.368901547734947 | 0.234 | 0.265 | 1 |
| R3HDM2 | 0.00266783170400733 | 0.268864777754978 | 0.152 | 0.169 | 1 |
| GATAD2B | 0.00278920794975359 | 0.360204925325639 | 0.114 | 0.127 | 1 |
| REL | 0.00286716416267086 | 0.428381175155883 | 0.164 | 0.162 | 1 |
| ZBTB40 | 0.00289977765196674 | 0.188975302680232 | 0.062 | 0.07 | 1 |
| EZH1 | 0.00291976790591391 | 0.198040290918144 | 0.109 | 0.121 | 1 |
| ATP8A1 | 0.00298332872808349 | 0.435643287971778 | 0.105 | 0.101 | 1 |
| MGA | 0.0030404398354282 | 0.253738991408494 | 0.058 | 0.065 | 1 |
| ATP11B | 0.00304957477249308 | 0.365915338461787 | 0.199 | 0.223 | 1 |
| ZEB2 | 0.00306872252422624 | 0.411881612820012 | 0.322 | 0.371 | 1 |
| ANKFY1 | 0.0032424535761064 | 0.344377854076024 | 0.061 | 0.068 | 1 |
| TRAF3 | 0.00324363475478473 | 0.470570462991815 | 0.1 | 0.113 | 1 |
| NBAS | 0.0032652978454971 | 0.310900549161977 | 0.066 | 0.074 | 1 |
| BCL2L11 | 0.00331929687598516 | 0.233458404562945 | 0.144 | 0.159 | 1 |
| LACTB | 0.00335786344233926 | 0.455277053579295 | 0.046 | 0.052 | 1 |
| ARID1B | 0.00341936694146026 | 0.552640281694329 | 0.144 | 0.142 | 1 |
| SEL1L3 | 0.00357665188774562 | 0.258467238395786 | 0.075 | 0.084 | 1 |

| Gene | P_val | Avg_log2FC | Pct.1 | Pct.2 | P_val_adj |
|---|---|---|---|---|---|
| NIBAN1 | 0.00359829665770181 | 0.395350410949879 | 0.211 | 0.237 | 1 |
| NLRP1 | 0.00364798967753781 | 0.307483100662515 | 0.3 | 0.311 | 1 |
| SMAP2 | 0.0037998773917517 | 0.375890523195513 | 0.366 | 0.397 | 1 |
| SCARNA9 | 0.00380000233357758 | 0.186791260616904 | 0.053 | 0.06 | 1 |
| GOLGA8B | 0.00381216384377721 | 0.238709836677368 | 0.198 | 0.219 | 1 |
| GRAMD1B | 0.00382406747926363 | 0.395440929720698 | 0.06 | 0.067 | 1 |
| MYCBP2 | 0.00385529977534901 | 0.421782457400064 | 0.236 | 0.241 | 1 |
| IFRD1 | 0.00386931274682843 | 0.490302482566063 | 0.067 | 0.075 | 1 |
| WDR70 | 0.00390151553972007 | 0.435104875633514 | 0.1 | 0.111 | 1 |
| ZNF33B | 0.00392670033913381 | 0.24859056820427 | 0.046 | 0.052 | 1 |
| CSGALNACT2 | 0.00402367403321779 | 0.395766805381972 | 0.173 | 0.193 | 1 |
| HEATR5B | 0.0040723399832787 | 0.215436871591128 | 0.071 | 0.079 | 1 |
| RAB11FIP4 | 0.0040901026413906 | 0.270369374849428 | 0.083 | 0.092 | 1 |
| BCAS3 | 0.0040932691924128 | 0.647542434401371 | 0.125 | 0.122 | 1 |
| HIVEP2 | 0.00415781713084965 | 0.511761550329934 | 0.077 | 0.073 | 1 |
| GOLGA8A | 0.00417140415633482 | 0.226032565131092 | 0.17 | 0.187 | 1 |
| MFSD14C | 0.00430360697268922 | 0.321596577650435 | 0.082 | 0.091 | 1 |
| SAMD4B | 0.00448766047786016 | 0.302328387641405 | 0.065 | 0.072 | 1 |
| EPSTI1 | 0.00452928243514638 | 0.348596164332399 | 0.252 | 0.283 | 1 |
| TENT5C | 0.00458085352748481 | 0.459222693080816 | 0.123 | 0.119 | 1 |
| MEF2A | 0.00461804511029413 | 0.625965808471046 | 0.121 | 0.118 | 1 |
| PIK3IP1 | 0.00462103704410347 | 0.304410295179139 | 0.202 | 0.201 | 1 |
| SLC20A2 | 0.00488252020236973 | 0.288032262653471 | 0.049 | 0.055 | 1 |
| TET3 | 0.00504470027370566 | 0.472896530024373 | 0.05 | 0.045 | 1 |
| FGD3 | 0.00516490727198997 | 0.232771603075162 | 0.213 | 0.234 | 1 |
| FBXL20 | 0.00517148439607223 | 0.451803066465546 | 0.07 | 0.066 | 1 |
| UBAC2 | 0.00528179269486902 | 0.391413587455096 | 0.129 | 0.144 | 1 |
| PGGHG | 0.00529436532299442 | 0.474075114499592 | 0.082 | 0.077 | 1 |
| TEP1 | 0.00564577206768025 | 0.270758176683072 | 0.049 | 0.055 | 1 |
| SDCCAG8 | 0.00599504261575053 | 0.363164463458266 | 0.056 | 0.063 | 1 |
| GOLGA4 | 0.00601800757007873 | 0.324911661696486 | 0.222 | 0.248 | 1 |
| NCALD | 0.00602307547981234 | 0.443125513753105 | 0.108 | 0.12 | 1 |

| Gene | P_val | Avg_log2FC | Pct. 1 | Pct. 2 | P_val_adj |
|---|---|---|---|---|---|
| MINDY2 | 0.00610535516453589 | 0.236518962811577 | 0.063 | 0.069 | 1 |
| CEP85L | 0.00612721033121522 | 0.287138311612811 | 0.169 | 0.186 | 1 |
| SIDT1 | 0.0063037095821093 | 0.346943126465758 | 0.092 | 0.102 | 1 |
| CCDC91 | 0.00645682167470302 | 0.346897178437081 | 0.14 | 0.154 | 1 |
| HBP1 | 0.00648820150471823 | 0.304653933336292 | 0.148 | 0.164 | 1 |
| CUL3 | 0.0066951648884117 | 0.409094996347645 | 0.188 | 0.212 | 1 |
| ARAP2 | 0.00685009392103262 | 0.228584072389657 | 0.327 | 0.368 | 1 |
| PSME4 | 0.00686877723490913 | 0.374396668115068 | 0.127 | 0.14 | 1 |
| BCL11B | 0.00693941933691275 | 0.302110112370035 | 0.234 | 0.262 | 1 |
| COG5 | 0.00717054095749473 | 0.560551007883142 | 0.087 | 0.083 | 1 |
| LYN | 0.00724636448001827 | 0.667451808340171 | 0.181 | 0.186 | 1 |
| IFIT2 | 0.00726307289097691 | 0.492539590948644 | 0.072 | 0.079 | 1 |
| ARHGEF7 | 0.00736833275611905 | 0.333139570542068 | 0.073 | 0.081 | 1 |
| MBOAT7 | 0.00745751211990826 | 0.552146685177725 | 0.055 | 0.062 | 1 |
| TFCP2 | 0.00748231803717779 | 0.367189681474053 | 0.048 | 0.054 | 1 |
| NCK2 | 0.00777186571945374 | 0.340118387800552 | 0.094 | 0.104 | 1 |
| UVRAG | 0.00778796029425743 | 0.303330873101606 | 0.109 | 0.121 | 1 |
| HELB | 0.00786771596987132 | 0.257302745230978 | 0.094 | 0.103 | 1 |
| PNRC1 | 0.0079110545301695 | 0.172121145126472 | 0.555 | 0.61 | 1 |
| RASA1 | 0.00820098897356107 | 0.315957142885042 | 0.058 | 0.064 | 1 |
| ADAM17 | 0.00825701611066418 | 0.428356283660606 | 0.09 | 0.1 | 1 |
| KAT6A | 0.00825759325412937 | 0.303222902893451 | 0.197 | 0.218 | 1 |
| KLHL24 | 0.00855371427025546 | 0.433114859557575 | 0.122 | 0.119 | 1 |
| TBC1D5 | 0.00896529118900492 | 0.575018565105475 | 0.138 | 0.137 | 1 |
| PPP1R16B | 0.00923151823358126 | 0.594421500246417 | 0.143 | 0.142 | 1 |
| SNX13 | 0.00929443973019009 | 0.36153235906706 | 0.07 | 0.077 | 1 |
| OSBPL9 | 0.00990044835351137 | 0.485057949870312 | 0.08 | 0.088 | 1 |
| ACYP2 | 0.0100803805561033 | 0.33097793255443 | 0.069 | 0.076 | 1 |
| BCL3 | 0.0101238933225073 | 0.419525139335384 | 0.109 | 0.119 | 1 |
| EXOC4 | 0.0102918444789534 | 0.529648141533865 | 0.132 | 0.131 | 1 |
| TMEM154 | 0.0106150800253781 | 0.567303699927617 | 0.068 | 0.076 | 1 |
| TRERF1 | 0.0106291093842987 | 0.223445481661333 | 0.112 | 0.123 | 1 |

| Gene | P_val | Avg_log2FC | Pct. 1 | Pct. 2 | P_val_adj |
|---|---|---|---|---|---|
| VPS9D1 | 0.011305023216397 | 0.178338630365313 | 0.055 | 0.06 | 1 |
| ATP13A3 | 0.0113054300375332 | 0.519335530690769 | 0.08 | 0.088 | 1 |
| TTC21B | 0.0114269515174229 | 0.297059332169883 | 0.047 | 0.052 | 1 |
| XPR1 | 0.011558618327441 | 0.327770461729883 | 0.049 | 0.055 | 1 |
| NBPF12 | 0.0117521645223727 | 0.124904508031279 | 0.064 | 0.07 | 1 |
| ARFGEF1 | 0.0118517918596361 | 0.335061800932507 | 0.157 | 0.173 | 1 |
| STK17B | 0.0118802972930843 | 0.309895890751413 | 0.434 | 0.481 | 1 |
| TNIK | 0.0120975935508553 | 0.327099373409821 | 0.161 | 0.178 | 1 |
| IFITM3 | 0.0123339665015186 | 0.678697110272148 | 0.445 | 0.494 | 1 |
| IFIT3 | 0.0125014502826202 | 0.541839410892321 | 0.067 | 0.074 | 1 |
| SLC12A7 | 0.0125533210372029 | 0.168918078589389 | 0.053 | 0.058 | 1 |
| TRAF1 | 0.0134817223504041 | 0.310099224809996 | 0.092 | 0.1 | 1 |
| ITGAX | 0.0136233057910832 | 0.476713242758343 | 0.085 | 0.093 | 1 |
| RNF111 | 0.0138048842939712 | 0.368443765954923 | 0.065 | 0.072 | 1 |
| YPEL5 | 0.0140104941901176 | 0.298775700082533 | 0.227 | 0.251 | 1 |
| SATB1 | 0.0141099077555573 | 0.386941307912098 | 0.095 | 0.092 | 1 |
| TNFRSF25 | 0.0142475681673323 | 0.398560808864231 | 0.072 | 0.079 | 1 |
| HDAC4 | 0.0142961326164254 | 0.457620517340698 | 0.057 | 0.054 | 1 |
| ANKRD36 | 0.014401475729464 | 0.423310871842654 | 0.119 | 0.131 | 1 |
| VPS50 | 0.0153181669589178 | 0.400404373607552 | 0.045 | 0.05 | 1 |
| BCL2 | 0.0154978074790572 | 0.503591566939738 | 0.076 | 0.084 | 1 |
| KLF6 | 0.015780452600097 | 0.218991322638251 | 0.396 | 0.45 | 1 |
| SECISBP2L | 0.0160027093499224 | 0.281855657823068 | 0.108 | 0.118 | 1 |
| SPIDR | 0.0160960178615169 | 0.589434443312903 | 0.132 | 0.132 | 1 |
| ZER1 | 0.0163799246216967 | 0.235276608271326 | 0.062 | 0.068 | 1 |
| PIK3R5 | 0.0173773926165177 | 0.274308610671726 | 0.274 | 0.304 | 1 |
| MAPK14 | 0.0176426169067399 | 0.567626361225627 | 0.139 | 0.155 | 1 |
| SCAPER | 0.0176858769538601 | 0.588681544762788 | 0.07 | 0.066 | 1 |
| FOXO1 | 0.0178871046415169 | 0.469449736478103 | 0.088 | 0.085 | 1 |
| ZXDC | 0.0182145988536255 | 0.309065554533454 | 0.084 | 0.092 | 1 |
| BTAF1 | 0.018299116126496 | 0.26385751469211 | 0.152 | 0.166 | 1 |
| AC092683.1 | 0.0183356231313226 | 0.636145637246971 | 0.092 | 0.089 | 1 |

| Gene | P_val | Avg_log2FC | Pct. 1 | Pct. 2 | P_val_adj |
|---|---|---|---|---|---|
| SRGAP2B | 0.0184449330478067 | 0.458753077733428 | 0.05 | 0.055 | 1 |
| CTSZ | 0.0187081473659266 | 0.51445004402156 | 0.082 | 0.09 | 1 |
| AKAP13 | 0.0188863250983557 | 0.177547264968308 | 0.428 | 0.487 | 1 |
| MAP3K5 | 0.0188868307154483 | 0.517946429665492 | 0.143 | 0.143 | 1 |
| ORAI2 | 0.0190292130395211 | 0.310898405781957 | 0.09 | 0.098 | 1 |
| APLP2 | 0.0193971713977533 | 0.672437962013963 | 0.135 | 0.135 | 1 |
| GUSBP11 | 0.0197432879585259 | 0.200902507871899 | 0.056 | 0.061 | 1 |
| BOD1L1 | 0.0205310016089204 | 0.277161992512668 | 0.215 | 0.236 | 1 |
| PARP14 | 0.0207066748181347 | 0.337317720678313 | 0.218 | 0.243 | 1 |
| KAT6B | 0.0209677042757394 | 0.32423129645173 | 0.114 | 0.124 | 1 |
| ZDHHC17 | 0.0212546261681003 | 0.253606074624997 | 0.079 | 0.086 | 1 |
| SPATA20 | 0.0212712929030247 | 0.279047998623681 | 0.046 | 0.051 | 1 |
| MCTP2 | 0.0213326084653679 | 0.497710183535813 | 0.128 | 0.141 | 1 |
| RAD51-AS1 | 0.0217246416970038 | 0.1679154871635 | 0.058 | 0.063 | 1 |
| PHACTR2 | 0.0222951555533996 | 0.494084233711226 | 0.193 | 0.198 | 1 |
| ZFC3H1 | 0.022317496433977 | 0.264605085630205 | 0.236 | 0.261 | 1 |
| MXI1 | 0.0227650107794487 | 0.392449270165962 | 0.079 | 0.075 | 1 |
| LRRC37B | 0.0238135003967606 | 0.261172574736523 | 0.06 | 0.065 | 1 |
| TMEM260 | 0.0238490693742666 | 0.36465203284151 | 0.045 | 0.05 | 1 |
| PLA2G6 | 0.024286014729384 | 0.171922773368487 | 0.052 | 0.057 | 1 |
| NCF4 | 0.0244502514759243 | 0.658733956999433 | 0.059 | 0.065 | 1 |
| USP42 | 0.0247976648921539 | 0.259789698395383 | 0.076 | 0.083 | 1 |
| ARFGEF2 | 0.0250943130043986 | 0.348725593991187 | 0.081 | 0.089 | 1 |
| CERS6 | 0.0252191558663804 | 0.485425117729903 | 0.063 | 0.069 | 1 |
| PIGB | 0.0253516444934467 | 0.438704812095845 | 0.05 | 0.056 | 1 |
| CELF1 | 0.0258634269390685 | 0.342911791809878 | 0.123 | 0.134 | 1 |
| POMT1 | 0.0260299886365319 | 0.299723937186571 | 0.054 | 0.059 | 1 |
| GZMK | 0.0260663590135116 | 0.279543380248052 | 0.219 | 0.237 | 1 |
| SLA | 0.0263932148314092 | 0.333659471830518 | 0.289 | 0.303 | 1 |
| LRBA | 0.0267912024393766 | 0.537021022167243 | 0.171 | 0.175 | 1 |
| STAT1 | 0.0268632400303633 | 0.145509441740526 | 0.456 | 0.517 | 1 |
| RHOQ | 0.0273987604870456 | 0.362864960333458 | 0.05 | 0.054 | 1 |

| Gene | P_val | Avg_log2FC | Pct.1 | Pct.2 | P_val_adj |
|---|---|---|---|---|---|
| PABPC1 | 0.028730647676306 | 0.104286926419402 | 0.742 | 0.81 | 1 |
| NR3C1 | 0.0309160109743278 | 0.279103993258188 | 0.196 | 0.215 | 1 |
| GRINA | 0.0310420877285535 | 0.568922877276375 | 0.096 | 0.093 | 1 |
| STAT2 | 0.0312912000703382 | 0.323318997002738 | 0.144 | 0.157 | 1 |
| TMEM135 | 0.031408089196609 | 0.355932641515837 | 0.046 | 0.05 | 1 |
| FNBP1 | 0.0320589735128616 | 0.210352056093844 | 0.437 | 0.502 | 1 |
| SNHG22 | 0.0323467558486672 | 0.157757058737075 | 0.076 | 0.082 | 1 |
| KLRB1 | 0.0323905341232737 | 0.157882574159999 | 0.078 | 0.084 | 1 |
| MIR22HG | 0.0331015545776931 | 0.28615629431813 | 0.054 | 0.058 | 1 |
| LINC00342 | 0.0331642536938962 | 0.492309741512834 | 0.086 | 0.094 | 1 |
| SLC35D2 | 0.0332444823708658 | 0.159792944492261 | 0.053 | 0.057 | 1 |
| HERC1 | 0.0335326531246106 | 0.355426262456186 | 0.224 | 0.23 | 1 |
| DCP1A | 0.033678271386652 | 0.319229183476547 | 0.096 | 0.105 | 1 |
| MFSD8 | 0.0338267869823099 | 0.355270605289529 | 0.047 | 0.051 | 1 |
| OGA | 0.0338639763051087 | 0.248213405732239 | 0.32 | 0.359 | 1 |
| ATXN7L1 | 0.0360945456836183 | 0.30245856561122 | 0.086 | 0.094 | 1 |
| PPP2R5C | 0.0376008889026718 | 0.144948282738674 | 0.516 | 0.594 | 1 |
| ZNF292 | 0.037843258990331 | 0.445590678231537 | 0.166 | 0.168 | 1 |
| APPL2 | 0.0378584774601903 | 0.382677071127684 | 0.07 | 0.076 | 1 |
| BTBD7 | 0.038084381414803 | 0.336819044258748 | 0.065 | 0.071 | 1 |
| MX1 | 0.0383864038225466 | 0.400364051018638 | 0.174 | 0.19 | 1 |
| FAR1 | 0.0388447734002719 | 0.384890809034372 | 0.113 | 0.124 | 1 |
| THEMIS2 | 0.0392742811995834 | 0.391647647561144 | 0.126 | 0.136 | 1 |
| ATM | 0.0402751157841614 | 0.298733151418652 | 0.314 | 0.332 | 1 |
| GOLGB1 | 0.041363687620091 | 0.30193064906943 | 0.226 | 0.247 | 1 |
| CREBRF | 0.0424919597201671 | 0.260086189150757 | 0.183 | 0.198 | 1 |
| RBL2 | 0.0442601258509659 | 0.197849236202681 | 0.351 | 0.391 | 1 |
| BACH1 | 0.0450033985367829 | 0.677903584603557 | 0.119 | 0.119 | 1 |
| CERT1 | 0.0451128717077885 | 0.454732559673862 | 0.103 | 0.113 | 1 |
| SGK3 | 0.0455479173071395 | 0.341514365103844 | 0.058 | 0.063 | 1 |
| SLC6A6 | 0.046527049197652 | 0.431562254266195 | 0.062 | 0.067 | 1 |
| TRAPPC9 | 0.0468541499364433 | 0.408563097842048 | 0.088 | 0.085 | 1 |

| Gene | P_val | Avg_log2FC | Pct. 1 | Pct. 2 | P_val_adj |
|---|---|---|---|---|---|
| APBA2 | 0.0468570015022709 | 0.2135442898995 | 0.047 | 0.052 | 1 |
| FAM210B | 0.04687209658613 | 0.192648217067519 | 0.051 | 0.056 | 1 |
| CDK17 | 0.0470367081870044 | 0.317946651624259 | 0.156 | 0.17 | 1 |
| KLHL20 | 0.0491646927932994 | 0.334217670180714 | 0.064 | 0.069 | 1 |
| ZC3H12A | 0.0494326558634748 | 0.266022944964676 | 0.063 | 0.068 | 1 |
| RELB | 0.049976211177484 | 0.479301523394066 | 0.069 | 0.066 | 1 |
| CCSER2 | 0.051442856087031 | 0.284332031850975 | 0.233 | 0.257 | 1 |
| CREBBP | 0.0523249607862293 | 0.252903763021756 | 0.18 | 0.196 | 1 |
| FOXP1 | 0.0524952173129627 | 0.230787965003015 | 0.115 | 0.123 | 1 |
| MON2 | 0.0545380915347759 | 0.494240586443761 | 0.111 | 0.11 | 1 |
| LMBRD1 | 0.0553310460724026 | 0.386496289213088 | 0.098 | 0.106 | 1 |
| TAOK1 | 0.0554205183452591 | 0.320469498286613 | 0.108 | 0.116 | 1 |
| DOP1B | 0.0570952311823101 | 0.274023719265241 | 0.047 | 0.051 | 1 |
| INTS6L | 0.0571138530185121 | 0.28785211995991 | 0.048 | 0.052 | 1 |
| BIRC6 | 0.0571612024074546 | 0.336508944812518 | 0.229 | 0.254 | 1 |
| ATG14 | 0.0576554264911131 | 0.354761518310253 | 0.065 | 0.071 | 1 |
| PCNX1 | 0.0577751195800232 | 0.495340821625428 | 0.158 | 0.16 | 1 |
| ELF1 | 0.0586095187561968 | 0.217017987609576 | 0.402 | 0.459 | 1 |
| SLAMF1 | 0.0592752980536371 | 0.370499597612575 | 0.07 | 0.075 | 1 |
| ATP11A | 0.0611103984349918 | 0.593303129701073 | 0.059 | 0.056 | 1 |
| UBR1 | 0.0632087083066535 | 0.376272117196813 | 0.109 | 0.118 | 1 |
| RPS6KA5 | 0.0642395481973687 | 0.619005284419132 | 0.059 | 0.056 | 1 |
| PACS1 | 0.0655054562858966 | 0.344973293058967 | 0.209 | 0.229 | 1 |
| LTB4R | 0.066335885129463 | 0.386403238714391 | 0.046 | 0.05 | 1 |
| TNRC6B | 0.0672239024055079 | 0.397956451680688 | 0.235 | 0.245 | 1 |
| ZNF609 | 0.0675763990488063 | 0.186071981294015 | 0.067 | 0.072 | 1 |
| DNAJB1 | 0.0677088281331812 | 0.222984070663476 | 0.313 | 0.349 | 1 |
| TMEM131L | 0.0677363556736542 | 0.407571804566953 | 0.125 | 0.135 | 1 |
| SKIL | 0.0695371682115771 | 0.582173502902006 | 0.051 | 0.048 | 1 |
| TNRC6A | 0.0696662440041027 | 0.376916139528137 | 0.127 | 0.137 | 1 |
| KIAA0232 | 0.0698183074085826 | 0.326834936941653 | 0.084 | 0.09 | 1 |
| TBC1D22A | 0.0701462891928326 | 0.321243114800788 | 0.118 | 0.127 | 1 |

| Gene | P_val | Avg_log2FC | Pct. 1 | Pct. 2 | P_val_adj |
|---|---|---|---|---|---|
| NCOA1 | 0.0710115174881951 | 0.269302340343841 | 0.23 | 0.252 | 1 |
| CHD6 | 0.0723174840490094 | 0.535530326240403 | 0.074 | 0.072 | 1 |
| RFX3 | 0.0729835234965914 | 0.463193138073734 | 0.056 | 0.054 | 1 |
| CDR2 | 0.0735517887239455 | 0.450159458473109 | 0.112 | 0.121 | 1 |
| P2RY8 | 0.0745344642605857 | 0.390085402637607 | 0.322 | 0.343 | 1 |
| CSRNP1 | 0.0756505821031144 | 0.594620683551771 | 0.058 | 0.056 | 1 |
| USP15 | 0.0772908056330564 | 0.359674585250343 | 0.34 | 0.371 | 1 |
| EPG5 | 0.0798300697562134 | 0.215157895649659 | 0.049 | 0.053 | 1 |
| IER2 | 0.0825960746053514 | 0.323507361765186 | 0.437 | 0.485 | 1 |
| CEBPD | 0.0830188493947175 | 0.51920051300514 | 0.117 | 0.127 | 1 |
| PLEKHO1 | 0.0841321467921842 | 0.284771962023572 | 0.119 | 0.128 | 1 |
| RYBP | 0.0846000197485738 | 0.595033669761925 | 0.07 | 0.068 | 1 |
| ARNTL | 0.087928143966861 | 0.295534169184276 | 0.104 | 0.112 | 1 |
| CASK | 0.088353242170818 | 0.659481403882072 | 0.056 | 0.054 | 1 |
| UBE2H | 0.088897998281528 | 0.461109782835805 | 0.129 | 0.129 | 1 |
| LENG8 | 0.0901143067408546 | 0.297975322993567 | 0.125 | 0.134 | 1 |
| SLC9A9 | 0.0901240880129037 | 0.449366751784411 | 0.092 | 0.1 | 1 |
| TRAK1 | 0.0905043120957457 | 0.505708334918742 | 0.067 | 0.065 | 1 |
| TSPYL2 | 0.0905493107727255 | 0.325370099546418 | 0.124 | 0.123 | 1 |
| JMY | 0.0909998405808749 | 0.226317692716158 | 0.073 | 0.078 | 1 |
| TMEM181 | 0.0923756911385591 | 0.447007496062888 | 0.104 | 0.103 | 1 |
| AC002316.1 | 0.0925481568505266 | 0.314846787067753 | 0.157 | 0.17 | 1 |
| SUSD6 | 0.0925632905412396 | 0.459194167767382 | 0.086 | 0.085 | 1 |
| MT1G | 0.0933520504337187 | 0.482991912263566 | 0.084 | 0.09 | 1 |
| KMT2D | 0.0947916460196008 | 0.338521020120005 | 0.079 | 0.085 | 1 |
| STK10 | 0.0953074250600089 | 0.179517130009779 | 0.408 | 0.459 | 1 |
| NIPBL | 0.0953548109193191 | 0.290064069605092 | 0.259 | 0.285 | 1 |
| PRKAG2 | 0.0982411626065086 | 0.445674018346314 | 0.053 | 0.051 | 1 |
| ARID5B | 0.0998402232573687 | 0.306175496585364 | 0.218 | 0.238 | 1 |
| FCHSD2 | 0.0998977177335194 | 0.506057741833319 | 0.092 | 0.091 | 1 |
| WDR7 | 0.100006299301783 | 0.399040874614755 | 0.077 | 0.083 | 1 |
| FBXO34 | 0.100850838434633 | 0.317908980328462 | 0.065 | 0.07 | 1 |

| Gene | P_val | Avg_log2FC | Pct. 1 | Pct. 2 | P_val_adj |
|---|---|---|---|---|---|
| DDHD1 | 0.102395527638067 | 0.415712899342646 | 0.117 | 0.117 | 1 |
| MLXIP | 0.102530901308319 | 0.524973508253596 | 0.054 | 0.052 | 1 |
| ATG16L1 | 0.103714228288629 | 0.290786599419188 | 0.107 | 0.115 | 1 |
| MBTD1 | 0.104828852550176 | 0.46409139965879 | 0.061 | 0.059 | 1 |
| REPS1 | 0.10546302927147 | 0.403276197978419 | 0.074 | 0.08 | 1 |
| PTPRJ | 0.105657057206299 | 0.339560755791451 | 0.234 | 0.259 | 1 |
| DIS3L2 | 0.110659421464579 | 0.343977697996815 | 0.058 | 0.063 | 1 |
| HMBOX1 | 0.111286153994257 | 0.362709915031225 | 0.063 | 0.067 | 1 |
| RUNX1 | 0.115021964989517 | 0.397244386070926 | 0.174 | 0.19 | 1 |
| ARID4B | 0.115063095550572 | 0.311518688549696 | 0.296 | 0.316 | 1 |
| QKI | 0.115154465284948 | 0.550809783516153 | 0.153 | 0.167 | 1 |
| CNOT4 | 0.115223737852659 | 0.323741323498817 | 0.092 | 0.099 | 1 |
| SRGAP2C | 0.115626769432107 | 0.431292118579463 | 0.059 | 0.063 | 1 |
| SLC36A4 | 0.118363863938126 | 0.454714675712499 | 0.059 | 0.063 | 1 |
| POU2F1 | 0.119087338860488 | 0.607330089723618 | 0.057 | 0.055 | 1 |
| DGCR2 | 0.120801609885054 | 0.386867866948122 | 0.064 | 0.069 | 1 |
| MEGF9 | 0.120879725687225 | 0.440712755100838 | 0.105 | 0.113 | 1 |
| CRYBG1 | 0.123853492078683 | 0.555906142131757 | 0.103 | 0.103 | 1 |
| BNIP3L | 0.124166541505497 | 0.407102392336467 | 0.182 | 0.187 | 1 |
| RNASET2 | 0.125890262443471 | 0.406139182079299 | 0.267 | 0.282 | 1 |
| SELL | 0.12621603214827 | 0.42594301146067 | 0.253 | 0.279 | 1 |
| AC245297.4 | 0.129909979483827 | 0.252107631084371 | 0.108 | 0.115 | 1 |
| ENC1 | 0.129954175720517 | 0.216953001410895 | 0.125 | 0.133 | 1 |
| IQSEC1 | 0.135583155950586 | 0.246142182026565 | 0.2 | 0.216 | 1 |
| ZNF407 | 0.137686462854099 | 0.559792490381523 | 0.068 | 0.066 | 1 |
| SMURF2 | 0.142142529943493 | 0.351051730252632 | 0.048 | 0.051 | 1 |
| PIKFYVE | 0.143158446918492 | 0.412239242277457 | 0.058 | 0.056 | 1 |
| WWOX | 0.146269044077717 | 0.697125133970528 | 0.071 | 0.077 | 1 |
| ARHGAP27P1-BPTFP1-KPNA2P3 | 0.147703572513477 | 0.261708360240799 | 0.074 | 0.078 | 1 |
| FYN | 0.148290382884069 | 0.258265690590998 | 0.496 | 0.558 | 1 |
| GPR132 | 0.148343029122662 | 0.456438795878676 | 0.053 | 0.051 | 1 |
| TUT4 | 0.148425328776715 | 0.344115425682488 | 0.185 | 0.201 | 1 |

| Gene | P_val | Avg_log2FC | Pct. 1 | Pct. 2 | P_val_adj |
|---|---|---|---|---|---|
| MTSS1 | 0.153098577006165 | 0.430708346915869 | 0.063 | 0.068 | 1 |
| ODF3B | 0.157823596266062 | 0.380941261613876 | 0.052 | 0.055 | 1 |
| ELL | 0.159215951077995 | 0.372986987332399 | 0.05 | 0.053 | 1 |
| FAM214A | 0.161018706227563 | 0.331041874739612 | 0.114 | 0.122 | 1 |
| RPS6KC1 | 0.161268940816215 | 0.440127890223743 | 0.052 | 0.05 | 1 |
| RGS1 | 0.164578582922239 | 0.489159299156603 | 0.137 | 0.139 | 1 |
| PAM | 0.165308357286706 | 0.624923462326091 | 0.082 | 0.081 | 1 |
| PER1 | 0.168168499443274 | 0.298242257875001 | 0.138 | 0.137 | 1 |
| LRRC37A2 | 0.170386696670368 | 0.250781246979704 | 0.057 | 0.06 | 1 |
| MAF | 0.17312501762005 | 0.366504343581112 | 0.104 | 0.104 | 1 |
| SSH2 | 0.173715718281614 | 0.556805507293015 | 0.198 | 0.208 | 1 |
| MVB12B | 0.17510313551367 | 0.324118118601715 | 0.066 | 0.07 | 1 |
| ERN1 | 0.17892847334992 | 0.359594862590419 | 0.163 | 0.166 | 1 |
| TYMP | 0.180326463977328 | 0.519296222651539 | 0.281 | 0.305 | 1 |
| ERO1B | 0.180346567518323 | 0.355965784445415 | 0.05 | 0.054 | 1 |
| FAM118A | 0.18122745455046 | 0.203070085425688 | 0.095 | 0.1 | 1 |
| RBPJ | 0.181894201001967 | 0.571972924097618 | 0.118 | 0.119 | 1 |
| LGALS3BP | 0.187572013905073 | 0.421646645340683 | 0.083 | 0.082 | 1 |
| RAB11FIP1 | 0.189884477291103 | 0.438552805261426 | 0.098 | 0.105 | 1 |
| VNN2 | 0.190090998556249 | 0.719559790884305 | 0.088 | 0.088 | 1 |
| XPO4 | 0.191163270373111 | 0.407658703359688 | 0.054 | 0.057 | 1 |
| PTPRE | 0.192078482194113 | 0.437415304997791 | 0.202 | 0.221 | 1 |
| LMBR1L | 0.206636855706101 | 0.195836962717358 | 0.084 | 0.088 | 1 |
| XRN1 | 0.212578971295666 | 0.34034088530216 | 0.249 | 0.273 | 1 |
| KDM4C | 0.214477805068564 | 0.388758773368003 | 0.103 | 0.103 | 1 |
| PSAP | 0.21534292100304 | 0.494789684542024 | 0.471 | 0.538 | 1 |
| LINC01934 | 0.215455599327196 | 0.704887659215287 | 0.079 | 0.084 | 1 |
| SGMS1 | 0.21742631450718 | 0.668384210367438 | 0.065 | 0.064 | 1 |
| UBR3 | 0.218135948117428 | 0.459667719453787 | 0.065 | 0.064 | 1 |
| PECAM1 | 0.219168277052506 | 0.387652027269542 | 0.166 | 0.178 | 1 |
| STX12 | 0.220422453110121 | 0.406391753079065 | 0.08 | 0.085 | 1 |
| DENND4C | 0.2207536267014 | 0.292596412077012 | 0.1 | 0.106 | 1 |

| Gene | P_val | Avg_log2FC | Pct.1 | Pct.2 | P_val_adj |
|---|---|---|---|---|---|
| WASHC4 | 0.220784249708617 | 0.478188414584759 | 0.102 | 0.109 | 1 |
| EPB41 | 0.221189064527572 | 0.29617664476027 | 0.324 | 0.362 | 1 |
| PSEN1 | 0.222234110462144 | 0.486779929505036 | 0.076 | 0.075 | 1 |
| DDX60L | 0.223002150001163 | 0.506818936250601 | 0.157 | 0.17 | 1 |
| CDC42SE2 | 0.223444549569566 | 0.291150854143882 | 0.437 | 0.49 | 1 |
| CPEB4 | 0.224132147823554 | 0.563366193938371 | 0.072 | 0.077 | 1 |
| FBXO11 | 0.224397486891449 | 0.321870013049407 | 0.138 | 0.147 | 1 |
| XRRA1 | 0.226066868486749 | 0.416601572170484 | 0.048 | 0.051 | 1 |
| CBFA2T2 | 0.226676199095783 | 0.389805018104539 | 0.061 | 0.059 | 1 |
| ITGA4 | 0.227211598692725 | 0.134684370998175 | 0.575 | 0.656 | 1 |
| MAP3K3 | 0.228704688792692 | 0.352781248348263 | 0.125 | 0.133 | 1 |
| RASSF2 | 0.23018529283546 | 0.615048902386964 | 0.074 | 0.079 | 1 |
| VPS13D | 0.232427963674819 | 0.279672861041642 | 0.102 | 0.107 | 1 |
| GPATCH2L | 0.237809625498595 | 0.351980424577512 | 0.07 | 0.074 | 1 |
| SGSM2 | 0.238094741231496 | 0.278194505298909 | 0.063 | 0.066 | 1 |
| ANKRD12 | 0.245037164940149 | 0.318610880110058 | 0.3 | 0.319 | 1 |
| ATXN1 | 0.245333568767638 | 0.452677796824887 | 0.238 | 0.253 | 1 |
| IL4R | 0.245707884721169 | 0.510588972234379 | 0.084 | 0.09 | 1 |
| SCLT1 | 0.24633737086292 | 0.462331822556406 | 0.163 | 0.176 | 1 |
| PIK3CA | 0.246769557131731 | 0.430592008548526 | 0.06 | 0.063 | 1 |
| KANSL1 | 0.249172156388457 | 0.423917599145936 | 0.164 | 0.177 | 1 |
| KMT2E | 0.250413795662033 | 0.204400229048746 | 0.4 | 0.448 | 1 |
| YES1 | 0.256300607356358 | 0.522369672376159 | 0.057 | 0.056 | 1 |
| TMEM140 | 0.25658049377085 | 0.367907420620756 | 0.051 | 0.054 | 1 |
| BLVRB | 0.263208579000965 | 0.653280935611076 | 0.07 | 0.069 | 1 |
| NFE2L2 | 0.264719986387085 | 0.399593470392327 | 0.168 | 0.173 | 1 |
| N4BP2L2 | 0.265152451005555 | 0.330146807866071 | 0.25 | 0.264 | 1 |
| ARID2 | 0.265936322357254 | 0.359023319775817 | 0.118 | 0.126 | 1 |
| HDAC5 | 0.267024729948617 | 0.162978980993593 | 0.062 | 0.065 | 1 |
| TMEM41B | 0.269104866654172 | 0.454187789523774 | 0.057 | 0.056 | 1 |
| RELL1 | 0.269986766301494 | 0.461693706662127 | 0.059 | 0.063 | 1 |
| SOD2 | 0.278002134178566 | 0.798600579419397 | 0.087 | 0.093 | 1 |

| Gene | P_val | Avg_log2FC | Pct.1 | Pct.2 | P_val_adj |
|---|---|---|---|---|---|
| MAPK8IP3 | 0.284968899440119 | 0.236045749095618 | 0.107 | 0.112 | 1 |
| SENP7 | 0.286770903788699 | 0.352466655603916 | 0.094 | 0.099 | 1 |
| SLC4A7 | 0.287088926680445 | 0.362963071547284 | 0.14 | 0.149 | 1 |
| CALCOCO1 | 0.292095978380361 | 0.221070246363167 | 0.146 | 0.155 | 1 |
| IL6ST | 0.292590244468746 | 0.451494380175304 | 0.106 | 0.112 | 1 |
| VPS13C | 0.299085127599439 | 0.255427146469331 | 0.334 | 0.369 | 1 |
| SMG6 | 0.302768086745211 | 0.346544716024746 | 0.073 | 0.077 | 1 |
| LCORL | 0.305505657099462 | 0.293589890475415 | 0.064 | 0.067 | 1 |
| USP3 | 0.313468022797659 | 0.491629467043109 | 0.182 | 0.191 | 1 |
| GRAMD1A | 0.31392935666847 | 0.300909548048164 | 0.083 | 0.087 | 1 |
| WDR26 | 0.322875503760836 | 0.3075056279786 | 0.197 | 0.211 | 1 |
| VPS54 | 0.329769721519265 | 0.45446564240569 | 0.073 | 0.077 | 1 |
| MARCHF6 | 0.338846060455297 | 0.345949438168129 | 0.151 | 0.161 | 1 |
| KLHL5 | 0.343680498675623 | 0.53313396827911 | 0.05 | 0.053 | 1 |
| ANKRD11 | 0.347532724697774 | 0.283136536226812 | 0.307 | 0.337 | 1 |
| BRWD3 | 0.350770905425517 | 0.519488552203115 | 0.068 | 0.067 | 1 |
| ITSN2 | 0.351838940399255 | 0.36956230014164 | 0.249 | 0.273 | 1 |
| SLC9A8 | 0.357563782385987 | 0.458093795473642 | 0.069 | 0.072 | 1 |
| ELMSAN1 | 0.362440936145903 | 0.370604726710565 | 0.053 | 0.055 | 1 |
| DENND4A | 0.362594075562269 | 0.368912292632297 | 0.119 | 0.127 | 1 |
| KDM6A | 0.371031134924931 | 0.493610347494561 | 0.11 | 0.112 | 1 |
| MAP3K2 | 0.376411489804605 | 0.431046677323318 | 0.156 | 0.167 | 1 |
| DGKA | 0.37666364806925 | 0.43023861812621 | 0.134 | 0.137 | 1 |
| CLIP4 | 0.380857941144082 | 0.322318614615429 | 0.069 | 0.072 | 1 |
| AFF4 | 0.381829507113959 | 0.490971813293251 | 0.138 | 0.142 | 1 |
| NBPF14 | 0.382570556977135 | 0.269006852753929 | 0.064 | 0.063 | 1 |
| NUMB | 0.385218994273346 | 0.632978975715039 | 0.089 | 0.09 | 1 |
| KDM4B | 0.386036811839141 | 0.254196336151717 | 0.068 | 0.067 | 1 |
| ZCCHC2 | 0.388946455666946 | 0.451791758444436 | 0.094 | 0.094 | 1 |
| TNFAIP3 | 0.389755576297143 | 0.144680242024381 | 0.454 | 0.489 | 1 |
| PDCD4 | 0.391902388782268 | 0.311359053773979 | 0.206 | 0.215 | 1 |
| RELCH | 0.394023877618522 | 0.316089648346348 | 0.107 | 0.112 | 1 |

| Gene | P_val | Avg_log2FC | Pct. 1 | Pct. 2 | P_val_adj |
|---|---|---|---|---|---|
| PHKB | 0.401956562671316 | 0.435178497630123 | 0.089 | 0.094 | 1 |
| ITM2B | 0.402932675880904 | 0.203222843750131 | 0.59 | 0.673 | 1 |
| PPP1R15A | 0.403653880318316 | 0.451171296428996 | 0.19 | 0.198 | 1 |
| SCPEP1 | 0.404195323278407 | 0.761702050333736 | 0.053 | 0.053 | 1 |
| FAM120B | 0.407204374577889 | 0.426046350044327 | 0.07 | 0.073 | 1 |
| BICDL1 | 0.409226920608802 | 0.467590811371724 | 0.079 | 0.079 | 1 |
| MXD1 | 0.419986309500646 | 0.69253942530913 | 0.117 | 0.12 | 1 |
| AVL9 | 0.420137141504985 | 0.431879065865979 | 0.062 | 0.065 | 1 |
| ACER3 | 0.420179228486038 | 0.633391590199462 | 0.054 | 0.054 | 1 |
| RIC1 | 0.421420227384309 | 0.528740060649398 | 0.066 | 0.066 | 1 |
| TRIM22 | 0.4214631333385 | 0.242717312544667 | 0.364 | 0.404 | 1 |
| TBCK | 0.42468480994005 | 0.365847672055332 | 0.054 | 0.056 | 1 |
| CD84 | 0.431887651456146 | 0.327359853774744 | 0.153 | 0.157 | 1 |
| SAMSN1 | 0.432463621611462 | 0.545976812088848 | 0.22 | 0.235 | 1 |
| TBL1X | 0.437907336172665 | 0.438506352989273 | 0.088 | 0.089 | 1 |
| DENND3 | 0.442470806404965 | 0.289420885321685 | 0.092 | 0.096 | 1 |
| USP25 | 0.443720923656723 | 0.446495847452434 | 0.17 | 0.177 | 1 |
| INPP4B | 0.44404195752653 | 0.518755317760875 | 0.118 | 0.121 | 1 |
| PDE7A | 0.447274847102671 | 0.310051734087302 | 0.111 | 0.117 | 1 |
| FCRL3 | 0.44749769395868 | 0.320764525761289 | 0.054 | 0.053 | 1 |
| HIVEP1 | 0.44755714270493 | 0.528862869050511 | 0.057 | 0.057 | 1 |
| KDM2A | 0.449240876202042 | 0.31182640905973 | 0.208 | 0.224 | 1 |
| RALGAPA1 | 0.455490158060749 | 0.368555226228619 | 0.097 | 0.101 | 1 |
| RNF38 | 0.4610346909019 | 0.43600592822233 | 0.125 | 0.128 | 1 |
| SAMD8 | 0.465388317108923 | 0.439347676089306 | 0.063 | 0.063 | 1 |
| LRCH3 | 0.476709644633138 | 0.381309386752483 | 0.075 | 0.075 | 1 |
| RNF19A | 0.484516270260384 | 0.291604847848026 | 0.305 | 0.329 | 1 |
| CAMK2D | 0.484544624421416 | 0.510467402327019 | 0.113 | 0.116 | 1 |
| TC2N | 0.488586275275234 | 0.362366230226056 | 0.232 | 0.246 | 1 |
| FRS2 | 0.488716712851574 | 0.5180433935274 | 0.052 | 0.055 | 1 |
| USP34 | 0.492375838507936 | 0.383053997486676 | 0.217 | 0.234 | 1 |
| TP53INP1 | 0.496760033458854 | 0.292188706358469 | 0.052 | 0.054 | 1 |

| Gene | P_val | Avg_log2FC | Pct.1 | Pct.2 | P_val_adj |
|---|---|---|---|---|---|
| CYTH1 | 0.501043056695425 | 0.254014084946176 | 0.366 | 0.407 | 1 |
| JAK2 | 0.507273515358338 | 0.368826103560959 | 0.175 | 0.188 | 1 |
| MKLN1 | 0.517561691940256 | 0.43829122129271 | 0.12 | 0.123 | 1 |
| ULK1 | 0.519039476333722 | 0.422808586385322 | 0.059 | 0.059 | 1 |
| CCDC18-AS1 | 0.522496134968415 | 0.291225168180499 | 0.134 | 0.141 | 1 |
| SFMBT2 | 0.523112762992521 | 0.520537208998414 | 0.074 | 0.074 | 1 |
| JARID2 | 0.523922831966378 | 0.484162620380912 | 0.144 | 0.154 | 1 |
| ITPR1 | 0.528543803392221 | 0.494581694349064 | 0.059 | 0.062 | 1 |
| TNKS | 0.532285438351052 | 0.425202561598841 | 0.081 | 0.084 | 1 |
| USP9Y | 0.537279297816622 | 0.405585248605521 | 0.088 | 0.089 | 1 |
| AC044849.1 | 0.538936195430923 | 0.293618650433053 | 0.067 | 0.069 | 1 |
| MICAL1 | 0.540188914822298 | 0.275902855483797 | 0.09 | 0.093 | 1 |
| PHF21A | 0.544988858285759 | 0.355509714478022 | 0.165 | 0.175 | 1 |
| ZFP36L1 | 0.54773347815937 | 0.467852446419415 | 0.199 | 0.215 | 1 |
| NFKB2 | 0.551262464812292 | 0.329870845604662 | 0.067 | 0.069 | 1 |
| TET2 | 0.558086175061335 | 0.481817263106754 | 0.127 | 0.131 | 1 |
| GARS-DT | 0.559601341989079 | 0.399945179337923 | 0.076 | 0.079 | 1 |
| CDKAL1 | 0.569558491174783 | 0.492035370462401 | 0.088 | 0.09 | 1 |
| DOCK5 | 0.574395810105976 | 0.428582024479259 | 0.108 | 0.113 | 1 |
| DDX17 | 0.576910401425635 | 0.229513331122232 | 0.395 | 0.441 | 1 |
| MSI2 | 0.577870847401809 | 0.478017231524436 | 0.104 | 0.107 | 1 |
| FNDC3B | 0.579028867330711 | 0.572171444822508 | 0.124 | 0.131 | 1 |
| HECTD4 | 0.583903161133981 | 0.284079164308735 | 0.092 | 0.096 | 1 |
| MBD5 | 0.601933213488183 | 0.416850851449875 | 0.11 | 0.115 | 1 |
| SPOPL | 0.61181277105495 | 0.457304549372307 | 0.066 | 0.069 | 1 |
| IP6K1 | 0.621651807494767 | 0.409580049820926 | 0.066 | 0.067 | 1 |
| ZNF831 | 0.639532928221541 | 0.364958265365381 | 0.081 | 0.084 | 1 |
| BRI3 | 0.640800582514595 | 0.620147714216317 | 0.231 | 0.254 | 1 |
| SDCBP | 0.642203486209264 | 0.598776076567992 | 0.186 | 0.197 | 1 |
| PIK3C2A | 0.643078043949142 | 0.384665895223357 | 0.08 | 0.083 | 1 |
| KIAA0319L | 0.653144211894969 | 0.368266788497716 | 0.112 | 0.117 | 1 |
| AKAP9 | 0.662314481587228 | 0.287017147888352 | 0.266 | 0.287 | 1 |

| Gene | P_val | Avg_log2FC | Pct. 1 | Pct. 2 | P_val_adj |
|---|---|---|---|---|---|
| ZMYM2 | 0.666616733662722 | 0.418806172828089 | 0.138 | 0.143 | 1 |
| NF1 | 0.681757464005822 | 0.333459549821525 | 0.099 | 0.103 | 1 |
| TNRC6C | 0.683450474776086 | 0.341151186903294 | 0.065 | 0.065 | 1 |
| NOTCH2 | 0.684356370671582 | 0.505454688441389 | 0.068 | 0.069 | 1 |
| KDM5D | 0.689174903141033 | 0.286374485028903 | 0.07 | 0.072 | 1 |
| LMBR1 | 0.68979833391873 | 0.469851335602165 | 0.066 | 0.068 | 1 |
| PLCD1 | 0.691006688144268 | 0.392492924838992 | 0.053 | 0.055 | 1 |
| VWA8 | 0.699896480382727 | 0.544861906351919 | 0.051 | 0.051 | 1 |
| TENT5A | 0.700598793837135 | 0.451296450696574 | 0.05 | 0.05 | 1 |
| MIAT | 0.709355174105549 | 0.327401101848334 | 0.167 | 0.172 | 1 |
| SPG11 | 0.714910711460147 | 0.412880313390575 | 0.127 | 0.133 | 1 |
| QSOX1 | 0.717933248210675 | 0.453278040486881 | 0.051 | 0.051 | 1 |
| CLASP2 | 0.718088656751345 | 0.426323703211313 | 0.089 | 0.093 | 1 |
| OGT | 0.720283669183218 | 0.245445614900107 | 0.324 | 0.351 | 1 |
| MAP3K8 | 0.723526928754882 | 0.294595017751838 | 0.284 | 0.309 | 1 |
| DOCK8 | 0.725669704724372 | 0.414236247628479 | 0.293 | 0.327 | 1 |
| NINJ1 | 0.733909278320367 | 0.503520033000189 | 0.122 | 0.128 | 1 |
| PELI1 | 0.738149770731293 | 0.499178302391012 | 0.136 | 0.141 | 1 |
| MT1X | 0.745076688981247 | 0.362037865235992 | 0.379 | 0.421 | 1 |
| ASAH1 | 0.747727683332829 | 0.63371530044407 | 0.134 | 0.14 | 1 |
| SYNJ1 | 0.753282606332082 | 0.396187848854474 | 0.06 | 0.061 | 1 |
| NBPF26 | 0.757691336608711 | 0.312168810496033 | 0.077 | 0.078 | 1 |
| ATG2A | 0.760118064250017 | 0.299129241939429 | 0.153 | 0.159 | 1 |
| CHD2 | 0.762746937424107 | 0.324927120413949 | 0.221 | 0.237 | 1 |
| SLCO3A1 | 0.763879170123667 | 0.535824964997045 | 0.111 | 0.115 | 1 |
| PHF1 | 0.764739863938065 | 0.289363347293364 | 0.093 | 0.094 | 1 |
| RB1CC1 | 0.765179438640279 | 0.295821392494363 | 0.184 | 0.195 | 1 |
| DOP1A | 0.767095228767209 | 0.339136942559148 | 0.061 | 0.061 | 1 |
| HIPK3 | 0.768472357481137 | 0.495779601420698 | 0.079 | 0.082 | 1 |
| CD44 | 0.770674743528481 | 0.308409584861197 | 0.37 | 0.415 | 1 |
| RABGAP1 | 0.776327641842759 | 0.467343353329287 | 0.081 | 0.084 | 1 |
| DCTN4 | 0.779103450807977 | 0.477052750826846 | 0.065 | 0.067 | 1 |

| Gene | P_val | Avg_log2FC | Pct.1 | Pct.2 | P_val_adj |
|---|---|---|---|---|---|
| LMTK2 | 0.7807263543739 | 0.41901919279834 | 0.065 | 0.066 | 1 |
| PCNX2 | 0.784773234916211 | 0.59061854268705 | 0.061 | 0.062 | 1 |
| ABCC1 | 0.789498204849101 | 0.345484497357861 | 0.099 | 0.101 | 1 |
| ZFP36L2 | 0.802698054458968 | 0.177824366350094 | 0.497 | 0.561 | 1 |
| INO80D | 0.81031001222661 | 0.35993824289233 | 0.079 | 0.082 | 1 |
| CDK19 | 0.825892598981414 | 0.476021406022414 | 0.055 | 0.056 | 1 |
| CLASP1 | 0.831753972826877 | 0.434626520473014 | 0.085 | 0.088 | 1 |
| TASOR2 | 0.835875337964651 | 0.460724980568638 | 0.082 | 0.084 | 1 |
| POU2F2 | 0.847975543747888 | 0.414095497639875 | 0.078 | 0.081 | 1 |
| TAB2 | 0.848467108046953 | 0.413478357147572 | 0.131 | 0.136 | 1 |
| TTC39B | 0.853039686300231 | 0.297842253888273 | 0.071 | 0.073 | 1 |
| ABLIM1 | 0.861916879032236 | 0.427602133284845 | 0.102 | 0.105 | 1 |
| TMCC1 | 0.86223899890424 | 0.496716978545483 | 0.049 | 0.05 | 1 |
| TULP4 | 0.871528894614245 | 0.441126598974816 | 0.062 | 0.063 | 1 |
| TLE4 | 0.873590838064971 | 0.444977023722711 | 0.104 | 0.108 | 1 |
| PIAS1 | 0.876978344022521 | 0.441841612050993 | 0.178 | 0.187 | 1 |
| PKN2 | 0.877575472607201 | 0.422376367591812 | 0.138 | 0.144 | 1 |
| SARAF | 0.881206883690295 | 0.193093607700661 | 0.503 | 0.579 | 1 |
| PARP8 | 0.887311953672273 | 0.287276566861216 | 0.316 | 0.343 | 1 |
| XYLT1 | 0.89747552920431 | 0.305382611243555 | 0.06 | 0.062 | 1 |
| WAKMAR2 | 0.90862099590563 | 0.333463132358724 | 0.056 | 0.057 | 1 |
| MINK1 | 0.917034062548756 | 0.324112613316492 | 0.118 | 0.121 | 1 |
| SEC24B | 0.917559859689797 | 0.395172816602479 | 0.098 | 0.1 | 1 |
| GMDS-DT | 0.923651439849892 | 0.516072582280376 | 0.059 | 0.06 | 1 |
| WSB1 | 0.923727991934853 | 0.391699280300206 | 0.206 | 0.219 | 1 |
| SENP5 | 0.930648912132026 | 0.45525165757585 | 0.101 | 0.104 | 1 |
| CD55 | 0.931075636885335 | 0.488232859175852 | 0.236 | 0.256 | 1 |
| ZNRF2 | 0.935927465048775 | 0.452012012006516 | 0.073 | 0.075 | 1 |
| ARID5A | 0.9420449557847 | 0.239712921246883 | 0.171 | 0.177 | 1 |
| SMAD3 | 0.942095159903252 | 0.476532170677518 | 0.12 | 0.126 | 1 |
| ITFG1 | 0.945538539674569 | 0.544942000387171 | 0.122 | 0.128 | 1 |
| DNAH1 | 0.948172012667217 | 0.365892779482743 | 0.051 | 0.051 | 1 |

| Gene | P_val | Avg_log2FC | Pct.1 | Pct.2 | P_val_adj |
|---|---|---|---|---|---|
| BICRAL | 0.950435287933217 | 0.411846137525961 | 0.103 | 0.107 | 1 |
| CMIP | 0.955589877195789 | 0.284032524565733 | 0.186 | 0.195 | 1 |
| STXBP5 | 0.956057099922879 | 0.476358037577123 | 0.082 | 0.084 | 1 |
| USP20 | 0.962007057334192 | 0.430046332377155 | 0.07 | 0.071 | 1 |
| PRKY | 0.966740844630793 | 0.333921701180626 | 0.109 | 0.112 | 1 |
| HECA | 0.97474969438093 | 0.357924662556053 | 0.182 | 0.191 | 1 |
| TSC22D3 | 0.983290163427979 | 0.136871755122713 | 0.488 | 0.533 | 1 |
| UNC93B1 | 0.988533570653219 | 0.30396654691177 | 0.053 | 0.054 | 1 |
| P2RX4 | 0.994214410615291 | 0.483282403228801 | 0.049 | 0.05 | 1 |
| PDCD1 | 0.99979358213576 | 0.531832832126708 | 0.062 | 0.064 | 1 |

Supplementary Figure S1：Sampling schedule for different patients. Blue indicates the sampling time.

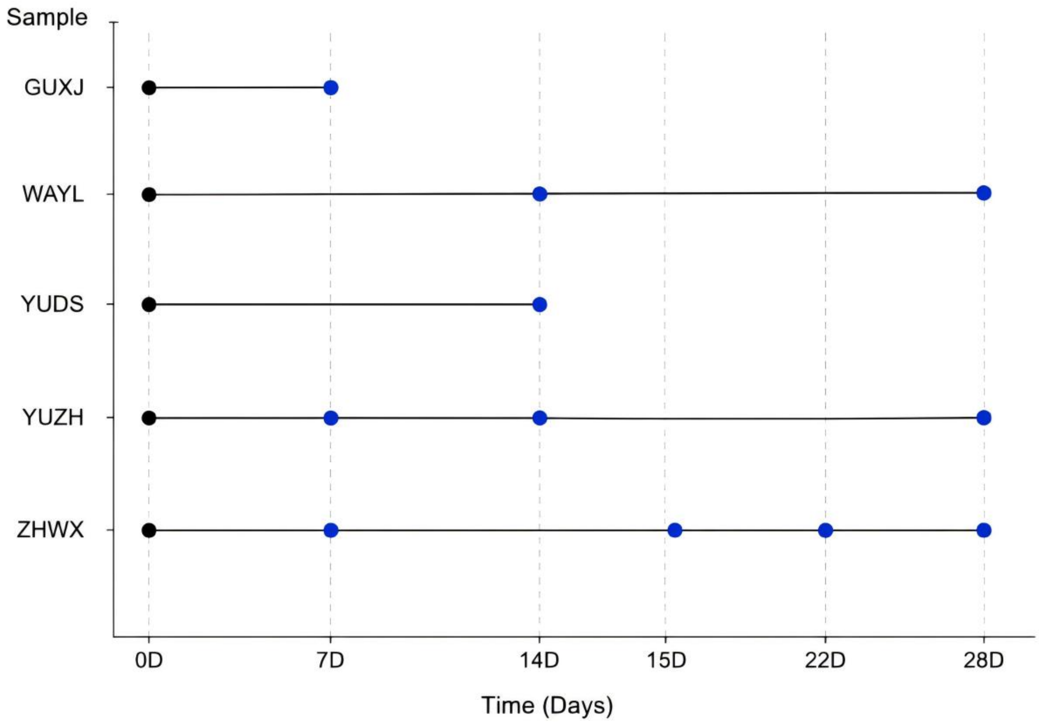

Supplement: Supplementary file 1 [file genes-17-01272-s001.zip › genes-4530963-supplementary.pdf]
